# Comparative efficacy of 5-hydroxytryptamine-3 (5-HT3) receptor antagonists with or without dexamethasone for prevention of chemotherapy-induced nausea and vomiting following highly emetogenic chemotherapy (HEC): a network meta-analysis

**DOI:** 10.7717/peerj.21047

**Published:** 2026-04-02

**Authors:** Hongxia Xu, Jiankun Xing, Shaohui Yang, Lingyan Rong, Lingyan Liu, Xiaotao Chen

**Affiliations:** 1 Department of Clinical Pharmacy, Wendeng Hospital of Traditional Chinese Orthopedics and Traumatology of Shandong Province, Wendeng District, Weihai City, Shandong Province, China; 2 Rehabilitation Department, Wendeng Hospital of Traditional Chinese Orthopedics and Traumatology of Shandong Province, Wendeng District, Weihai City, Shandong Province, China; 3 Department of Anesthesiology, Wendeng Hospital of Traditional Chinese Orthopedics and Traumatology of Shandong Province, Wendeng District, Weihai City, Shandong Province, China

**Keywords:** Highly emetogenic chemotherapy-induced nausea and vomiting, 5-HT3 antagonists (with dexamethasone or not), Nausea and vomiting, Highly emetogenic chemotherapy, 5-HT3 antagonists, With dexamethasone or not

## Abstract

**Objective:**

This network meta-analysis evaluated the efficacy of 5-hydroxytryptamine-3 (5-HT3) receptor antagonists, with or without Dexamethasone (D), for preventing chemotherapy-induced nausea and vomiting (CINV) in patients undergoing highly emetogenic chemotherapy (HEC) who were limited to these regimens.

**Methods:**

Randomized controlled studies (RCTs) were searched in PubMed, Embase, Cochrane and Web of Science from their inception up to January 28, 2026 (a supplementary search was conducted from April 1, 2025, to January 28, 2026), to identify studies on patients who used 5-HT3 receptor antagonists (with or without Dexamethasone) to prevent nausea and vomiting caused by highly emetogenic chemotherapy. A total of 36 randomized controlled trials (reported in 37 articles) enrolling 11,131 patients were included in this analysis. The outcome measures included acute nausea, acute vomiting, acute complete control, delayed nausea, delayed vomiting and delayed complete control.

**Results:**

Palonosetron (P) is generally more effective than first-generation 5-HT3 receptor antagonists (1^st^ 5-HT3 antagonists), with a significantly greater advantage over Ondansetron in the delayed phase (such as in delayed vomiting (20 trials/6,347 patients) (RR 1.47, 95% CI [1.03–2.08]). In all phases, adding Dexamethasone to 1^st^ 5-HT3 antagonists significantly improved efficacy compared to using them alone. However, combining Dexamethasone with palonosetron showed no significant advantage over Palonosetron alone in any outcome measure in indirect comparisons. In the delayed phase [such as in delayed vomiting (20 trials/6,347 patients)), Palonosetron + Dexamethasone (P+D) demonstrated statistically significant superiority over both Ondansetron + Dexamethasone (O+D) (RR 1.35, 95% CI [1.14–1.60]) and Granisetron + Dexamethasone (G+D) (RR 1.52, 95% CI [1.22–1.91]). However, in the acute phase, Palonosetron + Dexamethasone showed statistically significant superiority over Ondansetron + Dexamethasone (RR 0.81, 95% CI [0.69–0.96]) only in preventing acute vomiting (23 trials/9096 patients). In contrast, no significant efficacy differences were observed between Palonosetron + Dexamethasone and Tropisetron + Dexamethasone (T+D), or Ramosetron + Dexamethasone (R+D). No significant differences were observed between Palonosetron and 1^st^ 5-HT3 antagonists + Dexamethasone.

**Conclusions:**

Dexamethasone may enhance the efficacy of first-generation 5-HT_3_ receptor antagonists, but may not provide a similar benefit for Palonosetron. Palonosetron is generally more effective than first-generation 5-HT3 antagonists. Palonosetron + Dexamethasone demonstrates superior efficacy over Ondansetron + Dexamethasone or Granisetron + Dexamethasone, particularly in the delayed phase. Further studies are needed between Palonosetron + Dexamethasone and Tropisetron + Dexamethasone or Ramosetron + Dexamethasone, as well as between Palonosetron monotherapy and 1^st^ 5-HT3 antagonists + Dexamethasone.

## Introduction

Chemotherapy-induced nausea and vomiting (CINV) are highly prevalent among cancer patients (Badarudin et al., 2022). As a clinically significant adverse effect, CINV frequently leads to chemotherapy discontinuation due to its substantial detrimental impact on patient quality of life (Navari & Aapro, 2016; National Comprehensive Cancer Network (NCCN), 2025). Various chemotherapy agents cause different levels of emesis (Grunberg et al., 2011). Emetogenicity is divided into four categories: high, moderate, low, and minimal. Without antiemetic treatment, over 90% of patients undergoing highly emetogenic chemotherapy (HEC) may experience CINV (National Comprehensive Cancer Network (NCCN), 2025).

Currently, guidelines recommend three or four regimens for HEC, including 5-HT_3_ receptor antagonist (5-HT_3_RA), Dexamethasone (D), Neurokinin 1 receptor antagonist (NK_1_RA) or Olanzapine (National Comprehensive Cancer Network (NCCN), 2025; Hesketh et al., 2016; Roila et al., 2016). However, suboptimal adherence to guidelines was observed in highly emetogenic chemotherapy. Specifically, the guideline-recommended regimen consisting of NK1RA, 5-HT3RA, and Dexamethasone was prescribed to only 12.2%–15% of patients, while the four-drug combination that includes Olanzapine in addition to these agents was used in merely 2.1% of cases (Aapro et al., 2021; Dranitsaris et al., 2017; Scotté, 2017). Guideline adherence in clinical practice has shown a sluggish advancement in recent periods (Navari et al., 2019). Several factors contribute to the unsatisfactory compliance with the guidelines: a lack of awareness of antiemetic guidelines among physicians (Aapro et al., 2021), the limited availability of certain drugs (Andrianandrasana et al., 2023), financial constraints in developing countries, especially in rural areas (Dulal et al., 2019; Du et al., 2017), and the exclusion of newer antiemetic agents from national medical insurance formularies (Sun et al., 2021). 5-HT_3_ antagonists play a critical role in antiemetic therapy in chemotherapy patients (National Comprehensive Cancer Network (NCCN), 2025). Dexamethasone and 5-HT_3_ antagonists are used by most patients receiving HEC (O’Sullivan et al., 2018).

Prophylaxis with 5-HT_3_ receptor antagonists and Dexamethasone, whether used as monotherapy or in combination, is insufficient for the prevention of CINV following HEC. However, owing to a variety of constraints (Aapro et al., 2021; Andrianandrasana et al., 2023; Dulal et al., 2019; Du et al., 2017; Sun et al., 2021), patients access to antiemetic therapy is often limited to a narrow range of options.

In such scenarios, a more detailed comparison of the efficacy of various 5-HT_3_ antagonists—both as monotherapy and in combination with Dexamethasone—may be needed: for example, although Palonosetron is widely recognized as exhibiting superior efficacy compared to first-generation 5-HT_3_ antagonists (1^st^ 5-HT_3_ antagonists) (National Comprehensive Cancer Network (NCCN), 2025), it remains unclear more detail question, such as , whether Palonosetron monotherapy outperforms a regimen combining a 1^st^ 5-HT_3_ antagonists with Dexamethasone? What is the magnitude of the enhancement provided by Dexamethasone to the antiemetic effect of 5-HT_3_ antagonists in highly emetogenic chemotherapy regimens? Is it possible to determine which treatment regimen holds a superior therapeutic advantage over others in highly emetogenic chemotherapy?

However, existing network meta-analyses fail to provide the comparative conclusions outlined above:some studies have evaluated all first-generation 5-HT_3_ receptor antagonists as a unified class rather than comparing them individually (Filetti et al., 2023; Cheng et al., 2018; Yokoe et al., 2019); some analyses despite comparing different 5-HT_3_ antagonists separately, did not encompass all the available 5-HT_3_ antagonists (Piechotta et al., 2021); some were limited to monotherapy comparisons of 5-HT_3_ antagonists without evaluating combination regimens with Dexamethasone (Sun et al., 2025); while others were limited by restricted patients (only pediatric patients included) inclusion criteria (Walker, Dias & Phillips, 2024). So, we will perform a network meta-analysis to systematically compare the efficacy of all available 5-HT_3_ receptor antagonists, both as monotherapies and in combination with dexamethasone undergoing highly emetogenic chemotherapy. The analysis will enable direct and indirect comparisons across these regimens to address gaps in the current evidence.Based on the current situation where a significant proportion of patients continue to receive only 5-HT_3_ antagonists (with or without Dexamethasone) for HEC, on the one hand, it is necessary to take a variety of measures to push physicians to adhere to the guidelines by selecting from the recommended combinations of three or four antiemetic drugs for HEC, while a refined selection program of 5-HT_3_ antagonists (with or without Dexamethasone) used in HEC should be provided for clinical reference. This study aims to conduct a comprehensive network meta-analysis comparing the efficacy of individual 5-HT_3_ receptor antagonists, with or without Dexamethasone, for the prevention of nausea, vomiting, and complete control in patients undergoing highly emetogenic chemotherapy. The objective is to provide optimized antiemetic regimens for patients who, due to various constraints, can only receive 5-HT_3_ antagonists (with or without Dexamethasone) for the prevention of nausea and vomiting during HEC.

## Materials & Methods

### Methods

#### Registration

This network meta-analysis was performed in accordance with the Preferred Reporting Items for Systematic Reviews and Meta-Analyses (PRISMA) guidelines (Liberati et al., 2009). The protocol for this analysis was registered under the reference CRD42023403570.

#### Search strategy

Four electronic databases, including Pubmed, Embase, Cochrane, and Web of Science, were systematically searched from their inception up to January 28, 2026. (A supplementary search was performed from April 1, 2025, to January 28, 2026.)

Two reviewers (Hongxia Xu and Jiankun Xing) independently worked on the study of identification, selection, quality assessment and data abstraction. A third reviewer (Shaohui Yang) was consulted for any discrepancies. The search strategy involved the use of medical subject headings (MeSH) such as Ondansetron (O), Granisetron (G), Dolasetron (Do), Tropisetron (T), Ramosetron (R), Azasetron (A), Palonosetron (P) and text words (Supplemental Information 12). The search strategy used in PubMed was as followed: “Ondansetron” [Mesh] OR Ondansetron, (+,-)-Isomer [Title/Abstract] OR Ondansetron Hydrochloride [Title/Abstract] OR Hydrochloride, Ondansetron [Title/Abstract] OR Ondansetron Monohydrochloride [Title/Abstract] OR Monohydrochloride, Ondansetron [Title/Abstract] OR Ondansetron Monohydrochloride Dihydrate [Title/Abstract] OR Dihydrate, Ondansetron Monohydrochloride [Title/Abstract] OR Monohydrochloride Dihydrate, Ondansetron [Title/Abstract] OR Ondansetron, (S)-Isomer [Title/Abstract] OR Zofran [Title/Abstract] OR Ondansetron, (R)-Isomer [Title/Abstract] OR “Granisetron” [Mesh] OR Kytril [Title/Abstract] OR Granisetron Hydrochloride [Title/Abstract] OR Hydrochloride, Granisetron [Title/Abstract]) OR (Granisetron Monohydrochloride [Title/Abstract] OR Monohydrochloride, Granisetron [Title/Abstract] OR “dolasetron” [Supplementary Concept] OR dolasetron mesylate [Title/Abstract] OR dolasetron mesylate monohydrate [Title/Abstract] OR dolasetronmesilate monohydrate [Title/Abstract] OR Anzemet [Title/Abstract] OR “Tropisetron” [Mesh] OR Navoban [Title/Abstract] OR Indole 3 carboxylic Acid Tropine Ester [Title/Abstract] OR Tropisetron Hydrochloride [Title/Abstract] OR “ramosetron” [Supplementary Concept] OR ramosetron hydrochloride [Title/Abstract] OR Nasea [Title/Abstract] OR (“azasetron” [Supplementary Concept]) OR (“azasetron” [Supplementary Concept] OR “azasetron” [All Fields]) AND “isomer” [Title/Abstract]) OR “Palonosetron” [Mesh]) OR Palonosetron, (R-(R*,R*))-isomer [Title/Abstract] OR Palonosetron, 3R [Title/Abstract] OR Palonosetron, (R-(R*,S*))-isomer [Title/Abstract] OR Aloxi [Title/Abstract] OR Palonosetron, (S-(R*,S*))-isomer [Title/Abstract] OR Palonosetron Hydrochloride [Title/Abstract].

#### Study selection criteria

Study inclusion criteria

(1) Patients receiving highly emetogenic chemotherapy (HEC) who were administered 5-HT_3_ antagonists, with or without Dexamethasone, for the prevention of nausea and vomiting were included.

(2) Only randomized controlled trials (RCTs) were included.

(3) Only English literature was included.

Study exclusion criteria

(1) Patients who received low, moderate, or mixed emetogenic chemotherapy regimens were excluded and the trials that reported radiotherapy-induced nausea and vomiting were excluded.

(2) Patients who received 5-HT_3_ antagonists with other antiemetic drugs except for Dexamethasone, were excluded.

(3) Non-RCTs, animal experiments, conference abstracts, protocols, reviews, letters, retrospective studies were excluded.

#### Outcomes measures

I. Acute nausea (<24 h post-chemotherapy after the first chemotherapy dose)

II. Acute vomiting (<24 h post-chemotherapy after the first chemotherapy dose)

III. Acute complete control (<24 h post-chemotherapy after the first chemotherapy dose)

In this article, complete control (CC) was defined as no emetic episodes, no use of rescue medication, and experiencing only mild or no nausea.

IV. Delayed nausea (≥24 h post-chemotherapy after the first chemotherapy dose)

V. Delayed vomiting (≥24 h post-chemotherapy after the first chemotherapy dose)

VI. Delayed complete control (≥24 h post-chemotherapy after the first chemotherapy dose)

#### Risk of bias assessment

The risk of bias in the study was assessed by the Cochrane handbook (Version 5.3.5) (Higgins, Green & The Cochrane Collaboration, 2018), using the Cochrane risk of bias tool designed for RCTs, which including the indicators of sequence generation, allocation concealment, blinding of participants and outcome assessment, incomplete or selective outcome reporting. The risk of bias was assessed independently by two authors (Hongxia Xu and Jiankun Xing). Any discrepancies between their assessments were resolved by consultation with a third author (Shaohui Yang).

#### Data extraction

The selected literature has been imported into EndNote for systematic management and organization.Two authors (Hongxia Xu and Jiankun Xing) independently extracted information from eligible RCTs, including author, year of publication, characteristics of the population (including number of patients, age, chemotherapy regimen and course, score of status), intervention (including 5-HT_3_ antagonists with or without Dexamethasone were used), outcome measure, and so on. If any discrepancies between two authors (Hongxia Xu and Jiankun Xing) appeared, a third author (Shaohui Yang) will be consulted.

#### Statistical analysis

R software (version 4.2.1) and STATA 17 were employed in this network meta-analysis to compare different treatments with a frequentist approach and the conclusions of direct and indirect comparisons were obtained.

Heterogeneity indirect-comparison meta-analysis was measured by using the I^2^ statistic and the Q statistic was employed to evaluate consistency with a random-effects model. We reported the outcome with Risk Ratio (RR), 95% confidence intervals (95% CI), with statistical significance defined as *P* < 0.05.

*P*-scores were utilized for ranking of treatments, quantifying the level of confidence that one treatment is superior to another, averaged across all competing treatments. The transitivity assumption was assessed by comparing the distribution of potential effect modifiers across comparisons, such as publication years, mean age. Sensitivity analyses were performed by separately excluding studies involving children.

Egger’s test and funnel plot (including contour funnel plots) were applied to evaluate publication bias.

## Results

### Study search, selection and characteristic

**Figure 1:**
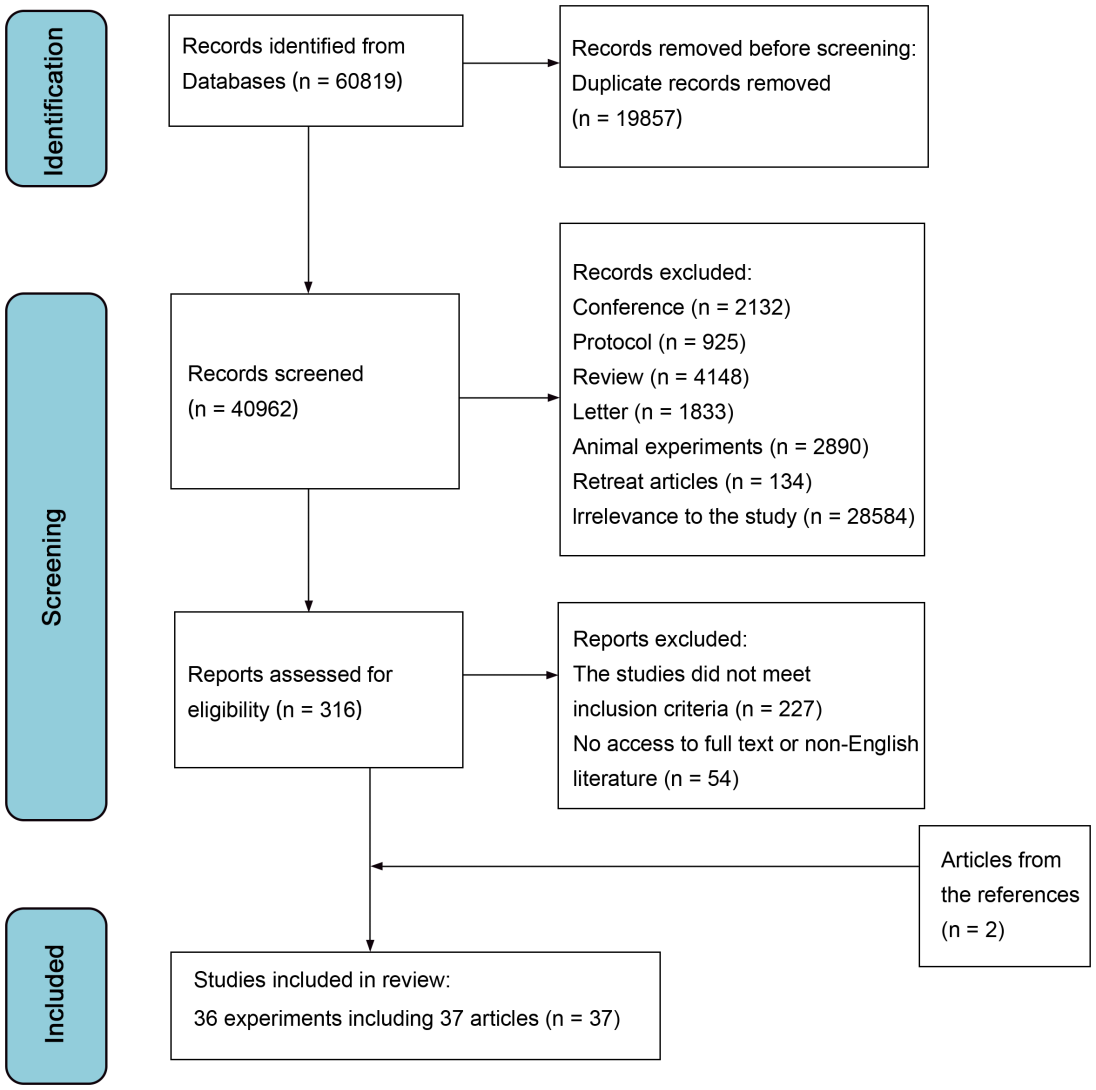
Flow diagram of literature screening and selection process.

A total of 60,819 articles were initially identified from the databases (Fig. 1). After screening, a final set of 36 randomized controlled trials (Aapro et al., 2006; Aksoylar et al., 2001; Audhuy et al., 1996; Cheirsilpa et al., 2005; Dong et al., 2011; Fauser, Pizzocaro & Schueller, 2000; Garcia-del Muro et al., 1998; Gebbia et al., 1994; Gralla et al., 1998; Heron et al., 1994; Hesketh et al., 1996; Ho et al., 2010; Italian Group of Antiemetic Research, 1995; Joss et al., 1994; Kang et al., 2002; Keyhanian et al., 2009; Kim et al., 2004; Latreille et al., 1995; Mahrous, El-Azab & Tawfik, 2021; Mantovani et al., 1996; Martoni et al., 1996; Marty et al., 1995; Mattiuzzi et al., 2010; Nakamura et al., 2012; Navari et al., 1995; Noda et al., 2002; Öge et al., 2000; Olver et al., 1996; Roila et al., 1991; Ruff et al., 1994; Saito et al., 2009; Sorbe et al., 1994; Spector et al., 1998; Tan et al., 2018; Villalon & Chan, 2004; Yu et al., 2009) (reported in 37 articles) enrolling 11,131 patients were included in this analysis. The trial conducted by Kubota et al. (2016) was a follow-up study to Saito et al. (2009). Table 1, Supplemental Information 1 summarized the characteristics of the RCTs included.

**Table 1:** Characteristics of the included RCTs.

Number	Year	Test type	Included population	Intervention (The number of each group)	Outcome indicators
Aapro et al. (2006)	2006	RCT Multicenter	673 cancer patients, age ≥18 years, including cisplatin≥80 mg/m^2^.	Palonosetron(dose1)+ Dexamethasone (150) Palonosetron(dose2)+ Dexamethasone (150) Ondansetron+ Dexamethasone (147)	②⑤
Aksoylar et al. (2001)	2001	Prospective RCT	51 children, highly emetogenic chemotherapy, in 133 chemotherapy cycles.	Tropisetron (66) Granisetron (67)	①②
Audhuy et al. (1996)	1996	RCT Multicenter	474 cancer patients over 18 years, cisplatin ≥80 mg/m^2^.	Dolasetron(dose1) (163) Dolasetron(dose2) (161) Granisetron (150)	①②
Cheirsilpa et al. (2005)	2005	RCT	73 cancer patients, aged between 20 and 80 years, cisplatin ≥70 mg/m^2^.	Ramosetron+ Dexamethasone (36) Granisetron+ Dexamethasone (37)	①②③④⑤⑥
Dong et al. (2011)	2011	RCT	89 patients, non-small cell lung carcinoma, age ≥18 years.	Palonosetron (44) Ondasetron (45)	①②③④⑤⑥
Fauser, Pizzocaro & Schueller (2000)	2000	RCT Multicenter	210 cancer patients, age ≥18 years, cisplatin (15–50 mg/m^2^).	Dolasetron (103) Dolasetrone+ Dexamethasone (107)	④⑤
Garcia-del Muro et al. (1998)	1998	RCT Multicenter	278 cancer patients, cisplatin ≥50 mg/m^2^.	Tropisetron (143) Tropisetron+ Dexamethasone (135)	①②③④⑤
Gebbia et al. (1994)	1994	RCT	182 cancer patients, cisplatin≥70 mg/m^2^.	Ondansetron (84) Granisetron (82)	②④⑤
Gralla et al. (1998)	1998	RCT Multicenter	1054 patients with malignant disease, age ≥18 year, cisplatin ≥60 mg/m^2^.	Granisetron+ Dexamethasone (417) Ondansetron+ Dexamethasone (413) Granisetron (117) Ondansetron (107)	①②③
Heron et al. (1994)	1994	RCT	357 cancer patients, cisplatin ≥50 mg/m^2^.	Granisetron (119) Granisetron+ Dexamethasone (117)	①②③④⑤⑥
Hesketh et al. (1996)	1996	RCT	609 cancer patients, age≥18 years, cisplatin ≥70 mg/m^2^.	Dolasetron(dose1 (198) Dolasetron(dose2) (205) Ondansetron (206)	②
Ho et al. (2010)	2010	RCT	124 cancer patients, age between 20 and 74 years, cisplatin ≥50 mg/m^2^.	Ramosetron+ Dexamethasone (62) Granisetron+ Dexamethasone (62)	②③
Italian Group of Antiemetic Research (1995)	1995	RCT Multicenter	966 cancer patients, cisplatin ≥50 mg/m^2^.	Ondansetron+ Dexamethasone (483) Granisetron+ Dexamethasone (483)	①②③④⑤⑥
Joss et al. (1994)	1994	Prospective RCT	215 cancer patients, cisplatin ≥50 mg/m^2^.	Ondansetron (58) Ondansetron+ Dexamethasone (53)	①②③④⑤⑥
Kang et al. (2002)	2002	RCT	203 cancer patients, 20–75 years, cisplatin ≥ 50 mg/m^2^.	Ramosetron (94) Granisetron (100)	①②
Keyhanian et al. (2009)	2009	RCT	138 cancer patients, aged 15–82 years, 30–80 mg/m^2^ cisplatin or >40 mg/m^2^ doxorubicin.	Granisetron (63) Granisetron+ Dexamethasone (62)	①②
Kim et al. (2004)	2004	RCT	114 cancer patients, age ≥18 years, cisplatin ≥60 mg/m^2^.	Dolasetron (56) Ondansetron (58)	①②④⑤
Latreille et al. (1995)	1995	RCT	292 cancer patients, age ≥18 years, cisplatin≥50 mg/m^2^.	Granisetron (98) Granisetron+ Dexamethasone (194)	②
Mahrous, El-Azab & Tawfik (2021)	2021	RCT	115 cancer patients, aged between 20 and 60 years, cisplatin ≥50 mg/m^2^ or AC/EC.	Palonosetron+ Dexamethasone (51) Granisetron+ Dexamethasone (64)	④⑤
Mantovani et al. (1996)	1996	RCT	117 cancer patients, cisplatin≥80 mg/m^2^.	Granisetron (38) Ondansetron (39) Tropisetron (40)	③
Martoni et al. (1996)	1996	RCT Crossover	124 cancer patients, cisplatinum ≥50 mg/m^2^.	Granisetron (66) Ondansetron (58)	①②③
Marty et al. (1995)	1995	Prospective RCT Multicenter	231 cancer patients, cisplatin≥50 mg/m^2^.	Tropisetron (117) Ondansetron (114)	①②④⑤
Mattiuzzi et al. (2010)	2010	RCT	150 cancer patients, >18 years, high-dose cytarabine.	Ondansetron (47) Palonosetron(dose1) (48) Palonosetron (dose2) (48)	①②③④⑤
Nakamura et al. (2012)	2012	RCT Crossover	27 breast cancer patients, ≥20 years, FEC100 (high emetic risk) treatment were enrolled.	Granisetron+ Dexamethasone (13) Azasetron+ Dexamethasone (14)	①
Navari et al. (1995)	1995	RCT Multicenter	994 cancer patients, aged ≥20 years, cisplatin ≥60 mg/m^2^.	Granisetron(dose1) (328) Granisetron(dose2) (328) Ondansetron (331)	①②③
Noda et al. (2002)	2002	RCT Multicenter	151 cancer patients, aged ≥20 years, cisplatin ≥50 mg/m^2^.	Ramosetron (75) Ondansetron (76)	①②
Öge et al. (2000)	2000	RCT	106 patients received cisplatin based chemotherapy.	Granisetron (36) Ondansetron (35) Tropisetron (35)	②⑤
Olver et al. (1996)	1996	RCT Multicenter	642 cancer patients, ≥12 years (18 years in France), cisplatin ≥70 mg/m^2^.	Ondanaetron (214) Ondanaetron + Dexamethasone (66)	②③④⑤⑥
Roila et al. (1991)	1991	RCT, Multicent Crossover	102 cancer patients, with cisplatin ≥50 mg/m^2^.	Ondansetron (41) Ondansetron+ Dexamethasone (48)	②
Ruff et al. (1994)	1994	RCT Multicenter	496 cancer patients, ≥18 years, cisplatin ≥50 mg/m^2^.	Ondansetron (dose1)(165) Ondansetron (dose2)(162) Granisetron (169)	①②③
Saito et al. (2009)	2009	RCT Multicenter	1114 cancer patients, age ≥20 years, cisplatin ≥50 mg/m^2^ or AC/EC.	Palonosetron+ Dexamethosone (555) Granisetron + Dexamethosone (559)	①②③④⑤⑥
Sorbe et al. (1994)	1994	RCT Multicenter	Women with gynaecological cancers. 100 mg/m^2^≥cisplatin≥50 mg/m^2^.	Tropisetron (35) Tropisetron+ Dexamethasone (28)	②⑤
Spector et al. (1998)	1998	RCT Multicenter	371 cancer patients, ≥ 12 years, cisplatin 50-75 mg/m^2^.	Ondansetron (184) Granisetron (187)	①②③
Tan et al. (2018)	2017	Prospective RCT	555 cancer patients, <18 years scheduled for HEC.	Palonosetron(dose1)+ Dexamethasone (181) Palonosetron(dose2)+ Dexamethasone (185) Ondansetron+ Dexamethasone (189)	①②③④⑤⑥
Villalon & Chan (2004)	2004	RCT Multicenter	283 cancer patients, 18-75 years of age, cisplatin ≥50 mg/m^2^.	Ramosetron+ Dexamethasone (149) Ramosetron (134)	①②③④⑤
Yu et al. (2009)	2009	RCT Multicenter	208 cancer patients, age ≥18 and ≤70 years, epirubicin 60 mg/m^2^ or cisplatin 75 mg/m^2^.	Granisetron (104) Palonosetron (104)	②⑤

**Notes.**

① Acute nausea; ② Acute vomiting; ③ Acute complete control; ④ Delayed nausea; ⑤ Delayed vomiting; ⑥ Delayed complete control.

AC/EC: Adriamycin + Cyclophosphamide/ Epirubicin + Cyclophosphamide.

FEC100: Fluorouracil (5-FU) + Epirubicin (100 mg/m^2^) + Cyclophosphamide.

The extracted data encompassed the author’s name, publication year, study design, sample size, patient age, interventional drugs, and the outcomes. Most data were obtained from the articles directly, but some data from the first round of chemotherapy (Mantovani et al., 1996; Marty et al., 1995; Nakamura et al., 2012; Roila et al., 1991), some data were derived from average values calculated in studies that reported the daily number of patients experiencing nausea and vomiting in the delayed phase (Cheirsilpa et al., 2005; Fauser, Pizzocaro & Schueller, 2000; Hesketh et al., 1996; Joss et al., 1994; Yu et al., 2009). For cases with multiple sets of data, only data meeting the requirements were extracted (Aapro et al., 2006; Gebbia et al., 1994; Heron et al., 1994; Ho et al., 2010; Joss et al., 1994; Latreille et al., 1995; Mattiuzzi et al., 2010; Sorbe et al., 1994). (Given that the article revision was an extended process, we have supplemented the literature search accordingly. No new eligible trials were identified, despite a supplemental search covering the period from April 1, 2025, to January 28, 2026).

### Risk of bias

**Figure 2:**
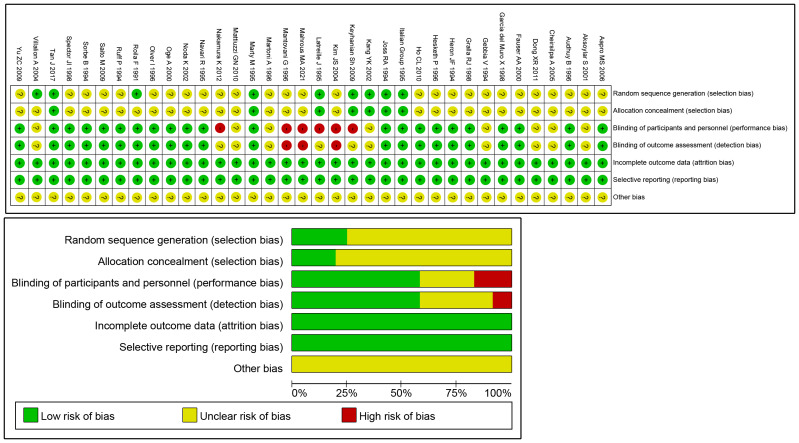
Risk of bias graph.

With Revman 5.3, a total of 36 trials were evaluated for article quality (Fig. 2).

The overall risk of bias for each study was classified according to the ratings of the individual items (Furukawa et al., 2016). Among the included studies, twenty-two (Aapro et al., 2006; Audhuy et al., 1996; Fauser, Pizzocaro & Schueller, 2000; Garcia-del Muro et al., 1998; Gralla et al., 1998; Heron et al., 1994; Hesketh et al., 1996; Ho et al., 2010; Italian Group of Antiemetic Research, 1995; Joss et al., 1994; Kang et al., 2002; Marty et al., 1995; Navari et al., 1995; Noda et al., 2002; Olver et al., 1996; Roila et al., 1991; Ruff et al., 1994; Saito et al., 2009; Sorbe et al., 1994; Spector et al., 1998; Tan et al., 2018; Yu et al., 2009) were assessed as having a Low risk of bias, eleven (Aksoylar et al., 2001; Cheirsilpa et al., 2005; Dong et al., 2011; Gebbia et al., 1994; Keyhanian et al., 2009; Latreille et al., 1995; Martoni et al., 1996; Mattiuzzi et al., 2010; Nakamura et al., 2012; Öge et al., 2000; Villalon & Chan, 2004) were categorized as Moderate risk, and three (Kim et al., 2004; Mahrous, El-Azab & Tawfik, 2021; Mantovani et al., 1996) were evaluated as High risk (Supplemental Information 2). We conducted a sensitivity analysis by removing the three studies with a high risk of bias. The results for outcomes were largely unchanged, except in cases where the network connectivity was broken, making comparison impossible. As these studies did not significantly influence the overall results (Supplemental Information 3), we ultimately included them in our final analysis. We employed a leave-one-out analysis, and the results from all altered outcome metrics demonstrate the robustness of our findings (Supplemental Information 4).

### Outcome Indicators

The outcomes of nausea, vomiting, and complete control were evaluated separately for both the acute and delayed phases. Additionally, a summary of the key outcome measures is presented in Table 2.

**Table 2:** Summary of the main results.

Comparisons	Acute nausea (Direct (n, %, I^**2**^) or Indirect; RR (95% CI)), (*p*-score)	Acute vomiting (Direct (n, %, I^**2**^) or Indirect; RR (95% CI)), (*p*-score)	Acute complete control (Direct (n, %, I^**2**^) or Indirect; RR (95% CI)), (*p*-score)	Delayed nausea (Direct (n, %, I^**2**^) or Indirect; RR (95% CI)), (*p*-score)	Delayed vomiting (Direct (n, %, I^**2**^) or Indirect; RR (95% CI)), (*p*-score)	Delayed complete control (Direct (n, %, I^**2**^) or Indirect; RR (95% CI)), (*p*-score)
Granisetron *vs* Granisetron+ Dexamethasone	Direct (2, 0.81, 0%); 1.33 (1.10–1.61)^*^, (0.2164; 0.6443)	Direct (3, 0.77, 11%); 1.57 (1.29–1.91)^*^, (0.1171; 0.6792)	Direct (1, 0.36); 0.80 (0.61–1.04), (0.2377; 0.6900)	Direct (1, 0.77); 0.97 (0.67–1.40), (0.3140; 0.4156)	Direct (1, 0.63); 0.93 (0.62–1.40), (0.4119; 0.4059)	Direct (1, 1.00); 1.12 (0.75–1.67), (0.6529; 0.4799 )
Ondansetron *vs* Ondansetron+ Dexamethasone	Direct (1, 0.18); 1.32 (1.07–1.63)^*^, (0.1927; 0.6287)	Direct (3, 0.33, 83%); 1.27 (0.88–1.85), (0.2791; 0.6304)	Direct (2, 0.73, 38%); 0.94 (0.80–1.12), (0.4027; 0.6537)	Direct (2, 0.82, 26%); 1.43 (1.03–1.98)^*^, (0.1903; 0.5603)	Direct (2, 0.71, 0%); 1.28 (0.89–1.84), (0.3100; 0.5736)	Direct (2, 1.00, 0%); 0.61 (0.47–0.81)^*^, (0.0100; 0.4811)
Ramosetron *vs* Ramosetron+ Dexamethasone	Direct (1, 0.81); 1.61 (1.16–2.24)^*^, (0.5321; 0.9473)	Direct (1, 0.71); 1.58 (1.06–2.37)^*^, (0.3661; 0.6304)	Direct (1, 1.00); 0.79 (0.65–0.96)^*^, (0.1082; 0.5415)	Direct (1, 1.00); 1.53 (1.05–2.24)^*^, (0.0952; 0.4533 )	Direct (1, 1.00); 1.63 (1.10–2.41)^*^, (0.0807; 0.4175)	–
Tropisetron *vs* Tropisetron+ Dexamethasone	Direct (1, 0.81); 1.85 (1.26–2.74)^*^, (0.0652; 0.7827)	Direct (2, 1.0, 0%); 1.99 (1.43–2.78)^*^, (0.0197; 0.7625)	Direct (1, 1.00); 0.65 (0.53–0.80)^*^, (0.1097; 0.9110)	Direct (1, 1.00); 1.49 (1.00–2.22)^*^, (0.4350; 0.8109)	Direct (2, 1.00, 0%); 1.59 (1.10–2.28)^*^, (0.3497; 0.8178)	–
Granisetron+ Dexamethasone *vs* Ondansetron	Indirect; 1.32 (1.07–1.63)^*^, (0.6443; 0.1927)	Indirect; 0.69 (0.57–0.83)^*^, (0.6792; 0.2791)	Indirect; 1.10 (0.95–1.28), (0.6900; 0.4027)	Indirect; 0.84 (0.60–1.19), (0.4156; 0.1903)	Indirect; 0.93 (0.67–1.29), (0.4059; 0.3100)	Indirect; 1.63 (1.18–2.27)^*^, (0.4799; 0.0100)
Ondansetron+ Dexamethasone *vs* Tropisetron	Indirect; 0.64 (0.44–0.93)^*^, (0.6287; 0.0652)	Indirect; 0.56 (0.41–0.77)^*^, (0.6304; 0.0197)	Indirect; 1.29 (0.99–1.67), (0.6537; 0.1097)	Indirect; 0.92 (0.55–1.53), (0.5603; 0.4350)	Indirect; 0.84 (0.56–1.28), (0.5736; 0.3497)	–
Granisetron+ Dexamethasone *vs* Tropisetron	Indirect; 0.64 (0.45–0.91)^*^, (0.6443, 0.0652)	Indirect; 0.55 (0.41–0.73)^*^, (0.6792; 0.0197)	Indirect; 1.30 (1.00–1.69)^*^, (0.6900; 0.1097)	Indirect; 1.02 (0.59–1.75), (0.4156; 0.4350)	Indirect; 0.95 (0.62–1.46), (0.4059; 0.3497)	–
Palonosetron *vs* Palonosetron+ Dexamethasone	Indirect; 1.03 (0.59–1.79), (0.6031, 0.6933)	Indirect; 1.10 (0.64–1.90), (0.7447; 0.8960)	Indirect; 0.90 (0.75–1.07), (0.5029; 0.8426)	Indirect; 1.22 (0.76–1.95), (0.6393; 0.8422)	Indirect; 1.12 (0.71–1.75), (0.7585; 0.8598)	Indirect; 0.66 (0.41–1.06), (0.3494; 0.9215)
Granisetron *vs* Palonosetron	Indirect; 1.32 (0.80–2.20), (0.2164; 0.6031)	Indirect; 1.69 (1.03;2.77)^*^, (0.1171; 0.7447)	Indirect; 0.93 (0.83–1.03), (0.2377; 0.5029)	Indirect; 1.30 (0.79–2.15), (0.3140; 0.6393)	Direct (1, 0.63); 1.12 (0.73–1.71), (0.4119; 0.7585)	Indirect; 1.27 (0.68–2.37), (0.6529; 0.3494)
Ondansetron *vs* Palonosetron	Direct (3, 1.00, 0%); 1.33 (0.80–2.20), (0.1927; 0.6031)	Direct (3, 0.17, 0%); 1.98 (0.60–6.52), (0.2791; 0.7447)	Direct (3, 1.00, 0%); 0.98 (0.91–1.05), (0.4027; 0.5029)	Direct (3, 1.00, 0%); 1.42 (1.04–1.95)^*^, (0.1903; 0.6393)	Direct (3, 0.54, 26%); 1.88 (1.17–3.02)^*^, (0.3100; 0.7585)	Direct (1, 1.00); 0.70 (0.49–0.99)^*^, (0.0100; 0.3494)
Palonosetron *vs* Ramosetron	Indirect; 0.93 (0.53–1.63), (0.6031; 0.5321)	Indirect; 0.70 (0.40–1.20), (0.7447; 0.3661)	Indirect; 1.23 (0.90–1.70), (0.5029; 0.1082)	Indirect; 0.55 (0.26–1.18), (0.6393; 0.0952)	Indirect; 0.44 (0.19–1.00)^*^, (0.7585; 0.0807)	–
Palonosetron *vs* Tropisetron	Indirect; 0.64 (0.36–1.15), (0.6031; 0.0652)	Indirect; 0.51 (0.29–0.87)^*^, (0.7447; 0.0197)	Indirect; 1.21 (0.96–1.52), (0.5029; 0.1097)	Indirect; 0.85 (0.50–1.43), (0.6393; 0.4350)	Indirect; 0.70 (0.45–1.09), (0.7585; 0.3497)	–
Ondansetron+ Dexamethasone *vs* Palonosetron+ Dexamethasone	Direct (2, 0.49, 0%); 1.14 (0.93–1.38), (0.6287; 0.6933)	Direct (2, 0.72, 29%); 1.32 (1.09–1.60)^*^, (0.6304; 0.8960)	Direct (2, 0.49, 0%); 0.93 (0.84-1.03), (0.6537; 0.8426)	Direct (2, 0.71, 19%); 1.37 (1.06–1.78)^*^, (0.5603; 0.8422)	Direct (4, 0.84, 0%); 1.33 (1.10–1.60)^*^, (0.5736; 0.8598)	Direct (2, 0.66, 61%); 0.70 (0.56–0.88)^*^, (0.4811; 0.9215)
Granisetron+ Dexamethasone *vs* Palonosetron+ Dexamethasone	Direct (1, 0.73); 0.97 (0.84–1.12), (0.6443; 0.6933)	Direct (1, 0.50); 1.05 (0.81–1.35), (0.6792; 0.8960)	Direct (1, 0.74); 0.98 (0.91–1.05), (0.6900; 0.8426)	Direct (2, 0.71, 90%); 1.41 (1.09–1.83)^*^, (0.4156; 0.8422)	Direct (2, 0.61, 93%); 1.60 (1.20–2.13)^*^, (0.4059; 0.8598 )	Direct (1, 0.68); 0.80 (0.65–0.99)^*^, (0.4799; 0.9215)
Palonosetron+ Dexamethasone *vs* Ramosetron+ Dexamethasone	Indirect; 1.39 (0.95–2.05), (0.6933; 0.9473)	Indirect; 0.89 (0.59–1.34), (0.8960; 0.7435)	Indirect; 1.09 (0.89–1.33), (0.8426; 0.5415)	Indirect; 0.69 (0.42–1.16), (0.8422; 0.4533)	Indirect; 0.64 (0.34–1.18), (0.8598; 0.4175)	Indirect; 1.23 (0.75–2.01), (0.9215; 0.6053)
Palonosetron+ Dexamethasone *vs* Tropisetron+ Dexamethasone	Indirect; 1.15 (0.67–1.98), (0.6933; 0.7827)	Indirect; 0.91 (0.57–1.46), (0.8960; 0.7625)	Indirect; 0.87 (0.62–1.23), (0.8426; 0.9110)	Indirect; 1.04 (0.53–2.03), (0.8422; 0.8109)	Indirect; 0.99 (0.56–1.74), (0.8598; 0.8178)	–
Granisetron+ Dexamethasone *vs* Palonosetron	Indirect; 0.99 (0.58–1.71), (0.6443; 0.6031)	Indirect; 1.08 (0.64–1.82), (0.6792; 0.7447)	Indirect; 1.08 (0.91–1.27), (0.6900; 0.5029)	Indirect; 1.20 (0.75–1.92), (0.4156; 0.6393)	Indirect; 1.36 (0.88–2.11), (0.4059; 0.7585)	Indirect; 1.14 (0.71–1.84), (0.4799; 0.3494)
Ondansetron+ Dexamethasone *vs* Palonosetron	Indirect; 1.00 (0.58–1.73), (0.6287; 0.6031)	Indirect; 1.11 (0.65–1.89), (0.6304; 0.7447)	Indirect; 1.07 (0.91–1.26), (0.6537; 0.5029)	Indirect; 1.09 (0.70–1.67), (0.5603; 0.6393)	Indirect; 1.21 (0.79–1.87), (0.5736; 0.7585)	Indirect; 1.14 (0.73–1.77), (0.4811; 0.3494)
Palonosetron *vs* Ramosetron + Dexamethasone	Indirect; 1.43 (0.77–2.65), (0.6031; 0.9473)	Indirect; 0.98 (0.53–1.81), (0.7447; 0.7435)	Indirect; 0.98 (0.76–1.26), (0.5029; 0.5415)	Indirect; 0.84 (0.44–1.63), (0.6393; 0.4533)	Indirect; 0.71 (0.35–1.47), (0.7585; 0.4175)	Indirect; 0.81 (0.42–1.58), (0.3494; 0.6053)
Palonosetron *vs* Tropisetron +Dexamethasone	Indirect; 1.19 (0.59–2.40); (0.6031; 0.7827)	Indirect; 1.01 (0.53–1.91), (0.7447; 0.7625)	Indirect; 0.78 (0.57–1.07), (0.5029; 0.9110)	Indirect; 1.26 (0.65–2.43), (0.6393; 0.8109)	Indirect; 1.10 (0.62–1.96), (0.7585; 0.8178)	–

**Notes.**

Direct (n, %, I^**2**^)= Direct comparision(number of studies, direct comparison of contribution proportions, heterogeneity between studies). comparision.

* A statistically significant difference was observed among the comparative results.

–, Comparison results are not available.

*p*-score, The *p*-score values.

#### The rate of acute nausea

**Figure 3:**
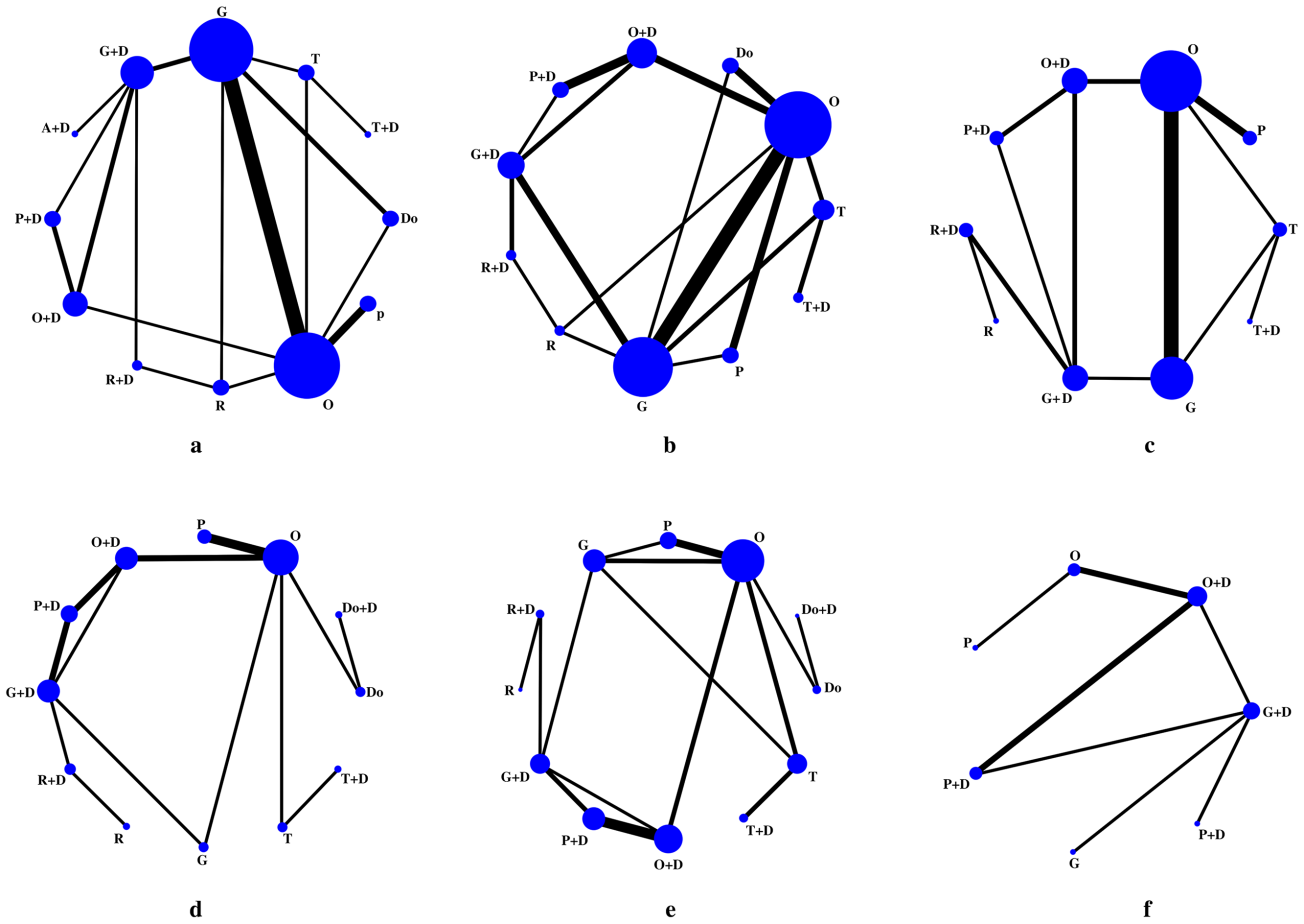
Network graph for the outcomes. (A) Network graph for the outcome of the acute nausea; (B) Network graph for the outcome of the acute vomiting; (C) Network graph for the outcome of the acute complete control; (D) Network graph for the outcome of the delayed nausea; (E) Network graph for the outcome of the delayed vomiting; (F) Network graph for the outcome of the delayed complete control. A, Azasetron; O, Ondansetron; G, Granisetron; Do, Dolasetron; T, Tropisetron; R, Ramosetron; P, Palonosetron; D, Dexamethasone.

A total of 23 RCTs and 9,096 patients were included in “the rate of acute nausea” analysis (Fig. 3). The heterogeneity within the network was low (I^2^ = 0% [0.0%; 48.9%]) (Supplemental Information 5), with no statistically significant inconsistency (*p* = 0.0896 > 0.05) (Supplemental Information 6, Supplemental Information 7).

Based on the league table (Table 3) and the forest plot (Supplemental Information 8), the key findings for acute nausea are summarized below. Significant differences were shown in acute nausea between T and T+D (RR 1.85, 95% CI [1.26–2.74]), between G and G+D (RR 1.33, 95% CI [1.10–1.61]), between O and O+D (RR 1.33, 95% CI [1.07–1.66]), and between R and R+D (RR 1.54, 95% CI [1.14–2.07]) in direct comparisons. In indirect comparisons, a 5-HT_3_ antagonists used alone was still significantly less effective than a different 5-HT_3_ antagonists + Dexamethasone. For example, Granisetron was less effective than Ondansetron + Dexamethasone (RR 1.32, 95% CI [1.07–1.63]). However, no statistically significant difference was observed between Palonosetron and Palonosetron + Dexamethasone (RR 1.03, 95% CI [0.59–1.79]). No evidence indicated a statistically significant difference between Palonosetron monotherapy and 1^st^ 5-HT_3_ antagonists in the management of acute nausea. No significant difference was observed between either Palonosetron or Palonosetron + Dexamethasone and 1^st^ 5-HT_3_ antagonists combined with Dexamethasone.

**Table 3:** Network calculation of acute nausea.

A+D	–	–	1.16 (0.67; 2.00)	–	–	–	–	–	–	–	–
0.89 (0.49; 1.61)	Do	0.98 (0.85; 1.12)	–	0.99 (0.68; 1.42)	–	–	–	–	–	–	–
0.87 (0.49; 1.55)	0.98 (0.86; 1.11)	G	1.32 (1.07; 1.63)^*^	1.00 (0.93; 1.07)	–	–	–	1.32 (0.97; 1.81)	–	0.41 (0.23; 0.73)^*^	–
1.16 (0.67; 2.00)	1.30 (1.04; 1.64) ^*^	1.33 (1.10; 1.61)^*^	G+D	–	1.04 (0.91; 1.19)	–	0.97 (0.84; 1.12)	.	1.22 (0.66; 2.23)	–	–
0.87 (0.49; 1.55)	0.97 (0.85; 1.12)	0.99 (0.93; 1.06)	0.75 (0.61; 0.91)	O	1.26 (0.74; 2.12)	1.33 (0.80; 2.20)	–	1.16 (0.72; 1.85)	–	1.10 (0.78; 1.56)	–
1.15 (0.66; 2.01)	1.29 (1.01; 1.66) ^*^	1.32 (1.07; 1.63)^*^	0.99 (0.89; 1.11)	1.33 (1.07; 1.66)^*^	O+D	–	1.14 (0.93; 1.38)	–	–	–	–
1.15 (0.54; 2.49)	1.30 (0.77; 2.19)	1.32 (0.80; 2.20)	0.99 (0.58; 1.71)	1.33 (0.80; 2.20)	1.00 (0.58; 1.73)	P	–	–	–	–	–
1.19 (0.68; 2.07)	1.33 (1.03; 1.72) ^*^	1.36 (1.09; 1.70)^*^	1.02 (0.91; 1.15)	1.37 (1.09; 1.72)^*^	1.03 (0.90; 1.18)	1.03 (0.59; 1.79)	P+D	–	–	–	–
1.07 (0.58; 1.99)	1.20 (0.91; 1.59)	1.23 (0.96; 1.57)	0.92 (0.69; 1.24)	1.24 (0.96; 1.59)	0.93 (0.68; 1.27)	0.93 (0.53; 1.63)	0.90 (0.66; 1.24)	R	1.61 (1.16; 2.24)^*^	–	–
1.65 (0.86; 3.18)	1.85 (1.28; 2.69) ^*^	1.89 (1.33; 2.69)^*^	1.42 (0.99; 2.06)	1.90 (1.34; 2.71)^*^	1.43 (0.98; 2.10)	1.43 (0.77; 2.65)	1.39 (0.95; 2.05)	1.54 (1.14; 2.07)^*^	R+D	–	–
0.74 (0.39; 1.41)	0.83 (0.60; 1.15)	0.85 (0.63; 1.14)	0.64 (0.45; 0.91)^*^	0.85 (0.63; 1.15)	0.64 (0.44; 0.93)^*^	0.64 (0.36; 1.15)	0.62 (0.43; 0.90)^*^	0.69 (0.47; 1.01)	0.45 (0.28; 0.71)^*^	T	1.85 (1.26; 2.74)^*^
1.37 (0.64; 2.92)	1.54 (0.93; 2.56)	1.57 (0.96; 2.57)	1.18 (0.70; 2.00)	1.58 (0.97; 2.58)	1.19 (0.70; 2.03)	1.19 (0.59; 2.40)	1.15 (0.67; 1.98)	1.28 (0.74; 2.21)	0.83 (0.45; 1.52)	1.85 (1.26; 2.74)^*^	T+D

**Notes.**

Indirect comparisons results: left lower half of the table; Direct pairwise comparison results: upper right half of the table.

A, Azasetron; O, Ondansetron; G, Granisetron; Do, Dolasetron; T, Tropisetron; R, Ramosetron; P, Palonosetron; D, Dexamethason.

* A statistically significant difference was observed among the comparative results.

–, Comparison results are not available.

It was most likely that Ramosetron + Dexamethasone would be the most effective treatment for acute nausea (*p*-score 0.9473), followed by T+D (*p*-score 0.7827), P+D (*p*-score 0.6933), G+D (*p*-score 0.6443), O+D (*p*-score 0.6287), P (*p*-score 0.6031), and 1^st^ 5-HT_3_ antagonists (Table 4).

**Table 4:** *p*-score value of outcome indicators.

Drugs	Acute nausea	Acute vomiting	Acute CC	Delayed nausea	Delayed vomiting	Delayed CC
P+D	0.6933	0.8960	0.8426	0.8422	0.8598	0.9215
O+D	0.6287	0.6304	0.6537	0.5603	0.5736	0.4811
G+D	0.6443	0.6792	0.6900	0.4156	0.4059	0.4799
T+D	0.7827	0.7625	0.9110	0.8109	0.8178	/
R+D	0.9473	0.7435	0.5415	0.4533	0.4175	0.6053
P	0.6031	0.7447	0.5029	0.6393	0.7585	0.3494
O	0.1927	0.2791	0.4027	0.1903	0.3100	0.0100
G	0.2164	0.1171	0.2377	0.3140	0.4119	0.6529
T	0.0652	0.0197	0.1097	0.4350	0.3497	/
R	0.5321	0.3661	0.1082	0.0952	0.0807	/
Do	0.2580	0.2617	/	0.3289	0.1485	/
Do +D	/	/	/	0.9149	0.8660	/
A+D	0.4317	/	/	/	/	/

**Notes.**

P, Palonosetron; D, Dexamethasone; O, Ondansetron; G, Granisetron; T, Tropisetron; R, Ramosetron; Do, Dolasetron; A, Azasetron.

#### The rate of acute vomiting

Thirty-two RCTs with a total of 11,898 patients reported on acute vomiting (Fig. 3). Among the studies in this network, heterogeneity was low (I^2^ = 18% [0.0%; 47.2%]) (Supplemental Information 5), and there was no statistically significant inconsistency (*p* = 0.5130 > 0.05) (Supplemental Information 6, Supplemental Information 7).

The results for acute vomiting are presented in the league table (Table 5) and forest plot (Supplemental Information 9). Significant differences were noted in acute vomiting between G and G+D (RR 1.57, 95% CI [1.32–1.86]), between O and O+D (RR 1.41, 95% CI [1.14–1.74]), between R and R+D (RR 1.40, 95%CI [1.00–1.97]), and between T and T+D (RR 1.99, 95% CI [1.43–2.78]). Additional significant differences were noted in indirect regimen comparisons, including G+D *vs* R (RR 0.75, 95% CI [0.57–0.99]), G+D *vs* O (RR 0.69, 95% CI [0.57–0.83]), O+D *vs* T (RR 0.56, 95% CI [0.41–0.77]). No significant superiority of Palonosetron + Dexamethasone over Palonosetron alone was observed (RR 1.10, 95% CI [0.64–1.90]). In indirect comparisons, Palonosetron monotherapy exhibited significant advantages compared to Granisetron (RR 1.69, 95% CI [1.03–2.77]) and Tropisetron (RR 0.51, 95% CI [0.29–0.87]).

**Table 5:** Network calculation of acute vomiting.

Do	0.96 (0.80; 1.15)	–	0.99 (0.85; 1.15)	–	–	–	–	–	–	–
0.93 (0.82; 1.05)	G	1.57 (1.29;1.91)^*^	1.09 (0.99; 1.19)	–	1.61 (0.94; 2.76)	–	1.32 (0.94; 1.86)	–	0.91 (0.65; 1.27)	–
1.46(1.18 ; 1.80)^*^	1.57 (1.32;1.86)^*^	G + D	–	1.06 (0.88; 1.28)	–	1.05 (0.81; 1.35)	–	0.84 (0.48; 1.46)	–	–
1.01 (0.89; 1.14)	1.08 (1.00;1.18)^*^	0.69 (0.57;0.83)^*^	O	1.27 (0.88; 1.85)	1.98 (0.60; 6.52)	–	1.06 (0.74; 1.52)	–	0.78 (0.60; 1.03)	–
1.41 (1.11; 1.79)^*^	1.52 (1.24;1.87)^*^	0.97 (0.84; 1.13)	1.41 (1.14; 1.74)^*^	O + D	–	1.32 (1.09; 1.60)^*^	–	–	–	–
1.57 (0.95; 2.61)	1.69 (1.03;2.77)^*^	1.08 (0.64; 1.82)	1.56 (0.95; 2.57)	1.11 (0.65; 1.89)	P	–	–	–	–	–
1.73 (1.33; 2.26)^*^	1.87 (1.47;2.37)^*^	1.19 (0.99; 1.43)	1.72 (1.35; 2.20)^*^	1.23 (1.04; 1.44)^*^	1.10 (0.64; 1.90)	P+D	–	–	–	–
1.10 (0.84; 1.42)	1.18 (0.93;1.50)	0.75 (0.57;0.99)^*^	1.09 (0.86; 1.38)	0.78 (0.57; 1.05)	0.70 (0.40; 1.20)	0.63 (0.46; 0.87)^*^	R	1.58(1.06; 2.37)^*^	–	–
1.54 (1.05; 2.26)^*^	1.66 (1.15;2.39)^*^	1.06 (0.73; 1.53)	1.53 (1.05; 2.22)^*^	1.09 (0.73; 1.62)	0.98 (0.53; 1.81)	0.89 (0.59; 1.34)	1.40 (1.00; 1.97)^*^	R + D	–	–
0.79 (0.61; 1.03)	0.85 (0.67;1.08)	0.55 (0.41;0.73)^*^	0.79 (0.62; 1.00)^*^	0.56 (0.41; 0.77)^*^	0.51 (0.29; 0.87)^*^	0.46 (0.33; 0.64)	0.72 (0.52; 1.01)	0.52 (0.33; 0.80)^*^	T	1.99(1.43; 2.78)^*^
1.58 (1.04; 2.41)^*^	1.70 (1.13;2.57)^*^	1.09 (0.70; 1.69)	1.57 (1.05; 2.36)^*^	1.12 (0.71; 1.77)	1.01 (0.53; 1.91)	0.91 (0.57; 1.46)	1.44 (0.90; 2.31)	1.03 (0.59; 1.78)	1.99 (1.43;2.78)^*^	T+ D

**Notes.**

Indirect comparisons results: left lower half of the table; Direct pairwise comparison results: upper right half of the table.

O, Ondansetron; G, Granisetron; Do, Dolasetron; T, Tropisetron; R, Ramosetron; P, Palonosetron; D, Dexamethason.

* A statistically significant difference was observed among the comparative results.

–, Comparison results are not available.

While a significant difference was observed between Ondansetron + Dexamethasone and Palonosetron + Dexamethasone (P+D) (RR 1.23, 95% CI [1.04–1.44]), no statistically significant differences were found between P+D and other 1^st^5-HT_3_ antagonists combined with Dexamethasone. No evidence was found to suggest that the effects of Palonosetron are significantly different from those of first-generation 5-HT_3_ receptor antagonists combined with Dexamethasone.

Palonosetron + Dexamethasone achieved the highest efficacy for acute vomiting control (*p*-score 0.8960), followed by T+D (*p*-score 0.7625), P (*p*-score 0.7447), R+D (*p*-score 0.7435), G+D (*p*-score 0.6792), O+D (*p*-score 0.6304), and 1^st^ 5-HT3 antagonists . The *p*-scores of P+D, T+D, P, and R+D were numerically similar (Table 4).

#### The rate of acute complete control

For the outcome of acute complete control, 18 RCTs involving 8,128 patients were included in the network (Fig. 3). The heterogeneity was low (I^2^ = 0% [0.0%; 52.3%]) (Supplemental Information 5), and there was no statistically significant inconsistency (*p* = 0.6249 > 0.05) (Supplemental Information 6, Supplemental Information 7).

As presented in the league table (Table 6) and forest plot (Supplemental Information 10), the results are as follows. There was a statistically statistical significance between R and R+D (RR 0.79, 95% CI [0.65–0.96]), between T and T+D (RR 0.65, 95% CI [0.53–0.80]), between G+D and R (RR 1.33, 95% CI [1.01–1.74]), and so on. In contrast, no significant difference was found between Palonosetron and Palonosetron + Dexamethasone (RR 0.90, 95% CI [0.75–1.07]). Nevertheless, no statistically significant differences were observed between either Palonosetron monotherapy or the Palonosetron + Dexamethasone regimen and 1^st^ 5-HT_3_ antagonists administered concomitantly with Dexamethasone.

**Table 6:** Network calculation of acute complete control.

G	0.80 (0.61; 1.04)	0.96 (0.89; 1.03)	–	–	–	–	–	1.16 (0.92; 1.47)	–
0.86 (0.74; 1.01)	G+D	–	0.99 (0.92; 1.06)	–	0.98 (0.91; 1.05)	–	1.05 (0.87; 1.27)	–	–
0.95 (0.88; 1.02)	1.10 (0.95; 1.28)	O	0.94 (0.80; 1.12)	0.98 (0.91; 1.05)	–	–	–	1.13 (0.89; 1.44)	–
0.87 (0.74; 1.01)	1.01 (0.95; 1.07)	0.91 (0.79; 1.06)	O+D	–	0.93 (0.84; 1.03)	–	–	–	–
0.93 (0.83; 1.03)	1.08 (0.91; 1.27)	0.98 (0.91; 1.05)	1.07 (0.91; 1.26)	P	–	–	–	–	–
0.83 (0.71; 0.98)^*^	0.96 (0.91; 1.03)	0.87 (0.75; 1.02)	0.96 (0.89; 1.03)	0.90 (0.75; 1.07)	P+D	–	–	–	–
1.14 (0.83; 1.57)	1.33 (1.01; 1.74)^*^	1.20 (0.88; 1.64)	1.32 (1.00; 1.74)^*^	1.23 (0.90; 1.70)	1.37 (1.04; 1.82)^*^	R	0.79 (0.65; 0.96)^*^	–	–
0.91 (0.71; 1.16)	1.05 (0.87; 1.27)	0.95 (0.75; 1.22)	1.04 (0.85; 1.28)	0.98 (0.76; 1.26)	1.09 (0.89; 1.33)	0.79 (0.65; 0.96)^*^	R+D	–	–
1.12 (0.90; 1.39)	1.30 (1.00; 1.69)^*^	1.18 (0.95; 1.47)	1.29 (0.99; 1.67)	1.21 (0.96; 1.52)	1.35 (1.03; 1.76)^*^	0.98 (0.67; 1.43)	1.24 (0.89; 1.71)	T	0.65 (0.53; 0.80)^*^
0.73 (0.54; 0.98)^*^	0.84 (0.60; 1.18)	0.77 (0.57; 1.03)	0.84 (0.60; 1.17)	0.78 (0.57; 1.07)	0.87 (0.62; 1.23)	0.64 (0.41; 0.98)^*^	0.80 (0.55; 1.18)	0.65 (0.53; 0.80)^*^	T+D

**Notes.**

Indirect comparisons results: left lower half of the table; Direct pairwise comparison results: upper right half of the table.

O, Ondansetron; G, Granisetron; T, Tropisetron; R, Ramosetron; P, Palonosetron; D, Dexamethason.

* A statistically significant difference was observed among the comparative results.

–, Comparison results are not available.

Tropisetron + Dexamethasone seemed to be the most effective treatment for acute complete control (*p*-score 0.9110), followed by P+D (*p*-score 0.8426), G+D (*p*-score 0.6900), O+D (*p*-score 0.6537), R+D (*p*-score 0.5415), P (*p*-score 0.5029), and 1^st^ 5-HT3 antagonists. The *p*-scores for T+D and P+D were numerically similar. Likewise, the *p*-scores of R+D and P demonstrated comparable values (Table 4).

#### The rate of delayed nausea

A total of 16 RCTs with 5075 patients were included in this analysis (Fig. 3). The analysis indicated moderate heterogeneity among studies (*I*^2^ = 65.5% [26.5%; 83.8%]) (Supplemental Information 5). No statistically significant inconsistency was detected (*p* = 0.4987 >0.05) (Supplemental Information 6, Supplemental Information 7).

In the league table (Table 7) and forest plot (Supplemental Information 11), the results are as follows. Regarding delayed nausea control, statistical significance was identified in multiple comparative analyses: R *vs* R+D (RR 1.53, 95% CI [1.05–2.24]), T *vs* T+D (RR 1.49, 95% CI [1.00–2.22]), Do *vs* Do+D (RR 1.99, 95% CI [1.28–3.08]), Do+D *vs* O (RR 0.46, 95% CI [0.26–0.81]), and so on. However, the adjunct administration of Dexamethasone failed to demonstrate a statistically significant improvement in the therapeutic efficacy of Palonosetron (RR 1.22, 95% CI [0.76–1.95]).

**Table 7:** Network calculation of delayed nausea.

Do	1.99 (1.28; 3.08)^*^	–	–	0.91 (0.63; 1.31)	–	–	–	–	–	–	–
1.99 (1.28; 3.08)^*^	Do+D	–	–	–	–	–	–	–	–	–	–
1.00 (0.59; 1.69)	0.50 (0.25; 1.00)^*^	G	0.97 (0.67; 1.40)	1.17 (0.68; 2.02)	–	–	–	–	–	–	–
1.08 (0.65; 1.79)	0.54 (0.28; 1.06)	1.09 (0.79; 1.50)	G+D	–	1.07 (0.76; 1.51)	–	1.41 (1.09; 1.83)^*^	–	1.02 (0.64; 1.61)	–	–
0.91 (0.63; 1.31)	0.46 (0.26; 0.81)^*^	0.92 (0.62; 1.35)	0.84 (0.60; 1.19)	O	1.43 (1.03; 1.98)^*^	1.42 (1.04; 1.95)^*^	–	–	–	1.21 (0.80; 1.83)	–
1.20 (0.75; 1.91)	0.60 (0.32; 1.14)	1.20 (0.84; 1.73)	1.11 (0.87; 1.40)	1.31 (0.98; 1.76)	O+D	–	1.37 (1.06; 1.78)^*^	–	–	–	–
1.30 (0.80; 2.10)	0.65 (0.34; 1.25)	1.30 (0.79; 2.15)	1.20 (0.75; 1.92)	1.42 (1.04; 1.95)^*^	1.09 (0.70; 1.67)	P	–	–	–	–	–
1.58 (0.95; 2.62)	0.80 (0.41; 1.55)	1.59 (1.09; 2.30)^*^	1.46 (1.18; 1.82)^*^	1.73 (1.22; 2.46)^*^	1.32 (1.06; 1.64)^*^	1.22 (0.76; 1.95)	P+D	–	–	–	–
0.72 (0.33; 1.57)	0.36 (0.15; 0.88)^*^	0.72 (0.36; 1.42)	0.66 (0.36; 1.21)	0.78 (0.39; 1.57)	0.60 (0.31; 1.14)	0.55 (0.26; 1.18)	0.45 (0.24; 0.86)^*^	R	1.53 (1.05; 2.24)^*^	–	–
1.10 (0.55; 2.18)	0.55 (0.25; 1.24)	1.10 (0.63; 1.94)	1.02 (0.64; 1.61)	1.20 (0.67; 2.15)	0.92 (0.55; 1.54)	0.84 (0.44; 1.63)	0.69 (0.42; 1.16)	1.53 (1.05; 2.24)^*^	R+D	–	–
1.10 (0.64; 1.91)	0.55 (0.27; 1.12)	1.11 (0.63; 1.95)	1.02 (0.59; 1.75)	1.21 (0.80; 1.83)	0.92 (0.55; 1.53)	0.85 (0.50; 1.43)	0.70 (0.41; 1.20)	1.54 (0.69; 3.45)	1.00 (0.49; 2.05)	T	1.49 (1.00; 2.22)^*^
1.64 (0.83; 3.23)	0.82 (0.37; 1.85)	1.64 (0.82; 3.29)	1.51 (0.77; 2.96)	1.79 (1.01; 3.19)^*^	1.37 (0.72; 2.61)	1.26 (0.65; 2.43)	1.04 (0.53; 2.03)	2.29 (0.93; 5.63)	1.49 (0.66; 3.37)	1.49 (1.00; 2.22)^*^	T+D

**Notes.**

Indirect comparisons results: left lower half of the table; Direct pairwise comparison results: upper right half of the table.

O, Ondansetron; G, Granisetron; Do, Dolasetron; T, Tropisetron; R, Ramosetron; P, Palonosetron; D, Dexamethason.

* A statistically significant difference was observed among the comparative results.

–, Comparison results are not available.

In direct comparative analyses, Palonosetron exhibited statistically superior efficacy relative to Ondansetron (RR 1.42, 95% CI [1.04–1.95]), not to other first-generation 5-HT_3_ antagonists. Palonosetron + Dexamethasone was significantly more effective than Granisetron + Dexamethasone (RR 1.46, 95% CI [1.18–1.82]) and Ondansetron + Dexamethasone (RR 1.32, 95% CI [1.06–1.64]). However, indirect comparisons revealed no significant efficacy differences between Palonosetron + Dexamethasone and either Dolasetron + Dexamethasone, Tropisetron + Dexamethasone, or Ramosetron + Dexamethasone. Similarly, no evidence has been established to indicate a difference in therapeutic efficacy between Palonosetron and 1^st^ 5-HT_3_ antagonists + Dexamethasone.

In delayed nausea, Dolasetron + Dexamethasone was the most likely effective treatment (*p*-score 0.9149), followed by P+D (*p*-score 0.8422), T+D (*p*-score 0.8109), P (*p*-score 0.6393), O+D (*p*-score 0.5603), R+D (*p*-score 0.4533), T (*p*-score 0.4350), G+D (*p*-score 0.4156), and so on. The *p*-scores of Dolasetron + Dexamethasone, Tropisetron + Dexamethasone, and Palonosetron + Dexamethasone were relatively close. Similarly, the *p*-score of Palonosetron was comparable to those of 1^st^ 5-HT_3_ antagonists + Dexamethasone (Table 4).

#### The rate of delayed vomiting

This analysis included 20 RCTs (Fig. 3), involving 6,347 patients. Heterogeneity within the network was moderate (I^2^ = 49% [5.4%; 72.5%]) (Supplemental Information 5), but there was no statistically significant inconsistency in this indicator (*p* = 0.6766 >0.05) (Supplemental Informations 6,  7.

As illustrated in the league table (Table 8) and forest plot (Supplemental Information 12), the results are as follows. In delayed vomiting, statistical significance was found between 1^st^5-HT_3_ antagonists and 1^st^ 5-HT_3_ antagonists + Dexamethasone, such as R *vs* R+D (RR 1.63, 95% CI [1.10–2.41]), T *vs* T+D (RR 1.59, 95% CI [1.10–2.28]), Do *vs* Do+D (RR 2.33, 95% CI [1.54–3.53]), O *vs* T+D (RR 1.62, 95% CI [1.01–2.60]), Do+D *vs* R (RR 0.35, 95% CI [0.13–0.94]). In contrast, no evidence was found to indicate that the therapeutic efficacy of Palonosetron was significantly inferior to that of its combination with Dexamethasone (RR 1.12, 95% CI [0.71–1.75]). Palonosetron was significantly more effective than Ondansetron (RR 1.47, 95% CI [1.03–2.08]), Ramosetron (RR 0.44, 95% CI [0.19–1.00]), Dolasetron (RR 1.86 , 95% CI [1.04–3.34]). In direct comparisons, Palonosetron + Dexamethasone (P+D) demonstrated significantly greater efficacy than both Ondansetron + Dexamethasone (RR 1.35, 95% CI [1.14–1.60]) and Granisetron + Dexamethasone (RR 1.52, 95% CI [1.22–1.91]). However, indirect comparison analyses revealed no statistically significant differences in efficacy between P+D and Do+D, T+D, or R+D. Similarly, no significant efficacy difference was observed between Palonosetron monotherapy and 1^st^ 5-HT_3_ antagonists + Dexamethasone in indirect comparisons.

**Table 8:** Network calculation of delayed vomiting.

Do	2.33 (1.54; 3.53)^*^	–	–	1.27 (0.80; 2.02)	–	–	–	–	–	–	–
2.33 (1.54; 3.53)^*^	Do+D	–	–	–	–	–	–	–	–	–	–
1.36 (0.80; 2.34)	0.59 (0.30; 1.15)	G	0.93 (0.62; 1.40)	1.09 (0.77; 1.55)	–	1.12 (0.73; 1.71)	–	–	–	1.14 (0.67; 1.96)	–
1.37 (0.77; 2.42)	0.59 (0.29; 1.19)	1.00 (0.72; 1.38)	G+D	–	0.99 (0.69; 1.41)	–	1.60 (1.20; 2.13)^*^	–	0.97 (0.55; 1.73)	–	–
1.27 (0.80; 2.02)	0.54 (0.29; 1.01)	0.93 (0.71; 1.21)	0.93 (0.67; 1.29)	O	1.28 (0.89; 1.84)	1.88 (1.17; 3.02)^*^	–	–	–	0.98 (0.71; 1.33)	–
1.54 (0.88; 2.68)	0.66 (0.33; 1.32)	1.13 (0.81; 1.57)	1.13 (0.90; 1.42)	1.21 (0.89; 1.64)	O+D	–	1.33 (1.10; 1.60)^*^	–	–	–	–
1.86 (1.04; 3.34)^*^	0.80 (0.39; 1.63)	1.36 (0.97; 1.92)	1.36 (0.88; 2.11)	1.47 (1.03; 2.08)^*^	1.21 (0.79; 1.87)	P	–	–	–	–	–
2.08 (1.17; 3.69)^*^	0.89 (0.44; 1.81)	1.52 (1.07; 2.16)^*^	1.52 (1.22; 1.91)^*^	1.64 (1.17; 2.29)^*^	1.35 (1.14; 1.60)^*^	1.12 (0.71; 1.75)	P+D	–	–	–	–
0.82 (0.33; 2.01)	0.35 (0.13; 0.94)^*^	0.60 (0.28; 1.29)	0.60 (0.30; 1.20)	0.64 (0.30; 1.39)	0.53 (0.26; 1.11)	0.44 (0.19; 1.00)^*^	0.39 (0.19; 0.82)^*^	R	1.63 (1.10; 2.41)^*^	–	–
1.33 (0.59; 2.99)	0.57 (0.23; 1.41)	0.97 (0.50; 1.88)	0.97 (0.55; 1.73)	1.05 (0.54; 2.03)	0.86 (0.47; 1.60)	0.71 (0.35; 1.47)	0.64 (0.34; 1.18)	1.63 (1.10; 2.41)^*^	R+D	–	–
1.30 (0.74; 2.26)	0.56 (0.28; 1.11)	0.95 (0.66; 1.37)	0.95 (0.62; 1.46)	1.02 (0.75; 1.38)	0.84 (0.56; 1.28)	0.70 (0.45; 1.09)	0.62 (0.40; 0.96)^*^	1.59 (0.70; 3.60)	0.98 (0.48; 2.00)	T	1.59 (1.10; 2.28)^*^
2.06 (1.06; 4.00)^*^	0.88 (0.40; 1.93)	1.51 (0.90; 2.52)	1.51 (0.86; 2.64)	1.62 (1.01; 2.60)^*^	1.34 (0.77; 2.32)	1.10 (0.62; 1.96)	0.99 (0.56; 1.74)	2.52 (1.03; 6.16)^*^	1.55 (0.69; 3.46)	1.59 (1.10; 2.28)^*^	T+D

**Notes.**

Indirect comparisons results: left lower half of the table; Direct pairwise comparison results: upper right half of the table.

O, Ondansetron; G, Granisetron; Do, Dolasetron; T, Tropisetron; R, Ramosetron; P, Palonosetron; D, Dexamethason.

* A statistically significant difference was observed among the comparative results.

–, Comparison results are not available.

Dolasetron + Dexamethasone appeared to be the most effective treatment for delayed vomiting (*p*-score 0.8660), followed by P+D (*p*-score 0.8598), T+D (*p*-score 0.8178), P (*p*-score 0.7585), O+D (*p*-score 0.5736), R+D (*p*-score 0.4175), G (*p*-score 0.4119), G+D (*p*-score 0.4059), and so forth . The *p*-scores for Palonosetron + Dexamethasone, Dolasetron + Dexamethasone (Do+D), and Tropisetron + Dexamethasone (T+D) were relatively comparable (Table 4).

#### The rate of delayed complete control

For delayed complete control), 8 RCTs involving 3589 patients were analyzed (Fig. 3). The heterogeneity within the network was low (*I*^2^ = 37.9% [0.0%; 78.7%]) (Supplemental Information 5), and no statistically significant inconsistency appeared in this indicator (*p* = 0.2986 > 0.05) (Supplemental Informations 6,  7).

The results, derived from the league table (Table 9) and forest plot (Supplemental Information 13), are summarized below. In the delayed complete response analysis, statistically significant differences were observed between O and O+D (RR 0.61, 95% CI [0.47–0.81]), between G+D and O (RR 1.63, 95% CI [1.18–2.27]) in direct comparative assessments. Notably, indirect comparative analyses revealed no statistically significant difference between Palonosetron and Palonosetron + Dexamethasone (RR 0.66, 95% CI [0.41–1.06]). Palonosetron was significantly more effective than Ondansetron (RR 0.70, 95% CI [0.49–0.99]). P+D demonstrated superior efficacy *versus* both G+D (RR 0.75, 95% CI [0.63–0.90]) and O+D (RR 0.75, 95% CI [0.63–0.90]) in direct comparisons. However, no significant difference was observed between P+D and R+D in indirect comparisons (RR 1.23, 95% CI [0.75–2.01]).

**Table 9:** Network calculation of delayed complete control.

G	1.12 (0.75; 1.67)	–	–	–	–	–
1.12 (0.75; 1.67)	G+D	–	0.94 (0.75; 1.17)	–	0.80 (0.65; 0.99)^*^	0.92 (0.58; 1.47)
1.82 (1.09; 3.06)^*^	1.63 (1.18; 2.27)^*^	O	0.61 (0.47; 0.81)^*^	0.70 (0.49; 0.99)^*^	–	–
1.12 (0.72; 1.73)	1.00 (0.84; 1.20)	0.61 (0.47; 0.81)^*^	O+D	–	0.70 (0.56; 0.88)^*^	–
1.27 (0.68; 2.37)	1.14 (0.71; 1.84)	0.70 (0.49; 0.99)^*^	1.14 (0.73; 1.77)	P	–	–
0.84 (0.54; 1.30)	0.75 (0.63; 0.90)^*^	0.46 (0.33; 0.64)^*^	0.75 (0.63; 0.90)^*^	0.66 (0.41; 1.06)	P+D	–
1.03 (0.56; 1.90)	0.92 (0.58; 1.47)	0.57 (0.32; 1.00)^*^	0.92 (0.56; 1.51)	0.81 (0.42; 1.58)	1.23 (0.75; 2.01)	R+D

**Notes.**

Indirect comparisons results: left lower half of the table; Direct pairwise comparison results: upper right half of the table.

O, Ondansetron; G, Granisetron; R, Ramosetron; P, Palonosetron; D, Dexamethason.

* A statistically significant difference was observed among the comparative results.

–, Comparison results are not available.

Palonosetron + Dexamethasone showed the most effectiveness in delayed complete control (*p*-score 0.9215), followed by G (*p*-score 0.6529), R+D (*p*-score 0.6053), O+D (*p*-score 0.4811), G+D (*p*-score 0.4799), P (*p*-score 0.3494), O (*p*-score 0.0100). The *p*-score values for Palonosetron + Dexamethasone and Ramosetron + Dexamethasone were similar (Table 4).

#### Transitivity and sensitivity analyses

No statistically significant differences were observed in mean age across the treatment groups. Although a significant difference in publication year was observed—a finding attributable to the sequential market introduction of the first- and second-generation agents—subsequent regression analysis demonstrated that the year of publication exerted no significant influence on treatment efficacy, indicating the robustness of the results (Supplemental Information 14).

Two studies were pediatric trials (Aksoylar et al., 2001; Tan et al., 2018), and the pooled estimates were not significantly impacted by the exclusion of data from these studies. A sensitivity analysis was conducted by excluding one study (Roila et al., 1991) that was not analyzed according to the intention-to-treat (ITT) principle. This exclusion did not significantly change the results of the meta-analysis . And we also have demonstrated the robustness of the primary results through Bayesian sensitivity analysis (Supplemental Information 3).

#### Publication bias

No evidence of significant publication bias was detected for any of the six outcome measures (acute nausea, acute vomiting, acute complete control, delayed nausea, delayed vomiting and delayed complete control) assessed. Although the contour-enhanced funnel plot exhibits asymmetry, the majority of study points fall within the region of significance at *p* < 0.05. This suggests that the asymmetry may be attributed more to small sample effects and heterogeneity among studies rather than to pure publication bias (Supplemental Information 15).

## Discussion

The comparative efficacy of 5-HT_3_ antagonists, administered with or without Dexamethasone, for the management of nausea, vomiting, and complete control in highly emetogenic chemotherapy (HEC) was evaluated in this study. The principal conclusions derived from the study are as follows:

### Palonosetron is generally more effective than first-generation 5-HT_3_ receptor antagonists, with a significantly greater advantage over Ondansetron in the delayed phase

Palonosetron consistently achieved higher *p*-score ratings compared to 1^st^ 5-HT_3_ antagonists in this study. In the acute phase, a significant advantage of Palonosetron over Granisetron and Tropisetron was observed only in acute vomiting in indirect comparisons. Palonosetron demonstrated statistically significant superiority over Ondansetron in the delayed phases. Generally, as Palonosetron is associated with prolonged inhibition of the 5-HT_3_ receptor and thus differs from 1^st^ 5-HT_3_ antagonists, it is reasonable to conclude that Palonosetron exerts superior antiemetic effects in the delayed phase (Rojas et al., 2010a; Rojas et al., 2010b). The superior efficacy of Palonosetron over Ondansetron in the delayed phase, as identified in a prior review (Nisaurrahmah, Sri & Ifora, 2021), is corroborated by the results of this study. A meta-analysis revealed that the use of Palonosetron resulted in a statistically significant improvement in chemotherapy-induced vomiting control compared to either Granisetron or Ondansetron in HEC (Patel et al., 2022). A pooled Phase III analysis confirmed Palonosetron’s superior efficacy over 1^st^ 5-HT_3_ antagonists in delayed-phase CINV prevention, but showed no significant advantage in acute-phase control (Schwartzberg et al., 2014). A meta-analysis also determined that Palonosetron did not demonstrate superiority over first-generation 5-HT_3_ receptor antagonists in the prevention of acute CINV. However, Palonosetron was found to provide significant protection against delayed CINV, particularly in patients receiving highly emetogenic chemotherapy (HEC) (Jin et al., 2013). But a meta-analysis (Chow et al., 2018) found that there were no statistically significant differences between Palonosetron and 1^st^ 5-HT_3_ antagonists in acute vomiting, delayed vomiting, acute complete control, delayed complete control, and concluded that Palonosetron was not superior to 1^st^ 5-HT_3_ antagonists. Additionally, other articles suggested that Palonosetron’s efficacy was not significantly better than that of other 5-HT_3_ antagonists and given the cost differences among these agents, Palonosetron may not offer favorable cost-effectiveness (Du et al., 2017; Kolesar, Eickhoff & Vermeulen, 2014; Hatoum et al., 2012; Avritscher et al., 2010).

### Dexamethasone might significantly increase the efficacy of 1^st^ 5-HT_3_ antagonists in all phases. However, it does not provide a similar benefit for Palonosetron

This study found that when Dexamethasone was added to 1^st^ 5-HT_3_ antagonists, the efficacy of 1^st^ 5-HT_3_ antagonists + Dexamethasone was significantly better than 1^st^ 5-HT_3_ antagonists alone. There were significant differences between Ramosetron and Ramosetron + Dexamethasone (R+D), between Tropisetron and Tropisetron + Dexamethasone (T+D) across all outcome measures in all phases. Similarly, the efficacy of Ondansetron and Ondansetron + Dexamethasone (O+D) showed significant differences in acute nausea, acute vomiting, delayed nausea, and delayed complete control. Granisetron and Granisetron + Dexamethasone (G+D) exhibited significant differences in acute nausea and acute vomiting. However, when examining Palonosetron, Dexamethasone did not significantly enhance Palonosetron’s effectiveness. Palonosetron + Dexamethasone (P+D) did not demonstrate significantly improved efficiency over Palonosetron in any outcome measures, although the *p*-score ratings of Palonosetron + Dexamethasone was higher than Palonosetron. Many articles have indicated that corticosteroids can enhance the effectiveness of other antiemetics. A meta-analysis concluded that adding Dexamethasone enhanced the efficacy of 5-HT_3_ receptor antagonists. However, this conclusion was based solely on studies of first-generation antagonists and did not include Palonosetron (Ioannidis, Hesketh & Lau, 2000). Another Network Meta-Analysis (Yokoe et al., 2019) showed that there was no significant difference between Palonosetron and Palonosetron + Dexamethasone in overall complete response rates (OR1.26, 95%CI [0.85–1.86]). Given the absence of direct comparisons in this study, further research may be warranted to fully understand the influence of Dexamethasone on Palonosetron. The divergent effects of Dexamethasone on 1^st^ 5-HT_3_ antagonists and Palonosetron may be attributed to various factors. Some studies suggested that 1^st^ 5-HT_3_ antagonists may have less success in managing delayed emesis, even with repeated dosing (Aapro et al., 2003; Huang et al., 2004). However, since Dexamethasone was effective for both acute and delayed CINV (National Comprehensive Cancer Network (NCCN), 2025; Herrstedt, lindberg & Petersen, 2022), the effect of 1^st^ 5-HT_3_ antagonists + Dexamethasone was greatly increased throughout the whole treatment period. The combination of Dexamethasone with 1^st^ 5-HT_3_ antagonists receptor antagonists demonstrates superior efficacy compared to 5-HT_3_ receptor antagonists monotherapy in the prevention of CINV (Grunberg, 2007). However, compared to 1^st^ 5-HT_3_ antagonists, Palonosetron exhibits superior antiemetic efficacy due to its long plasma elimination half-life and higher receptor binding affinity (Navari, 2009) and triggering of receptor internalization leading to prolonged inhibition of receptor function and NK1 cross talk (Rojas et al., 2010b). This advantage positions Palonosetron as particularly effective during the delayed phase. Unlike 1^st^ 5-HT_3_ antagonists, Palonosetron by itself might have a good antiemetic effect, especially during the delay period. For Palonosetron, the addition of Dexamethasone appears to have more synergistic than critically enhancing role. Therefore, in contrast to 1^st^ 5-HT_3_ antagonists, Dexamethasone does not significantly enhance Palonosetron ’s efficacy. This inference requires confirmation through further research.

### Palonosetron + Dexamethasone demonstrates superior efficacy over both Ondansetron + Dexamethasone and Granisetron + Dexamethasone, particularly during the delayed phase. However, no significant differences in efficacy were observed between Tropisetron/Ramosetron plus Dexamethasone and Palonosetron + Dexamethasone, or between first-generation 5-HT _**3**_ antagonists combined with Dexamethasone and Palonosetron

Based on *p*-score ratings, the combination of Palonosetron and Dexamethasone emerged as the most effective therapeutic strategy in this study. In the delayed phase, Palonosetron + Dexamethasone demonstrated statistically significant superiority over both Ondansetron + Dexamethasone and Granisetron + Dexamethasone. However, in the acute phase, Palonosetron + Dexamethasone showed statistically significant superiority over Ondansetron + Dexamethasone only in preventing acute vomiting.

The indirect comparison analysis revealed no significant differences in any outcome measures between the Ramosetron + Dexamethasone and Palonosetron + Dexamethasone groups, or between the Tropisetron + Dexamethasone and Palonosetron + Dexamethasone groups. A network meta-analysis found Palonosetron+ Dexamethasone superior to both Ondansetron+Dexamethasone and Granisetron+Dexamethasone in achieving complete response during the overall treatment phase among HEC patients (Piechotta et al., 2021). But another meta-analysis suggested that Palonosetron + Dexamethasone (P+D) might not be the preferred option compared to 1^st^ 5-HT_3_ antagonists combined with Dexamethasone. However, it is important to note that that meta-analysis evaluated 1^st^5-HT_3_ antagonists collectively as a class rather than individually (Chow et al., 2018).

This study concluded that Palonosetron + Dexamethasone outperformed Ondansetron + Dexamethasone and Granisetron+Dexamethasone, especially during the delayed period, although not surpassing Tropisetron + Dexamethasone or Ramosetron + Dexamethasone in all outcome measures. Currently, no studies have directly compared the efficacy of Tropisetron + Dexamethasone or Ramosetron + Dexamethasone with Palonosetron + Dexamethasone for the prevention of nausea and vomiting in highly emetogenic chemotherapy (HEC). Indirect comparisons in this study suggest that there is no evidence demonstrating significant differences in efficacy between Tropisetron + Dexamethasone or Ramosetron + Dexamethasone and Palonosetron+ Dexamethasone in the HEC setting . Further research is needed to determine whether Tropisetron + Dexamethasone or Ramosetron + Dexamethasone could serve as viable alternatives to Palonosetron+ Dexamethasone.

In this study, there were no significant differences in outcome measures between 1^st^ 5-HT_3_ antagonists + Dexamethasone and Palonosetron. The *p*-score of Palonosetron is comparatively close to that of 1^st^ 5-HT_3_ antagonists + Dexamethasone.These conclusions were drawn from indirect comparisons. It might be necessary to conduct a direct comparative study between 1^st^ 5-HT_3_ antagonists + Dexamethasone and Palonosetron in the future to validate these findings.

However, there were still some limitations in this study. Given the complexity of highly emetogenic regimens, no assessment was conducted regarding heterogeneity in chemotherapy regimens, dosing schedules. To focus on the efficacy analysis and comply with space constraints, outcomes such as adverse effects or safety profiles, cost-effectiveness assessment were not reported. The number of articles for some agents (*e.g.*, tropisetron, azasetron) was limited and some studies had a relatively small sample size, potentially influencing the outcome analysis.

## Conclusions

This network meta-analysis on the prevention of vomiting and nausea in highly emetogenic chemotherapy (HEC), indicates that Dexamethasone should be co-administered with first-generation 5-HT_3_ antagonists whenever clinically feasible. For patients who are unable to receive Dexamethasone, Palonosetron may be considered the preferred therapeutic option based on its established efficacy advantage over 1^st^ 5-HT_3_ antagonists during the delayed phase, particularly in comparison with Ondansetron. Overall, based on the network estimates, the Palonosetron+ Dexamethasone ranks the highest, especially during the delayed phase; however, the direct evidence remains limited, and thus the findings should be interpreted considering the inconsistent assessments. No evidence has been established to indicate a difference in therapeutic efficacy between Palonosetron and 1^st^ 5-HT_3_ antagonists + Dexamethasone in all outcomes. Future rigorously designed trials are needed to corroborate these comparative efficacy outcomes.

## Supplemental Information

10.7717/peerj.21047/supp-1 Supplemental Information 1 PRISMA checklist

10.7717/peerj.21047/supp-2 Supplemental Information 2 Covariates across the comparisons

10.7717/peerj.21047/supp-3 Supplemental Information 3 Risk of bias rating of studies

10.7717/peerj.21047/supp-4 Supplemental Information 4 Sensitivity analyses

10.7717/peerj.21047/supp-5 Supplemental Information 5 The leave-one-out analysis

10.7717/peerj.21047/supp-6 Supplemental Information 6 The heterogeneity within the network

10.7717/peerj.21047/supp-7 Supplemental Information 7 Inconsistency between studies

10.7717/peerj.21047/supp-8 Supplemental Information 8 Netsplit of the outcomes

10.7717/peerj.21047/supp-9 Supplemental Information 9 Forest diagram of acute nausea

10.7717/peerj.21047/supp-10 Supplemental Information 10 Forest diagram of acute vomiting

10.7717/peerj.21047/supp-11 Supplemental Information 11 Forest diagram of acute complete control

10.7717/peerj.21047/supp-12 Supplemental Information 12 Forest diagram of delayed nausea

10.7717/peerj.21047/supp-13 Supplemental Information 13 Forest diagram of delayed vomiting

10.7717/peerj.21047/supp-14 Supplemental Information 14 Forest diagram of delayed complete control

10.7717/peerj.21047/supp-15 Supplemental Information 15 Transitivity analyses

10.7717/peerj.21047/supp-16 Supplemental Information 16 Funnel plot

10.7717/peerj.21047/supp-17 Supplemental Information 17 Raw Data

10.7717/peerj.21047/supp-18 Supplemental Information 18 Search strategies for all databases

## Additional Information and Declarations

### Data Availability

The following information was supplied regarding data availability: This is a Systematic Review/Meta-analysis.
