# Supplementary material for: Comparative efficacy of 5-hydroxytryptamine-3 (5-HT3) receptor antagonists with or without dexamethasone for prevention of chemotherapy-induced nausea and vomiting following highly emetogenic chemotherapy (HEC): a network meta-analysis
Source: PeerJ. 2026 Apr 2;14:e21047. doi: 10.7717/peerj.21047 (PMC13050518; doi:10.7717/peerj.21047)
Supplement: Supplemental Information 1 [file peerj-14-21047-s001.docx]

| **Section and Topic** | **Item #** | **Checklist item** | **Location where item is reported** |
| --- | --- | --- | --- |
| **TITLE** | | |  |
| Title | 1 | Identify the report as a systematic review. | 1  Comparative Efficacy of 5-HT3 Receptor Antagonists with or without Dexamethasone for Prevention of Chemotherapy-Induced Nausea and Vomiting: A Network Meta-analysis |
| **ABSTRACT** | | |  |
| Abstract | 2 | See the PRISMA 2020 for Abstracts checklist. | 19-53  Abstract  Objective：This network meta-analysis evaluated the efficacy of 5-hydroxytryptamine-3 （5-HT3）receptor antagonists, with or without Dexamethasone (D), for preventing chemotherapy-induced nausea and vomiting (CINV) in patients undergoing highly emetogenic chemotherapy (HEC) who were limited to these regimens.  Methods：The randomized controlled studies (RCTs) were searched in PubMed, Embase, Cochrane and Web of Science from their inception up to March 31, 2025, to identify patients who used 5-HT3 receptor antagonists (with or without Dexamethasone) to prevent nausea and vomiting caused by highly emetogenic chemotherapy. The outcome measures included acute nausea, acute vomiting, acute complete control, delayed nausea, delayed vomiting and delayed complete control.  Results：A total of 36 randomized controlled studies (reported in 37 articles ) were included in this analysis. Palonosetron (P) is generally more effective than first-generation 5-HT3 receptor antagonists（1st 5-HT3 antagonists）, with a significantly greater advantage over Ondansetron in the delayed phase (RR0.70, 95% CI 0.49-0.99). In all phases, adding Dexamethasone to 1st 5-HT3 antagonists significantly improved efficacy compared to using them alone. However, combining Dexamethasone with Palonosetron showed no significant advantage over Palonosetron alone in any outcome measure in indirect comparisons. In the delayed phase, Palonosetron + Dexamethasone (P+D) demonstrated statistically significant superiority over both Ondansetron + Dexamethasone (O+D) and Granisetron + Dexamethasone (G+D) in direct comparisons. However, in the acute phase, Palonosetron + Dexamethasone showed statistically significant superiority over Ondansetron + Dexamethasone (RR 0.81, 95% CI 0.69–0.96) only in preventing acute vomiting. In contrast, no significant efficacy differences were observed between Palonosetron + Dexamethasone and Tropisetron + Dexamethasone (T+D), or Ramosetron + Dexamethasone (R+D). No significant differences were observed between Paonosetron and 1st 5-HT3 antagonists + Dexamethasone.  Conclusions： Dexamethasone may enhance the efficacy of first-generation 5-HT₃ receptor antagonists; however, it may not provide a similar benefit for Palonosetron. Palonosetron is generally more effective than first-generation 5-HT3 antagonists, with significantly superior efficacy over Ondansetron in the delayed phase. Palonosetron + Dexamethasone demonstrates superior efficacy over Ondansetron + Dexamethasone or Granisetron + Dexamethasone, particularly in the delayed phase. Further studies are needed between Palonosetron + Dexamethasone and Tropisetron + Dexamethasone or Ramosetron + Dexamethasone, as well as between Palonosetron monotherapy and 1st 5-HT3 antagonists + Dexamethasone. |
| **INTRODUCTION** | | |  |
| Rationale | 3 | Describe the rationale for the review in the context of existing knowledge. | 63-89  Currently, guidelines recommend three or four regimens for HEC, including 5-HT3 receptor antagonist (5-HT3RA), Dexamethasone (D), Neurokinin 1 receptor antagonist（NK1RA）or Olanzapine [3, 5, 6]. However, suboptimal adherence to guidelines was observed in highly emetogenic chemotherapy. Specifically, the guideline-recommended regimen consisting of NK1RA, 5-HT3RA, and Dexamethasone was prescribed to only 12.2%-15% of patients, while the four-drug combination that includes Olanzapine in addition to these agents was used in merely 2.1% of cases [7, 8, 9]. Guideline adherence in clinical practice has shown a sluggish advancement in recent periods [10]. Several factors contribute to the unsatisfactory compliance with the guidelines：A lack of awareness of antiemetic guidelines among physicians [7], the limited availability of certain drugs [11], financial constraints in developing countries, especially in rural areas [12, 13], and the exclusion of newer antiemetic agents from national medical insurance formularies [14]. 5-HT3 antagonists play a critical role in antiemetic therapy in chemotherapy patients [3]. Dexamethasone and 5-HT3 antagonists are used by most patients receiving HEC [15].  Prophylaxis with 5-HT₃ receptor antagonists and Dexamethasone, whether used as monotherapy or in combination, is insufficient for the prevention of chemotherapy-induced nausea and vomiting (CINV) following HEC. However, owing to a variety of constraints [7, 11, 12, 13, 14], patients access to antiemetic therapy is often limited to a narrow range of options.  In such scenarios, a more detailed comparison of the efficacy of various 5-HT₃ antagonists—both as monotherapy and in combination with Dexamethasone—may be needed: for example, although Palonosetron is widely recognized as exhibiting superior efficacy compared to first-generation 5-HT₃ antagonists (1st 5-HT₃ antagonists) [3], it remains unclear more detail question, such as , whether Palonosetron monotherapy outperforms a regimen combining a 1st 5-HT₃ antagonists with Dexamethasone? What is the magnitude of the enhancement provided by Dexamethasone to the antiemetic effect of 5-HT₃ antagonists in highly emetogenic chemotherapy regimens? Is it possible to determine which treatment regimen holds a superior therapeutic advantage over others in highly emetogenic chemotherapy? |
| Objectives | 4 | Provide an explicit statement of the objective(s) or question(s) the review addresses. | 90-107  However, existing network meta-analyses fail to provide the comparative conclusions outlined above：some studies have evaluated all first-generation 5-HT₃ receptor antagonists as a unified class rather than comparing them individually [16, 17, 18]；some analyses despite comparing different 5-HT₃ antagonists separately, did not encompass all the available 5-HT₃ antagonists [19]; some were limited to monotherapy comparisons of 5-HT₃ antagonists without evaluating combination regimens with Dexamethasone [20]; while others were limited by restricted patients（only pediatric patients included）inclusion criteria [21].  Based on the current situation where a significant proportion of patients continue to receive only 5-HT3 antagonists (with or without Dexamethasone) for HEC, on the one hand, it is necessary to take a variety of measures to push physicians to adhere to the guidelines by selecting from the recommended combinations of three or four antiemetic drugs for HEC, while a refined selection program of 5-HT3 antagonists (with or without Dexamethasone) used in HEC should be provided for clinical reference. This study aims to conduct a comprehensive network meta-analysis comparing the efficacy of individual 5-HT3 receptor antagonists, with or without Dexamethasone, for the prevention of nausea, vomiting, and complete control in patients undergoing highly emetogenic chemotherapy. The objective is to provide optimized antiemetic regimens for patients who, due to various constraints, can only receive 5-HT₃ antagonists (with or without Dexamethasone) for the prevention of nausea and vomiting during HEC. |
| **METHODS** | | |  |
| Eligibility criteria | 5 | Specify the inclusion and exclusion criteria for the review and how studies were grouped for the syntheses. | 147-160  2.3 Study selection criteria  Study inclusion criteria  (1) Patients receiving highly emetogenic chemotherapy (HEC) who were administered 5-HT3 antagonists, with or without Dexamethasone, for the prevention of nausea and vomiting were included.  (2) Only randomized controlled trials (RCTs) were included.  (3) Only English literature was included.  Study exclusion criteria  (1) Patients who received low, moderate, or mixed emetogenic chemotherapy regimens were excluded and the trials that reported radiotherapy-induced nausea and vomiting were excluded.  (2) Patients who received 5-HT3 antagonists with other antiemetic drugs except for Dexamethasone, were excluded.  (3) Non-RCTs, animal experiments, conference abstracts, protocols, reviews, letters, retrospective studies were excluded. |
| Information sources | 6 | Specify all databases, registers, websites, organisations, reference lists and other sources searched or consulted to identify studies. Specify the date when each source was last searched or consulted. | 114-116  This network meta-analysis was performed in accordance with the Preferred Reporting Items for Systematic Reviews and Meta-Analyses (PRISMA) guidelines [22]. The protocol for this analysis was registered under the reference CRD42023403570. |
| Search strategy | 7 | Present the full search strategies for all databases, registers and websites, including any filters and limits used. | 125-145，Supplement 15  **PubMed**  The search strategy used in PubMed was as followed: "Ondansetron" [Mesh] OR Ondansetron, (+,-)-Isomer [Title/Abstract] OR Ondansetron Hydrochloride [Title/Abstract] OR Hydrochloride, Ondansetron [Title/Abstract] OR Ondansetron Monohydrochloride [Title/Abstract] OR Monohydrochloride, Ondansetron [Title/Abstract] OR Ondansetron Monohydrochloride Dihydrate [Title/Abstract] OR Dihydrate, Ondansetron Monohydrochloride [Title/Abstract] OR Monohydrochloride Dihydrate, Ondansetron [Title/Abstract] OR Ondansetron, (S)-Isomer [Title/Abstract] OR Zofran [Title/Abstract] OR Ondansetron, (R)-Isomer [Title/Abstract] OR "Granisetron" [Mesh] OR Kytril [Title/Abstract] OR Granisetron Hydrochloride [Title/Abstract] OR Hydrochloride, Granisetron [Title/Abstract]) OR (Granisetron Monohydrochloride [Title/Abstract] OR Monohydrochloride, Granisetron [Title/Abstract] OR "dolasetron" [Supplementary Concept] OR dolasetron mesylate [Title/Abstract] OR dolasetron mesylate monohydrate [Title/Abstract] OR dolasetronmesilate monohydrate [Title/Abstract] OR Anzemet [Title/Abstract] OR "Tropisetron" [Mesh] OR Navoban [Title/Abstract] OR Indole 3 carboxylic Acid Tropine Ester [Title/Abstract] OR Tropisetron Hydrochloride [Title/Abstract] OR "ramosetron" [Supplementary Concept] OR ramosetron hydrochloride [Title/Abstract] OR Nasea [Title/Abstract] OR ("azasetron" [Supplementary Concept]) OR ("azasetron" [Supplementary Concept] OR azasetron, (+-)-isomer [Title/Abstract]) OR "Palonosetron" [Mesh]) OR Palonosetron, (R-(R*,R*))-isomer [Title/Abstract] OR Palonosetron, 3R [Title/Abstract] OR Palonosetron, (R-(R*,S*))-isomer [Title/Abstract] OR Aloxi [Title/Abstract] OR Palonosetron, (S-(R*,S*))-isomer [Title/Abstract] OR Palonosetron Hydrochloride [Title/Abstract].  **Embase**  (((ondansetron,:ab,ti AND +,-:ab,ti AND -isomer:ab,ti OR 'ondansetron hydrochloride':ab,ti OR 'hydrochloride, ondansetron':ab,ti OR 'ondansetron monohydrochloride':ab,ti OR 'monohydrochloride, ondansetron':ab,ti OR 'ondansetron monohydrochloride dihydrate':ab,ti OR 'dihydrate, ondansetron monohydrochloride':ab,ti OR 'monohydrochloride dihydrate, ondansetron':ab,ti OR (ondansetron,:ab,ti AND s:ab,ti AND -isomer:ab,ti) OR zofran:ab,ti OR (ondansetron,:ab,ti AND r:ab,ti AND -isomer:ab,ti)) OR ('ondansetron'/exp OR 'ondansetron') OR (kytril:ab,ti OR 'granisetron hydrochloride':ab,ti OR 'hydrochloride, granisetron':ab,ti OR 'granisetron monohydrochloride':ab,ti OR 'monohydrochloride, granisetron':ab,ti) OR ('dolasetron mesylate':ab,ti OR 'dolasetron mesylate monohydrate':ab,ti OR 'dolasetron mesilate monohydrate':ab,ti OR anzemet:ab,ti) OR ('tropisetron'/exp OR 'tropisetron') OR ('dolasetron mesilate'/exp OR 'dolasetron mesilate') OR ('granisetron'/exp OR 'granisetron') OR (navoban:ab,ti OR 'indole 3 carboxylic acid tropine ester':ab,ti OR 'tropisetron hydrochloride':ab,ti) OR ('ramosetron'/exp OR 'ramosetron') OR ('ramosetron hydrochloride':ab,ti OR nasea:ab,ti) OR ('azasetron'/exp OR 'azasetron') OR (azasetron,:ab,ti AND +-:ab,ti AND -isomer:ab,ti) OR ('palonosetron'/exp OR 'palonosetron') OR (palonosetron,:ab,ti AND r-:ab,ti AND r*,r*:ab,ti AND -isomer:ab,ti OR (palonosetron,:ab,ti AND 3r:ab,ti AND -:ab,ti) OR (palonosetron,:ab,ti AND r-:ab,ti AND r*,s*:ab,ti AND -isomer:ab,ti) OR aloxi:ab,ti OR (palonosetron,:ab,ti AND s-:ab,ti AND r*,s*:ab,ti AND -isomer:ab,ti) OR 'palonosetron hydrochloride':ab,ti)) AND [01-01-2025]/sd NOT [01-04-2025]/sd AND [<1966-2025]/py) OR (((((ondansetron,:ab,ti AND +,-:ab,ti AND -isomer:ab,ti OR 'ondansetron hydrochloride':ab,ti OR 'hydrochloride, ondansetron':ab,ti OR 'ondansetron monohydrochloride':ab,ti OR 'monohydrochloride, ondansetron':ab,ti OR 'ondansetron monohydrochloride dihydrate':ab,ti OR 'dihydrate, ondansetron monohydrochloride':ab,ti OR 'monohydrochloride dihydrate, ondansetron':ab,ti OR (ondansetron,:ab,ti AND s:ab,ti AND -isomer:ab,ti) OR zofran:ab,ti OR (ondansetron,:ab,ti AND r:ab,ti AND -isomer:ab,ti)) OR ('ondansetron'/exp OR 'ondansetron') OR (kytril:ab,ti OR 'granisetron hydrochloride':ab,ti OR 'hydrochloride, granisetron':ab,ti OR 'granisetron monohydrochloride':ab,ti OR 'monohydrochloride, granisetron':ab,ti) OR ('dolasetron mesylate':ab,ti OR 'dolasetron mesylate monohydrate':ab,ti OR 'dolasetron mesilate monohydrate':ab,ti OR anzemet:ab,ti) OR ('tropisetron'/exp OR 'tropisetron') OR ('dolasetron mesilate'/exp OR 'dolasetron mesilate') OR ('granisetron'/exp OR 'granisetron') OR (navoban:ab,ti OR 'indole 3 carboxylic acid tropine ester':ab,ti OR 'tropisetron hydrochloride':ab,ti) OR ('ramosetron'/exp OR 'ramosetron') OR ('ramosetron hydrochloride':ab,ti OR nasea:ab,ti) OR ('azasetron'/exp OR 'azasetron') OR (azasetron,:ab,ti AND +-:ab,ti AND -isomer:ab,ti) OR ('palonosetron'/exp OR 'palonosetron') OR (palonosetron,:ab,ti AND r-:ab,ti AND r*,r*:ab,ti AND -isomer:ab,ti OR (palonosetron,:ab,ti AND 3r:ab,ti AND -:ab,ti) OR (palonosetron,:ab,ti AND r-:ab,ti AND r*,s*:ab,ti AND -isomer:ab,ti) OR aloxi:ab,ti OR (palonosetron,:ab,ti AND s-:ab,ti AND r*,s*:ab,ti AND -isomer:ab,ti) OR 'palonosetron hydrochloride':ab,ti)) AND [01-01-2025]/sd NOT [01-04-2025]/sd AND [<1966-2025]/py) OR (((ondansetron,:ab,ti AND +,-:ab,ti AND -isomer:ab,ti OR 'ondansetron hydrochloride':ab,ti OR 'hydrochloride, ondansetron':ab,ti OR 'ondansetron monohydrochloride':ab,ti OR 'monohydrochloride, ondansetron':ab,ti OR 'ondansetron monohydrochloride dihydrate':ab,ti OR 'dihydrate, ondansetron monohydrochloride':ab,ti OR 'monohydrochloride dihydrate, ondansetron':ab,ti OR (ondansetron,:ab,ti AND s:ab,ti AND -isomer:ab,ti) OR zofran:ab,ti OR (ondansetron,:ab,ti AND r:ab,ti AND -isomer:ab,ti)) OR ('ondansetron'/exp OR 'ondansetron') OR (kytril:ab,ti OR 'granisetron hydrochloride':ab,ti OR 'hydrochloride, granisetron':ab,ti OR 'granisetron monohydrochloride':ab,ti OR 'monohydrochloride, granisetron':ab,ti) OR ('dolasetron mesylate':ab,ti OR 'dolasetron mesylate monohydrate':ab,ti OR 'dolasetron mesilate monohydrate':ab,ti OR anzemet:ab,ti) OR ('tropisetron'/exp OR 'tropisetron') OR ('dolasetron mesilate'/exp OR 'dolasetron mesilate') OR ('granisetron'/exp OR 'granisetron') OR (navoban:ab,ti OR 'indole 3 carboxylic acid tropine ester':ab,ti OR 'tropisetron hydrochloride':ab,ti) OR ('ramosetron'/exp OR 'ramosetron') OR ('ramosetron hydrochloride':ab,ti OR nasea:ab,ti) OR ('azasetron'/exp OR 'azasetron') OR (azasetron,:ab,ti AND +-:ab,ti AND -isomer:ab,ti) OR ('palonosetron'/exp OR 'palonosetron') OR (palonosetron,:ab,ti AND r-:ab,ti AND r*,r*:ab,ti AND -isomer:ab,ti OR (palonosetron,:ab,ti AND 3r:ab,ti AND -:ab,ti) OR (palonosetron,:ab,ti AND r-:ab,ti AND r*,s*:ab,ti AND -isomer:ab,ti) OR aloxi:ab,ti OR (palonosetron,:ab,ti AND s-:ab,ti AND r*,s*:ab,ti AND -isomer:ab,ti) OR 'palonosetron hydrochloride':ab,ti)) AND [<1966-2025]/py)) AND [<1966-2024]/py)  **Cochrane**  #1 MeSH descriptor: [Ondansetron] explode all trees  #2 (Ondansetron, Isomer):ti,ab,kw OR (Ondansetron Hydrochloride):ti,ab,kw OR (Hydrochloride, Ondansetron):ti,ab,kw OR (Ondansetron Monohydrochloride):ti,ab,kw OR (Monohydrochloride, Ondansetron):ti,ab,kw (Word variations have been searched)  #3 (Ondansetron Monohydrochloride Dihydrate):ti,ab,kw OR (Dihydrate, Ondansetron Monohydrochloride):ti,ab,kw OR (Monohydrochloride Dihydrate, Ondansetron):ti,ab,kw OR (Ondansetron, (S) Isomer):ti,ab,kw OR (Zofran):ti,ab,kw (Word variations have been searched)  #4 (Ondansetron, (R) Isomer):ti,ab,kw (Word variations have been searched)  #5 MeSH descriptor: [Granisetron] explode all trees  #6 (Kytril):ti,ab,kw OR (Granisetron Hydrochloride):ti,ab,kw OR (Hydrochloride, Granisetron):ti,ab,kw OR (Granisetron Monohydrochloride):ti,ab,kw OR (Monohydrochloride, Granisetron):ti,ab,kw (Word variations have been searched)  #7 MeSH descriptor: [] explode all trees  #8 (dolasetron mesylate):ti,ab,kw OR (dolasetron mesylate monohydrate):ti,ab,kw OR (dolasetron mesilate monohydrate):ti,ab,kw OR (Anzemet):ti,ab,kw (Word variations have been searched)  #9 MeSH descriptor: [Tropisetron] explode all trees  #10 (Navoban):ti,ab,kw OR (Indole 3 carboxylic Acid Tropine Ester):ti,ab,kw OR (Tropisetron Hydrochloride):ti,ab,kw (Word variations have been searched)  #11 MeSH descriptor: [] explode all trees  #12 (ramosetron hydrochloride):ti,ab,kw OR (Nasea):ti,ab,kw (Word variations have been searched)  #13 MeSH descriptor: [] explode all trees  #14 (azasetron, isomer):ti,ab,kw (Word variations have been searched)  #15 MeSH descriptor: [Palonosetron] explode all trees  #16 (Palonosetron, (R (R*,R*)) isomer):ti,ab,kw OR (Palonosetron, (3R)):ti,ab,kw OR (Palonosetron, (R (R*,S*)) isomer):ti,ab,kw OR (Aloxi):ti,ab,kw OR (Palonosetron, (S (R*,S*)) isomer):ti,ab,kw (Word variations have been searched)  #17 (Palonosetron Hydrochloride):ti,ab,kw (Word variations have been searched)  #18 #1 or #2 or #3 or #4 or #5 or #6 or #7 or #8 or #9 or #10 or #11 or #12 or #13 or #14 or #15 or #16 or #17  #19 1 or #2 or #3 or #4 or #5 or #6 or #7 or #8 or #9 or #10 or #11 or #12 or #13 or #14 or #15 or #16 or #17 with Cochrane Library publication date Between Jan 1950 and Mar 2025  **Web of Science**  "(((((((((((TS=(Ondansetron)) OR TS=(Ondansetron, (+,-)-Isomer)) OR TS=(Ondansetron Hydrochloride)) OR TS=(Hydrochloride, Ondansetron)) OR TS=(Ondansetron Monohydrochloride)) OR TS=(Monohydrochloride, Ondansetron)) OR TS=(Ondansetron Monohydrochloride Dihydrate)) OR TS=(Dihydrate, Ondansetron Monohydrochloride)) OR TS=(Monohydrochloride Dihydrate, Ondansetron)) OR TS=(Ondansetron, (S)-Isomer)) OR TS=(Zofran)) OR TS=(Ondansetron, (R)-Isomer) and Preprint Citation Index  "(((((TS=(Granisetron)) OR TS=(Kytril)) OR TS=(Granisetron Hydrochloride)) OR TS=(Hydrochloride, Granisetron)) OR TS=(Granisetron Monohydrochloride)) OR TS=(Monohydrochloride, Granisetron) and Preprint Citation Index  "((((TS=(Dolasetron)) OR TS=(dolasetron mesylate)) OR TS=(dolasetron mesylate monohydrate)) OR TS=(dolasetron mesilate monohydrate)) OR TS=(Anzemet) and Preprint Citation Index  "(((TS=(Tropisetron)) OR TS=(Navoban)) OR TS=(Indole 3 carboxylic Acid Tropine Ester)) OR TS=(Tropisetron Hydrochloride) and Preprint Citation Index  "((TS=(Ramosetron)) OR TS=(ramosetron hydrochloride)) OR TS=(Nasea) and Preprint Citation Index  "(TS=(Azasetron)) OR TS=(azasetron, (+-)-isomer) and Preprint Citation Index  "((((((TS=(Palonosetron)) OR TS=(Palonosetron, (R-(R, R))-isomer)) OR TS=(Palonosetron, (3R))) OR TS=(Palonosetron, (R-(R,S))-isomer)) OR TS=(Aloxi)) OR TS=(Palonosetron, (S-(R,S))-isomer)) OR TS=(Palonosetron Hydrochloride) and Preprint Citation Index  "#160 OR #128 OR #127 OR #123 OR #119 OR #109 OR #13 and Preprint Citation Index  "#163 and Preprint Citation Index 入库时间: 1950-01-01 to 2025-03-31 |
| Selection process | 8 | Specify the methods used to decide whether a study met the inclusion criteria of the review, including how many reviewers screened each record and each report retrieved, whether they worked independently, and if applicable, details of automation tools used in the process. | 172-178  2 reviewers (Hongxia Xu and Jiankun Xing) independently worked on the study of identification, selection, quality assessment and data abstraction. A third reviewer (Shaohui Yang) was consulted for any discrepancies. |
| Data collection process | 9 | Specify the methods used to collect data from reports, including how many reviewers collected data from each report, whether they worked independently, any processes for obtaining or confirming data from study investigators, and if applicable, details of automation tools used in the process. | 180-187, 214-219  Two authors (Hongxia Xu and Jiankun Xing) independently extracted information from eligible RCTs, including author, year of publication, characteristics of the population (including number of patients, age, chemotherapy regimen and course, score of status), intervention (including 5-HT3 antagonists with or without Dexamethasone were used), outcome measure, and so on. If any discrepancies between two authors (Hongxia Xu and Jiankun Xing) appeared, a third author (Shaohui Yang) will be consulted.  Most data were obtained from the articles directly, but some data from the first round of chemotherapy [43, 45, 47, 52], some data were derived from average values calculated in studies that reported the daily number of patients experiencing nausea and vomiting in the delayed phase [27, 29, 34, 37, 59]. For cases with multiple sets of data, only data meeting the requirements were extracted [24, 31, 33, 35, 37, 41, 46, 55]. |
| Data items | 10a | List and define all outcomes for which data were sought. Specify whether all results that were compatible with each outcome domain in each study were sought (e.g. for all measures, time points, analyses), and if not, the methods used to decide which results to collect. | 162-170  Definitions of “Acute” and “Late” Periods:  “Acute”: (<24h post-chemotherapy after the first chemotherapy dose )  “Late”: (≥24h post-chemotherapy after the first chemotherapy dose)  In this article, complete control (CC) was defined as no emetic episodes, no use of rescue medication, and experiencing only mild or no nausea. |
|  | 10b | List and define all other variables for which data were sought (e.g. participant and intervention characteristics, funding sources). Describe any assumptions made about any missing or unclear information. | 181-187  Data extracted from the selected studies included the author’s name, publication year, study design, sample size, patient age, interventional drugs, and the outcomes. |
| Study risk of bias assessment | 11 | Specify the methods used to assess risk of bias in the included studies, including details of the tool(s) used, how many reviewers assessed each study and whether they worked independently, and if applicable, details of automation tools used in the process. | 172-178, 222  The risk of bias in the study was assessed by the Cochrane handbook (Version 5.3.5) [23], using the Cochrane risk of bias tool designed for RCTs, which including the indicators of sequence generation, allocation concealment, blinding of participants and outcome assessment, incomplete or selective outcome reporting. The risk of bias was assessed independently by two authors (Hongxia Xu and Jiankun Xing).  With Revman 5.3, a total of 36 trials were evaluated for article quality. (Figure2) |
| Effect measures | 12 | Specify for each outcome the effect measure(s) (e.g. risk ratio, mean difference) used in the synthesis or presentation of results. | 193-196  A random effects model was applied to pool data. Outcomes were reported as Risk Ratios (RR) with 95% credibility intervals (CI), and statistical significance was set at P values less than 0.05.  P-scores were utilized to rank the probability of effectiveness for each intervention. |
| Synthesis methods | 13a | Describe the processes used to decide which studies were eligible for each synthesis (e.g. tabulating the study intervention characteristics and comparing against the planned groups for each synthesis (item #5)). | 147-170  2.3 Study selection criteria  Study inclusion criteria  (1) Patients receiving highly emetogenic chemotherapy (HEC) who were administered 5-HT3 antagonists, with or without Dexamethasone, for the prevention of nausea and vomiting were included.  (2) Only randomized controlled trials (RCTs) were included.  (3) Only English literature was included.  Study exclusion criteria  (1) Patients who received low, moderate, or mixed emetogenic chemotherapy regimens were excluded and the trials that reported radiotherapy-induced nausea and vomiting were excluded.  (2) Patients who received 5-HT3 antagonists with other antiemetic drugs except for Dexamethasone, were excluded.  (3) Non-RCTs, animal experiments, conference abstracts, protocols, reviews, letters, retrospective studies were excluded.  2.4 Outcomes Measures  Ⅰ. Acute nausea (<24h post-chemotherapy after the first chemotherapy dose)  Ⅱ. Acute vomiting (<24h post-chemotherapy after the first chemotherapy dose)  Ⅲ. Acute complete control (<24h post-chemotherapy after the first chemotherapy dose)  In this article, complete control (CC) was defined as no emetic episodes, no use of rescue medication, and experiencing only mild or no nausea.  Ⅳ. Delayed nausea (≥24h post-chemotherapy after the first chemotherapy dose)  Ⅴ. Delayed vomiting (≥24h post-chemotherapy after the first chemotherapy dose)  Ⅵ. Delayed complete control (≥24h post-chemotherapy after the first chemotherapy dose) |
|  | 13b | Describe any methods required to prepare the data for presentation or synthesis, such as handling of missing summary statistics, or data conversions. | 214-219  Most data were obtained from the articles directly, but some data from the first round of chemotherapy [43, 45, 47, 52], some data were derived from average values calculated in studies that reported the daily number of patients experiencing nausea and vomiting in the delayed phase [27, 29, 34, 37, 59]. For cases with multiple sets of data, only data meeting the requirements were extracted [24, 31, 33, 35, 37, 41, 46, 55]. |
|  | 13c | Describe any methods used to tabulate or visually display results of individual studies and syntheses. | 197-202  P-scores were utilized for ranking of treatments, quantifying the level of confidence that one treatment is superior to another, averaged across all competing treatments. The transitivity assumption was assessed by comparing the distribution of potential effect modifiers across comparisons, such as publication years, mean age. Sensitivity analyses were performed by separately excluding studies involving children.  Egger’s test and funnel plot were applied to evaluate publication bias. |
|  | 13d | Describe any methods used to synthesize results and provide a rationale for the choice(s). If meta-analysis was performed, describe the model(s), method(s) to identify the presence and extent of statistical heterogeneity, and software package(s) used. | 190-196, Supplement 2, Supplement 3, Supplement 4,Table 4  R software (version 4.2.1) and STATA 17 were employed in this network meta-analysis to compare different treatments with a frequentist approach and the conclusions of direct and indirect comparisons were obtained.  Heterogeneity indirect-comparison meta-analysis was measured by using the I2 statistic and the Q statistic was employed to evaluate consistency with a random-effects model. We reported the outcome with RR (Risk Ratio), 95%CI (95%credibilityintervals), with statistical significance defined as P＜0.05.  P-scores were utilized for ranking of treatments, quantifying the level of confidence that one treatment is superior to another, averaged across all competing treatments. Egger’s test and funnel plot were applied to evaluate publication bias. |
|  | 13e | Describe any methods used to explore possible causes of heterogeneity among study results (e.g. subgroup analysis, meta-regression). | 198-200, Supplement 11  The transitivity assumption was assessed by comparing the distribution of potential effect modifiers across comparisons, such as publication years，mean age. |
|  | 13f | Describe any sensitivity analyses conducted to assess robustness of the synthesized results. | 200-201, 371-374 Supplement 12  Sensitivity analyses were performed by separately excluding studies involving children.  A sensitivity analysis was conducted by excluding one study (Roila F 1991 [52]) that was not analyzed according to the intention-to-treat (ITT) principle |
| Reporting bias assessment | 14 | Describe any methods used to assess risk of bias due to missing results in a synthesis (arising from reporting biases). | 202, Supplement 13  Egger’s test and funnel plots were employed to assess publication bias in studies with 10 or more trials. |
| Certainty assessment | 15 | Describe any methods used to assess certainty (or confidence) in the body of evidence for an outcome. | 173-176, 222  The risk of bias in the study was assessed by the Cochrane handbook (Version 5.3.5) [23], using the Cochrane risk of bias tool designed for RCTs, which including the indicators of sequence generation, allocation concealment, blinding of participants and outcome assessment, incomplete or selective outcome reporting. The risk of bias was assessed independently by two authors With Revman 5.3, a total of 36 trials were evaluated for article quality. (Figure2) |
| **RESULTS** | | |  |
| Study selection | 16a | Describe the results of the search and selection process, from the number of records identified in the search to the number of studies included in the review, ideally using a flow diagram. | 209-212  57078 articles were found from the database (Figure 1). Finally, 36 experiments [23-58], 37 articles were included in this study. The trial conducted by Kubota K in 2016 [59] was a follow-up study to Saito M's trial in 2009 [53]. Table 1 summarized the characteristics of the RCTs included. 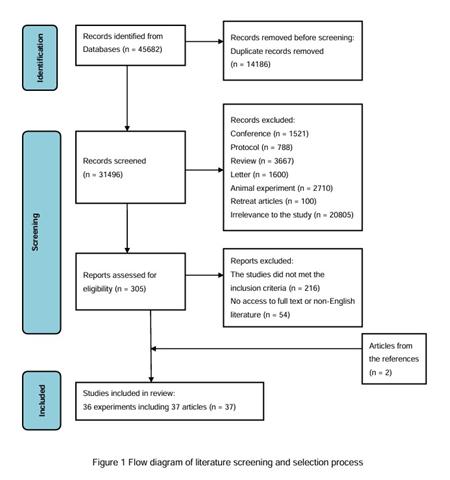 |
|  | 16b | Cite studies that might appear to meet the inclusion criteria, but which were excluded, and explain why they were excluded. | We have not encountered such a situation. |
| Study characteristics | 17 | Cite each included study and present its characteristics. | Table 1   \| Number \| Year \| Test type \| Included population \| Intervention(The number of each group) \| Outcome Indicators \| \| --- \| --- \| --- \| --- \| --- \| --- \| \| Aapro MS 2006 [24] \| 2006 \| RCT Multicenter \| 673 cancer patients, age ≥18 years, including cisplatin≥80mg/m^2^. \| Palonosetron(dose1)+Dexamethasone(150) Palonosetron(dose2)+Dexamethasone(150) Ondansetron+Dexamethasone (147) \| ②⑤ \| \| Aksoylar S 2001[25] \| 2001 \| Prospective RCT \| 51 children, highly emetogenic chemotherapy, in 133 chemotherapy cycles. \| Tropisetron（66）  Granisetron（67） \| ①② \| \| Audhuy B 1996 [26] \| 1996 \| RCT  Multicenter \| 474 cancer patients over 18 years, cisplatin ≥80mg/m^2^. \| Dolasetron(dose1) (163)  Dolasetron(dose2) (161)  Granisetron (150) \| ①② \| \| Cheirsilpa A 2005 [27] \| 2005 \| RCT \| 73 cancer patients, aged between 20 and 80 years, cisplatin ≥70mg/m^2^. \| Ramosetron+Dexamethasone（36） Granisetron+Dexamethasone（37） \| ①②③④⑤⑥ \| \| Dong XR 2011 [28] \| 2011 \| RCT \| 89 patients, non-small cell lung carcinoma, age ≥18 years. \| Palonosetron(44) Ondasetron(45) \| ①②③④⑤⑥ \| \| Fauser A.A. 2000 [29] \| 2000 \| RCT  Multicenter \| 210 cancer patients，age ≥18 years, cisplatin (15-50 mg/m^2^). \| Dolasetron(103) Dolasetrone+Dexamethasone(107) \| ④⑤ \| \| Garcia del Muro X 1998 [30] \| 1998 \| RCT Multicenter \| 278 cancer patients, cisplatin ≥50 mg/m^2^. \| Tropisetron (143）  Tropisetron+Dexamethasone（135） \| ①②③④⑤ \| \| Gebbia V 1994 [31] \| 1994 \| RCT \| 182 cancer patients, cisplatin≥70 mg/m^2^. \| Ondansetron (84) Granisetron (82) \| ②④⑤ \| \| Gralla RJ 1998 [32] \| 1998 \| RCT Multicenter \| 1054 patients with malignant disease, age ≥18 year, cisplatin ≥60mg/m^2^. \| Granisetron+Dexamethasone （417） Ondansetron+Dexamethasone（413） Granisetron（117）  Ondansetron (107） \| ①②③ \| \| Heron J.F. 1994 [33] \| 1994 \| RCT \| 357 cancer patients, cisplatin ≥50mg/m^2^. \| Granisetron （119）  Granisetron+Dexamethasone（117） \| ①②③④⑤⑥ \| \| Hesketh P 1996 [34] \| 1996 \| RCT \| 609 cancer patients, age≥18 years, cisplatin ≥70 mg/m^2^. \| Dolasetron(dose1 (198)  Dolasetron(dose2) (205)  Ondansetron (206) \| ② \| \| Ho CL 2010 [35] \| 2010 \| RCT \| 124 cancer patients, age between 20 and 74 years, cisplatin ≥50mg/m^2^. \| Ramosetron+Dexamethasone（62） Granisetron+Dexamethasone（62） \| ②③ \| \| Italian Group 1995 [36] \| 1995 \| RCT Multicenter \| 966 cancer patients, cisplatin ≥50mg/m^2^. \| Ondansetron+Dexamethasone （483） Granisetron+Dexamethasone（483） \| ①②③④⑤⑥ \| \| Joss R.A. 1994 [37] \| 1994 \| Prospective RCT \| 215 cancer patients, cisplatin ≥50 mg/m^2^. \| Ondansetron (58) Ondansetron+Dexamethasone(53) \| ①②③④⑤⑥ \| \| Kang YK 2002 [38] \| 2002 \| RCT \| 203 cancer patients , 20-75 years, cisplatin ≥50mg/m^2^. \| Ramosetron（94） Granisetron（100） \| ①② \| \| Keyhanian Sh 2009 [39] \| 2009 \| RCT \| 138 cancer patients, aged 15-82 years, 30-80 mg/m^2^ cisplatin or >40mg/m^2^ doxorubicin. \| Granisetron（63） Granisetron+Dexamethasone（62） \| ①② \| \| Kim JS 2004 [40] \| 2004 \| RCT \| 114 cancer patients, age ≥18 years, cisplatin ≥60mg/m^2^. \| Dolasetron（56） Ondansetron（58） \| ①②④⑤ \| \| Latreille J 1995 [41] \| 1995 \| RCT \| 292 cancer patients, age ≥18 years, cisplatin≥50mg/m^2^. \| Granisetron（98） Granisetron+Dexamethasone（194） \| ② \| \| Mahrous MA 2021 [42] \| 2021 \| RCT \| 115 cancer patients, aged between 20 and 60 years, cisplatin ≥50mg/m^2^ or AC/EC. \| Palonosetron+Dexamethasone (51） Granisetron+Dexamethasone (64） \| ④⑤ \| \| Mantovani G 1996 [43] \| 1996 \| RCT \| 117 cancer patients, cisplatin≥80 mg/m^2^. \| Granisetron（38） Ondansetron（39）  Tropisetron（40） \| ③ \| \| Martoni A 1996 [44] \| 1996 \| RCT Crossover \| 124 cancer patients, cisplatinum ≥50mg/m^2^. \| Granisetron (66)  Ondansetron (58) \| ①②③ \| \| Marty M 1995 [45] \| 1995 \| Prospective RCT  Multicenter \| 231 cancer patients, cisplatin≥50 mg/m^2^. \| Tropisetron (117) Ondansetron (114) \| ①②④⑤ \| \| Mattiuzzi GN 2010 [46] \| 2010 \| RCT \| 150 cancer patients, >18 years , high-dose cytarabine. \| Ondansetron (47) Palonosetron(dose1) (48) Palonosetron(dose2) (48) \| ①②③④⑤ \| \| Nakamura K 2012 [47] \| 2012 \| RCT Crossover \| 27 breast cancer patients, ≥20 years , FEC100 (high emetic risk) treatment were enrolled. \| Granisetron+Dexamethasone (13) Azasetron+Dexamethasone (14) \| ① \| \| Navari R 1995 [48] \| 1995 \| RCT Multicenter \| 994 cancer patients, aged ≥20 years, cisplatin ≥60mg/m^2^. \| Granisetron(dose1) (328) Granisetron(dose2) (328) Ondansetron (331) \| ①②③ \| \| Noda K 2002 [49] \| 2002 \| RCT Multicenter \| 151 cancer patients, aged ≥20 years, cisplatin ≥50mg/m^2^. \| Ramosetron (75) Ondansetron (76) \| ①② \| \| Öge A 2000 [50] \| 2000 \| RCT \| 106 patients received cisplatin based chemotherapy. \| Granisetron (36) Ondansetron (35)  Tropisetron (35) \| ②⑤ \| \| Olver I 1996 [51] \| 1996 \| RCT Multicenter \| 642 cancer patients, ≥12 years (18 years in France), cisplatin ≥70 mg/m^2^. \| Ondanaetron (214) Ondanaetron +Dexamethasone (66) \| ②③④⑤⑥ \| \| Roila F 1991 [52] \| 1991 \| RCT, Multicent  Crossover \| 102 cancer patients, with cisplatin ≥50mg/m^2^. \| Ondansetron (41)  Ondansetron+Dexamethasone (48) \| ② \| \| Ruff P 1994 [53] \| 1994 \| RCT Multicenter \| 496 cancer patients , ≥18 years, cisplatin ≥50 mg/m^2^. \| Ondansetron（dose1）(165)  Ondansetron（dose2）(162)  Granisetron (169) \| ①②③ \| \| Saito M 2009 [54] \| 2009 \| RCT  Multicenter \| 1114 cancer patients, age ≥20 years, cisplatin ≥50 mg/m^2^ or AC/EC. \| Palonosetron+ Dexamethosone (555) Granisetron + Dexamethosone (559) \| ①②③④⑤⑥ \| \| Sorbe B 1994 [55] \| 1994 \| RCT Multicenter \| Women with gynaecological cancers. 100 mg/m^2^≥cisplatin≥50 mg/m^2^. \| Tropisetron（35） Tropisetron+ Dexamethasone（28） \| ②⑤ \| \| Spector JI 1998 [56] \| 1998 \| RCT Multicenter \| 371 cancer patients, ≥ 12 years, cisplatin 50-75 mg/m^2^. \| Ondansetron (184) Granisetron (187) \| ①②③ \| \| Tan J 2017 [57] \| 2017 \| Prospective RCT \| 555 cancer patients, <18 years scheduled for HEC. \| Palonosetron(dose1)+Dexamethasone (181) Palonosetron(dose2)+Dexamethasone (185) Ondansetron+Dexamethasone（189） \| ①②③④⑤⑥ \| \| Villalon A 2004 [58] \| 2004 \| RCT  Multicenter \| 283 cancer patients, 18-75 years of age, cisplatin ≥50mg/m^2^. \| Ramosetron+Dexamethasone（149） Ramosetron（134） \| ①②③④⑤ \| \| Yu ZC 2009 [59] \| 2009 \| RCT Multicenter \| 208 cancer patients，age ≥18 and ≤70 years, epirubicin 60 mg/m^2^ or cisplatin 75 mg/m^2^. \| Granisetron (104) Palonosetron (104) \| ②⑤ \| |
| Risk of bias in studies | 18 | Present assessments of risk of bias for each included study. | Figure2 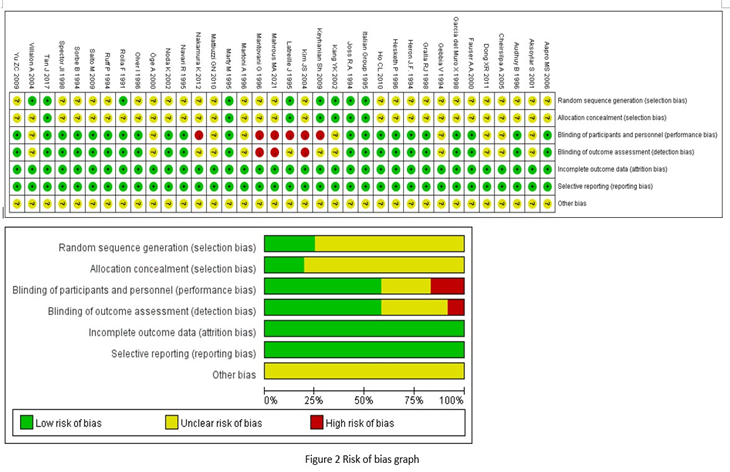 |
| Results of individual studies | 19 | For all outcomes, present, for each study: (a) summary statistics for each group (where appropriate) and (b) an effect estimate and its precision (e.g. confidence/credible interval), ideally using structured tables or plots. | 234-363  The rate of acute nausea  A total of 23 RCTs and 8211 patients were included in “the rate of acute nausea” analysis (Figure 3). The heterogeneity within the network was low (I^2^ = 0% [0.0%; 48.9%])（Supplement 2）, and there was no statistically significant inconsistency in this indicator (p = 0.0896 > 0.05) （Supplement 3, Supplement 4).  Significant differences were showed in acute nausea between T/T+D (RR 1.85, 95 %CI 1.26-2.74), G/G+D (RR1.33, 95%CI 1.10-1.61), O/O+D (RR1.33, 95%CI 1.07-1.66), R/R+D (RR1.54, 95%CI 1.14-2.07) in direct comparisons. In indirect comparisons, there were still significant differences between 5-HT₃ antagonists vs different 5-HT₃ antagonists + Dexamethasone , such as Granisetron vs Ondansetron + Dexamethasone (RR1.32, 95%CI 1.07-1.63), etc. However, no statistically significant difference was observed between Palonosetron and Palonosetron + Dexamethasone (RR1.03, 95%CI 0.59-1.79). No evidence indicated a statistically significant difference between Palonosetron monotherapy and 1^st^ 5-HT₃ antagonists in the management of acute nausea. No significant difference was observed between either Palonosetron or Palonosetron + Dexamethasone and 1^st^ 5-HT₃ antagonists combined with Dexamethasone (Table 3, Supplement 5).  It was most likely that Ramosetron + Dexamethasone would be the most effective treatment for acute nausea (p-score 0.9473), followed by T+D (p-score 0.7827), P+D (p-score 0.6933), G+D (p-score 0.6443), O+D (p-score 0.6287), P (p-score 0.6031), and 1^st^ 5-HT₃ antagonists (Table 4).  The rate of acute vomiting  32 RCTs reported acute vomiting statistics（Figure 3）, and 10659 patients were included. Among the studies in this network, heterogeneity was low (I^2^ = 18% [0.0%; 47.2%])（Supplement 2）, and there was no statistically significant inconsistency in this indicator (p = 0.5130 > 0.05) （Supplement 3, Supplement 4).  Significant differences were noted in acute vomiting between G/G+D (RR1.57, 95%CI 1.32-1.86), O/O+D (RR1.41, 95%CI 1.14-1.74), R/R+D (RR1.40, 95%CI 1.00-1.97), T/T+D (RR1.99, 95%CI 1.43-2.78). Additional significant differences were noted in indirect regimen comparisons, including G+D/R (RR0.75, 95%CI 0.57-0.99)，G+D/O (RR0.69, 95%CI 0.57-0.83), O+D/T (RR0.56, 95%CI 0.41-0.77). No significant superiority of Palonosetron + Dexamethasone over Palonosetron alone was observed (RR1.10, 95%CI 0.64-1.90).  In indirect comparisons, Palonosetron monotherapy exhibited significant advantages compared to Granisetron (RR1.69, 95% CI 1.03-2.77) and Tropisetron (RR0.51, 95% CI 0.29-0.87).  While a significant difference was observed between Ondansetron + Dexamethasone and Palonosetron +Dexamethasone (P+D) (RR1.23, 95% CI 1.04-1.44), no statistically significant differences were found between P+D and other 1^st^ 5-HT₃ antagonists combined with Dexamethasone. No evidence was found to suggest that the effects of Palonosetron are significantly different from those of first-generation 5-HT_3_ receptor antagonists combined with Dexamethasone (Table 5, Supplement 6).  Palonosetron + Dexamethasone achieved the highest efficacy for acute vomiting control (p-score 0.8960), followed by T+D (p-score 0.7625), P (p-score 0.7447), R+D (p-score 0.7435), G+D (p-score 0.6792), O+D (p-score 0.6304), and 1^st^ 5-HT3 antagonists (Table 8) . The p-scores of P+D, T+D, P, and R+D were numerically similar (Table 4).  The rate of acute complete control  In the analysis of “the rate of acute complete control”, 18 RCTs were included（Figure 3), and 7399 patients were involved. The heterogeneity was low (I^2^ = 0% [0.0%; 52.3%])（Supplement 2）, and there was no statistically significant inconsistency in this indicator (p = 0.6249 > 0.05) （Supplement 3, Supplement 4).  There was statistical significance between R/R+D (RR0.79, 95%CI 0.65-0.96), T/T+D (RR0.65, 95%CI 0.53-0.80), G+D/R (RR1.33, 95%CI 1.01-1.74), and so on, but not between Palonosetron and Palonosetron + Dexamethasone（RR0.90, 95%CI 0.75-1.07）. Nevertheless, no statistically significant differences in therapeutic outcomes were observed between either Palonosetron monotherapy or the Palonosetron + Dexamethasone regimen and 1^st^ 5-HT₃ antagonists administered concomitantly with Dexamethasone (Table 6, Supplement 7).  Tropisetron + Dexamethasone seemed to be the most effective treatment for acute complete control (p-score 0.9110), followed by P+D (p-score 0.8426), G+D (p-score 0.6900), O+D (p-score 0.6537), R+D (p-score 0.5415), P (p-score 0.5029), and 1^st^ 5-HT3 antagonists. The p-scores for T+D and P+D were numerically similar. Likewise, the p-scores of R+D and P demonstrated comparable values (Table 4).  The rate of delayed nausea  16 RCTs were included in this analysis (Figure 3）, and 5317 patients were included. The analyzed studies showed moderate heterogeneity (I^2^ = 65.5% [26.5%; 83.8%])（Supplement 2）, and there was no statistically significant inconsistency in this indicator (p = 0.4987 > 0.05) （Supplement 3, Supplement 4).  Regarding delayed nausea control, statistical significance was identified in multiple comparative analyses: R/R+D (RR1.53, 95%CI 1.05-2.24), T/T+D (RR1.49, 95%CI 1.00-2.22), Do/Do+D (RR1.99, 95%CI 1.28-3.08), Do+D/O(RR0.46, 95%CI 0.26-0.81), and so on. However, the adjunct administration of Dexamethasone failed to demonstrate a statistically significant improvement in the therapeutic efficacy of Palonosetron（RR1.22, 95%CI 0.76-1.95).  In direct comparative analyses, Palonosetron exhibited statistically superior efficacy relative to Ondansetron (RR1.42, 95% CI 1.04-1.95), not to other first-generation 5-HT_3_ antagonists. Palonosetron + Dexamethasone was significantly more effective than Granisetron + Dexamethasone (RR1.46, 95% CI 1.18-1.82) and Ondansetron + Dexamethasone (RR1.32, 95% CI 1.06-1.64). However, indirect comparisons revealed no significant efficacy differences between Palonosetron + Dexamethasone and either Dolasetron + Dexamethasone, Tropisetron + Dexamethasone, or Ramosetron + Dexamethasone. Similarly, no evidence has been established to indicate a difference in therapeutic efficacy between Palonosetron and 1^st^ 5-HT_3_ antagonists + Dexamethasone (Table 7, Supplement 8).  In delayed nausea, Dolasetron + Dexamethasone was the most likely effective treatment (p-score 0.9149), followed by P+D (p-score 0.8422), T+D (p-score 0.8109), P (p-score 0.6393), O+D (p-score 0.5603), R+D (p-score 0.4533), T (p-score 0.4350), G+D (p-score 0.4156)，and so on. The p-scores of Dolasetron + Dexamethasone, Tropisetron + Dexamethasone, and Palonosetron + Dexamethasone were relatively close. Similarly, the p-score of Palonosetron was comparable to those of 1^st^ 5-HT_3_ antagonists + Dexamethasone (Table 4).  The rate of delayed vomiting  20 RCTs were included in this analysis (Figure 3）, and 5663 patients were evaluated. Heterogeneity within the network was moderate (I^2^ = 49% [5.4%; 72.5%])（Supplement 2）, but there was no statistically significant inconsistency in this indicator (p = 0.6766 > 0.05) （Supplement 3, Supplement 4).  In delayed vomiting, statistical significance was found between 1^st^ 5-HT_3_ antagonists and 1^st^ 5-HT_3_ antagonists + Dexamethasone, such as R/R+D (RR1.63, 95%CI 1.10-2.41), T/T+D (RR1.59, 95%CI 1.10-2.28), Do/Do+D (RR2.33, 95%CI 1.54-3.53), O/T+D (RR 1.62, 95%CI 1.01-2.60), Do+D/R (RR0.35, 95%CI 0.13-0.94), and so on. In contrast, no evidence was found to indicate that the therapeutic efficacy of Palonosetron was significantly inferior to that of its combination with Dexamethasone (RR1.12, 95% CI 0.71-1.75). Palonosetron was significantly more effective than Ondansetron (RR1.47, 95%CI 1.03-2.08), Ramosetron (RR0.44, 95%CI 0.19-1.00), Dolasetron (RR1.86 , 95%CI 1.04-3.34). In direct comparisons, Palonosetron + Dexamethasone (P+D) demonstrated significantly greater efficacy than both Ondansetron + Dexamethasone (RR 1.35, 95% CI 1.14-1.60) and Granisetron + Dexamethasone (RR 1.52, 95% CI 1.22-1.91). However, indirect comparison analyses revealed no statistically significant differences in efficacy between P+D and Do+D, T+D, or R+D. Similarly, no significant efficacy difference was observed between Palonosetron monotherapy and 1^st^ 5-HT_3_ antagonists + Dexamethasone in indirect comparisons (Table 8, Supplement 9).  Dolasetron + Dexamethasone appeared to be the most effective treatment for delayed vomiting (p-score 0.8660), followed by P+D (p-score 0.8598), T+D (p-score 0.8178), P (p-score 0.7585), O+D (p-score 0.5736), R+D (p-score 0.4175), G (p-score 0.4119), G+D (p-score 0.4059), and so forth . The p-scores for Palonosetron + Dexamethasone, Dolasetron + Dexamethasone (Do+D), and Tropisetron + Dexamethasone (T+D) were relatively comparable (Table 4).  The rate of delayed complete control  8 RCTs were included in the analysis of “the rate of delayed complete control”（Figure 3）, and 3400 patients were included. The heterogeneity within the network was low（I^2^ = 37.9% [0.0%; 78.7%])（Supplement 2）, and no statistically significant inconsistency appeared in this indicator (p = 0.2986 > 0.05) (Supplement 3).  In the delayed complete response analysis, statistically significant differences were observed between O/O+D (RR0.61, 95%CI 0.47-0.81), G+D/O(RR1.63, 95%CI 1.18-2.27) in direct comparative assessments. Notably, indirect comparative analyses revealed no statistically significant difference between Palonosetron and Palonosetron + Dexamethasone (RR 0.66, 95%CI 0.41-1.06）. Palonosetron was significantly more effective than Ondansetron (RR0.70, 95%CI 0.49-0.99). P+D demonstrated superior efficacy versus both G+D (RR 0.75, 95% CI 0.63-0.90) and O+D (RR0.75, 95% CI 0.63-0.90) in direct comparisons. But, no significant difference was observed between P+D and R+D in indirect comparisons (RR1.23, 95% CI 0.75-2.01) (Table 9, Supplement 10).  Palonosetron + Dexamethasone showed the most effectiveness in delayed complete control (p-score 0.9215), followed by G (p-score 0.6529), R+D (p-score 0.6053), O+D (p-score 0.4811), G+D (p-score 0.4799), P (p-score 0.3494), O (p-score 0.0100). The p-score values for Palonosetron + Dexamethasone and Ramosetron + Dexamethasone were similar (Table 4). |
| Results of syntheses | 20a | For each synthesis, briefly summarise the characteristics and risk of bias among contributing studies. | Supplement 2, Supplement 3, Supplement 4, Supplement 13  We observed low heterogeneity between studies in this network and no significant inconsistencies among direct and indirect comparisons (Supplement 2, Supplement 3, Supplement 4).  The funnel plot of the studies with 10 or more trials all demonstrated a fundamental symmetry, suggesting the absence of publication bias in the reviewed articles (Supplement 13). |
|  | 20b | Present results of all statistical syntheses conducted. If meta-analysis was done, present for each the summary estimate and its precision (e.g. confidence/credible interval) and measures of statistical heterogeneity. If comparing groups, describe the direction of the effect. | Table 3-9 |
|  | 20c | Present results of all investigations of possible causes of heterogeneity among study results. | Supplement 2  We observed low heterogeneity between studies in this network (Supplement 2). |
|  | 20d | Present results of all sensitivity analyses conducted to assess the robustness of the synthesized results. | 371-375  Supplement 12  Two studies reported children trails (Aksoylar S 2001[25], Tan J 2017[57]), and the pooled estimates were not significantly impacted by the exclusion of data from these studies. A sensitivity analysis was conducted by excluding one study (Roila F 1991 [52]) that was not analyzed according to the intention-to-treat (ITT) principle. This exclusion did not significantly change the results of the meta-analysis (Supplement 12). |
| Reporting biases | 21 | Present assessments of risk of bias due to missing results (arising from reporting biases) for each synthesis assessed. | We did not meet this situation. 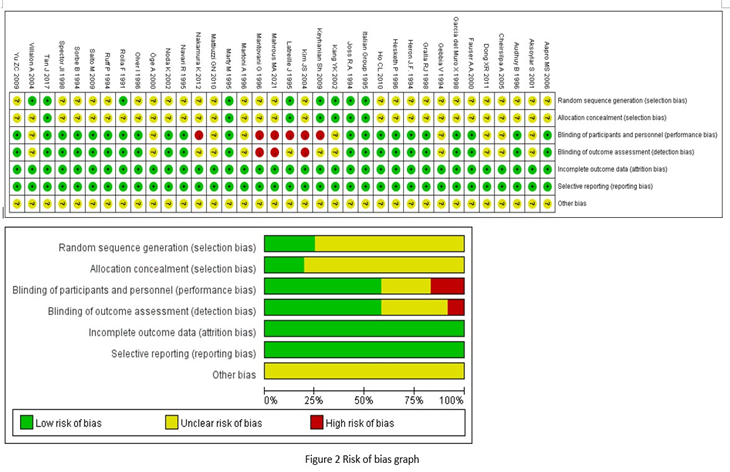 |
| Certainty of evidence | 22 | Present assessments of certainty (or confidence) in the body of evidence for each outcome assessed. | Supplement 3，Supplement 4  no significant inconsistencies among direct and indirect comparisons. |
| **DISCUSSION** | | |  |
| Discussion | 23a | Provide a general interpretation of the results in the context of other evidence. | 389-390, 414-415, 450-455  In our study, we compared the efficacy of 1st 5-HT3 antagonists, Palonosetron (with Dexamethasone or not) of nausea, vomiting, and complete control in HEC. We reached the following conclusions by synthesizing these results.  Palonosetron is generally more effective than first-generation 5-HT3 receptor antagonists, with a significantly greater advantage over Ondansetron in the delayed phase.  Dexamethasone might significantly increase the efficacy of 1st 5-HT3 antagonists in all phases, however, it does not provide a similar benefit for Palonosetron.  Palonosetron + Dexamethasone demonstrates superior efficacy over both Ondansetron + Dexamethasone and Granisetron + Dexamethasone, particularly during the delayed phase. However, no significant differences in efficacy were observed between Tropisetron/Ramosetron plus Dexamethasone and Palonosetron + Dexamethasone, or between first-generation 5-HT3 antagonists combined with Dexamethasone and Palonosetron. |
|  | 23b | Discuss any limitations of the evidence included in the review. | 487-490  However, there were still some limitations in this study. No assessment was conducted regarding heterogeneity in chemotherapy regimens, dosing schedules and a lack of reporting on adverse effects or safety profiles.The number of articles for some medicines（e.g., tropisetron, azasetron）was limited, potentially influencing the outcome analysis. |
|  | 23c | Discuss any limitations of the review processes used. | Only PubMed, Embase, the Cochrane Library, and Web of Science were searched, and only English-language literatures were included. The indirect comparison method employed might not possess sufficient statistical power to accurately compare treatments with each other. |
|  | 23d | Discuss implications of the results for practice, policy, and future research. | 493-506  This network meta-analysis on the prevention of vomiting and nausea in highly emetogenic chemotherapy (HEC), indicates that Dexamethasone should be co-administered with first-generation 5-HT3 antagonists whenever clinically feasible. For patients who are unable to receive Dexamethasone, Palonosetron may be considered the preferred therapeutic option based on its established efficacy advantage over 1st 5-HT3 antagonists during the delayed phase, particularly in comparison with Ondansetron. Overall, the Palonosetron + Dexamethasone was identified as the most effective regimen, demonstrating superior clinical efficacy over both Granisetron + Dexamethasone and Ondansetron + Dexamethasone, especially during the delayed phase. However, due to the limited amount of available evidence, the superiority of Palonosetron + Dexamethasone over Ramosetron + Dexamethasone, and Tropisetron + Dexamethasone has not been conclusively established in these indirect comparisons. No evidence has been established to indicate a difference in therapeutic efficacy between Palonosetron and 1st 5-HT3 antagonists + Dexamethasone in all outcomes. Future rigorously designed trials are needed to corroborate these comparative efficacy outcomes. |
| **OTHER INFORMATION** | | |  |
| Registration and protocol | 24a | Provide registration information for the review, including register name and registration number, or state that the review was not registered. | 114-116  This network meta-analysis was performed in accordance with the Preferred Reporting Items for Systematic Reviews and Meta-Analyses (PRISMA) guidelines [22]. The protocol for this analysis was registered under the reference CRD42023403570. |
|  | 24b | Indicate where the review protocol can be accessed, or state that a protocol was not prepared. | We did not possess a formal review protocol. |
|  | 24c | Describe and explain any amendments to information provided at registration or in the protocol | No amendments. |
| Support | 25 | Describe sources of financial or non-financial support for the review, and the role of the funders or sponsors in the review. | 2021 Clinical Research Funds of Shandong Medical Association-Qilu Specialized Funding  Subject Number：YXH2022ZX02058 |
| Competing interests | 26 | Declare any competing interests of review authors. | The authors declare that they have no conflict of interest. |
| Availability of data, code and other materials | 27 | Report which of the following are publicly available and where they can be found: template data collection forms; data extracted from included studies; data used for all analyses; analytic code; any other materials used in the review. | We have yet to determine the appropriate method for disseminating the materials. |

*From:*  Page MJ, McKenzie JE, Bossuyt PM, Boutron I, Hoffmann TC, Mulrow CD, et al. The PRISMA 2020 statement: an updated guideline for reporting systematic reviews. BMJ 2021;372:n71. doi: 10.1136/bmj.n71
