# Supplementary material for: Comparative efficacy of 5-hydroxytryptamine-3 (5-HT3) receptor antagonists with or without dexamethasone for prevention of chemotherapy-induced nausea and vomiting following highly emetogenic chemotherapy (HEC): a network meta-analysis
Source: PeerJ. 2026 Apr 2;14:e21047. doi: 10.7717/peerj.21047 (PMC13050518; doi:10.7717/peerj.21047)
Supplement: Supplemental Information 2 [file peerj-14-21047-s002.docx]

**Supplement 1 The table comparing the** **covariates across the comparisons**

| Number | Intervention |  |  | age(Mean±SD) |  |  | Sex(Female) |  |  | Tumor type(number) |  |  | Chemotherapy(number) |  |  |
| --- | --- | --- | --- | --- | --- | --- | --- | --- | --- | --- | --- | --- | --- | --- | --- |
|  | Group 1 | Group 2 | Group 3 | Group 1 | Group 2 | Group 3 | Group 1 | Group 2 | Group 3 | Group 1 | Group 2 | Group 3 | Group 1 | Group 2 | Group 3 |
| Aapro MS 2006 [24] | Palonosetron (0.25mg）+ Dexamethasone (20mg，iv, 15min before chemotherapy initiation) | Palonosetron (0.75mg）+ Dexamethasone (20mg, iv, 15min before chemotherapy initiation) | Ondansetron + Dexamethasone (20mg, iv,15min before chemotherapy initiatio ) | 53.4±13.7 | 50.6±14,1 | 50.9±14.2 | 115 | 113 | 113 | Ovarian(38), Lung(35), Hodgkin(23), Gastric(9),Breast(13) | Ovarian(41), Lung(30), Hodgkin(14), Gastric(12),  Breast(6) | Ovarian(39),  Lung(333),  Hodgkin(17),  Gastric(14),  Breast(14) | Cisplatin(184), Cyclophosphamide (57), Dacarbazine(28) | Cisplatin(189), Cyclophosphamide (53), Dacarbazine(24) | Cisplatin(181), Cyclophosphamide (59), Dacarbazine(30) |
| Aksoylar S 2001[25] | Tropisetron | Granisetron | / | 6.9±4.9 | 9.2±4.5 | / | / | / | / | / | / | / | Grade 3（39），Grade 4（27） | Grade 3（45），Grade 4（22） | / |
| Audhuy B 1996 [26] | Dolasetron (1.8mg/kg) | Dolasetron (2.4mg/kg) | Granisetron | 54±11 | 54±13 | 56±12 | 58 | 51 | 50 | / | / | / | Cisplatin dose(mg/m2):  96±12 | Cisplatin dose(mg/m2):  96±11 | Cisplatin dose(mg/m2) :  98±11 |
| Cheirsilpa A 2005 [27] | Ramosetron +Dexamethasone (20mg，iv,15min before chemotherapy initiation) | Granisetron + Dexamethasone (20mg，iv, 15min before chemotherapy initiation) | / | 54.5±10.2 | 54.0±10.5 | / | 18 | 19 |  | Head and neck(16),  Cervix(11),Lung(5), ovary（1），  Stomach（2），Urinary bladder(1) | Head and neck(11), Cervix(155),Lung(2), ovary（2）， Stomach（11），Testis(1), other(5) | / | Cisplatin (25), Cisplatin +5-Fluorouracil (5),  Cisplatin + Etoposide(5), Cisplatin +other（1） | Cisplatin (26), Cisplatin +5-Fluorouracil (8),  Cisplatin + Etoposide (2), Cisplatin +other（1） | / |
| Dong XR 2011 [28] | Palonosetron | Ondansetron | / | / | / | / | 14 | 18 | / | Adnocarcinoma(19),squamous carcinoma(23), others(2) | Adnocarcinoma(21), squamous carcinoma(23), others(1) | / | / | / | / |
| Fauser A.A. 2000 [29] | Dolasetron | Dolasetron + Dexamethasone (20mg，iv, 5min before cisplatin infusion) | / | 46 | 44 | / | 19 | 24 | / | Testicular cancer(48),Lung(19),Head/neck(12),  Gynaecological tumours(6),  gastrointestinal(4),  Breast(1),  Lymphoma(2),  Other(11) | Testicular cancer(51),Lung(15),Head/neck(15),  Gynaecological tumours(4),  gastrointestinal(7),  Breast(4),  Lymphoma(1),  Other(10) | / | / | / | / |
| Garcia del Muro X 1998 [30] | Tropisetron | Tropisetron + Dexamethasone(20mg，iv, before cisplatin infusion on day 1) | / | 58 | 57 | / | 24 | 26 | / | Lung(59), Head/neck(40), Ovary(14), Oesophageal(13), other(17) | Lung(57), Head/neck(37), Ovary(19), Oesophageal(10), other(12) | / | Cisplatin dose(mg/m2):  50-75(26),  76-99(21),  ≥100(96) | Cisplatin dose(mg/m2):  50-75(29),  76-99(17),  ≥100(89) | / |
| Gebbia V 1994 [31] | Ondansetron | Granisetron | / | 59.6 | 58.5 | / | 26 | 34 | / | Head/neck(38),  Lung (14),  Urinary bladder(8),  Ovary(8),Stomach(4),  Endomatrium(6), Vulva(3),Breast(1),  Testis(1),Sarcoma(1) | Head/neck(40),  Lung (12),  Urinary bladder(4), Ovary(4),Stomach(6),Endomatrium(4), Vulva(8),Breast(4) | / | Mean Cisplatin dose(mg/m2):84 | Mean Cisplatin dose(mg/m2):83 | / |
| Gralla RJ 1998 | Granisetron | Ondansetron | / | 61.5±11.6 | 61.9±11.3 | / | 188 | 175 | / | / | / | / | / | / | / |
| Heron J.F. 1994 [33] | Granisetron | Granisetron + Dexamethasone (12 mg, iv, 5min before cisplatin infusion) | / | 54.9±11.0 | 54.9±12.3 | / | 71 | 67 | / | Ovary(26),Cervix(27),Head and neck(20),  Lung(10),  Urethra/bladder(11),other(14) | Ovary(30),Cervix(21),Head and neck(16),  Lung(10),  Urethra/bladder(8),  other(11) | / | Cisplatin dose(mg/m2):  80.0±24.6 | Cisplatin dose(mg/m2):  81.9±23.1 | / |
| Hesketh P 1996 [34] | Dolasetron (1.8mg/kg) | Dolasetron (2.4mg/kg) | Ondansetron | 62.0 | 62.0 | 62.0 | 71 | 76 | 85 | Lung(111),  Gastrointestinal(23),  Gynecologic(18),  Head/neck（22），other（24） | Lung(116),  Gastrointestinal(16),  Gynecologic(20),  Head/neck（22），other（31） | Lung(105),  Gastrointestinal(26),  Gynecologic(24),  Head/neck（22），other（29） | / | / | / |
| Ho CL 2010 [35] | Ramosetron +Dexamethasone (20mg，iv, 30min before chemotherapy initiation) | Granisetron + Dexamethasone (20mg，iv, 15min before chemotherapy initiation) | / | 51 | 51 | / | 90 | 85 | / | Breast(63), Lung(48),  Nasopharynx(6),  Mouth(6),Rectum(4),  Liver(2),Bladder(2),  Stomach(2),  Esophagus(2),  Testis(2),Brain(1),  others(1) | Breast(60), Lung(35),  Nasopharynx(10),  Mouth(7),Rectum(4),  Liver(5),Bladder(7),  Stomach(6),  Esophagus(1), Brain(1) | / | Cisplatin(68),  Doxorubicin(41)  ,Epirubicin(25),  Oxaliplatin(10) | Cisplatin(64),  Doxorubicin(46),  Epirubicin(19),  Oxaliplatin(12) | / |
| Italian Group 1995 [36] | Granisetron + Dexamethasone (20mg，iv, 45min before chemotherapy initiation) | Ondansetron +Dexamethasone (20mg，iv, 45min before chemotherapy initiation) | / | 61 | 61 | / | 166 | 143 | / | Ovary(70),Lung(189),Head-neck(57),  Bladder(63),  Other(104) | Ovary(71),Lung(182),Head-neck(55),  Bladder(75),  Other(100) | / | Anthracyclines±others(430),  Cyclophosphamide±others(53) | Anthracyclines±others(430),  Cyclophosphamide±others(53) | / |
| Joss R.A. 1994 [37] | Ondansetron | Ondansetron +Dexamethasone(20mg，iv, 45min before chemotherapy initiation) | / | 53 | 52 | / | 16 | 18 | / | Head-neck(5),  Lung(21), Ovary(5), Testis(11),  other（16） | Head-neck(5),  ,Lung(17), Ovary(12), Testis(5),other（14） | / | No Cisplatin (1),  Cisplatin alnoe(3),  Cisplatin combination(54) | No Cisplatin (1),  Cisplatin alnoe(2),  Cisplatin combination(50) | / |
| Kang YK 2002 [38] | Ramosetron | Granisetron | / | 55.2 | 53.2 | / | 26 | 29 | / | Gastrointestinal(43),  others(51) | Gastrointestinal(35),  others(65) | / | Mean cisplatin dose(mg/m2):72.5 | Mean cisplatin dose(mg/m2):73.8 | / |
| Keyhanian Sh 2009 [39] | Granisetron | Granisetron + Dexamethasone (8mg，iv, 30min before chemotherapy initiation) | / | 64 | 63 | / | 42 | 37 | / | GIT(20),Breast(22), Hematology(21) | GIT(20),Breast(21), Hematology(21) | / | Cisplatin dose(mg/m2):<60(13),>60(50) | Cisplatin dose(mg/m2): <60(14),>60(48) | / |
| Kim JS 2004 [40] | Dolasetron | Ondansetron | / | 53.8±12.5 | 57.4±10.0 | / | 19 | 20 | / | Lung(25), Gastric cancer(10),Others(15) | Lung(28), Gastric cancer(12),Others(15) | / | 5-Fluorouracil +Clisplatin(12), others(38) | 5-Fluorouracil +Clisplatin(16), others(39) | / |
| Latreille J 1995 [41] | Granisetron | Granisetron + Dexamethasone (10mg，iv, 15min before chemotherapy initiation) | / | 60 | 60 | / | 64 | 64 | / | Bladder(6), Bronchus(40), Ovary(36),  Head and neck(2), Other(16) | Bladder(4), Bronchus(39), Ovary(36),  Head and neck(5), Other(16) | / | Cisplatin dose(mg/m2):50-74(23), ≥75(77) | Cisplatin dose(mg/m2):50-74(22), ≥75(78) | / |
| Mahrous MA 2021 [42] | Palonosetron +Dexamethasone(16mg，iv, before chemotherapy initiation) | Granisetron + Dexamethasone(16mg，iv, before chemotherapy initiation) | / | 47.5±9.2 | 49.9±9.0 | / | / | / | / | / | / | / | Adriamycin + Cyclophosphamide (27), Cisplatin(24) | Adriamycin + Cyclophosphamide (38), Cisplatin(26) | / |
| Mantovani G 1996 [43] | Granisetron | Ondansetron | Tropisetron | 58 | 59.3 | 56.4 | / | / | / | / | / | / | / | / | / |
| Martoni A 1996 [44] | Granisetron | Ondansetron | / | / | / | / | / | / | / | / | / | / | / | / | / |
| Marty M 1995 [45] | Tropisetron | Ondansetron | / | / | / | / | 64 | 62 | / | Lung(79).  Gastrointestinal(12),Other(16) | Lung(84).  Gastrointestinal(5),Other(16) | / | / | / | / |
| Mattiuzzi GN 2010 [46] | Ondansetron | Palonosetron (days1-5) | Palonosetron (Days 1,3,5) | 54 | 52 | 53 | 25 | 23 | 20 | Acute myelogenous leukemia(46) | Acute myelogenous leukemia(48) | Acute myelogenous leukemia(46) | Fludarabine+cytarabine(4), Idarubicin+cytarabine(43) | Fludarabine+cytarabine(6), Idarubicin+cytarabine(42) | Fludarabine+cytarabine(6), Idarubicin+cytarabine(42) |
| Nakamura K 2012 [47] | Azasetron + Dexamethasone (no mention dose，iv, before chemotherapy initiation) | Granisetron + Dexamethasone (no mention dose，iv, before chemotherapy initiation) | / | 51 | 50 | / | 14 | 13 | / | / | / | / | / | / | / |
| Navari R 1995 [48] | Granisetron (10ug/kg) | Granisetron (40ug/kg) | Ondansetron | 62.6 | 62.7 | 61.5 | 122 | 118 | 120 | / | / | / | Cisplatin dose(mg/m2):  80.7±17.4 | Cisplatin dose(mg/m2):  80.7±16.6 | Cisplatin dose(mg/m2):  83.1±16.4 |
| Noda K 2002 [49] | Ramosetron | Ondansetron | / | / | / | / | 41 | 34 | / | Lung(24),Ovary(18),  Uterus(11),  Urinary tract/bladder(7),  Stomach (3),  Unknown(2),  Others(2) | Lung(28),Ovary(13),  Uterus(11),  Urinary tract/bladder(6), Stomach (4),  Unknown(1), Others(4),Rectum(2) | / | Cisplatin dose(mg/m2):  47.6-59(14),  60-69(14),  70-79(20),  80-90(15),  90-99(2),  100+(2) | Cisplatin dose(mg/m2):  47.6-59(16),  60-69(9),  70-79(14),  80-90(23),  90-99(2),  100+(5) | / |
| Öge A 2000 [50] | Granisetron | Ondansetron | Tropisetron | / | / | / | / | / | / | / | / | / | / | / | / |
| Olver I 1996 [51] | Ondansetron | Ondansetron +Dexamethasone(20mg，iv, 45min before chemotherapy initiation) | / | 59±11 | 59±12 | / | 152 | 29 | / | Lung(123),  Gynaecological(123), Head and neck(74), Gastrointestinal(62),  Genito-urinary(51), Others(32) | Lung(16),  Gynaecological(18) . Head and neck(13), Gastrointestinal(11),  Genito-urinary(8), Others(6) | / | Cisplatin alone(29/214), Cisplatin with 5-fluororacil(55/214), Cisplatin with cyclophosphamide(38/214) | Cisplatin alone(9), Cisplatin with 5-fluororacil(20), Cisplatin with cyclophosphamide(12) | / |
| Roila F 1991 [52] | Ondansetron | Ondansetron +Dexamethasone (20mg，iv, 45min before chemotherapy initiation) | / | 60.8 | 59,2 | / | 22 | 22 | / | Genitourinary(22), Lung(13), Head and neck(7),Other(6) | Genitourinary(23), Lung(11), Head and neck(10),Other(9) | / | Cisplatin alone(13),  Cisplatin + Methotrexate + Adriamycin + Vinblastine (10),  Cisplatin + Cyclophosphamide (6),  Cisplatin + Etoposide (8),  Cisplatin + Adriamycin + Cyclophosphamide (3), Cisplatin +other(8) | Cisplatin alone(12), Cisplatin + Methotrexate + Adriamycin + Vinblastine (7),  Cisplatin + Cyclophosphamide (6),  Cisplatin + Etoposide (9),  Cisplatin + Adriamycin + Cyclophosphamide (7), Cisplatin +other(13) | / |
| Ruff P 1994 [53] | Ondansetron (8mg) | Ondansetron (32mg) | Granisetron | 55 | 54 | 55 | 72 | 74 | 71 | / | / | / | Cisplatin dose(mg/m2):  <50(25),  50-70(45),  70-100(72),  ≥100(23) | Cisplatin dose(mg/m2):  <50(21),  50-70(56),  70-100(65),  ≥100(20) | Cisplatin dose(mg/m2):  <50(22),  50-70(54),  70-100(65),  ≥100(28) |
| Saito M 2009 [54] | Palonosetron +Dexamethasone(16mg，iv, 45min before chemotherapy initiation) | Granisetron + Dexamethasone(16mg，iv, 45min before chemotherapy initiation) | / | / | / | / | 326 | 324 | / | / | / | / | Cisplatin(316),  AC/EC(239) | Cisplatin(323),  AC/EC(236) | / |
| Sorbe B 1994 [55] | Tropisetron | Tropisetron + Dexamethasone(20mg，iv, at the end of prehydration on day 1) | / | 59 | 58 | / | / | / | / | Ovarian(21),  Endometrial/cervical cancer(11),  Other(3) | Ovarian(20),  Endometrial/cervical cancer(8) | / | Cisplatin dose(mg/m2):60 | Cisplatin dose(mg/m2):60 | / |
| Spector JI 1998 [56] | Ondansetron | Granisetron | / | 63,8 | 64.3 | / | 79 | 86 | / | Lung(111),  Gynecological(18)  ,Genitourinary(17),  Gastrointestinal(15),  Head/neck(15),  Other(8) | Lung(109),  Gynecological(19),  Genitourinary(16),  Gastrointestinal(15),  Head/neck(11),  Other(17) | / | Cisplatin dose(mg/m2):  65.5±8.5 | Cisplatin dose(mg/m2):  65.2±9.5 | / |
| Tan J 2017 [57] | Palonosetron (5ug/kg)+ Dexamethasone (5mg/m2，iv, 30min before chemotherapy initiation ) | Palonosetron (10ug/kg)+ Dexamethasone (5mg/m2，iv, 30min before chemotherapy initiation ) | Ondansetron+ Dexamethasone (5mg/m2，iv, 30min before chemotherapy initiation ) | 5.07±4.13 | 4.97±4.38 | 5.11±4.81 | 93 | 95 | 100 | Neuroblasotoma(41),Non-Hodgkin Lymphoma(33),  Hodgkin disease(21),  Hepatoblastoma(19),Rhabdomyosarcoma(15),  Ewing(10),  Primitive neuroectodermaltumor(8),  Osteosarcoma(8),  Other(26) | Neuroblasotoma(45),Non-Hodgkin Lymphoma(32),Hodgkin disease(200),  Hepatoblastoma(20), Rhabdomyosarcoma(16),  Ewing(9),  Primitive neuroectodermaltumor(8),  Osteosarcoma(7),  Other(28) | Neuroblasotoma(48),Non-Hodgkin Lymphoma(35),Hodgkin disease(18),  Hepatoblastoma(16), Rhabdomyosarcoma(17),  Ewing(8),  Primitive neuroectodermaltumor(6),  Osteosarcoma(6),  Other(35) | Cisplatin(110),  Cyclophosphamide(70),ACregimen(20) | Cisplatin(116),  Cyclophosphamide(74),ACregimen(19) | Cisplatin(108),  Cyclophosphamide(75),ACregimen(22) |
| Villalon A 2004 [58] | Ramosetron +Dexamethasone(20mg，iv, 30min before chemotherapy initiation) | Ramosetron |  | 50.3 | 48 | / | 44 | 43 | / | Head and neck(40),  Lung(30),Ovary(8),  Others(22) | Head and neck(411),  Lung(30),Ovary(6),  Others(23) | / | Cisplatin(14), Cisplatin+other(86) | Cisplatin(155), Cisplatin+other(85) | / |
| Yu ZC 2009 [59] | Granisetron | Palonosetron |  | 50.3±9,5 | 52.±11.0 | / | 38 | 38 | / | Non-small lung cancer(18),  Gastric cancer(11),  Breast(9),  Esophageal(6), Hepatocellular(7),  Head and neck(35),  Miscellaneous(18) | Non-small lung cancer(12),  Gastric cancer(10),  Breast(6),  Esophageal(11), Hepatocellular(4),  Head and neck(31),  Miscellaneous(30) | / | Cisplatin contained (87),  Epirubicin contained(17) | Cisplatin contained (92),  Epirubicin contained(12) | / |
