# Supplementary material for: Comparative efficacy of 5-hydroxytryptamine-3 (5-HT3) receptor antagonists with or without dexamethasone for prevention of chemotherapy-induced nausea and vomiting following highly emetogenic chemotherapy (HEC): a network meta-analysis
Source: PeerJ. 2026 Apr 2;14:e21047. doi: 10.7717/peerj.21047 (PMC13050518; doi:10.7717/peerj.21047)
Supplement: Supplemental Information 3 [file peerj-14-21047-s003.docx]

Supplement 2 Risk of bias rating of studies

| Number | Study | Global risk of bias rating |
| --- | --- | --- |
| 1 | Aapro MS 2006 | Low risk |
| 2 | Aksoylar S 2001 | Moderate risk |
| 3 | Audhuy B 1996 | Low risk |
| 4 | Cheirsilpa A 2005 | Moderate risk |
| 5 | Dong XR 2011 | Moderate risk |
| 6 | Fauser A.A. 2000 | Low risk |
| 7 | Garcia del Muro X 1998 | Low risk |
| 8 | Gebbia V 1994 | Moderate risk |
| 9 | Gralla RJ 1998 | Low risk |
| 10 | Heron J.F. 1994 | Low risk |
| 11 | Hesketh P 1996 | Low risk |
| 12 | Ho CL 2010 | Low risk |
| 13 | Italian Group 1995 | Low risk |
| 14 | Joss R.A. 1994 | Low risk |
| 15 | Kang YK 2002 | Low risk |
| 16 | Keyhanian Sh 2009 | Moderate risk |
| 17 | Kim JS 2004 | High risk |
| 18 | Latreille J 1995 | Moderate risk |
| 19 | Mahrous MA 2021 | High risk |
| 20 | Mantovani G 1996 | High risk |
| 21 | Martoni A 1996 | Moderate risk |
| 22 | Marty M 1995 | Low risk |
| 23 | Mattiuzzi GN 2010 | Moderate risk |
| 24 | Nakamura K 2012 | Moderate risk |
| 25 | Navari R 1995 | Low risk |
| 26 | Noda K 2002 | Low risk |
| 27 | Öge A 2000 | Moderate risk |
| 28 | Olver I 1996 | Low risk |
| 29 | Roila F 1991 | Low risk |
| 30 | Ruff P 1994 | Low risk |
| 31 | Saito M 2009 | Low risk |
| 32 | Sorbe B 1994 | Low risk |
| 33 | Spector JI 1998 | Low risk |
| 34 | Tan J 2017 | Low risk |
| 35 | Villalon A 2004 | Moderate risk |
| 36 | Yu ZC 2009 | Low risk |
