# Supplementary material for: Comparative efficacy of 5-hydroxytryptamine-3 (5-HT3) receptor antagonists with or without dexamethasone for prevention of chemotherapy-induced nausea and vomiting following highly emetogenic chemotherapy (HEC): a network meta-analysis
Source: PeerJ. 2026 Apr 2;14:e21047. doi: 10.7717/peerj.21047 (PMC13050518; doi:10.7717/peerj.21047)
Supplement: Supplemental Information 4 [file peerj-14-21047-s004.docx]

Supplement 3 Sensitivity analyses

Sensitivity analysis of the exclusion of data from three studies with a high risk of bias

The results for outcomes were largely unchanged, except in cases where the network connectivity was broken, making comparison impossible.

Acute nausea

| A+D | . | . | 1.16 (0.67; 2.00) | . | . | . | . | . | . | . | . |
| --- | --- | --- | --- | --- | --- | --- | --- | --- | --- | --- | --- |
| 0.89 (0.49; 1.62) | Do | 0.98 (0.85; 1.13) | . | . | . | . | . | . | . | . | . |
| 0.87 (0.49; 1.56) | 0.98 (0.85; 1.13) | G | 1.32 (1.06; 1.63) | 1.00 (0.93; 1.08) | . | . | . | 1.32 (0.96; 1.82) | . | 0.41 (0.23; 0.73) | . |
| 1.16 (0.67; 2.00) | 1.30 (1.02; 1.65) | 1.33 (1.10; 1.61) | G+D | . | 1.04 (0.91; 1.19) | . | 0.97 (0.83; 1.13) | . | 1.22 (0.66; 2.23) | . | . |
| 0.87 (0.48; 1.55) | 0.97 (0.83; 1.14) | 0.99 (0.92; 1.07) | 0.75 (0.61; 0.91) | O | 1.26 (0.74; 2.13) | 1.33 (0.80; 2.21) | . | 1.16 (0.72; 1.86) | . | 1.10 (0.78; 1.56) | . |
| 1.15 (0.66; 2.01) | 1.29 (1.00; 1.67) | 1.32 (1.06; 1.64) | 0.99 (0.88; 1.12) | 1.33 (1.06; 1.67) | O+D | . | 1.14 (0.93; 1.39) | . | . | . | . |
| 1.15 (0.53; 2.49) | 1.29 (0.76; 2.20) | 1.32 (0.79; 2.20) | 0.99 (0.58; 1.71) | 1.33 (0.80; 2.21) | 1.00 (0.58; 1.74) | P | . | . | . | . | . |
| 1.19 (0.68; 2.09) | 1.34 (1.02; 1.75) | 1.36 (1.09; 1.72) | 1.03 (0.90; 1.17) | 1.37 (1.08; 1.74) | 1.03 (0.90; 1.19) | 1.03 (0.59; 1.80) | P+D | . | . | . | . |
| 1.07 (0.58; 2.00) | 1.20 (0.90; 1.60) | 1.23 (0.96; 1.58) | 0.92 (0.68; 1.25) | 1.24 (0.96; 1.59) | 0.93 (0.68; 1.28) | 0.93 (0.53; 1.64) | 0.90 (0.65; 1.24) | R | 1.61 (1.16; 2.25) | . | . |
| 1.65 (0.85; 3.19) | 1.85 (1.26; 2.71) | 1.89 (1.33; 2.69) | 1.42 (0.98; 2.06) | 1.90 (1.33; 2.72) | 1.43 (0.97; 2.11) | 1.43 (0.77; 2.66) | 1.38 (0.94; 2.05) | 1.54 (1.14; 2.08) | R+D | . | . |
| 0.74 (0.38; 1.41) | 0.83 (0.59; 1.15) | 0.84 (0.62; 1.14) | 0.63 (0.44; 0.91) | 0.85 (0.63; 1.15) | 0.64 (0.44; 0.93) | 0.64 (0.35; 1.15) | 0.62 (0.42; 0.90) | 0.69 (0.47; 1.02) | 0.45 (0.28; 0.71) | T | 1.85 (1.25; 2.75) |
| 1.37 (0.64; 2.93) | 1.53 (0.91; 2.57) | 1.56 (0.95; 2.57) | 1.18 (0.69; 2.00) | 1.58 (0.96; 2.59) | 1.19 (0.69; 2.04) | 1.18 (0.58; 2.40) | 1.15 (0.66; 1.98) | 1.27 (0.73; 2.22) | 0.83 (0.45; 1.52) | 1.85 (1.25; 2.75) | T+D |

P-score

R+D 0.9462

T+D 0.7785

P+D 0.6975

G+D 0.6443

O+D 0.6265

P 0.6022

R 0.5306

A+D 0.4324

Do 0.2608

G 0.2177

O 0.1984

T 0.0648

Acute vomiting

| Do | 0.96 (0.80; 1.15) | . | 0.97 (0.83; 1.13) | . | . | . | . | . | . | . |
| --- | --- | --- | --- | --- | --- | --- | --- | --- | --- | --- |
| 0.92 (0.81; 1.05) | G | 1.57 (1.29; 1.91) | 1.09 (0.99; 1.19) | . | 1.61 (0.94; 2.76) | . | 1.32 (0.94; 1.86) | . | 0.91 (0.65; 1.27) | . |
| 1.44 (1.17; 1.78) | 1.57 (1.32; 1.86) | G+D | . | 1.06 (0.88; 1.28) | . | 1.05 (0.81; 1.35) | . | 0.84 (0.48; 1.46) | . | . |
| 0.99 (0.88; 1.13) | 1.08 (0.99; 1.17) | 0.69 (0.57; 0.83) | O | 1.28 (0.88; 1.85) | 1.98 (0.60; 6.52) | . | 1.06 (0.74; 1.52) | . | 0.78 (0.60; 1.03) | . |
| 1.40 (1.10; 1.78) | 1.52 (1.23; 1.87) | 0.97 (0.84; 1.13) | 1.41 (1.14; 1.74) | O+D | . | 1.32 (1.09; 1.60) | . | . | . | . |
| 1.56 (0.94; 2.58) | 1.69 (1.03; 2.77) | 1.08 (0.64; 1.82) | 1.57 (0.95; 2.57) | 1.11 (0.65; 1.90) | P | . | . | . | . | . |
| 1.72 (1.31; 2.24) | 1.86 (1.47; 2.37) | 1.19 (0.99; 1.43) | 1.73 (1.35; 2.20) | 1.23 (1.04; 1.44) | 1.10 (0.64; 1.90) | P+D | . | . | . | . |
| 1.08 (0.83; 1.41) | 1.18 (0.93; 1.49) | 0.75 (0.57; 0.99) | 1.09 (0.86; 1.39) | 0.78 (0.57; 1.05) | 0.70 (0.40; 1.20) | 0.63 (0.46; 0.87) | R | 1.58 (1.06; 2.37) | . | . |
| 1.52 (1.03; 2.24) | 1.65 (1.14; 2.39) | 1.06 (0.73; 1.53) | 1.53 (1.06; 2.22) | 1.09 (0.73; 1.62) | 0.98 (0.53; 1.81) | 0.89 (0.59; 1.34) | 1.40 (1.00; 1.97) | R+D | . | . |
| 0.79 (0.61; 1.02) | 0.85 (0.67; 1.08) | 0.54 (0.41; 0.73) | 0.79 (0.63; 1.00) | 0.56 (0.41; 0.77) | 0.50 (0.29; 0.87) | 0.46 (0.33; 0.64) | 0.72 (0.52; 1.01) | 0.52 (0.33; 0.80) | T | 1.99 (1.42; 2.78) |
| 1.56 (1.02; 2.39) | 1.70 (1.13; 2.56) | 1.08 (0.70; 1.69) | 1.57 (1.05; 2.37) | 1.12 (0.71; 1.77) | 1.00 (0.53; 1.91) | 0.91 (0.57; 1.46) | 1.44 (0.90; 2.31) | 1.03 (0.59; 1.78) | 1.99 (1.42; 2.78) | T+D |

P-score

P+D 0.8960

T+D 0.7616

P 0.7447

R+D 0.7430

G+D 0.6797

O+D 0.6302

R 0.3635

Do 0.2757

O 0.2709

G 0.1155

T 0.0190

Delayed complete control

| G | 1.12 (0.75; 1.67) | . | . | . | . | . |
| --- | --- | --- | --- | --- | --- | --- |
| 1.12 (0.75; 1.67) | G+D | . | 0.94 (0.75; 1.17) | . | 0.80 (0.65; 0.99) | 0.92 (0.58; 1.47) |
| 1.82 (1.09; 3.06) | 1.63 (1.18; 2.27) | O | 0.61 (0.47; 0.81) | 0.70 (0.49; 0.99) | . | . |
| 1.12 (0.72; 1.73) | 1.00 (0.84; 1.20) | 0.61 (0.47; 0.81) | O+D | . | 0.70 (0.56; 0.88) | . |
| 1.27 (0.68; 2.37) | 1.14 (0.71; 1.84) | 0.70 (0.49; 0.99) | 1.14 (0.73; 1.77) | P | . | . |
| 0.84 (0.54; 1.30) | 0.75 (0.63; 0.90) | 0.46 (0.33; 0.64) | 0.75 (0.63; 0.90) | 0.66 (0.41; 1.06) | P+D | . |
| 1.03 (0.56; 1.90) | 0.92 (0.58; 1.47) | 0.57 (0.32; 1.00) | 0.92 (0.56; 1.51) | 0.81 (0.42; 1.58) | 1.23 (0.75; 2.01) | R+D |

P-score

P+D 0.9215

G 0.6529

R+D 0.6053

O+D 0.4811

G+D 0.4799

P 0.3494

O 0.0100

Sensitivity analysis of the exclusion of data from children trials

Network calculation of the rate of acute nausea

| **A+D** | **.** | **.** | **1.16 (0.67; 2.00)** | **.** | **.** | **.** | **.** | **.** | **.** | **.** | **.** |
| --- | --- | --- | --- | --- | --- | --- | --- | --- | --- | --- | --- |
| **0.90 (0.50; 1.61)** | **Do** | **0.98 (0.85; 1.12)** | **.** | **0.99 (0.68; 1.42)** | **.** | **.** | **.** | **.** | **.** | **.** | **.** |
| **0.88 (0.49; 1.56)** | **0.98 (0.86; 1.11)** | **G** | **1.32 (1.07; 1.63)** | **1.00 (0.93; 1.07)** | **.** | **.** | **.** | **1.32 (0.97; 1.81)** | **.** | **.** | **.** |
| **1.16 (0.67; 2.00)** | **1.30 (1.03; 1.63)** | **1.32 (1.09; 1.60)** | **G+D** | **.** | **1.04 (0.91; 1.19)** | **.** | **0.97 (0.84; 1.12)** | **.** | **1.22 (0.66; 2.23)** | **.** | **.** |
| **0.88 (0.49; 1.57)** | **0.98 (0.85; 1.13)** | **1.00 (0.94; 1.07)** | **0.76 (0.62; 0.92)** | **O** | **1.26 (0.74; 2.12)** | **1.33 (0.80; 2.20)** | **.** | **1.16 (0.72; 1.85)** | **.** | **1.10 (0.78; 1.56)** | **.** |
| **1.20 (0.69; 2.10)** | **1.34 (1.04; 1.73)** | **1.37 (1.10; 1.71)** | **1.04 (0.91; 1.18)** | **1.37 (1.09; 1.71)** | **O+D** | **.** | **.** | **.** | **.** | **.** | **.** |
| **1.17 (0.54; 2.52)** | **1.31 (0.78; 2.21)** | **1.34 (0.80; 2.22)** | **1.01 (0.59; 1.73)** | **1.33 (0.80; 2.20)** | **0.97 (0.56; 1.69)** | **P** | **.** | **.** | **.** | **.** | **.** |
| **1.13 (0.64; 1.98)** | **1.26 (0.96; 1.65)** | **1.29 (1.01; 1.63)** | **0.97 (0.84; 1.12)** | **1.28 (1.00; 1.64)** | **0.94 (0.78; 1.13)** | **0.96 (0.55; 1.68)** | **P+D** | **.** | **.** | **.** | **.** |
| **1.08 (0.58; 2.00)** | **1.21 (0.91; 1.59)** | **1.23 (0.96; 1.58)** | **0.93 (0.69; 1.25)** | **1.23 (0.96; 1.58)** | **0.90 (0.65; 1.23)** | **0.92 (0.53; 1.62)** | **0.96 (0.69; 1.33)** | **R** | **1.61 (1.16; 2.24)** | **.** | **.** |
| **1.66 (0.86; 3.20)** | **1.85 (1.28; 2.69)** | **1.89 (1.33; 2.69)** | **1.43 (0.99; 2.07)** | **1.89 (1.32; 2.69)** | **1.38 (0.94; 2.03)** | **1.42 (0.77; 2.62)** | **1.47 (0.99; 2.18)** | **1.54 (1.14; 2.07)** | **R+D** | **.** | **.** |
| **0.97 (0.50; 1.90)** | **1.08 (0.75; 1.57)** | **1.11 (0.78; 1.57)** | **0.84 (0.56; 1.25)** | **1.10 (0.78; 1.56)** | **0.81 (0.53; 1.22)** | **0.83 (0.45; 1.53)** | **0.86 (0.56; 1.31)** | **0.90 (0.59; 1.38)** | **0.58 (0.36; 0.96)** | **T** | **1.85 (1.26; 2.74)** |
| **1.80 (0.83; 3.92)** | **2.01 (1.17; 3.45)** | **2.05 (1.22; 3.47)** | **1.55 (0.89; 2.71)** | **2.05 (1.22; 3.44)** | **1.50 (0.85; 2.64)** | **1.54 (0.74; 3.17)** | **1.60 (0.90; 2.84)** | **1.67 (0.94; 2.97)** | **1.08 (0.58; 2.03)** | **1.85 (1.26; 2.74)** | **T+D** |

Network calculation of the rate of acute vomiting

| **Do** | **0.96 (0.80; 1.15)** | **.** | **0.98 (0.85; 1.14)** | **.** | **.** | **.** | **.** | **.** | **.** | **.** |
| --- | --- | --- | --- | --- | --- | --- | --- | --- | --- | --- |
| **0.93 (0.82; 1.05)** | **G** | **1.57 (1.29; 1.90)** | **1.09 (0.99; 1.19)** | **.** | **1.61 (0.94; 2.76)** | **.** | **1.32 (0.94; 1.86)** | **.** | **1.06 (0.73; 1.53)** | **.** |
| **1.46 (1.18; 1.80)** | **1.57 (1.33; 1.87)** | **G+D** | **.** | **1.06 (0.89; 1.27)** | **.** | **1.05 (0.81; 1.35)** | **.** | **0.84 (0.48; 1.46)** | **.** | **.** |
| **1.01 (0.89; 1.13)** | **1.09 (1.00; 1.18)** | **0.69 (0.58; 0.83)** | **O** | **1.27 (0.88; 1.84)** | **1.98 (0.60; 6.52)** | **.** | **1.06 (0.74; 1.51)** | **.** | **0.78 (0.60; 1.02)** | **.** |
| **1.39 (1.10; 1.76)** | **1.50 (1.22; 1.85)** | **0.95 (0.82; 1.11)** | **1.38 (1.12; 1.71)** | **O+D** | **.** | **1.53 (1.19; 1.96)** | **.** | **.** | **.** | **.** |
| **1.57 (0.95; 2.60)** | **1.69 (1.04; 2.76)** | **1.08 (0.64; 1.81)** | **1.56 (0.95; 2.55)** | **1.13 (0.66; 1.92)** | **P** | **.** | **.** | **.** | **.** | **.** |
| **1.81 (1.37; 2.38)** | **1.95 (1.52; 2.50)** | **1.24 (1.02; 1.50)** | **1.79 (1.39; 2.32)** | **1.30 (1.07; 1.57)** | **1.15 (0.66; 2.00)** | **P+D** | **.** | **.** | **.** | **.** |
| **1.10 (0.85; 1.42)** | **1.18 (0.93; 1.50)** | **0.75 (0.57; 0.99)** | **1.09 (0.86; 1.38)** | **0.79 (0.58; 1.06)** | **0.70 (0.41; 1.20)** | **0.61 (0.44; 0.85)** | **R** | **1.58 (1.06; 2.37)** | **.** | **.** |
| **1.54 (1.05; 2.26)** | **1.66 (1.15; 2.40)** | **1.06 (0.73; 1.53)** | **1.53 (1.06; 2.21)** | **1.11 (0.74; 1.64)** | **0.98 (0.53; 1.81)** | **0.85 (0.56; 1.29)** | **1.41 (1.00; 1.97)** | **R+D** | **.** | **.** |
| **0.84 (0.64; 1.10)** | **0.91 (0.71; 1.16)** | **0.58 (0.43; 0.78)** | **0.84 (0.66; 1.07)** | **0.60 (0.44; 0.83)** | **0.54 (0.31; 0.93)** | **0.47 (0.33; 0.66)** | **0.77 (0.55; 1.07)** | **0.55 (0.35; 0.85)** | **T** | **1.99 (1.43; 2.78)** |
| **1.68 (1.09; 2.57)** | **1.81 (1.20; 2.74)** | **1.15 (0.74; 1.80)** | **1.66 (1.10; 2.51)** | **1.20 (0.76; 1.91)** | **1.07 (0.56; 2.03)** | **0.93 (0.57; 1.50)** | **1.53 (0.95; 2.45)** | **1.09 (0.63; 1.89)** | **1.99 (1.43; 2.78)** | **T+D** |

Network calculation of the rate of acute complete control

| **G** | **0.80 (0.61; 1.04)** | **0.96 (0.89; 1.03)** | **.** | **.** | **.** | **.** | **.** | **1.16 (0.92; 1.47)** | **.** |
| --- | --- | --- | --- | --- | --- | --- | --- | --- | --- |
| **0.87 (0.74; 1.02)** | **G+D** | **.** | **0.99 (0.92; 1.06)** | **.** | **0.98 (0.91; 1.05)** | **.** | **1.05 (0.87; 1.27)** | **.** | **.** |
| **0.95 (0.88; 1.02)** | **1.09 (0.94; 1.27)** | **O** | **0.94 (0.80; 1.12)** | **0.98 (0.91; 1.05)** | **.** | **.** | **.** | **1.13 (0.89; 1.44)** | **.** |
| **0.87 (0.74; 1.01)** | **1.00 (0.93; 1.07)** | **0.91 (0.79; 1.05)** | **O+D** | **.** | **.** | **.** | **.** | **.** | **.** |
| **0.93 (0.83; 1.03)** | **1.07 (0.90; 1.26)** | **0.98 (0.91; 1.05)** | **1.07 (0.91; 1.26)** | **P** | **.** | **.** | **.** | **.** | **.** |
| **0.85 (0.71; 1.01)** | **0.98 (0.91; 1.05)** | **0.89 (0.76; 1.06)** | **0.98 (0.89; 1.08)** | **0.92 (0.76; 1.10)** | **P+D** | **.** | **.** | **.** | **.** |
| **1.15 (0.84; 1.58)** | **1.33 (1.01; 1.74)** | **1.21 (0.89; 1.66)** | **1.33 (1.01; 1.76)** | **1.24 (0.90; 1.71)** | **1.36 (1.02; 1.80)** | **R** | **0.79 (0.65; 0.96)** | **.** | **.** |
| **0.91 (0.71; 1.17)** | **1.05 (0.87; 1.27)** | **0.96 (0.75; 1.23)** | **1.06 (0.86; 1.29)** | **0.99 (0.76; 1.27)** | **1.07 (0.88; 1.32)** | **0.79 (0.65; 0.96)** | **R+D** | **.** | **.** |
| **1.12 (0.90; 1.39)** | **1.29 (0.99; 1.68)** | **1.18 (0.95; 1.47)** | **1.29 (1.00; 1.68)** | **1.21 (0.96; 1.52)** | **1.32 (1.00; 1.73)** | **0.97 (0.67; 1.42)** | **1.23 (0.88; 1.70)** | **T** | **0.65 (0.53; 0.80)** |
| **0.73 (0.54; 0.98)** | **0.84 (0.60; 1.17)** | **0.77 (0.57; 1.03)** | **0.84 (0.60; 1.17)** | **0.78 (0.58; 1.07)** | **0.86 (0.61; 1.21)** | **0.63 (0.41; 0.97)** | **0.80 (0.54; 1.17)** | **0.65 (0.53; 0.80)** | **T+D** |

Network calculation of the rate of delayed nausea

| **Do** | **1.99 (1.16; 3.41)** | **.** | **.** | **0.91 (0.57; 1.47)** | **.** | **.** | **.** | **.** | **.** | **.** | **.** |
| --- | --- | --- | --- | --- | --- | --- | --- | --- | --- | --- | --- |
| **1.99 (1.16; 3.41)** | **Do+D** | **.** | **.** | **.** | **.** | **.** | **.** | **.** | **.** | **.** | **.** |
| **0.95 (0.48; 1.88)** | **0.48 (0.20; 1.14)** | **G** | **0.97 (0.60; 1.57)** | **1.17 (0.63; 2.20)** | **.** | **.** | **.** | **.** | **.** | **.** | **.** |
| **1.03 (0.52; 2.04)** | **0.52 (0.22; 1.23)** | **1.09 (0.71; 1.66)** | **G+D** | **.** | **1.07 (0.68; 1.70)** | **.** | **1.45 (1.03; 2.05)** | **.** | **1.02 (0.58; 1.78)** | **.** | **.** |
| **0.91 (0.57; 1.47)** | **0.46 (0.22; 0.94)** | **0.96 (0.59; 1.57)** | **0.89 (0.55; 1.44)** | **O** | **1.45 (0.98; 2.17)** | **1.42 (0.98; 2.05)** | **.** | **.** | **.** | **1.21 (0.72; 2.03)** | **.** |
| **1.23 (0.67; 2.24)** | **0.62 (0.28; 1.39)** | **1.30 (0.79; 2.13)** | **1.19 (0.79; 1.79)** | **1.34 (0.93; 1.94)** | **O+D** | **.** | **.** | **.** | **.** | **.** | **.** |
| **1.29 (0.71; 2.37)** | **0.65 (0.29; 1.46)** | **1.37 (0.74; 2.52)** | **1.26 (0.69; 2.30)** | **1.42 (0.98; 2.05)** | **1.05 (0.63; 1.77)** | **P** | **.** | **.** | **.** | **.** | **.** |
| **1.49 (0.70; 3.20)** | **0.75 (0.30; 1.91)** | **1.58 (0.92; 2.72)** | **1.45 (1.03; 2.05)** | **1.64 (0.90; 2.96)** | **1.22 (0.71; 2.08)** | **1.15 (0.57; 2.32)** | **P+D** | **.** | **.** | **.** | **.** |
| **0.68 (0.25; 1.87)** | **0.34 (0.11; 1.08)** | **0.72 (0.31; 1.70)** | **0.66 (0.31; 1.40)** | **0.75 (0.31; 1.82)** | **0.56 (0.24; 1.30)** | **0.53 (0.20; 1.38)** | **0.46 (0.20; 1.04)** | **R** | **1.53 (0.94; 2.51)** | **.** | **.** |
| **1.05 (0.43; 2.53)** | **0.53 (0.19; 1.48)** | **1.11 (0.55; 2.23)** | **1.02 (0.58; 1.78)** | **1.15 (0.55; 2.40)** | **0.85 (0.43; 1.71)** | **0.81 (0.35; 1.84)** | **0.70 (0.36; 1.35)** | **1.53 (0.94; 2.51)** | **R+D** | **.** | **.** |
| **1.10 (0.54; 2.23)** | **0.55 (0.23; 1.35)** | **1.16 (0.57; 2.38)** | **1.07 (0.53; 2.17)** | **1.21 (0.72; 2.03)** | **0.90 (0.48; 1.69)** | **0.85 (0.45; 1.61)** | **0.74 (0.34; 1.62)** | **1.62 (0.58; 4.52)** | **1.05 (0.43; 2.60)** | **T** | **1.49 (0.89; 2.47)** |
| **1.64 (0.69; 3.91)** | **0.82 (0.30; 2.29)** | **1.73 (0.72; 4.15)** | **1.59 (0.66; 3.80)** | **1.79 (0.87; 3.71)** | **1.33 (0.59; 3.01)** | **1.26 (0.56; 2.85)** | **1.10 (0.43; 2.80)** | **2.40 (0.76; 7.56)** | **1.57 (0.56; 4.41)** | **1.49 (0.89; 2.47)** | **T+D** |

Network calculation of the rate of delayed vomiting

| **Do** | **2.33 (1.46; 3.72)** | **.** | **.** | **1.27 (0.76; 2.12)** | **.** | **.** | **.** | **.** | **.** | **.** | **.** |
| --- | --- | --- | --- | --- | --- | --- | --- | --- | --- | --- | --- |
| **2.33 (1.46; 3.72)** | **Do+D** | **.** | **.** | **.** | **.** | **.** | **.** | **.** | **.** | **.** | **.** |
| **1.36 (0.75; 2.45)** | **0.58 (0.27; 1.24)** | **G** | **0.93 (0.59; 1.48)** | **1.09 (0.74; 1.60)** | **.** | **1.12 (0.69; 1.80)** | **.** | **.** | **.** | **1.14 (0.64; 2.04)** | **.** |
| **1.35 (0.72; 2.55)** | **0.58 (0.26; 1.28)** | **1.00 (0.69; 1.43)** | **G+D** | **.** | **0.99 (0.65; 1.50)** | **.** | **1.67 (1.20; 2.34)** | **.** | **0.97 (0.53; 1.80)** | **.** | **.** |
| **1.27 (0.76; 2.12)** | **0.54 (0.27; 1.09)** | **0.93 (0.70; 1.25)** | **0.94 (0.65; 1.37)** | **O** | **1.29 (0.87; 1.93)** | **1.86 (1.13; 3.05)** | **.** | **.** | **.** | **0.98 (0.69; 1.39)** | **.** |
| **1.56 (0.84; 2.89)** | **0.67 (0.31; 1.45)** | **1.15 (0.79; 1.68)** | **1.16 (0.87; 1.53)** | **1.23 (0.88; 1.73)** | **O+D** | **.** | **1.29 (0.95; 1.74)** | **.** | **.** | **.** | **.** |
| **1.88 (0.99; 3.55)** | **0.80 (0.36; 1.77)** | **1.38 (0.95; 2.01)** | **1.39 (0.85; 2.25)** | **1.48 (1.01; 2.15)** | **1.20 (0.75; 1.93)** | **P** | **.** | **.** | **.** | **.** | **.** |
| **2.12 (1.11; 4.06)** | **0.91 (0.41; 2.02)** | **1.56 (1.04; 2.36)** | **1.57 (1.20; 2.06)** | **1.67 (1.12; 2.48)** | **1.36 (1.05; 1.75)** | **1.13 (0.68; 1.89)** | **P+D** | **.** | **.** | **.** | **.** |
| **0.81 (0.30; 2.18)** | **0.35 (0.12; 1.04)** | **0.60 (0.26; 1.38)** | **0.60 (0.28; 1.28)** | **0.64 (0.27; 1.49)** | **0.52 (0.23; 1.16)** | **0.43 (0.18; 1.06)** | **0.38 (0.17; 0.85)** | **R** | **1.63 (1.04; 2.55)** | **.** | **.** |
| **1.32 (0.54; 3.18)** | **0.56 (0.21; 1.53)** | **0.97 (0.47; 1.98)** | **0.97 (0.53; 1.80)** | **1.04 (0.50; 2.13)** | **0.84 (0.43; 1.65)** | **0.70 (0.32; 1.53)** | **0.62 (0.32; 1.21)** | **1.63 (1.04; 2.55)** | **R+D** | **.** | **.** |
| **1.30 (0.70; 2.41)** | **0.56 (0.26; 1.21)** | **0.96 (0.64; 1.43)** | **0.96 (0.59; 1.56)** | **1.02 (0.73; 1.44)** | **0.83 (0.52; 1.33)** | **0.69 (0.43; 1.13)** | **0.61 (0.37; 1.02)** | **1.61 (0.65; 3.96)** | **0.99 (0.45; 2.16)** | **T** | **1.60 (1.06; 2.41)** |
| **2.08 (0.99; 4.37)** | **0.89 (0.37; 2.14)** | **1.53 (0.86; 2.72)** | **1.54 (0.82; 2.91)** | **1.64 (0.96; 2.79)** | **1.33 (0.72; 2.48)** | **1.11 (0.59; 2.10)** | **0.98 (0.51; 1.88)** | **2.58 (0.96; 6.93)** | **1.58 (0.65; 3.83)** | **1.60 (1.06; 2.41)** | **T+D** |

Sensitivity analysis of the exclusion of data from per-protocol analysis

Network calculation of the rate of acute nausea

| **A+D** | **.** | **.** | **1.16 (0.67; 2.00)** | **.** | **.** | **.** | **.** | **.** | **.** | **.** | **.** |
| --- | --- | --- | --- | --- | --- | --- | --- | --- | --- | --- | --- |
| **0.89 (0.49; 1.61)** | **Do** | **0.98 (0.85; 1.12)** | **.** | **0.99 (0.68; 1.42)** | **.** | **.** | **.** | **.** | **.** | **.** | **.** |
| **0.87 (0.49; 1.55)** | **0.98 (0.86; 1.11)** | **G** | **1.32 (1.07; 1.63)** | **1.00 (0.93; 1.07)** | **.** | **.** | **.** | **1.32 (0.97; 1.81)** | **.** | **0.41 (0.23; 0.73)** | **.** |
| **1.16 (0.67; 2.00)** | **1.30 (1.04; 1.64)** | **1.33 (1.10; 1.61)** | **G+D** | **.** | **1.04 (0.91; 1.19)** | **.** | **0.97 (0.84; 1.12)** | **.** | **1.22 (0.66; 2.23)** | **.** | **.** |
| **0.87 (0.49; 1.55)** | **0.97 (0.85; 1.12)** | **0.99 (0.93; 1.06)** | **0.75 (0.61; 0.91)** | **O** | **1.26 (0.74; 2.12)** | **1.33 (0.80; 2.20)** | **.** | **1.16 (0.72; 1.85)** | **.** | **1.10 (0.78; 1.56)** | **.** |
| **1.15 (0.66; 2.01)** | **1.29 (1.01; 1.66)** | **1.32 (1.07; 1.63)** | **0.99 (0.89; 1.11)** | **1.33 (1.07; 1.66)** | **O+D** | **.** | **1.14 (0.93; 1.38)** | **.** | **.** | **.** | **.** |
| **1.15 (0.54; 2.49)** | **1.30 (0.77; 2.19)** | **1.32 (0.80; 2.20)** | **0.99 (0.58; 1.71)** | **1.33 (0.80; 2.20)** | **1.00 (0.58; 1.73)** | **P** | **.** | **.** | **.** | **.** | **.** |
| **1.19 (0.68; 2.07)** | **1.33 (1.03; 1.72)** | **1.36 (1.09; 1.70)** | **1.02 (0.91; 1.15)** | **1.37 (1.09; 1.72)** | **1.03 (0.90; 1.18)** | **1.03 (0.59; 1.79)** | **P+D** | **.** | **.** | **.** | **.** |
| **1.07 (0.58; 1.99)** | **1.20 (0.91; 1.59)** | **1.23 (0.96; 1.57)** | **0.92 (0.69; 1.24)** | **1.24 (0.96; 1.59)** | **0.93 (0.68; 1.27)** | **0.93 (0.53; 1.63)** | **0.90 (0.66; 1.24)** | **R** | **1.61 (1.16; 2.24)** | **.** | **.** |
| **1.65 (0.86; 3.18)** | **1.85 (1.28; 2.69)** | **1.89 (1.33; 2.69)** | **1.42 (0.99; 2.06)** | **1.90 (1.34; 2.71)** | **1.43 (0.98; 2.10)** | **1.43 (0.77; 2.65)** | **1.39 (0.95; 2.05)** | **1.54 (1.14; 2.07)** | **R+D** | **.** | **.** |
| **0.74 (0.39; 1.41)** | **0.83 (0.60; 1.15)** | **0.85 (0.63; 1.14)** | **0.64 (0.45; 0.91)** | **0.85 (0.63; 1.15)** | **0.64 (0.44; 0.93)** | **0.64 (0.36; 1.15)** | **0.62 (0.43; 0.90)** | **0.69 (0.47; 1.01)** | **0.45 (0.28; 0.71)** | **T** | **1.85 (1.26; 2.74)** |
| **1.37 (0.64; 2.92)** | **1.54 (0.93; 2.56)** | **1.57 (0.96; 2.57)** | **1.18 (0.70; 2.00)** | **1.58 (0.97; 2.58)** | **1.19 (0.70; 2.03)** | **1.19 (0.59; 2.40)** | **1.15 (0.67; 1.98)** | **1.28 (0.74; 2.21)** | **0.83 (0.45; 1.52)** | **1.85 (1.26; 2.74)** | **T+D** |

Network calculation of the rate of acute vomiting

| **Do** | **0.96 (0.81; 1.13)** | **.** | **0.98 (0.86; 1.12)** | **.** | **.** | **.** | **.** | **.** | **.** | **.** |
| --- | --- | --- | --- | --- | --- | --- | --- | --- | --- | --- |
| **0.92 (0.82; 1.04)** | **G** | **1.57 (1.30; 1.89)** | **1.08 (1.00; 1.18)** | **.** | **1.61 (0.95; 2.74)** | **.** | **1.32 (0.95; 1.83)** | **.** | **0.92 (0.66; 1.27)** | **.** |
| **1.41 (1.16; 1.72)** | **1.53 (1.29; 1.80)** | **G+D** | **.** | **1.06 (0.90; 1.26)** | **.** | **1.05 (0.83; 1.32)** | **.** | **0.84 (0.49; 1.46)** | **.** | **.** |
| **1.00 (0.90; 1.12)** | **1.09 (1.01; 1.17)** | **0.71 (0.60; 0.85)** | **O** | **1.06 (0.72; 1.56)** | **1.98 (0.60; 6.51)** | **.** | **1.06 (0.75; 1.49)** | **.** | **0.78 (0.60; 1.01)** | **.** |
| **1.35 (1.08; 1.70)** | **1.46 (1.20; 1.79)** | **0.96 (0.83; 1.10)** | **1.35 (1.10; 1.66)** | **O+D** | **.** | **1.32 (1.10; 1.59)** | **.** | **.** | **.** | **.** |
| **1.56 (0.95; 2.57)** | **1.69 (1.04; 2.74)** | **1.11 (0.66; 1.85)** | **1.56 (0.96; 2.53)** | **1.16 (0.68; 1.95)** | **P** | **.** | **.** | **.** | **.** | **.** |
| **1.66 (1.29; 2.13)** | **1.80 (1.43; 2.25)** | **1.18 (0.99; 1.39)** | **1.65 (1.31; 2.09)** | **1.23 (1.05; 1.43)** | **1.06 (0.62; 1.81)** | **P+D** | **.** | **.** | **.** | **.** |
| **1.09 (0.85; 1.40)** | **1.18 (0.94; 1.48)** | **0.77 (0.59; 1.01)** | **1.09 (0.87; 1.37)** | **0.81 (0.60; 1.08)** | **0.70 (0.41; 1.19)** | **0.66 (0.48; 0.90)** | **R** | **1.58 (1.07; 2.34)** | **.** | **.** |
| **1.52 (1.05; 2.21)** | **1.65 (1.15; 2.36)** | **1.08 (0.75; 1.56)** | **1.52 (1.06; 2.18)** | **1.13 (0.77; 1.66)** | **0.98 (0.53; 1.78)** | **0.92 (0.62; 1.37)** | **1.40 (1.00; 1.94)** | **R+D** | **.** | **.** |
| **0.79 (0.62; 1.02)** | **0.86 (0.68; 1.08)** | **0.56 (0.43; 0.74)** | **0.79 (0.63; 0.99)** | **0.59 (0.44; 0.79)** | **0.51 (0.30; 0.87)** | **0.48 (0.35; 0.66)** | **0.73 (0.53; 1.00)** | **0.52 (0.34; 0.79)** | **T** | **1.99 (1.44; 2.75)** |
| **1.58 (1.05; 2.37)** | **1.71 (1.15; 2.54)** | **1.12 (0.73; 1.71)** | **1.57 (1.06; 2.33)** | **1.17 (0.75; 1.81)** | **1.01 (0.54; 1.88)** | **0.95 (0.60; 1.50)** | **1.45 (0.92; 2.27)** | **1.04 (0.61; 1.76)** | **1.99 (1.44; 2.75)** | **T+D** |

Network calculation of the rate of acute complete control

| **G** | **0.80 (0.61; 1.04)** | **0.96 (0.89; 1.03)** | **.** | **.** | **.** | **.** | **.** | **1.16 (0.92; 1.47)** | **.** |
| --- | --- | --- | --- | --- | --- | --- | --- | --- | --- |
| **0.86 (0.74; 1.01)** | **G+D** | **.** | **0.99 (0.92; 1.06)** | **.** | **0.98 (0.91; 1.05)** | **.** | **1.05 (0.87; 1.27)** | **.** | **.** |
| **0.95 (0.88; 1.02)** | **1.10 (0.95; 1.28)** | **O** | **0.94 (0.80; 1.12)** | **0.98 (0.91; 1.05)** | **.** | **.** | **.** | **1.13 (0.89; 1.44)** | **.** |
| **0.87 (0.74; 1.01)** | **1.01 (0.95; 1.07)** | **0.91 (0.79; 1.06)** | **O+D** | **.** | **0.93 (0.84; 1.03)** | **.** | **.** | **.** | **.** |
| **0.93 (0.83; 1.03)** | **1.08 (0.91; 1.27)** | **0.98 (0.91; 1.05)** | **1.07 (0.91; 1.26)** | **P** | **.** | **.** | **.** | **.** | **.** |
| **0.83 (0.71; 0.98)** | **0.96 (0.91; 1.03)** | **0.87 (0.75; 1.02)** | **0.96 (0.89; 1.03)** | **0.90 (0.75; 1.07)** | **P+D** | **.** | **.** | **.** | **.** |
| **1.14 (0.83; 1.57)** | **1.33 (1.01; 1.74)** | **1.20 (0.88; 1.64)** | **1.32 (1.00; 1.74)** | **1.23 (0.90; 1.70)** | **1.37 (1.04; 1.82)** | **R** | **0.79 (0.65; 0.96)** | **.** | **.** |
| **0.91 (0.71; 1.16)** | **1.05 (0.87; 1.27)** | **0.95 (0.75; 1.22)** | **1.04 (0.85; 1.28)** | **0.98 (0.76; 1.26)** | **1.09 (0.89; 1.33)** | **0.79 (0.65; 0.96)** | **R+D** | **.** | **.** |
| **1.12 (0.90; 1.39)** | **1.30 (1.00; 1.69)** | **1.18 (0.95; 1.47)** | **1.29 (0.99; 1.67)** | **1.21 (0.96; 1.52)** | **1.35 (1.03; 1.76)** | **0.98 (0.67; 1.43)** | **1.24 (0.89; 1.71)** | **T** | **0.65 (0.53; 0.80)** |
| **0.73 (0.54; 0.98)** | **0.84 (0.60; 1.18)** | **0.77 (0.57; 1.03)** | **0.84 (0.60; 1.17)** | **0.78 (0.57; 1.07)** | **0.87 (0.62; 1.23)** | **0.64 (0.41; 0.98)** | **0.80 (0.55; 1.18)** | **0.65 (0.53; 0.80)** | **T+D** |

Network calculation of the rate of delayed nausea

| **Do** | **1.99 (1.28; 3.08)** | **.** | **.** | **0.91 (0.63; 1.31)** | **.** | **.** | **.** | **.** | **.** | **.** | **.** |
| --- | --- | --- | --- | --- | --- | --- | --- | --- | --- | --- | --- |
| **1.99 (1.28; 3.08)** | **Do+D** | **.** | **.** | **.** | **.** | **.** | **.** | **.** | **.** | **.** | **.** |
| **1.00 (0.59; 1.69)** | **0.50 (0.25; 1.00)** | **G** | **0.97 (0.67; 1.40)** | **1.17 (0.68; 2.02)** | **.** | **.** | **.** | **.** | **.** | **.** | **.** |
| **1.08 (0.65; 1.79)** | **0.54 (0.28; 1.06)** | **1.09 (0.79; 1.50)** | **G+D** | **.** | **1.07 (0.76; 1.51)** | **.** | **1.41 (1.09; 1.83)** | **.** | **1.02 (0.64; 1.61)** | **.** | **.** |
| **0.91 (0.63; 1.31)** | **0.46 (0.26; 0.81)** | **0.92 (0.62; 1.35)** | **0.84 (0.60; 1.19)** | **O** | **1.43 (1.03; 1.98)** | **1.42 (1.04; 1.95)** | **.** | **.** | **.** | **1.21 (0.80; 1.83)** | **.** |
| **1.20 (0.75; 1.91)** | **0.60 (0.32; 1.14)** | **1.20 (0.84; 1.73)** | **1.11 (0.87; 1.40)** | **1.31 (0.98; 1.76)** | **O+D** | **.** | **1.37 (1.06; 1.78)** | **.** | **.** | **.** | **.** |
| **1.30 (0.80; 2.10)** | **0.65 (0.34; 1.25)** | **1.30 (0.79; 2.15)** | **1.20 (0.75; 1.92)** | **1.42 (1.04; 1.95)** | **1.09 (0.70; 1.67)** | **P** | **.** | **.** | **.** | **.** | **.** |
| **1.58 (0.95; 2.62)** | **0.80 (0.41; 1.55)** | **1.59 (1.09; 2.30)** | **1.46 (1.18; 1.82)** | **1.73 (1.22; 2.46)** | **1.32 (1.06; 1.64)** | **1.22 (0.76; 1.95)** | **P+D** | **.** | **.** | **.** | **.** |
| **0.72 (0.33; 1.57)** | **0.36 (0.15; 0.88)** | **0.72 (0.36; 1.42)** | **0.66 (0.36; 1.21)** | **0.78 (0.39; 1.57)** | **0.60 (0.31; 1.14)** | **0.55 (0.26; 1.18)** | **0.45 (0.24; 0.86)** | **R** | **1.53 (1.05; 2.24)** | **.** | **.** |
| **1.10 (0.55; 2.18)** | **0.55 (0.25; 1.24)** | **1.10 (0.63; 1.94)** | **1.02 (0.64; 1.61)** | **1.20 (0.67; 2.15)** | **0.92 (0.55; 1.54)** | **0.84 (0.44; 1.63)** | **0.69 (0.42; 1.16)** | **1.53 (1.05; 2.24)** | **R+D** | **.** | **.** |
| **1.10 (0.64; 1.91)** | **0.55 (0.27; 1.12)** | **1.11 (0.63; 1.95)** | **1.02 (0.59; 1.75)** | **1.21 (0.80; 1.83)** | **0.92 (0.55; 1.53)** | **0.85 (0.50; 1.43)** | **0.70 (0.41; 1.20)** | **1.54 (0.69; 3.45)** | **1.00 (0.49; 2.05)** | **T** | **1.49 (1.00; 2.22)** |
| **1.64 (0.83; 3.23)** | **0.82 (0.37; 1.85)** | **1.64 (0.82; 3.29)** | **1.51 (0.77; 2.96)** | **1.79 (1.01; 3.19)** | **1.37 (0.72; 2.61)** | **1.26 (0.65; 2.43)** | **1.04 (0.53; 2.03)** | **2.29 (0.93; 5.63)** | **1.49 (0.66; 3.37)** | **1.49 (1.00; 2.22)** | **T+D** |

Network calculation of the rate of delayed vomiting

| **Do** | **2.33 (1.54; 3.53)** | **.** | **.** | **1.27 (0.80; 2.02)** | **.** | **.** | **.** | **.** | **.** | **.** | **.** |
| --- | --- | --- | --- | --- | --- | --- | --- | --- | --- | --- | --- |
| **2.33 (1.54; 3.53)** | **Do+D** | **.** | **.** | **.** | **.** | **.** | **.** | **.** | **.** | **.** | **.** |
| **1.36 (0.80; 2.34)** | **0.59 (0.30; 1.15)** | **G** | **0.93 (0.62; 1.40)** | **1.09 (0.77; 1.55)** | **.** | **1.12 (0.73; 1.71)** | **.** | **.** | **.** | **1.14 (0.67; 1.96)** | **.** |
| **1.37 (0.77; 2.42)** | **0.59 (0.29; 1.19)** | **1.00 (0.72; 1.38)** | **G+D** | **.** | **0.99 (0.69; 1.41)** | **.** | **1.60 (1.20; 2.13)** | **.** | **0.97 (0.55; 1.73)** | **.** | **.** |
| **1.27 (0.80; 2.02)** | **0.54 (0.29; 1.01)** | **0.93 (0.71; 1.21)** | **0.93 (0.67; 1.29)** | **O** | **1.28 (0.89; 1.84)** | **1.88 (1.17; 3.02)** | **.** | **.** | **.** | **0.98 (0.71; 1.33)** | **.** |
| **1.54 (0.88; 2.68)** | **0.66 (0.33; 1.32)** | **1.13 (0.81; 1.57)** | **1.13 (0.90; 1.42)** | **1.21 (0.89; 1.64)** | **O+D** | **.** | **1.33 (1.10; 1.60)** | **.** | **.** | **.** | **.** |
| **1.86 (1.04; 3.34)** | **0.80 (0.39; 1.63)** | **1.36 (0.97; 1.92)** | **1.36 (0.88; 2.11)** | **1.47 (1.03; 2.08)** | **1.21 (0.79; 1.87)** | **P** | **.** | **.** | **.** | **.** | **.** |
| **2.08 (1.17; 3.69)** | **0.89 (0.44; 1.81)** | **1.52 (1.07; 2.16)** | **1.52 (1.22; 1.91)** | **1.64 (1.17; 2.29)** | **1.35 (1.14; 1.60)** | **1.12 (0.71; 1.75)** | **P+D** | **.** | **.** | **.** | **.** |
| **0.82 (0.33; 2.01)** | **0.35 (0.13; 0.94)** | **0.60 (0.28; 1.29)** | **0.60 (0.30; 1.20)** | **0.64 (0.30; 1.39)** | **0.53 (0.26; 1.11)** | **0.44 (0.19; 1.00)** | **0.39 (0.19; 0.82)** | **R** | **1.63 (1.10; 2.41)** | **.** | **.** |
| **1.33 (0.59; 2.99)** | **0.57 (0.23; 1.41)** | **0.97 (0.50; 1.88)** | **0.97 (0.55; 1.73)** | **1.05 (0.54; 2.03)** | **0.86 (0.47; 1.60)** | **0.71 (0.35; 1.47)** | **0.64 (0.34; 1.18)** | **1.63 (1.10; 2.41)** | **R+D** | **.** | **.** |
| **1.30 (0.74; 2.26)** | **0.56 (0.28; 1.11)** | **0.95 (0.66; 1.37)** | **0.95 (0.62; 1.46)** | **1.02 (0.75; 1.38)** | **0.84 (0.56; 1.28)** | **0.70 (0.45; 1.09)** | **0.62 (0.40; 0.96)** | **1.59 (0.70; 3.60)** | **0.98 (0.48; 2.00)** | **T** | **1.59 (1.10; 2.28)** |
| **2.06 (1.06; 4.00)** | **0.88 (0.40; 1.93)** | **1.51 (0.90; 2.52)** | **1.51 (0.86; 2.64)** | **1.62 (1.01; 2.60)** | **1.34 (0.77; 2.32)** | **1.10 (0.62; 1.96)** | **0.99 (0.56; 1.74)** | **2.52 (1.03; 6.16)** | **1.55 (0.69; 3.46)** | **1.59 (1.10; 2.28)** | **T+D** |

Network calculation of the rate of delayed complete control

| **G** | **1.12 (0.75; 1.67)** | **.** | **.** | **.** | **.** | **.** |
| --- | --- | --- | --- | --- | --- | --- |
| **1.12 (0.75; 1.67)** | **G+D** | **.** | **0.94 (0.75; 1.17)** | **.** | **0.80 (0.65; 0.99)** | **0.92 (0.58; 1.47)** |
| **1.82 (1.09; 3.06)** | **1.63 (1.18; 2.27)** | **O** | **0.61 (0.47; 0.81)** | **0.70 (0.49; 0.99)** | **.** | **.** |
| **1.12 (0.72; 1.73)** | **1.00 (0.84; 1.20)** | **0.61 (0.47; 0.81)** | **O+D** | **.** | **0.70 (0.56; 0.88)** | **.** |
| **1.27 (0.68; 2.37)** | **1.14 (0.71; 1.84)** | **0.70 (0.49; 0.99)** | **1.14 (0.73; 1.77)** | **P** | **.** | **.** |
| **0.84 (0.54; 1.30)** | **0.75 (0.63; 0.90)** | **0.46 (0.33; 0.64)** | **0.75 (0.63; 0.90)** | **0.66 (0.41; 1.06)** | **P+D** | **.** |
| **1.03 (0.56; 1.90)** | **0.92 (0.58; 1.47)** | **0.57 (0.32; 1.00)** | **0.92 (0.56; 1.51)** | **0.81 (0.42; 1.58)** | **1.23 (0.75; 2.01)** | **R+D** |

A brief Bayesian sensitivity analysis :

Taking acute CC as an example, we evaluated the robustness of the model by comparing the results under two different prior distributions.

Scenario 1 (Weakly Informative Prior): A "Half-Cauchy"(0, 0.5) prior is used for τ. This is a commonly used weakly informative prior that favors smaller heterogeneity.

Scenario 2 (Less Informative Prior): A "Half-Cauchy"(0, 2.5) prior is used for τ. This is a flatter prior that allows for larger heterogeneity, used to test the sensitivity of the results to prior selection.

The table below shows the posterior estimates of the heterogeneity standard deviation under two different prior distributions.

τ(Mean) 95% HDI Lower Bound (hdi_3%) 95% HDI Upper Bound (hdi_97%)

Scenario 1 "Half-Cauchy"(0,0.5) 0.200 0.050 0.400

"Scenario 2 Half-Cauchy"(0,2.5) 0.250 0.030 0.550

From the results, it can be observed that when using the less informative prior (Scenario 2), the posterior mean of τ increased slightly (from 0.200 to 0.250), and the 95% credible interval (HDI) widened somewhat, which aligns with expectations. However, the estimates for τ in both scenarios remain at relatively low levels, indicating a moderate degree of heterogeneity.

The table below presents the posterior estimates RR for each treatment regimen relative to the reference treatment (assumed to be G, i.e., granisetron monotherapy) under the two scenarios.

Comparision RR (Mean) 95% HDI Lower Bound 95% HDI Upper Bound Scenario

T vs G 1.028 0.877 1.149 Scenario 1

T vs G 1.028 0.865 1.170 Scenario 2

G+D vs G 1.054 0.910 1.171 Scenario 1

G+D vs G 1.054 0.900 1.185 Scenario 2

O vs G 0.989 0.828 1.117 Scenario 1

O vs G 0.989 0.811 1.133 Scenario 2

P+D vs G 1.103 0.971 1.211 Scenario 1

P+D vs G 1.103 0.956 1.226 Scenario 2

The two prior choices had minimal impact on the posterior meanRR for key treatment comparisons, which remained consistent. Although the 95% HDI intervals were slightly wider under the less informative prior (Scenario 2) (e.g., the HDI upper bound for P+D vs. G increased from 1.211 to 1.226), this did not alter the statistical significance or clinical interpretation of any comparison. The RR point estimates and direction remained unchanged across all comparisons, indicating that the main conclusions of this study are robust with respect to the prior choice for the heterogeneity parameter τ.
