# Supplementary material for: Comparative efficacy of 5-hydroxytryptamine-3 (5-HT3) receptor antagonists with or without dexamethasone for prevention of chemotherapy-induced nausea and vomiting following highly emetogenic chemotherapy (HEC): a network meta-analysis
Source: PeerJ. 2026 Apr 2;14:e21047. doi: 10.7717/peerj.21047 (PMC13050518; doi:10.7717/peerj.21047)
Supplement: Supplemental Information 5 [file peerj-14-21047-s005.docx]

**The leave-one-out analysis**

1、The outcomes after the article of “Aapro MS 2006” was deleted.

Acute vomiting

| Do | 0.96 (0.81; 1.13) | . | 0.98 (0.86; 1.12) | . | . | . | . | . | . | . |
| --- | --- | --- | --- | --- | --- | --- | --- | --- | --- | --- |
| 0.93 (0.83; 1.04) | G | 1.57 (1.31; 1.89) | 1.08 (1.00; 1.18) | . | 1.61 (0.95; 2.73) | . | 1.32 (0.96; 1.83) | . | 0.92 (0.67; 1.27) | . |
| 1.44 (1.18; 1.74) | 1.55 (1.32; 1.83) | G+D | . | 1.06 (0.90; 1.26) | . | 1.05 (0.83; 1.32) | . | 0.84 (0.49; 1.46) | . | . |
| 1.00 (0.90; 1.11) | 1.08 (1.01; 1.17) | 0.70 (0.59; 0.83) | O | 1.26 (0.88; 1.81) | 1.98 (0.60; 6.51) | . | 1.06 (0.75; 1.49) | . | 0.78 (0.60; 1.01) | . |
| 1.47 (1.18; 1.83) | 1.59 (1.30; 1.93) | 1.02 (0.89; 1.18) | 1.47 (1.20; 1.79) | O+D | . | 1.07 (0.81; 1.43) | . | . | . | . |
| 1.56 (0.95; 2.56) | 1.69 (1.04; 2.74) | 1.09 (0.65; 1.81) | 1.56 (0.96; 2.54) | 1.06 (0.63; 1.79) | P | . | . | . | . | . |
| 1.53 (1.18; 1.99) | 1.66 (1.30; 2.11) | 1.07 (0.89; 1.29) | 1.53 (1.19; 1.96) | 1.04 (0.86; 1.27) | 0.98 (0.57; 1.68) | P+D | . | . | . | . |
| 1.09 (0.86; 1.40) | 1.18 (0.94; 1.48) | 0.76 (0.58; 0.99) | 1.09 (0.87; 1.37) | 0.74 (0.56; 0.99) | 0.70 (0.41; 1.19) | 0.71 (0.52; 0.98) | R | 1.58 (1.08; 2.33) | . | . |
| 1.54 (1.06; 2.23) | 1.66 (1.16; 2.37) | 1.07 (0.74; 1.54) | 1.53 (1.07; 2.19) | 1.05 (0.71; 1.53) | 0.98 (0.54; 1.79) | 1.00 (0.67; 1.50) | 1.40 (1.01; 1.95) | R+D | . | . |
| 0.79 (0.62; 1.01) | 0.86 (0.69; 1.08) | 0.55 (0.42; 0.73) | 0.79 (0.63; 0.99) | 0.54 (0.40; 0.73) | 0.51 (0.30; 0.86) | 0.52 (0.37; 0.72) | 0.73 (0.53; 0.99) | 0.52 (0.34; 0.79) | T | 1.99 (1.44; 2.74) |
| 1.58 (1.05; 2.36) | 1.70 (1.15; 2.52) | 1.10 (0.72; 1.68) | 1.57 (1.06; 2.33) | 1.07 (0.69; 1.66) | 1.01 (0.54; 1.88) | 1.03 (0.65; 1.63) | 1.44 (0.92; 2.26) | 1.03 (0.61; 1.74) | 1.99 (1.44; 2.74) | T+D |

P-score

P+D 0.8960

T+D 0.7616

P 0.7447

R+D 0.7430

G+D 0.6797

O+D 0.6302

R 0.3635

Do 0.2757

O 0.2709

G 0.1155

T 0.0190

| Do | 2.33 (1.45; 3.75) | . | . | 1.27 (0.75; 2.14) | . | . | . | . | . | . | . |
| --- | --- | --- | --- | --- | --- | --- | --- | --- | --- | --- | --- |
| 2.33 (1.45; 3.75) | Do+D | . | . | . | . | . | . | . | . | . | . |
| 1.36 (0.75; 2.49) | 0.58 (0.27; 1.26) | G | 0.93 (0.58; 1.49) | 1.09 (0.74; 1.61) | . | 1.12 (0.68; 1.82) | . | . | . | 1.14 (0.63; 2.06) | . |
| 1.37 (0.72; 2.62) | 0.59 (0.26; 1.31) | 1.01 (0.69; 1.46) | G+D | . | 0.99 (0.65; 1.52) | . | 1.69 (1.20; 2.37) | . | 0.97 (0.52; 1.81) | . | . |
| 1.27 (0.75; 2.14) | 0.54 (0.27; 1.10) | 0.93 (0.69; 1.25) | 0.93 (0.63; 1.36) | O | 1.30 (0.87; 1.94) | 1.85 (1.12; 3.06) | . | . | . | 0.98 (0.68; 1.40) | . |
| 1.55 (0.83; 2.91) | 0.67 (0.30; 1.46) | 1.14 (0.78; 1.67) | 1.13 (0.85; 1.51) | 1.22 (0.87; 1.73) | O+D | . | 1.37 (1.00; 1.87) | . | . | . | . |
| 1.88 (0.98; 3.60) | 0.81 (0.36; 1.80) | 1.38 (0.94; 2.02) | 1.37 (0.84; 2.25) | 1.48 (1.01; 2.17) | 1.21 (0.75; 1.96) | P | . | . | . | . | . |
| 2.21 (1.14; 4.28) | 0.95 (0.42; 2.14) | 1.62 (1.06; 2.47) | 1.61 (1.22; 2.13) | 1.74 (1.16; 2.61) | 1.42 (1.09; 1.85) | 1.17 (0.70; 1.98) | P+D | . | . | . | . |
| 0.82 (0.30; 2.25) | 0.35 (0.12; 1.07) | 0.60 (0.26; 1.42) | 0.60 (0.28; 1.29) | 0.65 (0.27; 1.53) | 0.53 (0.23; 1.20) | 0.44 (0.17; 1.09) | 0.37 (0.16; 0.84) | R | 1.63 (1.03; 2.57) | . | . |
| 1.33 (0.54; 3.28) | 0.57 (0.21; 1.58) | 0.98 (0.47; 2.02) | 0.97 (0.52; 1.81) | 1.05 (0.51; 2.18) | 0.86 (0.43; 1.70) | 0.71 (0.32; 1.57) | 0.60 (0.31; 1.19) | 1.63 (1.03; 2.57) | R+D | . | . |
| 1.30 (0.70; 2.44) | 0.56 (0.25; 1.23) | 0.95 (0.63; 1.44) | 0.95 (0.58; 1.56) | 1.03 (0.73; 1.45) | 0.84 (0.52; 1.35) | 0.69 (0.42; 1.14) | 0.59 (0.35; 0.99) | 1.59 (0.63; 3.97) | 0.98 (0.44; 2.16) | T | 1.60 (1.06; 2.44) |
| 2.09 (0.98; 4.44) | 0.90 (0.37; 2.19) | 1.53 (0.85; 2.75) | 1.52 (0.80; 2.91) | 1.65 (0.96; 2.83) | 1.34 (0.71; 2.53) | 1.11 (0.58; 2.12) | 0.95 (0.49; 1.84) | 2.55 (0.93; 6.98) | 1.57 (0.64; 3.84) | 1.60 (1.06; 2.44) | T+D |

Delayed vomiting

**P-score**

**P+D 0.8731**

**Do+D 0.8377**

**T+D 0.8029**

**P 0.7473**

**O+D 0.5703**

**R+D 0.4221**

**G+D 0.4106**

**G 0.4072**

**T 0.3559**

**O 0.3123**

**Do 0.1618**

**R 0.0990**

2、The outcomes after the article of “Aksoylar S 2001” was deleted.

Acute nausea

| Do | 2.33 (1.45; 3.75) | . | . | 1.27 (0.75; 2.14) | . | . | . | . | . | . | . |
| --- | --- | --- | --- | --- | --- | --- | --- | --- | --- | --- | --- |
| 2.33 (1.45; 3.75) | Do+D | . | . | . | . | . | . | . | . | . | . |
| 1.36 (0.75; 2.49) | 0.58 (0.27; 1.26) | G | 0.93 (0.58; 1.49) | 1.09 (0.74; 1.61) | . | 1.12 (0.68; 1.82) | . | . | . | 1.14 (0.63; 2.06) | . |
| 1.37 (0.72; 2.62) | 0.59 (0.26; 1.31) | 1.01 (0.69; 1.46) | G+D | . | 0.99 (0.65; 1.52) | . | 1.69 (1.20; 2.37) | . | 0.97 (0.52; 1.81) | . | . |
| 1.27 (0.75; 2.14) | 0.54 (0.27; 1.10) | 0.93 (0.69; 1.25) | 0.93 (0.63; 1.36) | O | 1.30 (0.87; 1.94) | 1.85 (1.12; 3.06) | . | . | . | 0.98 (0.68; 1.40) | . |
| 1.55 (0.83; 2.91) | 0.67 (0.30; 1.46) | 1.14 (0.78; 1.67) | 1.13 (0.85; 1.51) | 1.22 (0.87; 1.73) | O+D | . | 1.37 (1.00; 1.87) | . | . | . | . |
| 1.88 (0.98; 3.60) | 0.81 (0.36; 1.80) | 1.38 (0.94; 2.02) | 1.37 (0.84; 2.25) | 1.48 (1.01; 2.17) | 1.21 (0.75; 1.96) | P | . | . | . | . | . |
| 2.21 (1.14; 4.28) | 0.95 (0.42; 2.14) | 1.62 (1.06; 2.47) | 1.61 (1.22; 2.13) | 1.74 (1.16; 2.61) | 1.42 (1.09; 1.85) | 1.17 (0.70; 1.98) | P+D | . | . | . | . |
| 0.82 (0.30; 2.25) | 0.35 (0.12; 1.07) | 0.60 (0.26; 1.42) | 0.60 (0.28; 1.29) | 0.65 (0.27; 1.53) | 0.53 (0.23; 1.20) | 0.44 (0.17; 1.09) | 0.37 (0.16; 0.84) | R | 1.63 (1.03; 2.57) | . | . |
| 1.33 (0.54; 3.28) | 0.57 (0.21; 1.58) | 0.98 (0.47; 2.02) | 0.97 (0.52; 1.81) | 1.05 (0.51; 2.18) | 0.86 (0.43; 1.70) | 0.71 (0.32; 1.57) | 0.60 (0.31; 1.19) | 1.63 (1.03; 2.57) | R+D | . | . |
| 1.30 (0.70; 2.44) | 0.56 (0.25; 1.23) | 0.95 (0.63; 1.44) | 0.95 (0.58; 1.56) | 1.03 (0.73; 1.45) | 0.84 (0.52; 1.35) | 0.69 (0.42; 1.14) | 0.59 (0.35; 0.99) | 1.59 (0.63; 3.97) | 0.98 (0.44; 2.16) | T | 1.60 (1.06; 2.44) |
| 2.09 (0.98; 4.44) | 0.90 (0.37; 2.19) | 1.53 (0.85; 2.75) | 1.52 (0.80; 2.91) | 1.65 (0.96; 2.83) | 1.34 (0.71; 2.53) | 1.11 (0.58; 2.12) | 0.95 (0.49; 1.84) | 2.55 (0.93; 6.98) | 1.57 (0.64; 3.84) | 1.60 (1.06; 2.44) | T+D |

P-score

P+D 0.8731

Do+D 0.8377

T+D 0.8029

P 0.7473

O+D 0.5703

R+D 0.4221

G+D 0.4106

G 0.4072

T 0.3559

O 0.3123

Do 0.1618

R 0.0990

Acute vomiting

| Do | 0.96 (0.80; 1.14) | . | 0.98 (0.85; 1.14) | . | . | . | . | . | . | . |
| --- | --- | --- | --- | --- | --- | --- | --- | --- | --- | --- |
| 0.93 (0.82; 1.05) | G | 1.57 (1.29; 1.90) | 1.09 (0.99; 1.19) | . | 1.61 (0.94; 2.75) | . | 1.32 (0.94; 1.85) | . | 1.06 (0.74; 1.53) | . |
| 1.45 (1.18; 1.78) | 1.57 (1.32; 1.86) | G+D | . | 1.06 (0.89; 1.27) | . | 1.05 (0.82; 1.34) | . | 0.84 (0.48; 1.46) | . | . |
| 1.01 (0.90; 1.13) | 1.09 (1.00; 1.18) | 0.69 (0.58; 0.83) | O | 1.27 (0.88; 1.83) | 1.98 (0.60; 6.52) | . | 1.06 (0.74; 1.51) | . | 0.78 (0.60; 1.02) | . |
| 1.41 (1.12; 1.78) | 1.53 (1.24; 1.87) | 0.97 (0.84; 1.12) | 1.40 (1.14; 1.73) | O+D | . | 1.32 (1.09; 1.59) | . | . | . | . |
| 1.57 (0.95; 2.59) | 1.69 (1.04; 2.76) | 1.08 (0.64; 1.81) | 1.56 (0.95; 2.55) | 1.11 (0.65; 1.88) | P | . | . | . | . | . |
| 1.73 (1.33; 2.24) | 1.87 (1.48; 2.35) | 1.19 (1.00; 1.42) | 1.72 (1.35; 2.18) | 1.22 (1.04; 1.43) | 1.10 (0.64; 1.89) | P+D | . | . | . | . |
| 1.10 (0.85; 1.41) | 1.18 (0.94; 1.49) | 0.75 (0.57; 0.99) | 1.09 (0.86; 1.38) | 0.78 (0.58; 1.04) | 0.70 (0.41; 1.20) | 0.63 (0.46; 0.87) | R | 1.58 (1.06; 2.36) | . | . |
| 1.54 (1.05; 2.25) | 1.66 (1.15; 2.39) | 1.06 (0.73; 1.53) | 1.53 (1.06; 2.21) | 1.09 (0.74; 1.61) | 0.98 (0.53; 1.81) | 0.89 (0.59; 1.34) | 1.40 (1.00; 1.97) | R+D | . | . |
| 0.84 (0.65; 1.09) | 0.91 (0.71; 1.16) | 0.58 (0.43; 0.78) | 0.84 (0.66; 1.06) | 0.60 (0.44; 0.82) | 0.54 (0.31; 0.93) | 0.49 (0.35; 0.68) | 0.77 (0.55; 1.07) | 0.55 (0.35; 0.85) | T | 1.99 (1.43; 2.77) |
| 1.67 (1.10; 2.55) | 1.81 (1.20; 2.73) | 1.15 (0.74; 1.79) | 1.66 (1.11; 2.50) | 1.19 (0.75; 1.87) | 1.07 (0.57; 2.02) | 0.97 (0.61; 1.55) | 1.53 (0.96; 2.44) | 1.09 (0.63; 1.88) | 1.99 (1.43; 2.77) | T+D |

P-score

P+D 0.8866

T+D 0.8098

R+D 0.7378

P 0.7362

G+D 0.6681

O+D 0.6224

R 0.3609

O 0.2747

Do 0.2561

G 0.1006

T 0.0468

3、The outcomes after the article of “Audhuy B 1996” was deleted.

Acute nausea

| A+D | . | . | 1.16 (0.67; 2.01) | . | . | . | . | . | . | . | . |
| --- | --- | --- | --- | --- | --- | --- | --- | --- | --- | --- | --- |
| 0.88 (0.44; 1.76) | Do | . | . | 0.99 (0.68; 1.43) | . | . | . | . | . | . | . |
| 0.87 (0.49; 1.56) | 0.99 (0.68; 1.46) | G | 1.32 (1.06; 1.64) | 1.00 (0.93; 1.09) | . | . | . | 1.32 (0.95; 1.83) | . | 0.41 (0.23; 0.73) | . |
| 1.16 (0.67; 2.01) | 1.32 (0.86; 2.03) | 1.33 (1.09; 1.62) | G+D | . | 1.04 (0.90; 1.20) | . | 0.97 (0.82; 1.14) | . | 1.22 (0.66; 2.24) | . | . |
| 0.87 (0.48; 1.56) | 0.99 (0.68; 1.43) | 0.99 (0.92; 1.07) | 0.75 (0.61; 0.92) | O | 1.26 (0.74; 2.14) | 1.33 (0.80; 2.21) | . | 1.16 (0.72; 1.87) | . | 1.10 (0.77; 1.57) | . |
| 1.15 (0.66; 2.02) | 1.31 (0.84; 2.04) | 1.32 (1.06; 1.65) | 0.99 (0.88; 1.12) | 1.33 (1.05; 1.68) | O+D | . | 1.14 (0.93; 1.39) | . | . | . | . |
| 1.15 (0.53; 2.50) | 1.31 (0.70; 2.46) | 1.32 (0.79; 2.20) | 0.99 (0.57; 1.72) | 1.33 (0.80; 2.21) | 1.00 (0.57; 1.75) | P | . | . | . | . | . |
| 1.20 (0.68; 2.10) | 1.36 (0.87; 2.13) | 1.37 (1.08; 1.74) | 1.03 (0.90; 1.18) | 1.38 (1.08; 1.76) | 1.04 (0.90; 1.20) | 1.04 (0.59; 1.82) | P+D | . | . | . | . |
| 1.07 (0.57; 2.00) | 1.22 (0.77; 1.92) | 1.23 (0.95; 1.58) | 0.92 (0.68; 1.25) | 1.24 (0.96; 1.60) | 0.93 (0.67; 1.28) | 0.93 (0.53; 1.64) | 0.90 (0.64; 1.25) | R | 1.61 (1.15; 2.26) | . | . |
| 1.65 (0.85; 3.20) | 1.87 (1.11; 3.16) | 1.89 (1.32; 2.70) | 1.42 (0.97; 2.07) | 1.90 (1.32; 2.73) | 1.43 (0.97; 2.12) | 1.43 (0.77; 2.66) | 1.38 (0.92; 2.05) | 1.54 (1.13; 2.09) | R+D | . | . |
| 0.73 (0.38; 1.42) | 0.84 (0.52; 1.35) | 0.84 (0.62; 1.14) | 0.63 (0.44; 0.91) | 0.85 (0.62; 1.15) | 0.64 (0.44; 0.93) | 0.64 (0.35; 1.15) | 0.61 (0.42; 0.90) | 0.69 (0.46; 1.02) | 0.45 (0.28; 0.71) | T | 1.85 (1.25; 2.76) |
| 1.36 (0.63; 2.94) | 1.55 (0.83; 2.90) | 1.56 (0.94; 2.58) | 1.17 (0.68; 2.01) | 1.57 (0.95; 2.59) | 1.18 (0.68; 2.05) | 1.18 (0.58; 2.41) | 1.14 (0.65; 1.98) | 1.27 (0.72; 2.23) | 0.83 (0.45; 1.53) | 1.85 (1.25; 2.76) | T+D |

P-score

R+D 0.9439

T+D 0.7707

P+D 0.6960

G+D 0.6370

O+D 0.6170

P 0.5995

R 0.5217

A+D 0.4334

Do 0.2690

G 0.2283

O 0.2099

T 0.0736

Acute vomiting

| Do | . | . | 0.99 (0.84; 1.16) | . | . | . | . | . | . | . |
| --- | --- | --- | --- | --- | --- | --- | --- | --- | --- | --- |
| 0.90 (0.75; 1.09) | G | 1.57 (1.28; 1.92) | 1.09 (0.99; 1.20) | . | 1.61 (0.93; 2.78) | . | 1.32 (0.93; 1.88) | . | 0.91 (0.65; 1.28) | . |
| 1.42 (1.11; 1.82) | 1.57 (1.32; 1.88) | G+D | . | 1.06 (0.88; 1.28) | . | 1.05 (0.80; 1.37) | . | 0.84 (0.48; 1.47) | . | . |
| 0.99 (0.84; 1.16) | 1.09 (0.99; 1.20) | 0.69 (0.57; 0.84) | O | 1.28 (0.88; 1.86) | 1.98 (0.60; 6.53) | . | 1.06 (0.73; 1.53) | . | 0.78 (0.59; 1.03) | . |
| 1.38 (1.05; 1.81) | 1.52 (1.23; 1.89) | 0.97 (0.83; 1.13) | 1.40 (1.12; 1.74) | O+D | . | 1.32 (1.08; 1.60) | . | . | . | . |
| 1.53 (0.91; 2.60) | 1.70 (1.03; 2.78) | 1.08 (0.64; 1.82) | 1.55 (0.94; 2.57) | 1.11 (0.65; 1.91) | P | . | . | . | . | . |
| 1.69 (1.26; 2.28) | 1.87 (1.47; 2.39) | 1.19 (0.99; 1.44) | 1.72 (1.33; 2.21) | 1.23 (1.04; 1.45) | 1.10 (0.64; 1.92) | P+D | . | . | . | . |
| 1.07 (0.80; 1.43) | 1.18 (0.93; 1.51) | 0.75 (0.57; 1.00) | 1.08 (0.85; 1.38) | 0.78 (0.57; 1.06) | 0.70 (0.40; 1.21) | 0.63 (0.45; 0.88) | R | 1.58 (1.05; 2.39) | . | . |
| 1.50 (1.00; 2.26) | 1.66 (1.14; 2.41) | 1.05 (0.72; 1.54) | 1.52 (1.04; 2.21) | 1.09 (0.73; 1.62) | 0.98 (0.53; 1.82) | 0.88 (0.58; 1.34) | 1.40 (0.99; 1.98) | R+D | . | . |
| 0.78 (0.58; 1.03) | 0.86 (0.67; 1.09) | 0.55 (0.41; 0.73) | 0.79 (0.62; 1.00) | 0.56 (0.41; 0.77) | 0.51 (0.29; 0.88) | 0.46 (0.33; 0.64) | 0.72 (0.52; 1.01) | 0.52 (0.33; 0.80) | T | 1.99 (1.42; 2.80) |
| 1.55 (0.99; 2.41) | 1.71 (1.13; 2.59) | 1.09 (0.69; 1.71) | 1.57 (1.03; 2.37) | 1.12 (0.70; 1.78) | 1.01 (0.53; 1.92) | 0.91 (0.56; 1.48) | 1.45 (0.90; 2.33) | 1.03 (0.59; 1.80) | 1.99 (1.42; 2.80) | T+D |

P-score

P+D 0.8954

T+D 0.7620

P 0.7422

R+D 0.7399

G+D 0.6795

O+D 0.6301

R 0.3574

Do 0.2826

O 0.2715

G 0.1185

T 0.0211

4、The outcomes after the article of “Cheirsilpa A 2005” was deleted.

Acute nausea

| A+D | . | . | 1.16 (0.67; 2.00) | . | . | . | . | . | . | . | . |
| --- | --- | --- | --- | --- | --- | --- | --- | --- | --- | --- | --- |
| 0.91 (0.50; 1.64) | Do | 0.98 (0.85; 1.12) | . | 0.99 (0.68; 1.42) | . | . | . | . | . | . | . |
| 0.89 (0.50; 1.58) | 0.98 (0.86; 1.12) | G | 1.32 (1.06; 1.63) | 1.00 (0.93; 1.08) | . | . | . | 1.32 (0.96; 1.81) | . | 0.41 (0.23; 0.73) | . |
| 1.16 (0.67; 2.00) | 1.28 (1.01; 1.62) | 1.31 (1.07; 1.59) | G+D | . | 1.04 (0.91; 1.19) | . | 0.97 (0.84; 1.12) | . | . | . | . |
| 0.88 (0.49; 1.58) | 0.97 (0.84; 1.12) | 0.99 (0.93; 1.07) | 0.76 (0.62; 0.93) | O | 1.26 (0.74; 2.13) | 1.33 (0.80; 2.20) | . | 1.16 (0.72; 1.86) | . | 1.10 (0.78; 1.56) | . |
| 1.15 (0.66; 2.01) | 1.27 (0.99; 1.65) | 1.30 (1.04; 1.62) | 0.99 (0.89; 1.11) | 1.31 (1.04; 1.64) | O+D | . | 1.14 (0.93; 1.38) | . | . | . | . |
| 1.17 (0.54; 2.54) | 1.30 (0.77; 2.19) | 1.32 (0.79; 2.20) | 1.01 (0.59; 1.75) | 1.33 (0.80; 2.20) | 1.02 (0.59; 1.77) | P | . | . | . | . | . |
| 1.19 (0.68; 2.08) | 1.31 (1.01; 1.71) | 1.34 (1.06; 1.69) | 1.02 (0.90; 1.16) | 1.35 (1.06; 1.71) | 1.03 (0.90; 1.18) | 1.01 (0.58; 1.77) | P+D | . | . | . | . |
| 1.12 (0.59; 2.12) | 1.24 (0.92; 1.66) | 1.27 (0.97; 1.65) | 0.97 (0.70; 1.35) | 1.27 (0.97; 1.66) | 0.97 (0.69; 1.37) | 0.96 (0.54; 1.69) | 0.94 (0.67; 1.34) | R | 1.61 (1.16; 2.24) | . | . |
| 1.81 (0.88; 3.71) | 2.00 (1.29; 3.11) | 2.04 (1.34; 3.11) | 1.56 (0.98; 2.49) | 2.05 (1.34; 3.14) | 1.57 (0.97; 2.53) | 1.54 (0.80; 2.98) | 1.52 (0.94; 2.46) | 1.61 (1.16; 2.24) | R+D | . | . |
| 0.75 (0.39; 1.44) | 0.83 (0.60; 1.15) | 0.85 (0.63; 1.14) | 0.65 (0.45; 0.93) | 0.85 (0.63; 1.15) | 0.65 (0.45; 0.94) | 0.64 (0.36; 1.15) | 0.63 (0.43; 0.92) | 0.67 (0.45; 1.00) | 0.41 (0.25; 0.70) | T | 1.85 (1.25; 2.74) |
| 1.39 (0.65; 2.98) | 1.54 (0.92; 2.56) | 1.57 (0.96; 2.57) | 1.20 (0.70; 2.04) | 1.58 (0.96; 2.58) | 1.21 (0.70; 2.07) | 1.19 (0.59; 2.40) | 1.17 (0.68; 2.02) | 1.24 (0.71; 2.17) | 0.77 (0.40; 1.47) | 1.85 (1.25; 2.74) | T+D |

P-score

R+D 0.9568

T+D 0.7787

P+D 0.6796

G+D 0.6273

O+D 0.6124

P 0.6046

R 0.5789

A+D 0.4162

Do 0.2604

G 0.2182

O 0.2005

T 0.0665

Acute vomiting

| Do | 0.96 (0.79; 1.15) | . | 0.99 (0.84; 1.15) | . | . | . | . | . | . | . |
| --- | --- | --- | --- | --- | --- | --- | --- | --- | --- | --- |
| 0.93 (0.82; 1.06) | G | 1.57 (1.28; 1.91) | 1.09 (0.99; 1.19) | . | 1.61 (0.94; 2.77) | . | 1.32 (0.93; 1.87) | . | 0.91 (0.65; 1.27) | . |
| 1.46 (1.18; 1.82) | 1.58 (1.32; 1.88) | G+D | . | 1.06 (0.88; 1.28) | . | 1.05 (0.81; 1.36) | . | 0.57 (0.25; 1.28) | . | . |
| 1.01 (0.89; 1.14) | 1.08 (0.99; 1.18) | 0.69 (0.57; 0.83) | O | 1.28 (0.88; 1.85) | 1.98 (0.60; 6.53) | . | 1.06 (0.74; 1.52) | . | 0.78 (0.60; 1.03) | . |
| 1.42 (1.11; 1.81) | 1.53 (1.24; 1.89) | 0.97 (0.83; 1.13) | 1.41 (1.14; 1.75) | O+D | . | 1.32 (1.08; 1.60) | . | . | . | . |
| 1.57 (0.95; 2.62) | 1.69 (1.03; 2.77) | 1.07 (0.64; 1.81) | 1.56 (0.95; 2.57) | 1.11 (0.65; 1.89) | P | . | . | . | . | . |
| 1.74 (1.33; 2.28) | 1.87 (1.47; 2.39) | 1.19 (0.99; 1.43) | 1.73 (1.35; 2.22) | 1.23 (1.04; 1.45) | 1.11 (0.64; 1.92) | P+D | . | . | . | . |
| 1.09 (0.83; 1.42) | 1.17 (0.91; 1.49) | 0.74 (0.55; 0.99) | 1.08 (0.84; 1.38) | 0.76 (0.56; 1.05) | 0.69 (0.40; 1.20) | 0.62 (0.45; 0.87) | R | 1.58 (1.05; 2.38) | . | . |
| 1.49 (0.97; 2.29) | 1.60 (1.06; 2.42) | 1.01 (0.66; 1.56) | 1.48 (0.97; 2.24) | 1.05 (0.67; 1.64) | 0.94 (0.50; 1.80) | 0.85 (0.54; 1.36) | 1.37 (0.95; 1.98) | R+D | . | . |
| 0.79 (0.61; 1.03) | 0.85 (0.67; 1.08) | 0.54 (0.40; 0.73) | 0.79 (0.62; 1.00) | 0.56 (0.41; 0.77) | 0.50 (0.29; 0.87) | 0.46 (0.33; 0.64) | 0.73 (0.52; 1.02) | 0.53 (0.33; 0.86) | T | 1.99 (1.42; 2.79) |
| 1.58 (1.03; 2.42) | 1.70 (1.13; 2.57) | 1.08 (0.69; 1.69) | 1.57 (1.04; 2.37) | 1.11 (0.70; 1.77) | 1.01 (0.53; 1.91) | 0.91 (0.56; 1.46) | 1.46 (0.90; 2.35) | 1.06 (0.59; 1.91) | 1.99 (1.42; 2.79) | T+D |

P-score

P+D 0.9002

T+D 0.7643

P 0.7473

R+D 0.7072

G+D 0.6903

O+D 0.6396

R 0.3586

O 0.2845

Do 0.2657

G 0.1212

T 0.0211

Acute complete control

| G | 0.80 (0.61; 1.04) | 0.96 (0.89; 1.03) | . | . | . | . | . | 1.16 (0.92; 1.47) | . |
| --- | --- | --- | --- | --- | --- | --- | --- | --- | --- |
| 0.86 (0.74; 1.01) | G+D | . | 0.99 (0.92; 1.06) | . | 0.98 (0.91; 1.05) | . | 1.16 (0.89; 1.51) | . | . |
| 0.95 (0.88; 1.02) | 1.10 (0.95; 1.28) | O | 0.94 (0.80; 1.12) | 0.98 (0.91; 1.05) | . | . | . | 1.13 (0.89; 1.44) | . |
| 0.87 (0.74; 1.01) | 1.01 (0.95; 1.07) | 0.91 (0.79; 1.06) | O+D | . | 0.93 (0.84; 1.03) | . | . | . | . |
| 0.93 (0.83; 1.03) | 1.08 (0.91; 1.27) | 0.98 (0.91; 1.05) | 1.07 (0.91; 1.26) | P | . | . | . | . | . |
| 0.83 (0.71; 0.98) | 0.96 (0.91; 1.03) | 0.87 (0.75; 1.02) | 0.96 (0.89; 1.03) | 0.90 (0.75; 1.07) | P+D | . | . | . | . |
| 1.26 (0.88; 1.82) | 1.47 (1.06; 2.03) | 1.33 (0.93; 1.90) | 1.45 (1.04; 2.03) | 1.36 (0.94; 1.97) | 1.52 (1.09; 2.12) | R | 0.79 (0.65; 0.96) | . | . |
| 1.00 (0.74; 1.36) | 1.16 (0.89; 1.51) | 1.05 (0.78; 1.43) | 1.15 (0.88; 1.51) | 1.08 (0.79; 1.48) | 1.20 (0.92; 1.58) | 0.79 (0.65; 0.96) | R+D | . | . |
| 1.12 (0.90; 1.39) | 1.30 (1.00; 1.69) | 1.18 (0.95; 1.47) | 1.29 (0.99; 1.67) | 1.21 (0.96; 1.52) | 1.35 (1.03; 1.76) | 0.89 (0.58; 1.35) | 1.12 (0.77; 1.62) | T | 0.65 (0.53; 0.80) |
| 0.73 (0.54; 0.98) | 0.84 (0.60; 1.18) | 0.77 (0.57; 1.03) | 0.84 (0.60; 1.17) | 0.78 (0.57; 1.07) | 0.87 (0.62; 1.23) | 0.58 (0.36; 0.92) | 0.73 (0.47; 1.11) | 0.65 (0.53; 0.80) | T+D |

P-score

T+D 0.9189

P+D 0.8558

G+D 0.7104

O+D 0.6764

P 0.5371

O 0.4410

R+D 0.3704

G 0.2805

T 0.1484

R 0.0610

Delayed nausea: The network disconnection has made a comparison impossible.

Delayed vomiting: The network disconnection has made a comparison impossible.

Delayed complete control

| G | 1.12 (0.75; 1.67) | . | . | . | . | . |
| --- | --- | --- | --- | --- | --- | --- |
| 1.12 (0.75; 1.67) | G+D | . | 0.94 (0.75; 1.17) | . | 0.80 (0.65; 0.99) | 0.92 (0.58; 1.47) |
| 1.82 (1.09; 3.06) | 1.63 (1.18; 2.27) | O | 0.61 (0.47; 0.81) | 0.70 (0.49; 0.99) | . | . |
| 1.12 (0.72; 1.73) | 1.00 (0.84; 1.20) | 0.61 (0.47; 0.81) | O+D | . | 0.70 (0.56; 0.88) | . |
| 1.27 (0.68; 2.37) | 1.14 (0.71; 1.84) | 0.70 (0.49; 0.99) | 1.14 (0.73; 1.77) | P | . | . |
| 0.84 (0.54; 1.30) | 0.75 (0.63; 0.90) | 0.46 (0.33; 0.64) | 0.75 (0.63; 0.90) | 0.66 (0.41; 1.06) | P+D | . |
| 1.03 (0.56; 1.90) | 0.92 (0.58; 1.47) | 0.57 (0.32; 1.00) | 0.92 (0.56; 1.51) | 0.81 (0.42; 1.58) | 1.23 (0.75; 2.01) | R+D |

P-score

P+D 0.9215

G 0.6529

R+D 0.6053

O+D 0.4811

G+D 0.4799

P 0.3494

O 0.0100

5、The outcomes after the article of “Dong XR 2011” was deleted.

Acute nausea

| A+D | . | . | 1.16 (0.67; 2.00) | . | . | . | . | . | . | . | . |
| --- | --- | --- | --- | --- | --- | --- | --- | --- | --- | --- | --- |
| 0.89 (0.49; 1.61) | Do | 0.98 (0.85; 1.12) | . | 0.99 (0.68; 1.42) | . | . | . | . | . | . | . |
| 0.87 (0.49; 1.55) | 0.98 (0.86; 1.12) | G | 1.32 (1.06; 1.63) | 1.00 (0.93; 1.08) | . | . | . | 1.32 (0.96; 1.81) | . | 0.41 (0.23; 0.73) | . |
| 1.16 (0.67; 2.00) | 1.30 (1.03; 1.64) | 1.33 (1.10; 1.61) | G+D | . | 1.04 (0.91; 1.19) | . | 0.97 (0.84; 1.12) | . | 1.22 (0.66; 2.23) | . | . |
| 0.87 (0.49; 1.55) | 0.97 (0.84; 1.12) | 0.99 (0.93; 1.06) | 0.75 (0.61; 0.91) | O | 1.26 (0.74; 2.13) | 1.50 (0.83; 2.71) | . | 1.16 (0.72; 1.86) | . | 1.10 (0.78; 1.56) | . |
| 1.15 (0.66; 2.01) | 1.29 (1.01; 1.66) | 1.32 (1.07; 1.64) | 0.99 (0.89; 1.11) | 1.33 (1.07; 1.66) | O+D | . | 1.14 (0.93; 1.38) | . | . | . | . |
| 1.30 (0.57; 2.97) | 1.46 (0.79; 2.68) | 1.49 (0.82; 2.70) | 1.12 (0.60; 2.09) | 1.50 (0.83; 2.71) | 1.13 (0.60; 2.12) | P | . | . | . | . | . |
| 1.19 (0.68; 2.08) | 1.33 (1.03; 1.73) | 1.36 (1.09; 1.71) | 1.02 (0.91; 1.16) | 1.37 (1.09; 1.73) | 1.03 (0.90; 1.18) | 0.92 (0.49; 1.73) | P+D | . | . | . | . |
| 1.07 (0.58; 1.99) | 1.20 (0.91; 1.59) | 1.23 (0.96; 1.57) | 0.92 (0.69; 1.24) | 1.24 (0.96; 1.59) | 0.93 (0.68; 1.27) | 0.83 (0.43; 1.57) | 0.90 (0.66; 1.24) | R | 1.61 (1.16; 2.24) | . | . |
| 1.65 (0.86; 3.19) | 1.85 (1.27; 2.70) | 1.89 (1.33; 2.69) | 1.42 (0.98; 2.06) | 1.90 (1.33; 2.72) | 1.43 (0.98; 2.10) | 1.27 (0.64; 2.54) | 1.39 (0.94; 2.05) | 1.54 (1.14; 2.07) | R+D | . | . |
| 0.74 (0.39; 1.41) | 0.83 (0.60; 1.15) | 0.85 (0.63; 1.14) | 0.64 (0.45; 0.91) | 0.85 (0.63; 1.15) | 0.64 (0.44; 0.93) | 0.57 (0.29; 1.10) | 0.62 (0.43; 0.90) | 0.69 (0.47; 1.01) | 0.45 (0.28; 0.71) | T | 1.85 (1.25; 2.74) |
| 1.37 (0.64; 2.92) | 1.54 (0.92; 2.56) | 1.57 (0.96; 2.57) | 1.18 (0.70; 2.00) | 1.58 (0.97; 2.58) | 1.19 (0.69; 2.03) | 1.06 (0.49; 2.28) | 1.15 (0.67; 1.98) | 1.28 (0.74; 2.21) | 0.83 (0.45; 1.52) | 1.85 (1.25; 2.74) | T+D |

P-score

R+D 0.9361

T+D 0.7697

P 0.6982

P+D 0.6815

G+D 0.6312

O+D 0.6151

R 0.5210

A+D 0.4240

Do 0.2533

G 0.2127

O 0.1940

T 0.0632

Acute vomiting

| Do | 0.96 (0.79; 1.15) | . | 0.99 (0.84; 1.15) | . | . | . | . | . | . | . |
| --- | --- | --- | --- | --- | --- | --- | --- | --- | --- | --- |
| 0.93 (0.82; 1.06) | G | 1.57 (1.28; 1.91) | 1.09 (0.99; 1.19) | . | 1.61 (0.94; 2.77) | . | 1.32 (0.93; 1.87) | . | 0.91 (0.65; 1.27) | . |
| 1.46 (1.18; 1.80) | 1.57 (1.32; 1.87) | G+D | . | 1.06 (0.88; 1.28) | . | 1.05 (0.81; 1.36) | . | 0.84 (0.48; 1.47) | . | . |
| 1.01 (0.89; 1.14) | 1.08 (1.00; 1.18) | 0.69 (0.57; 0.83) | O | 1.28 (0.88; 1.85) | 1.99 (0.50; 7.90) | . | 1.06 (0.74; 1.52) | . | 0.78 (0.60; 1.03) | . |
| 1.41 (1.11; 1.80) | 1.52 (1.23; 1.88) | 0.97 (0.83; 1.13) | 1.40 (1.13; 1.74) | O+D | . | 1.32 (1.08; 1.60) | . | . | . | . |
| 1.56 (0.93; 2.62) | 1.68 (1.01; 2.78) | 1.07 (0.63; 1.82) | 1.55 (0.93; 2.58) | 1.10 (0.64; 1.90) | P | . | . | . | . | . |
| 1.73 (1.32; 2.27) | 1.87 (1.47; 2.38) | 1.19 (0.99; 1.43) | 1.72 (1.35; 2.21) | 1.23 (1.04; 1.45) | 1.11 (0.64; 1.95) | P+D | . | . | . | . |
| 1.10 (0.84; 1.43) | 1.18 (0.93; 1.50) | 0.75 (0.57; 1.00) | 1.09 (0.86; 1.39) | 0.78 (0.57; 1.05) | 0.70 (0.40; 1.23) | 0.63 (0.46; 0.88) | R | 1.58 (1.05; 2.38) | . | . |
| 1.54 (1.04; 2.27) | 1.65 (1.14; 2.40) | 1.05 (0.72; 1.54) | 1.53 (1.05; 2.22) | 1.09 (0.73; 1.62) | 0.99 (0.53; 1.85) | 0.89 (0.59; 1.34) | 1.40 (1.00; 1.98) | R+D | . | . |
| 0.79 (0.61; 1.03) | 0.85 (0.67; 1.09) | 0.54 (0.41; 0.73) | 0.79 (0.62; 1.00) | 0.56 (0.41; 0.77) | 0.51 (0.29; 0.89) | 0.46 (0.33; 0.64) | 0.72 (0.52; 1.01) | 0.52 (0.33; 0.80) | T | 1.99 (1.42; 2.79) |
| 1.58 (1.03; 2.42) | 1.70 (1.13; 2.57) | 1.09 (0.69; 1.70) | 1.57 (1.04; 2.37) | 1.12 (0.71; 1.77) | 1.02 (0.53; 1.95) | 0.91 (0.57; 1.47) | 1.44 (0.90; 2.32) | 1.03 (0.59; 1.79) | 1.99 (1.42; 2.79) | T+D |

P-score

P+D 0.8969

T+D 0.7631

R+D 0.7434

P 0.7347

G+D 0.6817

O+D 0.6321

R 0.3666

O 0.2807

Do 0.2617

G 0.1186

T 0.0204

Acute complete control

| G | 0.80 (0.61; 1.04) | 0.96 (0.89; 1.03) | . | . | . | . | . | 1.16 (0.92; 1.47) | . |
| --- | --- | --- | --- | --- | --- | --- | --- | --- | --- |
| 0.86 (0.74; 1.01) | G+D | . | 0.99 (0.92; 1.06) | . | 0.98 (0.91; 1.05) | . | 1.05 (0.87; 1.27) | . | . |
| 0.95 (0.88; 1.02) | 1.10 (0.95; 1.28) | O | 0.94 (0.80; 1.12) | 0.96 (0.88; 1.05) | . | . | . | 1.13 (0.89; 1.44) | . |
| 0.87 (0.74; 1.01) | 1.01 (0.95; 1.07) | 0.91 (0.79; 1.06) | O+D | . | 0.93 (0.84; 1.03) | . | . | . | . |
| 0.92 (0.81; 1.03) | 1.06 (0.89; 1.27) | 0.96 (0.88; 1.05) | 1.05 (0.89; 1.25) | P | . | . | . | . | . |
| 0.83 (0.71; 0.98) | 0.96 (0.91; 1.03) | 0.87 (0.75; 1.02) | 0.96 (0.89; 1.03) | 0.91 (0.76; 1.09) | P+D | . | . | . | . |
| 1.14 (0.83; 1.57) | 1.33 (1.01; 1.74) | 1.20 (0.88; 1.64) | 1.32 (1.00; 1.74) | 1.25 (0.90; 1.73) | 1.37 (1.04; 1.82) | R | 0.79 (0.65; 0.96) | . | . |
| 0.91 (0.71; 1.16) | 1.05 (0.87; 1.27) | 0.95 (0.75; 1.22) | 1.04 (0.85; 1.28) | 0.99 (0.76; 1.28) | 1.09 (0.89; 1.33) | 0.79 (0.65; 0.96) | R+D | . | . |
| 1.12 (0.90; 1.39) | 1.30 (1.00; 1.69) | 1.18 (0.95; 1.47) | 1.29 (0.99; 1.67) | 1.22 (0.97; 1.55) | 1.35 (1.03; 1.76) | 0.98 (0.67; 1.43) | 1.24 (0.89; 1.71) | T | 0.65 (0.53; 0.80) |
| 0.73 (0.54; 0.98) | 0.84 (0.60; 1.18) | 0.77 (0.57; 1.03) | 0.84 (0.60; 1.17) | 0.79 (0.58; 1.09) | 0.87 (0.62; 1.23) | 0.64 (0.41; 0.98) | 0.80 (0.55; 1.18) | 0.65 (0.53; 0.80) | T+D |

P-score

T+D 0.9095

P+D 0.8383

G+D 0.6841

O+D 0.6474

R+D 0.5372

P 0.5336

O 0.3973

G 0.2366

T 0.1089

R 0.1070

Delayed nausea

| Do | 1.99 (1.27; 3.12) | . | . | 0.91 (0.63; 1.33) | . | . | . | . | . | . | . |
| --- | --- | --- | --- | --- | --- | --- | --- | --- | --- | --- | --- |
| 1.99 (1.27; 3.12) | Do+D | . | . | . | . | . | . | . | . | . | . |
| 0.99 (0.57; 1.71) | 0.50 (0.25; 1.01) | G | 0.97 (0.66; 1.42) | 1.17 (0.67; 2.04) | . | . | . | . | . | . | . |
| 1.08 (0.64; 1.82) | 0.54 (0.27; 1.08) | 1.09 (0.78; 1.52) | G+D | . | 1.07 (0.75; 1.53) | . | 1.42 (1.08; 1.85) | . | 1.02 (0.63; 1.63) | . | . |
| 0.91 (0.63; 1.33) | 0.46 (0.26; 0.83) | 0.92 (0.62; 1.37) | 0.85 (0.59; 1.21) | O | 1.43 (1.03; 2.00) | 1.34 (0.87; 2.06) | . | . | . | 1.21 (0.79; 1.85) | . |
| 1.20 (0.74; 1.94) | 0.60 (0.31; 1.17) | 1.21 (0.83; 1.76) | 1.11 (0.87; 1.42) | 1.31 (0.97; 1.78) | O+D | . | 1.37 (1.05; 1.79) | . | . | . | . |
| 1.22 (0.69; 2.17) | 0.61 (0.30; 1.27) | 1.23 (0.69; 2.21) | 1.13 (0.65; 1.98) | 1.34 (0.87; 2.06) | 1.02 (0.60; 1.72) | P | . | . | . | . | . |
| 1.59 (0.94; 2.67) | 0.80 (0.40; 1.59) | 1.60 (1.09; 2.35) | 1.47 (1.17; 1.84) | 1.74 (1.21; 2.49) | 1.32 (1.05; 1.66) | 1.30 (0.74; 2.27) | P+D | . | . | . | . |
| 0.72 (0.32; 1.60) | 0.36 (0.14; 0.90) | 0.72 (0.36; 1.45) | 0.66 (0.36; 1.23) | 0.78 (0.38; 1.60) | 0.60 (0.31; 1.16) | 0.58 (0.25; 1.34) | 0.45 (0.23; 0.87) | R | 1.53 (1.03; 2.27) | . | . |
| 1.10 (0.54; 2.22) | 0.55 (0.24; 1.27) | 1.11 (0.62; 1.97) | 1.02 (0.63; 1.63) | 1.20 (0.66; 2.18) | 0.91 (0.54; 1.56) | 0.90 (0.43; 1.87) | 0.69 (0.41; 1.17) | 1.53 (1.03; 2.27) | R+D | . | . |
| 1.10 (0.62; 1.95) | 0.55 (0.27; 1.14) | 1.11 (0.62; 1.99) | 1.02 (0.59; 1.78) | 1.21 (0.79; 1.85) | 0.92 (0.55; 1.55) | 0.90 (0.49; 1.65) | 0.70 (0.40; 1.22) | 1.54 (0.67; 3.53) | 1.01 (0.48; 2.09) | T | 1.49 (0.98; 2.24) |
| 1.64 (0.81; 3.30) | 0.82 (0.36; 1.90) | 1.65 (0.81; 3.37) | 1.52 (0.76; 3.03) | 1.79 (0.99; 3.24) | 1.37 (0.70; 2.65) | 1.34 (0.64; 2.78) | 1.03 (0.52; 2.07) | 2.29 (0.91; 5.78) | 1.49 (0.65; 3.46) | 1.49 (0.98; 2.24) | T+D |

P-score

Do+D 0.9118

P+D 0.8442

T+D 0.8097

O+D 0.5715

P 0.5688

R+D 0.4599

T 0.4443

G+D 0.4245

Do 0.3403

G 0.3208

O 0.2012

R 0.1030

Delayed vomiting

| Do | 2.33 (1.59; 3.42) | . | . | 1.27 (0.82; 1.97) | . | . | . | . | . | . | . |
| --- | --- | --- | --- | --- | --- | --- | --- | --- | --- | --- | --- |
| 2.33 (1.59; 3.42) | Do+D | . | . | . | . | . | . | . | . | . | . |
| 1.27 (0.76; 2.12) | 0.54 (0.29; 1.03) | G | 0.93 (0.64; 1.36) | 1.09 (0.78; 1.52) | . | 1.12 (0.75; 1.66) | . | . | . | 1.14 (0.68; 1.91) | . |
| 1.32 (0.77; 2.26) | 0.57 (0.29; 1.09) | 1.04 (0.77; 1.40) | G+D | . | 0.99 (0.72; 1.36) | . | 1.54 (1.19; 2.00) | . | 0.97 (0.56; 1.69) | . | . |
| 1.27 (0.82; 1.97) | 0.54 (0.30; 0.97) | 1.00 (0.77; 1.30) | 0.96 (0.71; 1.31) | O | 1.27 (0.91; 1.79) | 1.30 (0.61; 2.77) | . | . | . | 0.97 (0.73; 1.30) | . |
| 1.48 (0.87; 2.49) | 0.63 (0.33; 1.21) | 1.16 (0.85; 1.59) | 1.12 (0.91; 1.38) | 1.16 (0.87; 1.55) | O+D | . | 1.33 (1.12; 1.57) | . | . | . | . |
| 1.46 (0.80; 2.67) | 0.63 (0.31; 1.28) | 1.15 (0.81; 1.65) | 1.11 (0.71; 1.75) | 1.15 (0.77; 1.74) | 0.99 (0.63; 1.56) | P | . | . | . | . | . |
| 1.98 (1.16; 3.39) | 0.85 (0.44; 1.64) | 1.56 (1.13; 2.16) | 1.50 (1.23; 1.84) | 1.56 (1.14; 2.13) | 1.34 (1.15; 1.56) | 1.35 (0.85; 2.16) | P+D | . | . | . | . |
| 0.79 (0.34; 1.85) | 0.34 (0.13; 0.86) | 0.62 (0.30; 1.28) | 0.60 (0.31; 1.16) | 0.62 (0.30; 1.29) | 0.53 (0.27; 1.07) | 0.54 (0.24; 1.20) | 0.40 (0.20; 0.79) | R | 1.63 (1.14; 2.33) | . | . |
| 1.28 (0.59; 2.77) | 0.55 (0.23; 1.30) | 1.01 (0.54; 1.90) | 0.97 (0.56; 1.69) | 1.01 (0.54; 1.91) | 0.87 (0.48; 1.57) | 0.88 (0.43; 1.79) | 0.65 (0.36; 1.17) | 1.63 (1.14; 2.33) | R+D | . | . |
| 1.27 (0.76; 2.14) | 0.55 (0.29; 1.04) | 1.00 (0.71; 1.42) | 0.96 (0.65; 1.44) | 1.00 (0.76; 1.33) | 0.86 (0.59; 1.27) | 0.87 (0.54; 1.39) | 0.64 (0.43; 0.96) | 1.61 (0.75; 3.48) | 0.99 (0.50; 1.96) | T | 1.58 (1.13; 2.20) |
| 2.01 (1.08; 3.73) | 0.86 (0.42; 1.78) | 1.58 (0.98; 2.56) | 1.52 (0.90; 2.56) | 1.58 (1.02; 2.45) | 1.36 (0.82; 2.27) | 1.37 (0.77; 2.45) | 1.01 (0.60; 1.71) | 2.54 (1.10; 5.89) | 1.56 (0.73; 3.34) | 1.58 (1.13; 2.20) | T+D |

P-score

Do+D 0.9030

P+D 0.8714

T+D 0.8489

O+D 0.5994

P 0.5665

R+D 0.4271

G+D 0.4235

T 0.3818

O 0.3698

G 0.3693

Do 0.1661

R 0.0731

Delayed complete control

| G | 1.12 (0.75; 1.67) | . | . | . | . |
| --- | --- | --- | --- | --- | --- |
| 1.12 (0.75; 1.67) | G+D | . | 0.94 (0.75; 1.17) | 0.80 (0.65; 0.99) | 0.92 (0.58; 1.47) |
| 1.82 (1.09; 3.06) | 1.63 (1.18; 2.27) | O | 0.61 (0.47; 0.81) | . | . |
| 1.12 (0.72; 1.73) | 1.00 (0.84; 1.20) | 0.61 (0.47; 0.81) | O+D | 0.70 (0.56; 0.88) | . |
| 0.84 (0.54; 1.30) | 0.75 (0.63; 0.90) | 0.46 (0.33; 0.64) | 0.75 (0.63; 0.90) | P+D | . |
| 1.03 (0.56; 1.90) | 0.92 (0.58; 1.47) | 0.57 (0.32; 1.00) | 0.92 (0.56; 1.51) | 1.23 (0.75; 2.01) | R+D |

P-score

P+D 0.9147

G 0.6284

R+D 0.5800

G+D 0.4350

O+D 0.4343

O 0.0077

6、The outcomes after the article of “Fauser A.A. 2000” was deleted.

Delayed nausea

| Do | . | . | 0.91 (0.63; 1.31) | . | . | . | . | . | . | . |
| --- | --- | --- | --- | --- | --- | --- | --- | --- | --- | --- |
| 1.00 (0.59; 1.69) | G | 0.97 (0.67; 1.40) | 1.17 (0.68; 2.02) | . | . | . | . | . | . | . |
| 1.08 (0.65; 1.79) | 1.09 (0.79; 1.50) | G+D | . | 1.07 (0.76; 1.51) | . | 1.41 (1.09; 1.83) | . | 1.02 (0.64; 1.61) | . | . |
| 0.91 (0.63; 1.31) | 0.92 (0.62; 1.35) | 0.84 (0.60; 1.19) | O | 1.43 (1.03; 1.98) | 1.42 (1.04; 1.95) | . | . | . | 1.21 (0.80; 1.83) | . |
| 1.20 (0.75; 1.91) | 1.20 (0.84; 1.73) | 1.11 (0.87; 1.40) | 1.31 (0.98; 1.76) | O+D | . | 1.37 (1.06; 1.78) | . | . | . | . |
| 1.30 (0.80; 2.10) | 1.30 (0.79; 2.15) | 1.20 (0.75; 1.92) | 1.42 (1.04; 1.95) | 1.09 (0.70; 1.67) | P | . | . | . | . | . |
| 1.58 (0.95; 2.62) | 1.59 (1.09; 2.30) | 1.46 (1.18; 1.82) | 1.73 (1.22; 2.46) | 1.32 (1.06; 1.64) | 1.22 (0.76; 1.95) | P+D | . | . | . | . |
| 0.72 (0.33; 1.57) | 0.72 (0.36; 1.42) | 0.66 (0.36; 1.21) | 0.78 (0.39; 1.57) | 0.60 (0.31; 1.14) | 0.55 (0.26; 1.18) | 0.45 (0.24; 0.86) | R | 1.53 (1.05; 2.24) | . | . |
| 1.10 (0.55; 2.18) | 1.10 (0.63; 1.94) | 1.02 (0.64; 1.61) | 1.20 (0.67; 2.15) | 0.92 (0.55; 1.54) | 0.84 (0.44; 1.63) | 0.69 (0.42; 1.16) | 1.53 (1.05; 2.24) | R+D | . | . |
| 1.10 (0.64; 1.91) | 1.11 (0.63; 1.95) | 1.02 (0.59; 1.75) | 1.21 (0.80; 1.83) | 0.92 (0.55; 1.53) | 0.85 (0.50; 1.43) | 0.70 (0.41; 1.20) | 1.54 (0.69; 3.45) | 1.00 (0.49; 2.05) | T | 1.49 (1.00; 2.22) |
| 1.64 (0.83; 3.23) | 1.64 (0.82; 3.29) | 1.51 (0.77; 2.96) | 1.79 (1.01; 3.19) | 1.37 (0.72; 2.61) | 1.26 (0.65; 2.43) | 1.04 (0.53; 2.03) | 2.29 (0.93; 5.63) | 1.49 (0.66; 3.37) | 1.49 (1.00; 2.22) | T+D |

P-score

P+D 0.9013

T+D 0.8601

P 0.6932

O+D 0.6103

R+D 0.4911

T 0.4735

G+D 0.4535

Do 0.3616

G 0.3430

O 0.2089

R 0.1035

Delayed vomiting

| Do | 2.33 (1.54; 3.53) | . | . | 1.27 (0.80; 2.02) | . | . | . | . | . | . | . |
| --- | --- | --- | --- | --- | --- | --- | --- | --- | --- | --- | --- |
| 2.33 (1.54; 3.53) | Do+D | . | . | . | . | . | . | . | . | . | . |
| 1.36 (0.80; 2.34) | 0.59 (0.30; 1.15) | G | 0.93 (0.62; 1.40) | 1.09 (0.77; 1.55) | . | 1.12 (0.73; 1.71) | . | . | . | 1.14 (0.67; 1.96) | . |
| 1.37 (0.77; 2.42) | 0.59 (0.29; 1.19) | 1.00 (0.72; 1.38) | G+D | . | 0.99 (0.69; 1.41) | . | 1.60 (1.20; 2.13) | . | 0.97 (0.55; 1.73) | . | . |
| 1.27 (0.80; 2.02) | 0.54 (0.29; 1.01) | 0.93 (0.71; 1.21) | 0.93 (0.67; 1.29) | O | 1.28 (0.89; 1.84) | 1.88 (1.17; 3.02) | . | . | . | 0.98 (0.71; 1.33) | . |
| 1.54 (0.88; 2.68) | 0.66 (0.33; 1.32) | 1.13 (0.81; 1.57) | 1.13 (0.90; 1.42) | 1.21 (0.89; 1.64) | O+D | . | 1.33 (1.10; 1.60) | . | . | . | . |
| 1.86 (1.04; 3.34) | 0.80 (0.39; 1.63) | 1.36 (0.97; 1.92) | 1.36 (0.88; 2.11) | 1.47 (1.03; 2.08) | 1.21 (0.79; 1.87) | P | . | . | . | . | . |
| 2.08 (1.17; 3.69) | 0.89 (0.44; 1.81) | 1.52 (1.07; 2.16) | 1.52 (1.22; 1.91) | 1.64 (1.17; 2.29) | 1.35 (1.14; 1.60) | 1.12 (0.71; 1.75) | P+D | . | . | . | . |
| 0.82 (0.33; 2.01) | 0.35 (0.13; 0.94) | 0.60 (0.28; 1.29) | 0.60 (0.30; 1.20) | 0.64 (0.30; 1.39) | 0.53 (0.26; 1.11) | 0.44 (0.19; 1.00) | 0.39 (0.19; 0.82) | R | 1.63 (1.10; 2.41) | . | . |
| 1.33 (0.59; 2.99) | 0.57 (0.23; 1.41) | 0.97 (0.50; 1.88) | 0.97 (0.55; 1.73) | 1.05 (0.54; 2.03) | 0.86 (0.47; 1.60) | 0.71 (0.35; 1.47) | 0.64 (0.34; 1.18) | 1.63 (1.10; 2.41) | R+D | . | . |
| 1.30 (0.74; 2.26) | 0.56 (0.28; 1.11) | 0.95 (0.66; 1.37) | 0.95 (0.62; 1.46) | 1.02 (0.75; 1.38) | 0.84 (0.56; 1.28) | 0.70 (0.45; 1.09) | 0.62 (0.40; 0.96) | 1.59 (0.70; 3.60) | 0.98 (0.48; 2.00) | T | 1.59 (1.10; 2.28) |
| 2.06 (1.06; 4.00) | 0.88 (0.40; 1.93) | 1.51 (0.90; 2.52) | 1.51 (0.86; 2.64) | 1.62 (1.01; 2.60) | 1.34 (0.77; 2.32) | 1.10 (0.62; 1.96) | 0.99 (0.56; 1.74) | 2.52 (1.03; 6.16) | 1.55 (0.69; 3.46) | 1.59 (1.10; 2.28) | T+D |

P-score

Do+D 0.8660

P+D 0.8598

T+D 0.8178

P 0.7585

O+D 0.5736

R+D 0.4175

G 0.4119

G+D 0.4059

T 0.3497

O 0.3100

Do 0.1485

R 0.0807

7、The outcomes after the article of “Garcia del Muro X 1998” was deleted.

Acute nausea

| A+D | . | . | 1.16 (0.67; 2.00) | . | . | . | . | . | . | . |
| --- | --- | --- | --- | --- | --- | --- | --- | --- | --- | --- |
| 0.89 (0.49; 1.61) | Do | 0.98 (0.85; 1.12) | . | 0.99 (0.68; 1.42) | . | . | . | . | . | . |
| 0.87 (0.49; 1.55) | 0.98 (0.86; 1.11) | G | 1.32 (1.07; 1.63) | 1.00 (0.93; 1.07) | . | . | . | 1.32 (0.97; 1.81) | . | 0.41 (0.23; 0.73) |
| 1.16 (0.67; 2.00) | 1.30 (1.04; 1.64) | 1.33 (1.10; 1.61) | G+D | . | 1.04 (0.91; 1.19) | . | 0.97 (0.84; 1.12) | . | 1.22 (0.66; 2.23) | . |
| 0.87 (0.49; 1.55) | 0.97 (0.85; 1.12) | 0.99 (0.93; 1.06) | 0.75 (0.61; 0.91) | O | 1.26 (0.74; 2.12) | 1.33 (0.80; 2.20) | . | 1.16 (0.72; 1.85) | . | 1.10 (0.78; 1.56) |
| 1.15 (0.66; 2.01) | 1.29 (1.01; 1.66) | 1.32 (1.07; 1.63) | 0.99 (0.89; 1.11) | 1.33 (1.07; 1.66) | O+D | . | 1.14 (0.93; 1.38) | . | . | . |
| 1.15 (0.54; 2.49) | 1.30 (0.77; 2.19) | 1.32 (0.80; 2.20) | 0.99 (0.58; 1.71) | 1.33 (0.80; 2.20) | 1.00 (0.58; 1.73) | P | . | . | . | . |
| 1.19 (0.68; 2.07) | 1.33 (1.03; 1.72) | 1.36 (1.09; 1.70) | 1.02 (0.91; 1.15) | 1.37 (1.09; 1.72) | 1.03 (0.90; 1.18) | 1.03 (0.59; 1.79) | P+D | . | . | . |
| 1.07 (0.58; 1.99) | 1.20 (0.91; 1.59) | 1.23 (0.96; 1.57) | 0.92 (0.69; 1.24) | 1.24 (0.96; 1.59) | 0.93 (0.68; 1.27) | 0.93 (0.53; 1.63) | 0.90 (0.66; 1.24) | R | 1.61 (1.16; 2.24) | . |
| 1.65 (0.86; 3.18) | 1.85 (1.28; 2.69) | 1.89 (1.33; 2.69) | 1.42 (0.99; 2.06) | 1.90 (1.34; 2.71) | 1.43 (0.98; 2.10) | 1.43 (0.77; 2.65) | 1.39 (0.95; 2.05) | 1.54 (1.14; 2.07) | R+D | . |
| 0.74 (0.39; 1.41) | 0.83 (0.60; 1.15) | 0.85 (0.63; 1.14) | 0.64 (0.45; 0.91) | 0.85 (0.63; 1.15) | 0.64 (0.44; 0.93) | 0.64 (0.36; 1.15) | 0.62 (0.43; 0.90) | 0.69 (0.47; 1.01) | 0.45 (0.28; 0.71) | T |

P-score

R+D 0.9693

P+D 0.7326

G+D 0.6820

O+D 0.6653

P 0.6318

R 0.5662

A+D 0.4542

Do 0.2790

G 0.2345

O 0.2136

T 0.0716

Acute vomiting

| A+D | . | . | 1.16 (0.67; 2.00) | . | . | . | . | . | . | . |
| --- | --- | --- | --- | --- | --- | --- | --- | --- | --- | --- |
| 0.89 (0.49; 1.61) | Do | 0.98 (0.85; 1.12) | . | 0.99 (0.68; 1.42) | . | . | . | . | . | . |
| 0.87 (0.49; 1.55) | 0.98 (0.86; 1.11) | G | 1.32 (1.07; 1.63) | 1.00 (0.93; 1.07) | . | . | . | 1.32 (0.97; 1.81) | . | 0.41 (0.23; 0.73) |
| 1.16 (0.67; 2.00) | 1.30 (1.04; 1.64) | 1.33 (1.10; 1.61) | G+D | . | 1.04 (0.91; 1.19) | . | 0.97 (0.84; 1.12) | . | 1.22 (0.66; 2.23) | . |
| 0.87 (0.49; 1.55) | 0.97 (0.85; 1.12) | 0.99 (0.93; 1.06) | 0.75 (0.61; 0.91) | O | 1.26 (0.74; 2.12) | 1.33 (0.80; 2.20) | . | 1.16 (0.72; 1.85) | . | 1.10 (0.78; 1.56) |
| 1.15 (0.66; 2.01) | 1.29 (1.01; 1.66) | 1.32 (1.07; 1.63) | 0.99 (0.89; 1.11) | 1.33 (1.07; 1.66) | O+D | . | 1.14 (0.93; 1.38) | . | . | . |
| 1.15 (0.54; 2.49) | 1.30 (0.77; 2.19) | 1.32 (0.80; 2.20) | 0.99 (0.58; 1.71) | 1.33 (0.80; 2.20) | 1.00 (0.58; 1.73) | P | . | . | . | . |
| 1.19 (0.68; 2.07) | 1.33 (1.03; 1.72) | 1.36 (1.09; 1.70) | 1.02 (0.91; 1.15) | 1.37 (1.09; 1.72) | 1.03 (0.90; 1.18) | 1.03 (0.59; 1.79) | P+D | . | . | . |
| 1.07 (0.58; 1.99) | 1.20 (0.91; 1.59) | 1.23 (0.96; 1.57) | 0.92 (0.69; 1.24) | 1.24 (0.96; 1.59) | 0.93 (0.68; 1.27) | 0.93 (0.53; 1.63) | 0.90 (0.66; 1.24) | R | 1.61 (1.16; 2.24) | . |
| 1.65 (0.86; 3.18) | 1.85 (1.28; 2.69) | 1.89 (1.33; 2.69) | 1.42 (0.99; 2.06) | 1.90 (1.34; 2.71) | 1.43 (0.98; 2.10) | 1.43 (0.77; 2.65) | 1.39 (0.95; 2.05) | 1.54 (1.14; 2.07) | R+D | . |
| 0.74 (0.39; 1.41) | 0.83 (0.60; 1.15) | 0.85 (0.63; 1.14) | 0.64 (0.45; 0.91) | 0.85 (0.63; 1.15) | 0.64 (0.44; 0.93) | 0.64 (0.36; 1.15) | 0.62 (0.43; 0.90) | 0.69 (0.47; 1.01) | 0.45 (0.28; 0.71) | T |

P-score

R+D 0.9693

P+D 0.7326

G+D 0.6820

O+D 0.6653

P 0.6318

R 0.5662

A+D 0.4542

Do 0.2790

G 0.2345

O 0.2136

T 0.0716

Acute complete control

| A+D | . | . | 1.16 (0.67; 2.00) | . | . | . | . | . | . | . |
| --- | --- | --- | --- | --- | --- | --- | --- | --- | --- | --- |
| 0.89 (0.49; 1.61) | Do | 0.98 (0.85; 1.12) | . | 0.99 (0.68; 1.42) | . | . | . | . | . | . |
| 0.87 (0.49; 1.55) | 0.98 (0.86; 1.11) | G | 1.32 (1.07; 1.63) | 1.00 (0.93; 1.07) | . | . | . | 1.32 (0.97; 1.81) | . | 0.41 (0.23; 0.73) |
| 1.16 (0.67; 2.00) | 1.30 (1.04; 1.64) | 1.33 (1.10; 1.61) | G+D | . | 1.04 (0.91; 1.19) | . | 0.97 (0.84; 1.12) | . | 1.22 (0.66; 2.23) | . |
| 0.87 (0.49; 1.55) | 0.97 (0.85; 1.12) | 0.99 (0.93; 1.06) | 0.75 (0.61; 0.91) | O | 1.26 (0.74; 2.12) | 1.33 (0.80; 2.20) | . | 1.16 (0.72; 1.85) | . | 1.10 (0.78; 1.56) |
| 1.15 (0.66; 2.01) | 1.29 (1.01; 1.66) | 1.32 (1.07; 1.63) | 0.99 (0.89; 1.11) | 1.33 (1.07; 1.66) | O+D | . | 1.14 (0.93; 1.38) | . | . | . |
| 1.15 (0.54; 2.49) | 1.30 (0.77; 2.19) | 1.32 (0.80; 2.20) | 0.99 (0.58; 1.71) | 1.33 (0.80; 2.20) | 1.00 (0.58; 1.73) | P | . | . | . | . |
| 1.19 (0.68; 2.07) | 1.33 (1.03; 1.72) | 1.36 (1.09; 1.70) | 1.02 (0.91; 1.15) | 1.37 (1.09; 1.72) | 1.03 (0.90; 1.18) | 1.03 (0.59; 1.79) | P+D | . | . | . |
| 1.07 (0.58; 1.99) | 1.20 (0.91; 1.59) | 1.23 (0.96; 1.57) | 0.92 (0.69; 1.24) | 1.24 (0.96; 1.59) | 0.93 (0.68; 1.27) | 0.93 (0.53; 1.63) | 0.90 (0.66; 1.24) | R | 1.61 (1.16; 2.24) | . |
| 1.65 (0.86; 3.18) | 1.85 (1.28; 2.69) | 1.89 (1.33; 2.69) | 1.42 (0.99; 2.06) | 1.90 (1.34; 2.71) | 1.43 (0.98; 2.10) | 1.43 (0.77; 2.65) | 1.39 (0.95; 2.05) | 1.54 (1.14; 2.07) | R+D | . |
| 0.74 (0.39; 1.41) | 0.83 (0.60; 1.15) | 0.85 (0.63; 1.14) | 0.64 (0.45; 0.91) | 0.85 (0.63; 1.15) | 0.64 (0.44; 0.93) | 0.64 (0.36; 1.15) | 0.62 (0.43; 0.90) | 0.69 (0.47; 1.01) | 0.45 (0.28; 0.71) | T |

P-score

T 0.9284

O 0.7864

G 0.7655

Do 0.7210

A+D 0.5458

R 0.4338

P 0.3682

O+D 0.3347

G+D 0.3180

P+D 0.2674

R+D 0.0307

Delayed nausea

| Do | 1.99 (1.28; 3.08) | . | . | 0.91 (0.63; 1.31) | . | . | . | . | . | . |
| --- | --- | --- | --- | --- | --- | --- | --- | --- | --- | --- |
| 1.99 (1.28; 3.08) | Do+D | . | . | . | . | . | . | . | . | . |
| 1.00 (0.59; 1.69) | 0.50 (0.25; 1.00) | G | 0.97 (0.67; 1.40) | 1.17 (0.68; 2.02) | . | . | . | . | . | . |
| 1.08 (0.65; 1.79) | 0.54 (0.28; 1.06) | 1.09 (0.79; 1.50) | G+D | . | 1.07 (0.76; 1.51) | . | 1.41 (1.09; 1.83) | . | 1.02 (0.64; 1.61) | . |
| 0.91 (0.63; 1.31) | 0.46 (0.26; 0.81) | 0.92 (0.62; 1.35) | 0.84 (0.60; 1.19) | O | 1.43 (1.03; 1.98) | 1.42 (1.04; 1.95) | . | . | . | 1.21 (0.80; 1.83) |
| 1.20 (0.75; 1.91) | 0.60 (0.32; 1.14) | 1.20 (0.84; 1.73) | 1.11 (0.87; 1.40) | 1.31 (0.98; 1.76) | O+D | . | 1.37 (1.06; 1.78) | . | . | . |
| 1.30 (0.80; 2.10) | 0.65 (0.34; 1.25) | 1.30 (0.79; 2.15) | 1.20 (0.75; 1.92) | 1.42 (1.04; 1.95) | 1.09 (0.70; 1.67) | P | . | . | . | . |
| 1.58 (0.95; 2.62) | 0.80 (0.41; 1.55) | 1.59 (1.09; 2.30) | 1.46 (1.18; 1.82) | 1.73 (1.22; 2.46) | 1.32 (1.06; 1.64) | 1.22 (0.76; 1.95) | P+D | . | . | . |
| 0.72 (0.33; 1.57) | 0.36 (0.15; 0.88) | 0.72 (0.36; 1.42) | 0.66 (0.36; 1.21) | 0.78 (0.39; 1.57) | 0.60 (0.31; 1.14) | 0.55 (0.26; 1.18) | 0.45 (0.24; 0.86) | R | 1.53 (1.05; 2.24) | . |
| 1.10 (0.55; 2.18) | 0.55 (0.25; 1.24) | 1.10 (0.63; 1.94) | 1.02 (0.64; 1.61) | 1.20 (0.67; 2.15) | 0.92 (0.55; 1.54) | 0.84 (0.44; 1.63) | 0.69 (0.42; 1.16) | 1.53 (1.05; 2.24) | R+D | . |
| 1.10 (0.64; 1.91) | 0.55 (0.27; 1.12) | 1.11 (0.63; 1.95) | 1.02 (0.59; 1.75) | 1.21 (0.80; 1.83) | 0.92 (0.55; 1.53) | 0.85 (0.50; 1.43) | 0.70 (0.41; 1.20) | 1.54 (0.69; 3.45) | 1.00 (0.49; 2.05) | T |

P-score

Do+D 0.9384

P+D 0.8805

P 0.6787

O+D 0.5993

R+D 0.4818

T 0.4759

G+D 0.4459

Do 0.3540

G 0.3374

O 0.2070

R 0.1011

Delayed vomiting

| Do | 2.33 (1.53; 3.55) | . | . | 1.27 (0.79; 2.04) | . | . | . | . | . | . | . |
| --- | --- | --- | --- | --- | --- | --- | --- | --- | --- | --- | --- |
| 2.33 (1.53; 3.55) | Do+D | . | . | . | . | . | . | . | . | . | . |
| 1.36 (0.79; 2.35) | 0.58 (0.29; 1.16) | G | 0.93 (0.61; 1.41) | 1.09 (0.77; 1.56) | . | 1.12 (0.72; 1.72) | . | . | . | 1.14 (0.66; 1.97) | . |
| 1.36 (0.76; 2.43) | 0.58 (0.29; 1.19) | 1.00 (0.72; 1.39) | G+D | . | 0.99 (0.69; 1.42) | . | 1.61 (1.20; 2.15) | . | 0.97 (0.55; 1.74) | . | . |
| 1.27 (0.79; 2.04) | 0.54 (0.29; 1.02) | 0.93 (0.71; 1.22) | 0.93 (0.67; 1.30) | O | 1.28 (0.89; 1.85) | 1.87 (1.16; 3.02) | . | . | . | 0.98 (0.71; 1.34) | . |
| 1.54 (0.88; 2.71) | 0.66 (0.33; 1.33) | 1.13 (0.80; 1.58) | 1.13 (0.89; 1.43) | 1.21 (0.89; 1.65) | O+D | . | 1.33 (1.10; 1.60) | . | . | . | . |
| 1.86 (1.03; 3.36) | 0.80 (0.39; 1.65) | 1.37 (0.97; 1.93) | 1.37 (0.88; 2.13) | 1.47 (1.03; 2.09) | 1.21 (0.78; 1.87) | P | . | . | . | . | . |
| 2.09 (1.17; 3.73) | 0.89 (0.44; 1.83) | 1.53 (1.07; 2.18) | 1.53 (1.22; 1.92) | 1.64 (1.17; 2.30) | 1.35 (1.14; 1.61) | 1.12 (0.71; 1.76) | P+D | . | . | . | . |
| 0.82 (0.33; 2.03) | 0.35 (0.13; 0.95) | 0.60 (0.28; 1.30) | 0.60 (0.30; 1.21) | 0.64 (0.29; 1.40) | 0.53 (0.25; 1.11) | 0.44 (0.19; 1.00) | 0.39 (0.19; 0.82) | R | 1.63 (1.09; 2.42) | . | . |
| 1.33 (0.59; 3.01) | 0.57 (0.23; 1.43) | 0.97 (0.50; 1.89) | 0.97 (0.55; 1.74) | 1.05 (0.54; 2.04) | 0.86 (0.46; 1.61) | 0.71 (0.34; 1.48) | 0.64 (0.34; 1.19) | 1.63 (1.09; 2.42) | R+D | . | . |
| 1.30 (0.74; 2.28) | 0.56 (0.28; 1.12) | 0.95 (0.66; 1.37) | 0.95 (0.61; 1.47) | 1.02 (0.75; 1.39) | 0.84 (0.55; 1.28) | 0.70 (0.44; 1.09) | 0.62 (0.40; 0.97) | 1.59 (0.69; 3.64) | 0.98 (0.47; 2.02) | T | 2.20 (0.75; 6.45) |
| 2.85 (0.85; 9.60) | 1.22 (0.34; 4.42) | 2.09 (0.67; 6.52) | 2.09 (0.66; 6.68) | 2.25 (0.73; 6.88) | 1.85 (0.58; 5.88) | 1.53 (0.48; 4.91) | 1.37 (0.43; 4.37) | 3.50 (0.90; 13.58) | 2.15 (0.59; 7.86) | 2.20 (0.75; 6.45) | T+D |

P-score

T+D 0.8520

Do+D 0.8417

P+D 0.8404

P 0.7459

O+D 0.5743

R+D 0.4151

G 0.4150

G+D 0.4073

T 0.3570

O 0.3157

Do 0.1526

R 0.0832

8、The outcomes after the article of “Gebbia V 1994” was deleted.

Acute vomiting

| Do | 0.96 (0.79; 1.15) | . | 0.99 (0.84; 1.15) | . | . | . | . | . | . | . |
| --- | --- | --- | --- | --- | --- | --- | --- | --- | --- | --- |
| 0.93 (0.82; 1.06) | G | 1.57 (1.28; 1.91) | 1.09 (0.98; 1.20) | . | 1.61 (0.94; 2.77) | . | 1.32 (0.93; 1.87) | . | 0.91 (0.65; 1.27) | . |
| 1.46 (1.18; 1.81) | 1.57 (1.32; 1.87) | G+D | . | 1.06 (0.88; 1.28) | . | 1.05 (0.81; 1.36) | . | 0.84 (0.48; 1.47) | . | . |
| 1.01 (0.89; 1.14) | 1.08 (0.99; 1.18) | 0.69 (0.57; 0.83) | O | 1.28 (0.88; 1.85) | 1.98 (0.60; 6.53) | . | 1.06 (0.74; 1.53) | . | 0.78 (0.59; 1.03) | . |
| 1.41 (1.11; 1.80) | 1.52 (1.23; 1.88) | 0.97 (0.83; 1.13) | 1.40 (1.13; 1.74) | O+D | . | 1.32 (1.08; 1.60) | . | . | . | . |
| 1.57 (0.94; 2.62) | 1.69 (1.03; 2.78) | 1.08 (0.64; 1.82) | 1.56 (0.95; 2.57) | 1.11 (0.65; 1.90) | P | . | . | . | . | . |
| 1.73 (1.32; 2.27) | 1.87 (1.47; 2.38) | 1.19 (0.99; 1.43) | 1.72 (1.34; 2.21) | 1.23 (1.04; 1.45) | 1.10 (0.64; 1.91) | P+D | . | . | . | . |
| 1.10 (0.84; 1.43) | 1.18 (0.93; 1.50) | 0.75 (0.57; 1.00) | 1.09 (0.85; 1.39) | 0.78 (0.57; 1.05) | 0.70 (0.40; 1.21) | 0.63 (0.45; 0.88) | R | 1.58 (1.05; 2.38) | . | . |
| 1.54 (1.04; 2.27) | 1.65 (1.14; 2.40) | 1.05 (0.72; 1.54) | 1.53 (1.05; 2.22) | 1.09 (0.73; 1.62) | 0.98 (0.53; 1.81) | 0.89 (0.58; 1.34) | 1.40 (0.99; 1.98) | R+D | . | . |
| 0.79 (0.61; 1.03) | 0.85 (0.67; 1.09) | 0.54 (0.41; 0.73) | 0.79 (0.62; 1.00) | 0.56 (0.41; 0.77) | 0.50 (0.29; 0.87) | 0.46 (0.33; 0.64) | 0.72 (0.52; 1.01) | 0.52 (0.33; 0.80) | T | 1.99 (1.42; 2.79) |
| 1.58 (1.03; 2.43) | 1.70 (1.12; 2.58) | 1.09 (0.69; 1.70) | 1.57 (1.04; 2.37) | 1.12 (0.70; 1.78) | 1.01 (0.53; 1.91) | 0.91 (0.57; 1.47) | 1.44 (0.90; 2.32) | 1.03 (0.59; 1.79) | 1.99 (1.42; 2.79) | T+D |

P-score

P+D 0.8957

T+D 0.7618

P 0.7446

R+D 0.7417

G+D 0.6802

O+D 0.6305

R 0.3654

O 0.2799

Do 0.2607

G 0.1190

T 0.0205

Delayed nausea

| Do | 1.99 (1.30; 3.04) | . | . | 0.91 (0.65; 1.29) | . | . | . | . | . | . | . |
| --- | --- | --- | --- | --- | --- | --- | --- | --- | --- | --- | --- |
| 1.99 (1.30; 3.04) | Do+D | . | . | . | . | . | . | . | . | . | . |
| 1.28 (0.68; 2.41) | 0.64 (0.30; 1.38) | G | 0.97 (0.68; 1.38) | . | . | . | . | . | . | . | . |
| 1.24 (0.74; 2.10) | 0.63 (0.32; 1.23) | 0.97 (0.68; 1.38) | G+D | . | 1.07 (0.78; 1.48) | . | 1.40 (1.09; 1.79) | . | 1.02 (0.65; 1.59) | . | . |
| 0.91 (0.65; 1.29) | 0.46 (0.27; 0.79) | 0.71 (0.42; 1.21) | 0.73 (0.50; 1.09) | O | 1.43 (1.04; 1.95) | 1.42 (1.05; 1.94) | . | . | . | 1.21 (0.81; 1.80) | . |
| 1.30 (0.82; 2.08) | 0.66 (0.35; 1.23) | 1.02 (0.67; 1.55) | 1.05 (0.83; 1.33) | 1.43 (1.04; 1.95) | O+D | . | 1.37 (1.07; 1.75) | . | . | . | . |
| 1.30 (0.82; 2.07) | 0.65 (0.35; 1.23) | 1.01 (0.55; 1.87) | 1.05 (0.63; 1.73) | 1.42 (1.05; 1.94) | 1.00 (0.64; 1.55) | P | . | . | . | . | . |
| 1.76 (1.05; 2.94) | 0.89 (0.46; 1.73) | 1.38 (0.91; 2.07) | 1.42 (1.15; 1.75) | 1.93 (1.32; 2.82) | 1.35 (1.10; 1.67) | 1.36 (0.83; 2.21) | P+D | . | . | . | . |
| 0.82 (0.38; 1.80) | 0.41 (0.17; 1.01) | 0.64 (0.33; 1.27) | 0.66 (0.37; 1.18) | 0.90 (0.45; 1.82) | 0.63 (0.34; 1.18) | 0.63 (0.29; 1.36) | 0.47 (0.25; 0.87) | R | 1.53 (1.07; 2.21) | . | . |
| 1.26 (0.63; 2.52) | 0.63 (0.28; 1.43) | 0.99 (0.56; 1.74) | 1.02 (0.65; 1.59) | 1.38 (0.76; 2.52) | 0.97 (0.58; 1.61) | 0.97 (0.49; 1.90) | 0.72 (0.44; 1.18) | 1.53 (1.07; 2.21) | R+D | . | . |
| 1.10 (0.65; 1.87) | 0.55 (0.28; 1.09) | 0.86 (0.44; 1.67) | 0.89 (0.51; 1.55) | 1.21 (0.81; 1.80) | 0.85 (0.51; 1.41) | 0.85 (0.51; 1.40) | 0.63 (0.36; 1.08) | 1.34 (0.60; 3.00) | 0.87 (0.43; 1.79) | T | 1.49 (1.01; 2.18) |
| 1.64 (0.85; 3.15) | 0.82 (0.38; 1.80) | 1.28 (0.59; 2.75) | 1.32 (0.67; 2.60) | 1.79 (1.03; 3.12) | 1.26 (0.66; 2.38) | 1.26 (0.67; 2.38) | 0.93 (0.47; 1.82) | 1.99 (0.81; 4.86) | 1.30 (0.57; 2.93) | 1.49 (1.01; 2.18) | T+D |

P-score

Do+D 0.8830

P+D 0.8730

T+D 0.7572

O+D 0.5538

P 0.5486

G 0.5220

R+D 0.5139

G+D 0.4816

T 0.3525

Do 0.2528

O 0.1320

R 0.1297

Delayed vomiting

| Do | 2.33 (1.53; 3.55) | . | . | 1.27 (0.79; 2.04) | . | . | . | . | . | . | . |
| --- | --- | --- | --- | --- | --- | --- | --- | --- | --- | --- | --- |
| 2.33 (1.53; 3.55) | Do+D | . | . | . | . | . | . | . | . | . | . |
| 1.45 (0.82; 2.58) | 0.62 (0.31; 1.27) | G | 0.93 (0.61; 1.41) | 1.14 (0.66; 1.97) | . | 1.12 (0.72; 1.73) | . | . | . | 1.14 (0.66; 1.97) | . |
| 1.41 (0.78; 2.54) | 0.60 (0.29; 1.24) | 0.97 (0.69; 1.36) | G+D | . | 0.99 (0.69; 1.42) | . | 1.61 (1.20; 2.16) | . | 0.97 (0.55; 1.74) | . | . |
| 1.27 (0.79; 2.04) | 0.54 (0.29; 1.02) | 0.87 (0.63; 1.22) | 0.90 (0.63; 1.28) | O | 1.28 (0.89; 1.85) | 1.87 (1.16; 3.02) | . | . | . | 0.98 (0.71; 1.34) | . |
| 1.57 (0.89; 2.78) | 0.67 (0.33; 1.37) | 1.09 (0.76; 1.56) | 1.12 (0.88; 1.41) | 1.24 (0.90; 1.70) | O+D | . | 1.33 (1.10; 1.60) | . | . | . | . |
| 1.93 (1.06; 3.51) | 0.83 (0.40; 1.72) | 1.33 (0.93; 1.89) | 1.37 (0.88; 2.13) | 1.52 (1.05; 2.20) | 1.22 (0.79; 1.90) | P | . | . | . | . | . |
| 2.14 (1.19; 3.84) | 0.92 (0.45; 1.89) | 1.47 (1.02; 2.14) | 1.52 (1.21; 1.91) | 1.69 (1.19; 2.38) | 1.36 (1.14; 1.62) | 1.11 (0.70; 1.75) | P+D | . | . | . | . |
| 0.84 (0.34; 2.11) | 0.36 (0.13; 0.99) | 0.58 (0.27; 1.27) | 0.60 (0.30; 1.21) | 0.66 (0.30; 1.46) | 0.54 (0.25; 1.12) | 0.44 (0.19; 1.00) | 0.39 (0.19; 0.83) | R | 1.63 (1.09; 2.43) | . | . |
| 1.37 (0.60; 3.13) | 0.59 (0.23; 1.48) | 0.95 (0.48; 1.85) | 0.97 (0.55; 1.74) | 1.08 (0.55; 2.13) | 0.87 (0.47; 1.63) | 0.71 (0.34; 1.47) | 0.64 (0.34; 1.20) | 1.63 (1.09; 2.43) | R+D | . | . |
| 1.31 (0.75; 2.31) | 0.56 (0.28; 1.14) | 0.91 (0.61; 1.35) | 0.93 (0.60; 1.45) | 1.04 (0.76; 1.41) | 0.83 (0.55; 1.27) | 0.68 (0.43; 1.07) | 0.61 (0.39; 0.96) | 1.56 (0.68; 3.58) | 0.96 (0.46; 1.98) | T | 1.59 (1.10; 2.30) |
| 2.09 (1.06; 4.10) | 0.89 (0.40; 1.98) | 1.44 (0.84; 2.47) | 1.48 (0.83; 2.63) | 1.64 (1.02; 2.66) | 1.33 (0.76; 2.32) | 1.08 (0.60; 1.94) | 0.98 (0.55; 1.74) | 2.48 (1.00; 6.14) | 1.52 (0.67; 3.44) | 1.59 (1.10; 2.30) | T+D |

P-score

P+D 0.8620

Do+D 0.8490

T+D 0.8081

P 0.7652

O+D 0.5679

G 0.4559

R+D 0.4251

G+D 0.4134

T 0.3398

O 0.2871

Do 0.1389

R 0.0874

9、The outcomes after the article of “Gralla RJ 1998” was deleted.

Acute nausea

| A+D | . | . | 1.16 (0.67; 2.00) | . | . | . | . | . | . | . | . |
| --- | --- | --- | --- | --- | --- | --- | --- | --- | --- | --- | --- |
| 0.88 (0.49; 1.59) | Do | 0.98 (0.85; 1.12) | . | 0.99 (0.68; 1.42) | . | . | . | . | . | . | . |
| 0.87 (0.49; 1.54) | 0.98 (0.86; 1.12) | G | 1.32 (1.07; 1.63) | 0.99 (0.92; 1.06) | . | . | . | 1.32 (0.97; 1.81) | . | 0.41 (0.23; 0.73) | . |
| 1.16 (0.67; 2.00) | 1.31 (1.04; 1.65) | 1.34 (1.10; 1.62) | G+D | . | 1.01 (0.82; 1.23) | . | 0.97 (0.84; 1.12) | . | 1.22 (0.66; 2.23) | . | . |
| 0.85 (0.48; 1.52) | 0.96 (0.84; 1.11) | 0.98 (0.91; 1.05) | 0.73 (0.60; 0.90) | O | 1.26 (0.74; 2.12) | 1.33 (0.80; 2.20) | . | 1.16 (0.72; 1.85) | . | 1.10 (0.78; 1.56) | . |
| 1.09 (0.62; 1.92) | 1.23 (0.95; 1.60) | 1.26 (1.00; 1.58) | 0.94 (0.81; 1.09) | 1.28 (1.01; 1.62) | O+D | . | 1.14 (0.93; 1.38) | . | . | . | . |
| 1.13 (0.53; 2.44) | 1.28 (0.76; 2.16) | 1.31 (0.79; 2.17) | 0.98 (0.57; 1.68) | 1.33 (0.80; 2.20) | 1.04 (0.60; 1.81) | P | . | . | . | . | . |
| 1.16 (0.67; 2.03) | 1.32 (1.02; 1.70) | 1.34 (1.07; 1.68) | 1.00 (0.88; 1.14) | 1.37 (1.08; 1.72) | 1.07 (0.92; 1.24) | 1.03 (0.59; 1.79) | P+D | . | . | . | . |
| 1.06 (0.57; 1.98) | 1.20 (0.91; 1.59) | 1.23 (0.96; 1.57) | 0.92 (0.68; 1.23) | 1.25 (0.97; 1.61) | 0.98 (0.71; 1.35) | 0.94 (0.53; 1.65) | 0.91 (0.66; 1.26) | R | 1.61 (1.16; 2.24) | . | . |
| 1.64 (0.85; 3.16) | 1.86 (1.28; 2.69) | 1.89 (1.33; 2.68) | 1.41 (0.98; 2.04) | 1.93 (1.35; 2.75) | 1.50 (1.02; 2.23) | 1.45 (0.78; 2.68) | 1.41 (0.96; 2.08) | 1.54 (1.15; 2.07) | R+D | . | . |
| 0.73 (0.38; 1.39) | 0.82 (0.59; 1.14) | 0.84 (0.62; 1.13) | 0.63 (0.44; 0.90) | 0.86 (0.64; 1.15) | 0.67 (0.46; 0.97) | 0.64 (0.36; 1.15) | 0.63 (0.43; 0.91) | 0.68 (0.47; 1.01) | 0.44 (0.28; 0.70) | T | 1.85 (1.26; 2.74) |
| 1.35 (0.63; 2.88) | 1.53 (0.92; 2.54) | 1.56 (0.95; 2.55) | 1.16 (0.69; 1.97) | 1.59 (0.97; 2.59) | 1.24 (0.72; 2.13) | 1.19 (0.59; 2.41) | 1.16 (0.68; 1.99) | 1.27 (0.73; 2.20) | 0.82 (0.45; 1.50) | 1.85 (1.26; 2.74) | T+D |

P-score

R+D 0.9500

T+D 0.7830

P+D 0.6888

G+D 0.6840

P 0.6001

O+D 0.5588

R 0.5445

A+D 0.4484

Do 0.2670

G 0.2324

O 0.1796

T 0.0633

Acute vomiting

| Do | 0.96 (0.81; 1.14) | . | 0.98 (0.86; 1.13) | . | . | . | . | . | . | . |
| --- | --- | --- | --- | --- | --- | --- | --- | --- | --- | --- |
| 0.94 (0.83; 1.05) | G | 1.57 (1.30; 1.89) | 1.07 (0.98; 1.17) | . | 1.61 (0.95; 2.74) | . | 1.32 (0.95; 1.84) | . | 0.92 (0.66; 1.27) | . |
| 1.49 (1.22; 1.83) | 1.59 (1.35; 1.88) | G+D | . | 0.97 (0.74; 1.27) | . | 1.05 (0.83; 1.33) | . | 0.84 (0.48; 1.46) | . | . |
| 1.00 (0.89; 1.12) | 1.06 (0.98; 1.15) | 0.67 (0.56; 0.80) | O | 1.27 (0.88; 1.83) | 1.98 (0.60; 6.51) | . | 1.06 (0.75; 1.50) | . | 0.78 (0.60; 1.02) | . |
| 1.31 (1.03; 1.67) | 1.40 (1.12; 1.74) | 0.88 (0.73; 1.05) | 1.31 (1.05; 1.64) | O+D | . | 1.32 (1.09; 1.59) | . | . | . | . |
| 1.58 (0.96; 2.60) | 1.69 (1.04; 2.74) | 1.06 (0.63; 1.77) | 1.58 (0.97; 2.58) | 1.20 (0.71; 2.05) | P | . | . | . | . | . |
| 1.66 (1.29; 2.15) | 1.78 (1.41; 2.25) | 1.12 (0.93; 1.34) | 1.67 (1.31; 2.12) | 1.27 (1.08; 1.49) | 1.05 (0.61; 1.81) | P+D | . | . | . | . |
| 1.10 (0.86; 1.42) | 1.18 (0.93; 1.48) | 0.74 (0.56; 0.97) | 1.10 (0.88; 1.39) | 0.84 (0.62; 1.14) | 0.70 (0.41; 1.19) | 0.66 (0.48; 0.91) | R | 1.58 (1.07; 2.35) | . | . |
| 1.56 (1.07; 2.27) | 1.67 (1.16; 2.39) | 1.05 (0.72; 1.51) | 1.56 (1.09; 2.25) | 1.19 (0.80; 1.78) | 0.99 (0.54; 1.81) | 0.94 (0.63; 1.41) | 1.42 (1.02; 1.98) | R+D | . | . |
| 0.79 (0.62; 1.02) | 0.85 (0.67; 1.07) | 0.53 (0.40; 0.71) | 0.80 (0.63; 1.00) | 0.61 (0.44; 0.83) | 0.50 (0.29; 0.86) | 0.48 (0.35; 0.66) | 0.72 (0.52; 0.99) | 0.51 (0.33; 0.78) | T | 1.99 (1.43; 2.76) |
| 1.58 (1.05; 2.38) | 1.69 (1.13; 2.51) | 1.06 (0.69; 1.63) | 1.58 (1.06; 2.36) | 1.21 (0.77; 1.90) | 1.00 (0.53; 1.87) | 0.95 (0.60; 1.50) | 1.43 (0.91; 2.26) | 1.01 (0.59; 1.73) | 1.99 (1.43; 2.76) | T+D |

P-score

P+D 0.8653

T+D 0.7730

R+D 0.7701

P 0.7590

G+D 0.7248

O+D 0.5576

R 0.3796

O 0.2654

Do 0.2651

G 0.1230

T 0.0171

Acute complete control

| G | 0.80 (0.61; 1.04) | 0.96 (0.89; 1.04) | . | . | . | . | . | 1.16 (0.92; 1.47) | . |
| --- | --- | --- | --- | --- | --- | --- | --- | --- | --- |
| 0.85 (0.73; 1.00) | G+D | . | 1.01 (0.93; 1.11) | . | 0.98 (0.91; 1.05) | . | 1.05 (0.87; 1.27) | . | . |
| 0.96 (0.89; 1.03) | 1.12 (0.96; 1.31) | O | 0.94 (0.80; 1.12) | 0.98 (0.91; 1.05) | . | . | . | 1.13 (0.89; 1.44) | . |
| 0.88 (0.75; 1.03) | 1.03 (0.96; 1.11) | 0.92 (0.79; 1.06) | O+D | . | 0.93 (0.84; 1.03) | . | . | . | . |
| 0.93 (0.84; 1.04) | 1.09 (0.92; 1.30) | 0.98 (0.91; 1.05) | 1.06 (0.90; 1.25) | P | . | . | . | . | . |
| 0.83 (0.70; 0.98) | 0.97 (0.91; 1.03) | 0.87 (0.74; 1.02) | 0.94 (0.87; 1.02) | 0.89 (0.74; 1.06) | P+D | . | . | . | . |
| 1.13 (0.82; 1.55) | 1.33 (1.01; 1.74) | 1.18 (0.86; 1.62) | 1.29 (0.97; 1.70) | 1.21 (0.88; 1.67) | 1.36 (1.03; 1.80) | R | 0.79 (0.65; 0.96) | . | . |
| 0.90 (0.70; 1.15) | 1.05 (0.87; 1.27) | 0.94 (0.73; 1.20) | 1.02 (0.83; 1.25) | 0.96 (0.74; 1.24) | 1.08 (0.88; 1.32) | 0.79 (0.65; 0.96) | R+D | . | . |
| 1.12 (0.90; 1.40) | 1.32 (1.01; 1.72) | 1.17 (0.94; 1.46) | 1.28 (0.99; 1.66) | 1.20 (0.96; 1.52) | 1.36 (1.04; 1.77) | 0.99 (0.68; 1.45) | 1.25 (0.90; 1.74) | T | 0.65 (0.53; 0.80) |
| 0.73 (0.54; 0.99) | 0.86 (0.61; 1.20) | 0.76 (0.56; 1.03) | 0.83 (0.60; 1.16) | 0.78 (0.57; 1.07) | 0.88 (0.63; 1.24) | 0.65 (0.42; 0.99) | 0.81 (0.55; 1.20) | 0.65 (0.53; 0.80) | T+D |

P-score

T+D 0.9067

P+D 0.8435

G+D 0.7306

O+D 0.6097

R+D 0.5700

P 0.4861

O 0.3862

G 0.2416

R 0.1207

T 0.1050

10、The outcomes after the article of “Heron J.F. 1994” was deleted.

Acute nausea

| A+D | . | . | 1.16 (0.67; 2.00) | . | . | . | . | . | . | . | . |
| --- | --- | --- | --- | --- | --- | --- | --- | --- | --- | --- | --- |
| 0.87 (0.46; 1.62) | Do | 0.98 (0.85; 1.13) | . | 0.99 (0.68; 1.43) | . | . | . | . | . | . | . |
| 0.85 (0.46; 1.57) | 0.98 (0.86; 1.12) | G | 1.35 (0.95; 1.92) | 1.00 (0.93; 1.08) | . | . | . | 1.32 (0.96; 1.82) | . | 0.41 (0.23; 0.73) | . |
| 1.16 (0.67; 2.00) | 1.34 (0.99; 1.82) | 1.36 (1.03; 1.80) | G+D | . | 1.04 (0.91; 1.19) | . | 0.97 (0.83; 1.13) | . | 1.22 (0.66; 2.23) | . | . |
| 0.85 (0.46; 1.56) | 0.97 (0.84; 1.13) | 0.99 (0.93; 1.07) | 0.73 (0.55; 0.96) | O | 1.26 (0.74; 2.13) | 1.33 (0.80; 2.21) | . | 1.16 (0.72; 1.86) | . | 1.10 (0.78; 1.56) | . |
| 1.15 (0.66; 2.01) | 1.33 (0.97; 1.82) | 1.35 (1.01; 1.80) | 0.99 (0.88; 1.11) | 1.36 (1.02; 1.82) | O+D | . | 1.14 (0.93; 1.39) | . | . | . | . |
| 1.13 (0.51; 2.49) | 1.30 (0.77; 2.19) | 1.32 (0.79; 2.20) | 0.97 (0.54; 1.73) | 1.33 (0.80; 2.21) | 0.98 (0.55; 1.75) | P | . | . | . | . | . |
| 1.19 (0.68; 2.08) | 1.37 (0.99; 1.90) | 1.40 (1.04; 1.89) | 1.03 (0.90; 1.17) | 1.41 (1.04; 1.91) | 1.03 (0.90; 1.19) | 1.06 (0.59; 1.91) | P+D | . | . | . | . |
| 1.05 (0.55; 2.00) | 1.21 (0.91; 1.60) | 1.23 (0.96; 1.58) | 0.90 (0.64; 1.28) | 1.24 (0.96; 1.60) | 0.91 (0.64; 1.30) | 0.93 (0.53; 1.64) | 0.88 (0.61; 1.27) | R | 1.61 (1.16; 2.25) | . | . |
| 1.62 (0.83; 3.18) | 1.87 (1.27; 2.74) | 1.91 (1.33; 2.73) | 1.40 (0.94; 2.07) | 1.92 (1.33; 2.76) | 1.41 (0.94; 2.11) | 1.44 (0.77; 2.68) | 1.36 (0.90; 2.06) | 1.55 (1.14; 2.09) | R+D | . | . |
| 0.72 (0.36; 1.42) | 0.83 (0.60; 1.15) | 0.84 (0.62; 1.14) | 0.62 (0.41; 0.93) | 0.85 (0.63; 1.15) | 0.62 (0.41; 0.95) | 0.64 (0.36; 1.15) | 0.60 (0.40; 0.92) | 0.69 (0.46; 1.01) | 0.44 (0.28; 0.71) | T | 1.85 (1.25; 2.75) |
| 1.33 (0.61; 2.93) | 1.54 (0.92; 2.56) | 1.57 (0.95; 2.57) | 1.15 (0.65; 2.02) | 1.58 (0.96; 2.58) | 1.16 (0.65; 2.05) | 1.18 (0.58; 2.40) | 1.12 (0.63; 2.00) | 1.27 (0.73; 2.21) | 0.82 (0.45; 1.52) | 1.85 (1.25; 2.75) | T+D |

P-score

R+D 0.9418

T+D 0.7646

P+D 0.7035

G+D 0.6513

O+D 0.6314

P 0.5912

R 0.5247

A+D 0.4476

Do 0.2604

G 0.2180

O 0.2005

T 0.0651

Acute vomiting

| Do | 0.96 (0.80; 1.15) | . | 0.98 (0.85; 1.14) | . | . | . | . | . | . | . |
| --- | --- | --- | --- | --- | --- | --- | --- | --- | --- | --- |
| 0.93 (0.82; 1.05) | G | 1.72 (1.36; 2.17) | 1.09 (0.99; 1.19) | . | 1.61 (0.94; 2.76) | . | 1.32 (0.94; 1.86) | . | 0.91 (0.65; 1.27) | . |
| 1.55 (1.23; 1.95) | 1.67 (1.37; 2.03) | G+D | . | 1.06 (0.89; 1.27) | . | 1.05 (0.82; 1.35) | . | 0.84 (0.48; 1.46) | . | . |
| 1.01 (0.89; 1.13) | 1.09 (1.00; 1.18) | 0.65 (0.53; 0.80) | O | 1.27 (0.88; 1.84) | 1.98 (0.60; 6.52) | . | 1.06 (0.74; 1.51) | . | 0.78 (0.60; 1.02) | . |
| 1.49 (1.16; 1.91) | 1.61 (1.29; 2.01) | 0.96 (0.83; 1.12) | 1.48 (1.18; 1.86) | O+D | . | 1.32 (1.09; 1.59) | . | . | . | . |
| 1.57 (0.95; 2.60) | 1.69 (1.04; 2.76) | 1.01 (0.60; 1.72) | 1.56 (0.95; 2.56) | 1.05 (0.61; 1.80) | P | . | . | . | . | . |
| 1.83 (1.39; 2.41) | 1.98 (1.54; 2.54) | 1.18 (0.99; 1.41) | 1.82 (1.41; 2.35) | 1.23 (1.05; 1.44) | 1.17 (0.67; 2.02) | P+D | . | . | . | . |
| 1.10 (0.85; 1.43) | 1.19 (0.94; 1.51) | 0.71 (0.53; 0.95) | 1.10 (0.87; 1.39) | 0.74 (0.54; 1.01) | 0.70 (0.41; 1.21) | 0.60 (0.43; 0.84) | R | 1.58 (1.06; 2.36) | . | . |
| 1.58 (1.07; 2.32) | 1.70 (1.18; 2.47) | 1.02 (0.70; 1.48) | 1.57 (1.08; 2.27) | 1.06 (0.71; 1.57) | 1.01 (0.55; 1.86) | 0.86 (0.57; 1.30) | 1.43 (1.02; 2.01) | R+D | . | . |
| 0.79 (0.61; 1.03) | 0.86 (0.68; 1.08) | 0.51 (0.38; 0.69) | 0.79 (0.63; 0.99) | 0.53 (0.39; 0.73) | 0.51 (0.29; 0.87) | 0.43 (0.31; 0.61) | 0.72 (0.52; 1.00) | 0.50 (0.33; 0.78) | T | 1.99 (1.43; 2.78) |
| 1.58 (1.04; 2.41) | 1.71 (1.14; 2.56) | 1.02 (0.65; 1.60) | 1.57 (1.05; 2.36) | 1.06 (0.67; 1.68) | 1.01 (0.53; 1.90) | 0.86 (0.54; 1.39) | 1.43 (0.90; 2.28) | 1.00 (0.58; 1.73) | 1.99 (1.43; 2.78) | T+D |

P-score

P+D 0.9158

R+D 0.7359

T+D 0.7314

P 0.7157

G+D 0.7126

O+D 0.6497

R 0.3677

O 0.2781

Do 0.2601

G 0.1138

T 0.0193

Acute complete control

| G | 0.80 (0.61; 1.04) | 0.96 (0.89; 1.03) | . | . | . | . | . | 1.16 (0.92; 1.47) | . |
| --- | --- | --- | --- | --- | --- | --- | --- | --- | --- |
| 0.86 (0.74; 1.01) | G+D | . | 0.99 (0.92; 1.06) | . | 0.98 (0.91; 1.05) | . | 1.05 (0.87; 1.27) | . | . |
| 0.95 (0.88; 1.02) | 1.10 (0.95; 1.28) | O | 0.94 (0.80; 1.12) | 0.98 (0.91; 1.05) | . | . | . | 1.13 (0.89; 1.44) | . |
| 0.87 (0.74; 1.01) | 1.01 (0.95; 1.07) | 0.91 (0.79; 1.06) | O+D | . | 0.93 (0.84; 1.03) | . | . | . | . |
| 0.93 (0.83; 1.03) | 1.08 (0.91; 1.27) | 0.98 (0.91; 1.05) | 1.07 (0.91; 1.26) | P | . | . | . | . | . |
| 0.83 (0.71; 0.98) | 0.96 (0.91; 1.03) | 0.87 (0.75; 1.02) | 0.96 (0.89; 1.03) | 0.90 (0.75; 1.07) | P+D | . | . | . | . |
| 1.14 (0.83; 1.57) | 1.33 (1.01; 1.74) | 1.20 (0.88; 1.64) | 1.32 (1.00; 1.74) | 1.23 (0.90; 1.70) | 1.37 (1.04; 1.82) | R | 0.79 (0.65; 0.96) | . | . |
| 0.91 (0.71; 1.16) | 1.05 (0.87; 1.27) | 0.95 (0.75; 1.22) | 1.04 (0.85; 1.28) | 0.98 (0.76; 1.26) | 1.09 (0.89; 1.33) | 0.79 (0.65; 0.96) | R+D | . | . |
| 1.12 (0.90; 1.39) | 1.30 (1.00; 1.69) | 1.18 (0.95; 1.47) | 1.29 (0.99; 1.67) | 1.21 (0.96; 1.52) | 1.35 (1.03; 1.76) | 0.98 (0.67; 1.43) | 1.24 (0.89; 1.71) | T | 0.65 (0.53; 0.80) |
| 0.73 (0.54; 0.98) | 0.84 (0.60; 1.18) | 0.77 (0.57; 1.03) | 0.84 (0.60; 1.17) | 0.78 (0.57; 1.07) | 0.87 (0.62; 1.23) | 0.64 (0.41; 0.98) | 0.80 (0.55; 1.18) | 0.65 (0.53; 0.80) | T+D |

P-score

T+D 0.9110

P+D 0.8426

G+D 0.6900

O+D 0.6537

R+D 0.5415

P 0.5029

O 0.4027

G 0.2377

T 0.1097

R 0.1082

Delayed nausea

| Do | 1.99 (1.30; 3.04) | . | . | 0.91 (0.65; 1.29) | . | . | . | . | . | . | . |
| --- | --- | --- | --- | --- | --- | --- | --- | --- | --- | --- | --- |
| 1.99 (1.30; 3.04) | Do+D | . | . | . | . | . | . | . | . | . | . |
| 0.78 (0.41; 1.47) | 0.39 (0.18; 0.84) | G | . | 1.17 (0.69; 2.00) | . | . | . | . | . | . | . |
| 1.24 (0.74; 2.10) | 0.63 (0.32; 1.23) | 1.60 (0.82; 3.10) | G+D | . | 1.07 (0.78; 1.48) | . | 1.40 (1.09; 1.79) | . | 1.02 (0.65; 1.59) | . | . |
| 0.91 (0.65; 1.29) | 0.46 (0.27; 0.79) | 1.17 (0.69; 2.00) | 0.73 (0.50; 1.09) | O | 1.43 (1.04; 1.95) | 1.42 (1.05; 1.94) | . | . | . | 1.21 (0.81; 1.80) | . |
| 1.30 (0.82; 2.08) | 0.66 (0.35; 1.23) | 1.67 (0.90; 3.11) | 1.05 (0.83; 1.33) | 1.43 (1.04; 1.95) | O+D | . | 1.37 (1.07; 1.75) | . | . | . | . |
| 1.30 (0.82; 2.07) | 0.65 (0.35; 1.23) | 1.67 (0.90; 3.10) | 1.05 (0.63; 1.73) | 1.42 (1.05; 1.94) | 1.00 (0.64; 1.55) | P | . | . | . | . | . |
| 1.76 (1.05; 2.94) | 0.89 (0.46; 1.73) | 2.26 (1.18; 4.36) | 1.42 (1.15; 1.75) | 1.93 (1.32; 2.82) | 1.35 (1.10; 1.67) | 1.36 (0.83; 2.21) | P+D | . | . | . | . |
| 0.82 (0.38; 1.80) | 0.41 (0.17; 1.01) | 1.06 (0.44; 2.55) | 0.66 (0.37; 1.18) | 0.90 (0.45; 1.82) | 0.63 (0.34; 1.18) | 0.63 (0.29; 1.36) | 0.47 (0.25; 0.87) | R | 1.53 (1.07; 2.21) | . | . |
| 1.26 (0.63; 2.52) | 0.63 (0.28; 1.43) | 1.62 (0.73; 3.61) | 1.02 (0.65; 1.59) | 1.38 (0.76; 2.52) | 0.97 (0.58; 1.61) | 0.97 (0.49; 1.90) | 0.72 (0.44; 1.18) | 1.53 (1.07; 2.21) | R+D | . | . |
| 1.10 (0.65; 1.87) | 0.55 (0.28; 1.09) | 1.42 (0.73; 2.76) | 0.89 (0.51; 1.55) | 1.21 (0.81; 1.80) | 0.85 (0.51; 1.41) | 0.85 (0.51; 1.40) | 0.63 (0.36; 1.08) | 1.34 (0.60; 3.00) | 0.87 (0.43; 1.79) | T | 1.49 (1.01; 2.18) |
| 1.64 (0.85; 3.15) | 0.82 (0.38; 1.80) | 2.10 (0.98; 4.54) | 1.32 (0.67; 2.60) | 1.79 (1.03; 3.12) | 1.26 (0.66; 2.38) | 1.26 (0.67; 2.38) | 0.93 (0.47; 1.82) | 1.99 (0.81; 4.86) | 1.30 (0.57; 2.93) | 1.49 (1.01; 2.18) | T+D |

P-score

Do+D 0.8940

P+D 0.8782

T+D 0.7786

O+D 0.5917

P 0.5877

R+D 0.5504

G+D 0.5255

T 0.3997

Do 0.3036

O 0.1881

R 0.1706

G 0.1319

Delayed vomiting

| Do | 2.33 (1.53; 3.56) | . | . | 1.27 (0.79; 2.04) | . | . | . | . | . | . | . |
| --- | --- | --- | --- | --- | --- | --- | --- | --- | --- | --- | --- |
| 2.33 (1.53; 3.56) | Do+D | . | . | . | . | . | . | . | . | . | . |
| 1.31 (0.75; 2.30) | 0.56 (0.28; 1.14) | G | . | 1.09 (0.76; 1.56) | . | 1.12 (0.72; 1.73) | . | . | . | 1.14 (0.66; 1.97) | . |
| 1.48 (0.77; 2.84) | 0.63 (0.29; 1.38) | 1.13 (0.66; 1.93) | G+D | . | 0.99 (0.69; 1.43) | . | 1.61 (1.20; 2.16) | . | 0.97 (0.54; 1.74) | . | . |
| 1.27 (0.79; 2.04) | 0.54 (0.29; 1.03) | 0.97 (0.72; 1.31) | 0.86 (0.55; 1.34) | O | 1.28 (0.89; 1.85) | 1.87 (1.16; 3.03) | . | . | . | 0.98 (0.71; 1.34) | . |
| 1.63 (0.89; 2.97) | 0.70 (0.34; 1.45) | 1.24 (0.77; 2.00) | 1.10 (0.85; 1.42) | 1.28 (0.89; 1.85) | O+D | . | 1.33 (1.10; 1.60) | . | . | . | . |
| 1.83 (1.01; 3.32) | 0.78 (0.38; 1.63) | 1.39 (0.98; 1.98) | 1.23 (0.69; 2.19) | 1.44 (1.00; 2.07) | 1.12 (0.67; 1.88) | P | . | . | . | . | . |
| 2.23 (1.19; 4.16) | 0.95 (0.45; 2.03) | 1.69 (1.02; 2.81) | 1.50 (1.18; 1.91) | 1.75 (1.17; 2.63) | 1.37 (1.15; 1.63) | 1.22 (0.71; 2.10) | P+D | . | . | . | . |
| 0.89 (0.34; 2.31) | 0.38 (0.13; 1.08) | 0.67 (0.28; 1.64) | 0.60 (0.30; 1.21) | 0.70 (0.30; 1.61) | 0.54 (0.26; 1.15) | 0.49 (0.20; 1.21) | 0.40 (0.19; 0.84) | R | 1.63 (1.09; 2.43) | . | . |
| 1.44 (0.60; 3.45) | 0.62 (0.23; 1.63) | 1.10 (0.50; 2.42) | 0.97 (0.54; 1.74) | 1.14 (0.55; 2.36) | 0.88 (0.47; 1.67) | 0.79 (0.35; 1.79) | 0.65 (0.35; 1.21) | 1.63 (1.09; 2.43) | R+D | . | . |
| 1.29 (0.73; 2.27) | 0.55 (0.27; 1.12) | 0.98 (0.67; 1.44) | 0.87 (0.50; 1.50) | 1.01 (0.74; 1.38) | 0.79 (0.49; 1.28) | 0.70 (0.45; 1.11) | 0.58 (0.35; 0.96) | 1.45 (0.60; 3.54) | 0.89 (0.40; 1.98) | T | 1.59 (1.10; 2.30) |
| 2.04 (1.04; 4.02) | 0.88 (0.39; 1.95) | 1.56 (0.91; 2.66) | 1.38 (0.72; 2.66) | 1.61 (0.99; 2.61) | 1.25 (0.68; 2.30) | 1.12 (0.62; 2.01) | 0.92 (0.49; 1.73) | 2.31 (0.88; 6.06) | 1.42 (0.59; 3.41) | 1.59 (1.10; 2.30) | T+D |

P-score

P+D 0.8793

Do+D 0.8448

T+D 0.7862

P 0.7078

O+D 0.5999

G+D 0.4741

R+D 0.4716

G 0.3503

T 0.3270

O 0.3008

Do 0.1411

R 0.1170

Delayed complete control

| G+D | . | 0.94 (0.75; 1.17) | . | 0.80 (0.65; 0.99) | 0.92 (0.58; 1.47) |
| --- | --- | --- | --- | --- | --- |
| 1.63 (1.18; 2.27) | O | 0.61 (0.47; 0.81) | 0.70 (0.49; 0.99) | . | . |
| 1.00 (0.84; 1.20) | 0.61 (0.47; 0.81) | O+D | . | 0.70 (0.56; 0.88) | . |
| 1.14 (0.71; 1.84) | 0.70 (0.49; 0.99) | 1.14 (0.73; 1.77) | P | . | . |
| 0.75 (0.63; 0.90) | 0.46 (0.33; 0.64) | 0.75 (0.63; 0.90) | 0.66 (0.41; 1.06) | P+D | . |
| 0.92 (0.58; 1.47) | 0.57 (0.32; 1.00) | 0.92 (0.56; 1.51) | 0.81 (0.42; 1.58) | 1.23 (0.75; 2.01) | R+D |

P-score

P+D 0.9493

R+D 0.6343

G+D 0.5168

O+D 0.5157

P 0.3743

O 0.0096

11、The outcome after the article of “Hesketh P 1996” was deleted.

Acute vomiting

| Do | 0.96 (0.79; 1.16) | . | 1.34 (0.72; 2.48) | . | . | . | . | . | . | . |
| --- | --- | --- | --- | --- | --- | --- | --- | --- | --- | --- |
| 0.98 (0.81; 1.18) | G | 1.57 (1.28; 1.92) | 1.09 (0.98; 1.20) | . | 1.61 (0.93; 2.78) | . | 1.32 (0.93; 1.88) | . | 0.91 (0.64; 1.28) | . |
| 1.54 (1.19; 1.99) | 1.57 (1.32; 1.88) | G+D | . | 1.06 (0.87; 1.29) | . | 1.05 (0.80; 1.37) | . | 0.84 (0.48; 1.47) | . | . |
| 1.07 (0.87; 1.31) | 1.10 (1.00; 1.20) | 0.70 (0.58; 0.84) | O | 1.28 (0.88; 1.86) | 1.98 (0.60; 6.53) | . | 1.06 (0.73; 1.53) | . | 0.78 (0.59; 1.03) | . |
| 1.49 (1.13; 1.98) | 1.53 (1.23; 1.89) | 0.97 (0.83; 1.13) | 1.39 (1.12; 1.74) | O+D | . | 1.31 (1.08; 1.60) | . | . | . | . |
| 1.66 (0.98; 2.82) | 1.70 (1.03; 2.79) | 1.08 (0.64; 1.83) | 1.55 (0.94; 2.56) | 1.11 (0.65; 1.91) | P | . | . | . | . | . |
| 1.83 (1.35; 2.49) | 1.88 (1.46; 2.40) | 1.19 (0.99; 1.44) | 1.71 (1.33; 2.21) | 1.23 (1.04; 1.45) | 1.10 (0.63; 1.92) | P+D | . | . | . | . |
| 1.16 (0.85; 1.57) | 1.18 (0.93; 1.51) | 0.75 (0.56; 1.00) | 1.08 (0.84; 1.38) | 0.78 (0.57; 1.06) | 0.70 (0.40; 1.21) | 0.63 (0.45; 0.88) | R | 1.58 (1.05; 2.39) | . | . |
| 1.62 (1.07; 2.46) | 1.66 (1.14; 2.41) | 1.05 (0.72; 1.54) | 1.51 (1.03; 2.21) | 1.09 (0.72; 1.63) | 0.98 (0.52; 1.82) | 0.88 (0.58; 1.35) | 1.40 (0.99; 1.98) | R+D | . | . |
| 0.84 (0.62; 1.14) | 0.86 (0.67; 1.10) | 0.55 (0.40; 0.74) | 0.78 (0.62; 1.00) | 0.56 (0.41; 0.77) | 0.51 (0.29; 0.88) | 0.46 (0.33; 0.65) | 0.73 (0.52; 1.02) | 0.52 (0.33; 0.81) | T | 1.99 (1.42; 2.81) |
| 1.68 (1.06; 2.65) | 1.71 (1.13; 2.61) | 1.09 (0.69; 1.72) | 1.56 (1.03; 2.37) | 1.12 (0.70; 1.79) | 1.01 (0.53; 1.93) | 0.91 (0.56; 1.48) | 1.45 (0.89; 2.34) | 1.03 (0.59; 1.81) | 1.99 (1.42; 2.81) | T+D |

P-score

P+D 0.8951

T+D 0.7640

P 0.7438

R+D 0.7403

G+D 0.6796

O+D 0.6312

R 0.3720

O 0.3049

Do 0.1948

G 0.1435

T 0.0308

12、The outcomes after the article of “Ho CL 2010” was deleted.

Acute vomiting

| Do | 0.96 (0.80; 1.14) | . | 0.98 (0.85; 1.13) | . | . | . | . | . | . | . |
| --- | --- | --- | --- | --- | --- | --- | --- | --- | --- | --- |
| 0.93 (0.82; 1.05) | G | 1.57 (1.29; 1.90) | 1.08 (0.99; 1.19) | . | 1.61 (0.94; 2.75) | . | 1.32 (0.95; 1.85) | . | 0.91 (0.66; 1.27) | . |
| 1.42 (1.16; 1.75) | 1.54 (1.30; 1.82) | G+D | . | 1.06 (0.89; 1.27) | . | 1.05 (0.82; 1.34) | . | 1.19 (0.56; 2.55) | . | . |
| 1.00 (0.90; 1.13) | 1.08 (1.00; 1.17) | 0.71 (0.59; 0.85) | O | 1.27 (0.88; 1.83) | 1.98 (0.60; 6.52) | . | 1.06 (0.75; 1.51) | . | 0.78 (0.60; 1.02) | . |
| 1.39 (1.10; 1.75) | 1.50 (1.22; 1.84) | 0.98 (0.84; 1.13) | 1.38 (1.12; 1.70) | O+D | . | 1.32 (1.09; 1.59) | . | . | . | . |
| 1.57 (0.95; 2.59) | 1.69 (1.04; 2.75) | 1.10 (0.66; 1.84) | 1.56 (0.95; 2.55) | 1.13 (0.67; 1.91) | P | . | . | . | . | . |
| 1.70 (1.31; 2.20) | 1.83 (1.45; 2.31) | 1.19 (1.00; 1.42) | 1.69 (1.33; 2.14) | 1.22 (1.04; 1.43) | 1.08 (0.63; 1.86) | P+D | . | . | . | . |
| 1.14 (0.88; 1.48) | 1.23 (0.97; 1.56) | 0.80 (0.60; 1.06) | 1.13 (0.89; 1.44) | 0.82 (0.61; 1.11) | 0.73 (0.42; 1.25) | 0.67 (0.49; 0.93) | R | 1.58 (1.06; 2.35) | . | . |
| 1.78 (1.18; 2.70) | 1.92 (1.29; 2.87) | 1.25 (0.82; 1.89) | 1.77 (1.19; 2.65) | 1.28 (0.83; 1.98) | 1.14 (0.60; 2.13) | 1.05 (0.67; 1.64) | 1.56 (1.09; 2.23) | R+D | . | . |
| 0.79 (0.62; 1.02) | 0.86 (0.68; 1.08) | 0.56 (0.42; 0.74) | 0.79 (0.63; 0.99) | 0.57 (0.42; 0.78) | 0.51 (0.30; 0.87) | 0.47 (0.34; 0.65) | 0.70 (0.50; 0.97) | 0.45 (0.28; 0.71) | T | 1.99 (1.43; 2.76) |
| 1.58 (1.04; 2.39) | 1.70 (1.14; 2.55) | 1.11 (0.72; 1.71) | 1.57 (1.05; 2.35) | 1.14 (0.73; 1.78) | 1.01 (0.54; 1.89) | 0.93 (0.59; 1.48) | 1.39 (0.87; 2.20) | 0.89 (0.50; 1.56) | 1.99 (1.43; 2.76) | T+D |

P-score

R+D 0.8608

P+D 0.8608

T+D 0.7505

P 0.7315

G+D 0.6421

O+D 0.6012

R 0.4016

O 0.2690

Do 0.2544

G 0.1103

T 0.0177

Acute complete control

| G | 0.80 (0.61; 1.04) | 0.96 (0.89; 1.03) | . | . | . | . | . | 1.16 (0.92; 1.47) | . |
| --- | --- | --- | --- | --- | --- | --- | --- | --- | --- |
| 0.86 (0.74; 1.01) | G+D | . | 0.99 (0.92; 1.06) | . | 0.98 (0.91; 1.05) | . | 0.94 (0.71; 1.24) | . | . |
| 0.95 (0.88; 1.02) | 1.10 (0.95; 1.28) | O | 0.94 (0.80; 1.12) | 0.98 (0.91; 1.05) | . | . | . | 1.13 (0.89; 1.44) | . |
| 0.87 (0.74; 1.01) | 1.01 (0.95; 1.07) | 0.91 (0.79; 1.06) | O+D | . | 0.93 (0.84; 1.03) | . | . | . | . |
| 0.93 (0.83; 1.03) | 1.08 (0.91; 1.27) | 0.98 (0.91; 1.05) | 1.07 (0.91; 1.26) | P | . | . | . | . | . |
| 0.83 (0.71; 0.98) | 0.96 (0.91; 1.03) | 0.87 (0.75; 1.02) | 0.96 (0.89; 1.03) | 0.90 (0.75; 1.07) | P+D | . | . | . | . |
| 1.02 (0.70; 1.48) | 1.18 (0.84; 1.66) | 1.07 (0.74; 1.56) | 1.17 (0.83; 1.66) | 1.10 (0.75; 1.61) | 1.23 (0.87; 1.73) | R | 0.79 (0.65; 0.96) | . | . |
| 0.81 (0.58; 1.12) | 0.94 (0.71; 1.24) | 0.85 (0.62; 1.17) | 0.93 (0.70; 1.24) | 0.87 (0.63; 1.21) | 0.97 (0.73; 1.30) | 0.79 (0.65; 0.96) | R+D | . | . |
| 1.12 (0.90; 1.39) | 1.30 (1.00; 1.69) | 1.18 (0.95; 1.47) | 1.29 (0.99; 1.67) | 1.21 (0.96; 1.52) | 1.35 (1.03; 1.76) | 1.10 (0.72; 1.69) | 1.39 (0.94; 2.04) | T | 0.65 (0.53; 0.80) |
| 0.73 (0.54; 0.98) | 0.84 (0.60; 1.18) | 0.77 (0.57; 1.03) | 0.84 (0.60; 1.17) | 0.78 (0.57; 1.07) | 0.87 (0.62; 1.23) | 0.71 (0.44; 1.15) | 0.90 (0.58; 1.40) | 0.65 (0.53; 0.80) | T+D |

P-score

T+D 0.8831

P+D 0.7882

R+D 0.7496

G+D 0.6325

O+D 0.5974

P 0.4542

O 0.3553

R 0.2635

G 0.1955

T 0.0807

13、The outcomes after the article of “Italian Group 1995” was deleted.

Acute nausea

| A+D | . | . | 1.16 (0.67; 2.00) | . | . | . | . | . | . | . | . |
| --- | --- | --- | --- | --- | --- | --- | --- | --- | --- | --- | --- |
| 0.89 (0.49; 1.61) | Do | 0.98 (0.85; 1.13) | . | 0.99 (0.68; 1.43) | . | . | . | . | . | . | . |
| 0.87 (0.49; 1.56) | 0.98 (0.86; 1.12) | G | 1.32 (1.06; 1.63) | 1.00 (0.93; 1.08) | . | . | . | 1.32 (0.96; 1.82) | . | 0.41 (0.23; 0.73) | . |
| 1.16 (0.67; 2.00) | 1.30 (1.03; 1.65) | 1.33 (1.10; 1.61) | G+D | . | 1.07 (0.89; 1.27) | . | 0.97 (0.83; 1.13) | . | 1.22 (0.66; 2.23) | . | . |
| 0.87 (0.48; 1.55) | 0.97 (0.84; 1.13) | 0.99 (0.92; 1.07) | 0.75 (0.61; 0.91) | O | 1.26 (0.74; 2.13) | 1.33 (0.80; 2.21) | . | 1.16 (0.72; 1.86) | . | 1.10 (0.78; 1.56) | . |
| 1.14 (0.65; 2.01) | 1.29 (0.99; 1.67) | 1.31 (1.04; 1.65) | 0.99 (0.86; 1.14) | 1.32 (1.05; 1.67) | O+D | . | 1.14 (0.93; 1.39) | . | . | . | . |
| 1.15 (0.53; 2.49) | 1.30 (0.77; 2.19) | 1.32 (0.79; 2.20) | 0.99 (0.58; 1.71) | 1.33 (0.80; 2.21) | 1.01 (0.58; 1.76) | P | . | . | . | . | . |
| 1.19 (0.68; 2.08) | 1.34 (1.02; 1.74) | 1.36 (1.08; 1.71) | 1.02 (0.90; 1.17) | 1.37 (1.08; 1.74) | 1.04 (0.89; 1.21) | 1.03 (0.59; 1.80) | P+D | . | . | . | . |
| 1.07 (0.57; 2.00) | 1.20 (0.91; 1.60) | 1.23 (0.96; 1.58) | 0.92 (0.68; 1.24) | 1.24 (0.96; 1.59) | 0.94 (0.68; 1.29) | 0.93 (0.53; 1.63) | 0.90 (0.65; 1.25) | R | 1.61 (1.16; 2.25) | . | . |
| 1.65 (0.85; 3.19) | 1.85 (1.27; 2.70) | 1.89 (1.33; 2.69) | 1.42 (0.98; 2.06) | 1.90 (1.33; 2.72) | 1.44 (0.97; 2.13) | 1.43 (0.77; 2.66) | 1.39 (0.94; 2.05) | 1.54 (1.14; 2.08) | R+D | . | . |
| 0.74 (0.38; 1.41) | 0.83 (0.59; 1.15) | 0.84 (0.62; 1.14) | 0.63 (0.44; 0.91) | 0.85 (0.63; 1.15) | 0.64 (0.44; 0.94) | 0.64 (0.35; 1.15) | 0.62 (0.42; 0.91) | 0.69 (0.47; 1.02) | 0.45 (0.28; 0.71) | T | 1.85 (1.25; 2.75) |
| 1.37 (0.64; 2.93) | 1.53 (0.92; 2.56) | 1.57 (0.95; 2.57) | 1.18 (0.69; 2.00) | 1.58 (0.96; 2.59) | 1.19 (0.69; 2.06) | 1.18 (0.58; 2.40) | 1.15 (0.67; 1.98) | 1.27 (0.73; 2.22) | 0.83 (0.45; 1.52) | 1.85 (1.25; 2.75) | T+D |

P-score

R+D 0.9462

T+D 0.7791

P+D 0.6960

G+D 0.6489

O+D 0.6182

P 0.6035

R 0.5328

A+D 0.4344

Do 0.2591

G 0.2179

O 0.1990

T 0.0649

Acute vomiting

| Do | 0.96 (0.79; 1.16) | . | 0.99 (0.84; 1.15) | . | . | . | . | . | . | . |
| --- | --- | --- | --- | --- | --- | --- | --- | --- | --- | --- |
| 0.93 (0.82; 1.06) | G | 1.57 (1.28; 1.91) | 1.09 (0.99; 1.20) | . | 1.61 (0.94; 2.77) | . | 1.32 (0.93; 1.87) | . | 0.91 (0.65; 1.27) | . |
| 1.46 (1.18; 1.81) | 1.57 (1.32; 1.87) | G+D | . | 1.13 (0.88; 1.45) | . | 1.05 (0.81; 1.36) | . | 0.84 (0.48; 1.47) | . | . |
| 1.01 (0.89; 1.14) | 1.08 (0.99; 1.18) | 0.69 (0.57; 0.83) | O | 1.28 (0.88; 1.85) | 1.98 (0.60; 6.53) | . | 1.06 (0.74; 1.53) | . | 0.78 (0.59; 1.03) | . |
| 1.41 (1.10; 1.82) | 1.52 (1.22; 1.90) | 0.97 (0.81; 1.16) | 1.40 (1.12; 1.76) | O+D | . | 1.32 (1.08; 1.60) | . | . | . | . |
| 1.57 (0.94; 2.62) | 1.69 (1.03; 2.78) | 1.08 (0.64; 1.82) | 1.56 (0.95; 2.58) | 1.11 (0.65; 1.91) | P | . | . | . | . | . |
| 1.74 (1.32; 2.28) | 1.87 (1.46; 2.39) | 1.19 (0.98; 1.45) | 1.72 (1.34; 2.22) | 1.23 (1.04; 1.45) | 1.10 (0.64; 1.91) | P+D | . | . | . | . |
| 1.10 (0.84; 1.43) | 1.18 (0.93; 1.50) | 0.75 (0.57; 1.00) | 1.09 (0.85; 1.39) | 0.78 (0.56; 1.06) | 0.70 (0.40; 1.21) | 0.63 (0.45; 0.88) | R | 1.58 (1.05; 2.38) | . | . |
| 1.54 (1.04; 2.27) | 1.65 (1.14; 2.40) | 1.05 (0.72; 1.54) | 1.53 (1.05; 2.22) | 1.09 (0.72; 1.63) | 0.98 (0.53; 1.81) | 0.89 (0.58; 1.35) | 1.40 (0.99; 1.98) | R+D | . | . |
| 0.79 (0.61; 1.03) | 0.85 (0.67; 1.09) | 0.54 (0.41; 0.73) | 0.79 (0.62; 1.00) | 0.56 (0.41; 0.78) | 0.50 (0.29; 0.87) | 0.46 (0.33; 0.64) | 0.72 (0.52; 1.01) | 0.52 (0.33; 0.80) | T | 1.99 (1.42; 2.80) |
| 1.58 (1.03; 2.43) | 1.70 (1.12; 2.58) | 1.08 (0.69; 1.70) | 1.57 (1.04; 2.37) | 1.12 (0.70; 1.79) | 1.01 (0.53; 1.91) | 0.91 (0.56; 1.47) | 1.44 (0.90; 2.32) | 1.03 (0.59; 1.79) | 1.99 (1.42; 2.80) | T+D |

P-score

P+D 0.8946

T+D 0.7614

P 0.7446

R+D 0.7414

G+D 0.6791

O+D 0.6326

R 0.3659

O 0.2802

Do 0.2609

G 0.1189

T 0.0205

Acute complete control

| G | 0.80 (0.61; 1.04) | 0.96 (0.89; 1.03) | . | . | . | . | . | 1.16 (0.92; 1.47) | . |
| --- | --- | --- | --- | --- | --- | --- | --- | --- | --- |
| 0.86 (0.73; 1.02) | G+D | . | 0.96 (0.86; 1.07) | . | 0.98 (0.91; 1.05) | . | 1.05 (0.87; 1.27) | . | . |
| 0.95 (0.88; 1.02) | 1.10 (0.94; 1.29) | O | 0.94 (0.80; 1.12) | 0.98 (0.91; 1.05) | . | . | . | 1.13 (0.89; 1.44) | . |
| 0.87 (0.74; 1.01) | 1.00 (0.93; 1.09) | 0.91 (0.79; 1.06) | O+D | . | 0.93 (0.84; 1.03) | . | . | . | . |
| 0.93 (0.83; 1.03) | 1.07 (0.90; 1.28) | 0.98 (0.91; 1.05) | 1.07 (0.91; 1.26) | P | . | . | . | . | . |
| 0.83 (0.71; 0.98) | 0.96 (0.90; 1.03) | 0.88 (0.75; 1.03) | 0.96 (0.89; 1.04) | 0.90 (0.75; 1.07) | P+D | . | . | . | . |
| 1.15 (0.83; 1.57) | 1.33 (1.01; 1.74) | 1.21 (0.88; 1.65) | 1.32 (0.99; 1.75) | 1.24 (0.90; 1.71) | 1.38 (1.04; 1.82) | R | 0.79 (0.65; 0.96) | . | . |
| 0.91 (0.71; 1.17) | 1.05 (0.87; 1.27) | 0.96 (0.75; 1.22) | 1.05 (0.85; 1.29) | 0.98 (0.76; 1.27) | 1.09 (0.89; 1.34) | 0.79 (0.65; 0.96) | R+D | . | . |
| 1.12 (0.90; 1.39) | 1.30 (0.99; 1.69) | 1.18 (0.95; 1.47) | 1.29 (0.99; 1.68) | 1.21 (0.96; 1.52) | 1.35 (1.03; 1.76) | 0.98 (0.67; 1.43) | 1.23 (0.89; 1.71) | T | 0.65 (0.53; 0.80) |
| 0.73 (0.54; 0.98) | 0.84 (0.60; 1.18) | 0.77 (0.57; 1.03) | 0.84 (0.60; 1.17) | 0.78 (0.57; 1.07) | 0.87 (0.62; 1.23) | 0.63 (0.41; 0.98) | 0.80 (0.54; 1.18) | 0.65 (0.53; 0.80) | T+D |

P-score

T+D 0.9113

P+D 0.8370

G+D 0.6795

O+D 0.6660

R+D 0.5373

P 0.5058

O 0.4056

G 0.2394

T 0.1109

R 0.1072

Delayed nausea

| Do | 1.99 (1.20; 3.28) | . | . | 0.91 (0.59; 1.41) | . | . | . | . | . | . | . |
| --- | --- | --- | --- | --- | --- | --- | --- | --- | --- | --- | --- |
| 1.99 (1.20; 3.28) | Do+D | . | . | . | . | . | . | . | . | . | . |
| 0.96 (0.51; 1.81) | 0.48 (0.21; 1.08) | G | 0.97 (0.62; 1.51) | 1.17 (0.65; 2.13) | . | . | . | . | . | . | . |
| 1.04 (0.55; 1.95) | 0.52 (0.23; 1.17) | 1.09 (0.74; 1.60) | G+D | . | . | . | 1.44 (1.05; 1.97) | . | 1.02 (0.60; 1.71) | . | . |
| 0.91 (0.59; 1.41) | 0.46 (0.24; 0.89) | 0.95 (0.60; 1.51) | 0.88 (0.56; 1.39) | O | 1.45 (1.00; 2.10) | 1.42 (1.00; 2.01) | . | . | . | 1.21 (0.75; 1.95) | . |
| 1.22 (0.70; 2.12) | 0.61 (0.29; 1.30) | 1.28 (0.80; 2.03) | 1.17 (0.80; 1.74) | 1.34 (0.95; 1.88) | O+D | . | 1.37 (1.00; 1.87) | . | . | . | . |
| 1.30 (0.74; 2.27) | 0.65 (0.31; 1.38) | 1.36 (0.76; 2.41) | 1.25 (0.70; 2.21) | 1.42 (1.00; 2.01) | 1.06 (0.65; 1.73) | P | . | . | . | . | . |
| 1.58 (0.87; 2.89) | 0.80 (0.36; 1.74) | 1.65 (1.06; 2.57) | 1.52 (1.13; 2.05) | 1.73 (1.14; 2.62) | 1.30 (0.97; 1.74) | 1.22 (0.71; 2.10) | P+D | . | . | . | . |
| 0.69 (0.27; 1.75) | 0.35 (0.12; 1.00) | 0.72 (0.33; 1.59) | 0.66 (0.33; 1.32) | 0.75 (0.33; 1.72) | 0.56 (0.25; 1.25) | 0.53 (0.22; 1.30) | 0.43 (0.20; 0.92) | R | 1.53 (0.98; 2.41) | . | . |
| 1.05 (0.46; 2.39) | 0.53 (0.20; 1.39) | 1.10 (0.57; 2.12) | 1.02 (0.60; 1.71) | 1.16 (0.58; 2.31) | 0.86 (0.45; 1.66) | 0.81 (0.37; 1.77) | 0.67 (0.37; 1.22) | 1.53 (0.98; 2.41) | R+D | . | . |
| 1.10 (0.58; 2.11) | 0.55 (0.24; 1.26) | 1.15 (0.59; 2.24) | 1.06 (0.55; 2.06) | 1.21 (0.75; 1.95) | 0.90 (0.50; 1.63) | 0.85 (0.47; 1.54) | 0.70 (0.37; 1.31) | 1.60 (0.62; 4.17) | 1.05 (0.45; 2.43) | T | 1.49 (0.93; 2.37) |
| 1.64 (0.74; 3.65) | 0.82 (0.32; 2.12) | 1.71 (0.76; 3.86) | 1.58 (0.70; 3.55) | 1.79 (0.92; 3.51) | 1.34 (0.63; 2.85) | 1.26 (0.59; 2.69) | 1.04 (0.47; 2.28) | 2.38 (0.82; 6.91) | 1.55 (0.59; 4.08) | 1.49 (0.93; 2.37) | T+D |

P-score

Do+D 0.8935

P+D 0.8312

T+D 0.7914

P 0.6342

O+D 0.5760

T 0.4477

R+D 0.4347

G+D 0.3939

Do 0.3539

G 0.3027

O 0.2268

R 0.1139

Delayed vomiting

| Do | 2.33 (1.49; 3.65) | . | . | 1.27 (0.77; 2.09) | . | . | . | . | . | . | . |
| --- | --- | --- | --- | --- | --- | --- | --- | --- | --- | --- | --- |
| 2.33 (1.49; 3.65) | Do+D | . | . | . | . | . | . | . | . | . | . |
| 1.33 (0.75; 2.37) | 0.57 (0.27; 1.18) | G | 0.93 (0.60; 1.45) | 1.09 (0.75; 1.59) | . | 1.12 (0.70; 1.77) | . | . | . | 1.14 (0.65; 2.02) | . |
| 1.27 (0.68; 2.38) | 0.54 (0.25; 1.18) | 0.95 (0.66; 1.37) | G+D | . | . | . | 1.65 (1.20; 2.27) | . | 0.97 (0.53; 1.77) | . | . |
| 1.27 (0.77; 2.09) | 0.54 (0.28; 1.06) | 0.95 (0.72; 1.27) | 1.00 (0.68; 1.47) | O | 1.29 (0.88; 1.90) | 1.86 (1.14; 3.04) | . | . | . | 0.98 (0.69; 1.37) | . |
| 1.61 (0.88; 2.93) | 0.69 (0.33; 1.46) | 1.21 (0.83; 1.76) | 1.27 (0.91; 1.76) | 1.27 (0.91; 1.77) | O+D | . | 1.33 (1.08; 1.63) | . | . | . | . |
| 1.85 (0.99; 3.44) | 0.79 (0.37; 1.70) | 1.39 (0.97; 2.00) | 1.46 (0.90; 2.36) | 1.46 (1.01; 2.11) | 1.15 (0.72; 1.84) | P | . | . | . | . | . |
| 2.12 (1.15; 3.92) | 0.91 (0.42; 1.95) | 1.59 (1.09; 2.34) | 1.67 (1.25; 2.23) | 1.67 (1.17; 2.39) | 1.32 (1.08; 1.61) | 1.15 (0.71; 1.86) | P+D | . | . | . | . |
| 0.76 (0.29; 2.00) | 0.33 (0.11; 0.95) | 0.57 (0.25; 1.30) | 0.60 (0.29; 1.25) | 0.60 (0.26; 1.38) | 0.47 (0.21; 1.06) | 0.41 (0.17; 0.99) | 0.36 (0.16; 0.79) | R | 1.63 (1.06; 2.50) | . | . |
| 1.23 (0.52; 2.95) | 0.53 (0.20; 1.41) | 0.93 (0.46; 1.87) | 0.97 (0.53; 1.77) | 0.97 (0.48; 1.98) | 0.77 (0.39; 1.52) | 0.67 (0.31; 1.44) | 0.58 (0.30; 1.13) | 1.63 (1.06; 2.50) | R+D | . | . |
| 1.29 (0.71; 2.35) | 0.55 (0.26; 1.17) | 0.97 (0.66; 1.44) | 1.02 (0.63; 1.65) | 1.02 (0.73; 1.41) | 0.80 (0.51; 1.27) | 0.70 (0.44; 1.12) | 0.61 (0.38; 0.98) | 1.70 (0.70; 4.12) | 1.05 (0.48; 2.26) | T | 1.60 (1.08; 2.37) |
| 2.06 (1.01; 4.22) | 0.88 (0.38; 2.06) | 1.55 (0.89; 2.71) | 1.63 (0.87; 3.03) | 1.63 (0.97; 2.72) | 1.28 (0.70; 2.35) | 1.12 (0.60; 2.07) | 0.97 (0.53; 1.80) | 2.72 (1.03; 7.15) | 1.67 (0.70; 3.97) | 1.60 (1.08; 2.37) | T+D |

P-score

P+D 0.8678

Do+D 0.8565

T+D 0.8136

P 0.7503

O+D 0.6210

G 0.4043

R+D 0.3756

T 0.3709

G+D 0.3506

O 0.3410

Do 0.1742

R 0.0742

Delayed complete control

| G | 1.12 (0.75; 1.66) | . | . | . | . | . |
| --- | --- | --- | --- | --- | --- | --- |
| 1.12 (0.75; 1.66) | G+D | . | . | . | 0.80 (0.65; 0.98) | 0.92 (0.58; 1.46) |
| 2.07 (1.18; 3.63) | 1.85 (1.24; 2.77) | O | 0.61 (0.47; 0.81) | 0.70 (0.50; 0.98) | . | . |
| 1.27 (0.78; 2.08) | 1.14 (0.85; 1.53) | 0.61 (0.47; 0.81) | O+D | . | 0.70 (0.57; 0.87) | . |
| 1.45 (0.75; 2.79) | 1.29 (0.76; 2.19) | 0.70 (0.50; 0.98) | 1.14 (0.73; 1.76) | P | . | . |
| 0.89 (0.57; 1.39) | 0.80 (0.65; 0.98) | 0.43 (0.31; 0.61) | 0.70 (0.57; 0.87) | 0.62 (0.38; 1.01) | P+D | . |
| 1.03 (0.56; 1.89) | 0.92 (0.58; 1.46) | 0.50 (0.27; 0.92) | 0.81 (0.47; 1.40) | 0.71 (0.36; 1.44) | 1.15 (0.70; 1.91) | R+D |

P-score

P+D 0.8935

G 0.7072

R+D 0.6611

G+D 0.5520

O+D 0.3852

P 0.2943

O 0.0066

14、The outcome after the article of “Joss R.A. 1994” was deleted.

Acute nausea

| A+D | . | . | 1.16 (0.67; 2.00) | . | . | . | . | . | . | . | . |
| --- | --- | --- | --- | --- | --- | --- | --- | --- | --- | --- | --- |
| 0.88 (0.49; 1.60) | Do | 0.98 (0.85; 1.13) | . | 0.99 (0.68; 1.43) | . | . | . | . | . | . | . |
| 0.87 (0.48; 1.55) | 0.98 (0.86; 1.12) | G | 1.32 (1.06; 1.63) | 1.00 (0.93; 1.08) | . | . | . | 1.32 (0.96; 1.82) | . | 0.41 (0.23; 0.73) | . |
| 1.16 (0.67; 2.00) | 1.31 (1.03; 1.68) | 1.34 (1.09; 1.65) | G+D | . | 1.04 (0.91; 1.19) | . | 0.97 (0.83; 1.13) | . | 1.22 (0.66; 2.23) | . | . |
| 0.86 (0.48; 1.54) | 0.97 (0.84; 1.13) | 0.99 (0.92; 1.07) | 0.74 (0.60; 0.92) | O | . | 1.33 (0.80; 2.21) | . | 1.16 (0.72; 1.86) | . | 1.10 (0.78; 1.56) | . |
| 1.16 (0.66; 2.02) | 1.31 (1.00; 1.72) | 1.34 (1.05; 1.69) | 1.00 (0.88; 1.12) | 1.35 (1.05; 1.72) | O+D | . | 1.14 (0.93; 1.39) | . | . | . | . |
| 1.14 (0.53; 2.48) | 1.29 (0.77; 2.19) | 1.32 (0.79; 2.20) | 0.98 (0.57; 1.71) | 1.33 (0.80; 2.21) | 0.99 (0.56; 1.74) | P | . | . | . | . | . |
| 1.19 (0.68; 2.09) | 1.35 (1.02; 1.78) | 1.38 (1.08; 1.76) | 1.03 (0.90; 1.17) | 1.39 (1.08; 1.79) | 1.03 (0.89; 1.19) | 1.04 (0.59; 1.83) | P+D | . | . | . | . |
| 1.06 (0.57; 1.99) | 1.21 (0.91; 1.60) | 1.23 (0.96; 1.58) | 0.92 (0.67; 1.24) | 1.24 (0.96; 1.60) | 0.92 (0.66; 1.28) | 0.93 (0.53; 1.64) | 0.89 (0.64; 1.24) | R | 1.61 (1.16; 2.25) | . | . |
| 1.64 (0.85; 3.18) | 1.86 (1.27; 2.71) | 1.89 (1.33; 2.70) | 1.41 (0.97; 2.06) | 1.91 (1.33; 2.73) | 1.42 (0.96; 2.10) | 1.43 (0.77; 2.67) | 1.38 (0.93; 2.04) | 1.54 (1.14; 2.08) | R+D | . | . |
| 0.73 (0.38; 1.41) | 0.83 (0.59; 1.15) | 0.84 (0.62; 1.14) | 0.63 (0.44; 0.91) | 0.85 (0.63; 1.15) | 0.63 (0.43; 0.93) | 0.64 (0.36; 1.15) | 0.61 (0.42; 0.90) | 0.69 (0.46; 1.01) | 0.45 (0.28; 0.71) | T | 1.85 (1.25; 2.75) |
| 1.35 (0.63; 2.91) | 1.53 (0.92; 2.56) | 1.56 (0.95; 2.57) | 1.17 (0.68; 2.00) | 1.58 (0.96; 2.59) | 1.17 (0.68; 2.03) | 1.18 (0.58; 2.40) | 1.14 (0.65; 1.97) | 1.27 (0.73; 2.21) | 0.83 (0.45; 1.52) | 1.85 (1.25; 2.75) | T+D |

P-score

R+D 0.9451

T+D 0.7736

P+D 0.7010

G+D 0.6458

O+D 0.6344

P 0.5973

R 0.5273

A+D 0.4380

Do 0.2585

G 0.2181

O 0.1969

T 0.0643

Acute vomiting

| Do | 0.96 (0.80; 1.15) | . | 0.99 (0.85; 1.15) | . | . | . | . | . | . | . |
| --- | --- | --- | --- | --- | --- | --- | --- | --- | --- | --- |
| 0.93 (0.82; 1.05) | G | 1.57 (1.29; 1.91) | 1.09 (0.99; 1.19) | . | 1.61 (0.94; 2.76) | . | 1.32 (0.94; 1.86) | . | 0.91 (0.65; 1.27) | . |
| 1.43 (1.15; 1.77) | 1.54 (1.29; 1.84) | G+D | . | 1.06 (0.88; 1.28) | . | 1.05 (0.81; 1.35) | . | 0.84 (0.48; 1.46) | . | . |
| 1.01 (0.89; 1.14) | 1.09 (1.00; 1.18) | 0.71 (0.58; 0.85) | O | 1.07 (0.68; 1.66) | 1.98 (0.60; 6.52) | . | 1.06 (0.74; 1.52) | . | 0.78 (0.60; 1.03) | . |
| 1.37 (1.07; 1.75) | 1.47 (1.19; 1.83) | 0.96 (0.82; 1.12) | 1.36 (1.08; 1.70) | O+D | . | 1.32 (1.09; 1.60) | . | . | . | . |
| 1.57 (0.95; 2.61) | 1.69 (1.04; 2.77) | 1.10 (0.65; 1.86) | 1.56 (0.95; 2.56) | 1.15 (0.67; 1.97) | P | . | . | . | . | . |
| 1.68 (1.28; 2.21) | 1.82 (1.42; 2.32) | 1.18 (0.98; 1.42) | 1.67 (1.30; 2.15) | 1.23 (1.05; 1.45) | 1.07 (0.62; 1.86) | P+D | . | . | . | . |
| 1.09 (0.84; 1.42) | 1.18 (0.93; 1.50) | 0.77 (0.58; 1.02) | 1.08 (0.85; 1.38) | 0.80 (0.59; 1.09) | 0.70 (0.40; 1.20) | 0.65 (0.47; 0.90) | R | 1.58 (1.06; 2.37) | . | . |
| 1.52 (1.03; 2.24) | 1.64 (1.14; 2.38) | 1.07 (0.73; 1.55) | 1.51 (1.04; 2.20) | 1.12 (0.75; 1.66) | 0.97 (0.53; 1.80) | 0.91 (0.60; 1.37) | 1.39 (0.99; 1.96) | R+D | . | . |
| 0.79 (0.61; 1.03) | 0.86 (0.68; 1.09) | 0.56 (0.41; 0.75) | 0.79 (0.62; 1.00) | 0.58 (0.42; 0.80) | 0.51 (0.29; 0.87) | 0.47 (0.34; 0.66) | 0.73 (0.52; 1.01) | 0.52 (0.34; 0.81) | T | 1.99 (1.42; 2.78) |
| 1.58 (1.03; 2.42) | 1.71 (1.13; 2.57) | 1.11 (0.71; 1.73) | 1.57 (1.04; 2.36) | 1.16 (0.73; 1.84) | 1.01 (0.53; 1.91) | 0.94 (0.58; 1.51) | 1.45 (0.90; 2.32) | 1.04 (0.60; 1.80) | 1.99 (1.42; 2.78) | T+D |

P-score

P+D 0.8831

T+D 0.7767

P 0.7560

R+D 0.7503

G+D 0.6767

O+D 0.6076

R 0.3680

O 0.2825

Do 0.2627

G 0.1160

T 0.0203

③ Acute complete control

| G | 0.80 (0.61; 1.04) | 0.96 (0.89; 1.03) | . | . | . | . | . | 1.16 (0.92; 1.47) | . |
| --- | --- | --- | --- | --- | --- | --- | --- | --- | --- |
| 0.89 (0.75; 1.06) | G+D | . | 0.99 (0.92; 1.06) | . | 0.98 (0.91; 1.05) | . | 1.05 (0.87; 1.27) | . | . |
| 0.95 (0.88; 1.02) | 1.06 (0.89; 1.26) | O | 1.03 (0.83; 1.27) | 0.98 (0.91; 1.05) | . | . | . | 1.13 (0.89; 1.44) | . |
| 0.90 (0.76; 1.07) | 1.01 (0.95; 1.07) | 0.95 (0.80; 1.13) | O+D | . | 0.93 (0.84; 1.03) | . | . | . | . |
| 0.92 (0.83; 1.03) | 1.03 (0.86; 1.25) | 0.98 (0.91; 1.05) | 1.02 (0.85; 1.23) | P | . | . | . | . | . |
| 0.86 (0.72; 1.03) | 0.97 (0.91; 1.03) | 0.91 (0.76; 1.09) | 0.96 (0.89; 1.03) | 0.93 (0.77; 1.13) | P+D | . | . | . | . |
| 1.18 (0.86; 1.64) | 1.33 (1.01; 1.74) | 1.25 (0.91; 1.73) | 1.31 (0.99; 1.73) | 1.28 (0.92; 1.78) | 1.37 (1.04; 1.82) | R | 0.79 (0.65; 0.96) | . | . |
| 0.94 (0.72; 1.22) | 1.05 (0.87; 1.27) | 0.99 (0.77; 1.28) | 1.04 (0.85; 1.27) | 1.02 (0.78; 1.33) | 1.09 (0.89; 1.33) | 0.79 (0.65; 0.96) | R+D | . | . |
| 1.12 (0.90; 1.39) | 1.25 (0.95; 1.65) | 1.18 (0.95; 1.47) | 1.24 (0.94; 1.63) | 1.21 (0.96; 1.53) | 1.30 (0.98; 1.72) | 0.94 (0.64; 1.39) | 1.19 (0.85; 1.67) | T | 0.65 (0.53; 0.80) |
| 0.73 (0.54; 0.98) | 0.81 (0.58; 1.15) | 0.77 (0.57; 1.04) | 0.80 (0.57; 1.13) | 0.79 (0.58; 1.07) | 0.84 (0.60; 1.19) | 0.61 (0.40; 0.95) | 0.77 (0.52; 1.15) | 0.65 (0.53; 0.80) | T+D |

P-score

T+D 0.9297

P+D 0.8040

G+D 0.6426

O+D 0.5924

P 0.5731

R+D 0.4956

O 0.4691

G 0.2739

T 0.1327

R 0.0867

Delayed nausea

| Do | 1.99 (1.29; 3.07) | . | . | 0.91 (0.64; 1.30) | . | . | . | . | . | . | . |
| --- | --- | --- | --- | --- | --- | --- | --- | --- | --- | --- | --- |
| 1.99 (1.29; 3.07) | Do+D | . | . | . | . | . | . | . | . | . | . |
| 0.93 (0.55; 1.59) | 0.47 (0.24; 0.93) | G | 0.97 (0.68; 1.39) | 1.17 (0.68; 2.01) | . | . | . | . | . | . | . |
| 0.98 (0.59; 1.65) | 0.49 (0.25; 0.97) | 1.05 (0.76; 1.45) | G+D | . | 1.07 (0.77; 1.49) | . | 1.40 (1.09; 1.81) | . | 1.02 (0.64; 1.61) | . | . |
| 0.91 (0.64; 1.30) | 0.46 (0.26; 0.80) | 0.98 (0.66; 1.45) | 0.93 (0.64; 1.35) | O | 1.29 (0.88; 1.91) | 1.42 (1.04; 1.95) | . | . | . | 1.21 (0.80; 1.82) | . |
| 1.07 (0.66; 1.75) | 0.54 (0.28; 1.04) | 1.15 (0.80; 1.65) | 1.09 (0.86; 1.37) | 1.18 (0.84; 1.65) | O+D | . | 1.37 (1.06; 1.77) | . | . | . | . |
| 1.30 (0.81; 2.09) | 0.65 (0.34; 1.24) | 1.39 (0.84; 2.30) | 1.32 (0.81; 2.15) | 1.42 (1.04; 1.95) | 1.21 (0.76; 1.92) | P | . | . | . | . | . |
| 1.43 (0.85; 2.41) | 0.72 (0.36; 1.41) | 1.53 (1.05; 2.21) | 1.45 (1.17; 1.79) | 1.56 (1.07; 2.29) | 1.33 (1.07; 1.65) | 1.10 (0.67; 1.80) | P+D | . | . | . | . |
| 0.65 (0.30; 1.43) | 0.33 (0.13; 0.80) | 0.70 (0.36; 1.37) | 0.66 (0.37; 1.20) | 0.71 (0.35; 1.44) | 0.61 (0.32; 1.15) | 0.50 (0.23; 1.08) | 0.46 (0.24; 0.86) | R | 1.53 (1.06; 2.23) | . | . |
| 1.00 (0.50; 1.99) | 0.50 (0.22; 1.14) | 1.07 (0.61; 1.87) | 1.02 (0.64; 1.61) | 1.09 (0.61; 1.98) | 0.93 (0.56; 1.56) | 0.77 (0.39; 1.50) | 0.70 (0.42; 1.16) | 1.53 (1.06; 2.23) | R+D | . | . |
| 1.10 (0.64; 1.89) | 0.55 (0.28; 1.11) | 1.18 (0.67; 2.08) | 1.12 (0.64; 1.95) | 1.21 (0.80; 1.82) | 1.03 (0.60; 1.75) | 0.85 (0.51; 1.42) | 0.77 (0.44; 1.35) | 1.69 (0.75; 3.80) | 1.10 (0.54; 2.26) | T | 1.49 (1.00; 2.20) |
| 1.64 (0.84; 3.20) | 0.82 (0.37; 1.83) | 1.75 (0.88; 3.50) | 1.66 (0.84; 3.28) | 1.79 (1.02; 3.16) | 1.53 (0.79; 2.95) | 1.26 (0.66; 2.41) | 1.15 (0.58; 2.27) | 2.51 (1.02; 6.18) | 1.64 (0.72; 3.72) | 1.49 (1.00; 2.20) | T+D |

P-score

Do+D 0.9310

T+D 0.8397

P+D 0.7965

P 0.6890

T 0.4898

O+D 0.4842

R+D 0.4076

Do 0.3846

G+D 0.3585

G 0.2986

O 0.2476

R 0.0728

Delayed vomiting

| Do | 2.33 (1.55; 3.52) | . | . | 1.27 (0.80; 2.02) | . | . | . | . | . | . | . |
| --- | --- | --- | --- | --- | --- | --- | --- | --- | --- | --- | --- |
| 2.33 (1.55; 3.52) | Do+D | . | . | . | . | . | . | . | . | . | . |
| 1.34 (0.78; 2.29) | 0.57 (0.29; 1.13) | G | 0.93 (0.62; 1.40) | 1.09 (0.77; 1.55) | . | 1.12 (0.73; 1.71) | . | . | . | 1.14 (0.67; 1.96) | . |
| 1.29 (0.72; 2.30) | 0.55 (0.27; 1.13) | 0.96 (0.69; 1.34) | G+D | . | 0.99 (0.70; 1.41) | . | 1.59 (1.20; 2.12) | . | 0.97 (0.55; 1.73) | . | . |
| 1.27 (0.80; 2.02) | 0.54 (0.29; 1.01) | 0.95 (0.73; 1.24) | 0.99 (0.69; 1.40) | O | 1.17 (0.77; 1.78) | 1.88 (1.17; 3.02) | . | . | . | 0.97 (0.71; 1.33) | . |
| 1.43 (0.80; 2.53) | 0.61 (0.30; 1.24) | 1.07 (0.75; 1.51) | 1.11 (0.88; 1.40) | 1.12 (0.80; 1.57) | O+D | . | 1.33 (1.10; 1.60) | . | . | . | . |
| 1.84 (1.03; 3.30) | 0.79 (0.39; 1.61) | 1.38 (0.98; 1.93) | 1.43 (0.92; 2.23) | 1.45 (1.02; 2.06) | 1.29 (0.82; 2.02) | P | . | . | . | . | . |
| 1.94 (1.08; 3.49) | 0.83 (0.41; 1.70) | 1.45 (1.01; 2.08) | 1.51 (1.20; 1.89) | 1.53 (1.07; 2.19) | 1.36 (1.15; 1.61) | 1.05 (0.66; 1.67) | P+D | . | . | . | . |
| 0.77 (0.31; 1.91) | 0.33 (0.12; 0.89) | 0.58 (0.27; 1.24) | 0.60 (0.30; 1.20) | 0.61 (0.28; 1.32) | 0.54 (0.26; 1.12) | 0.42 (0.18; 0.95) | 0.40 (0.19; 0.82) | R | 1.63 (1.10; 2.40) | . | . |
| 1.25 (0.55; 2.84) | 0.54 (0.22; 1.34) | 0.94 (0.48; 1.82) | 0.97 (0.55; 1.73) | 0.99 (0.50; 1.93) | 0.88 (0.47; 1.63) | 0.68 (0.33; 1.41) | 0.65 (0.35; 1.20) | 1.63 (1.10; 2.40) | R+D | . | . |
| 1.29 (0.74; 2.25) | 0.55 (0.28; 1.10) | 0.96 (0.67; 1.39) | 1.00 (0.64; 1.56) | 1.02 (0.75; 1.37) | 0.90 (0.58; 1.40) | 0.70 (0.45; 1.09) | 0.67 (0.42; 1.05) | 1.68 (0.74; 3.82) | 1.03 (0.50; 2.13) | T | 1.59 (1.10; 2.28) |
| 2.05 (1.06; 3.97) | 0.88 (0.40; 1.91) | 1.53 (0.92; 2.56) | 1.59 (0.90; 2.81) | 1.61 (1.01; 2.58) | 1.43 (0.81; 2.53) | 1.11 (0.63; 1.97) | 1.06 (0.59; 1.88) | 2.66 (1.08; 6.53) | 1.63 (0.73; 3.67) | 1.59 (1.10; 2.28) | T+D |

P-score

Do+D 0.8813

T+D 0.8355

P+D 0.8317

P 0.7760

O+D 0.5190

G 0.4260

R+D 0.3923

T 0.3803

G+D 0.3690

O 0.3506

Do 0.1686

R 0.0696

Delayed complete control

| G | 1.12 (0.73; 1.71) | . | . | . | . | . |
| --- | --- | --- | --- | --- | --- | --- |
| 1.12 (0.73; 1.71) | G+D | . | 0.94 (0.72; 1.23) | . | 0.80 (0.62; 1.04) | 0.92 (0.57; 1.50) |
| 1.89 (1.00; 3.56) | 1.69 (1.06; 2.71) | O | 0.59 (0.39; 0.90) | 0.70 (0.48; 1.02) | . | . |
| 1.12 (0.70; 1.81) | 1.01 (0.81; 1.24) | 0.59 (0.39; 0.90) | O+D | . | 0.70 (0.55; 0.90) | . |
| 1.32 (0.63; 2.76) | 1.18 (0.65; 2.16) | 0.70 (0.48; 1.02) | 1.18 (0.67; 2.07) | P | . | . |
| 0.84 (0.52; 1.35) | 0.75 (0.61; 0.93) | 0.44 (0.28; 0.71) | 0.75 (0.61; 0.91) | 0.63 (0.35; 1.16) | P+D | . |
| 1.03 (0.54; 1.97) | 0.92 (0.57; 1.50) | 0.55 (0.28; 1.07) | 0.92 (0.54; 1.56) | 0.78 (0.36; 1.70) | 1.23 (0.73; 2.09) | R+D |

P-score

P+D 0.9122

G 0.6478

R+D 0.6035

G+D 0.4828

O+D 0.4811

P 0.3529

O 0.0197

15、The outcomes after the article of “Kang YK 2002” was deleted.

①Acute nausea

| A+D | . | . | 1.16 (0.67; 2.00) | . | . | . | . | . | . | . | . |
| --- | --- | --- | --- | --- | --- | --- | --- | --- | --- | --- | --- |
| 0.90 (0.50; 1.62) | Do | 0.98 (0.85; 1.12) | . | 0.99 (0.68; 1.42) | . | . | . | . | . | . | . |
| 0.88 (0.49; 1.57) | 0.98 (0.86; 1.12) | G | 1.32 (1.06; 1.63) | 1.00 (0.93; 1.08) | . | . | . | . | . | 0.41 (0.23; 0.73) | . |
| 1.16 (0.67; 2.00) | 1.29 (1.02; 1.63) | 1.32 (1.09; 1.60) | G+D | . | 1.04 (0.91; 1.19) | . | 0.97 (0.84; 1.12) | . | 1.22 (0.66; 2.23) | . | . |
| 0.87 (0.49; 1.56) | 0.97 (0.84; 1.12) | 0.99 (0.92; 1.06) | 0.75 (0.62; 0.92) | O | 1.26 (0.74; 2.13) | 1.33 (0.80; 2.20) | . | 1.16 (0.72; 1.86) | . | 1.10 (0.78; 1.56) | . |
| 1.15 (0.66; 2.01) | 1.28 (1.00; 1.65) | 1.31 (1.05; 1.62) | 0.99 (0.89; 1.11) | 1.32 (1.06; 1.65) | O+D | . | 1.14 (0.93; 1.38) | . | . | . | . |
| 1.16 (0.54; 2.51) | 1.29 (0.77; 2.18) | 1.32 (0.79; 2.19) | 1.00 (0.58; 1.72) | 1.33 (0.80; 2.20) | 1.01 (0.58; 1.75) | P | . | . | . | . | . |
| 1.19 (0.68; 2.08) | 1.32 (1.02; 1.72) | 1.35 (1.08; 1.69) | 1.02 (0.91; 1.16) | 1.36 (1.08; 1.72) | 1.03 (0.90; 1.18) | 1.02 (0.59; 1.78) | P+D | . | . | . | . |
| 0.96 (0.49; 1.91) | 1.07 (0.71; 1.63) | 1.09 (0.73; 1.63) | 0.83 (0.55; 1.26) | 1.10 (0.74; 1.64) | 0.84 (0.55; 1.28) | 0.83 (0.44; 1.58) | 0.81 (0.53; 1.25) | R | 1.61 (1.16; 2.24) | . | . |
| 1.52 (0.76; 3.04) | 1.69 (1.08; 2.65) | 1.73 (1.12; 2.65) | 1.31 (0.85; 2.02) | 1.74 (1.13; 2.67) | 1.32 (0.85; 2.05) | 1.31 (0.68; 2.54) | 1.28 (0.82; 2.00) | 1.58 (1.16; 2.14) | R+D | . | . |
| 0.74 (0.39; 1.42) | 0.83 (0.60; 1.15) | 0.84 (0.62; 1.14) | 0.64 (0.45; 0.92) | 0.85 (0.63; 1.15) | 0.64 (0.45; 0.93) | 0.64 (0.36; 1.15) | 0.63 (0.43; 0.91) | 0.77 (0.47; 1.27) | 0.49 (0.29; 0.82) | T | 1.85 (1.25; 2.74) |
| 1.38 (0.65; 2.95) | 1.54 (0.92; 2.56) | 1.57 (0.96; 2.57) | 1.19 (0.70; 2.02) | 1.58 (0.97; 2.58) | 1.20 (0.70; 2.05) | 1.19 (0.59; 2.40) | 1.16 (0.67; 2.00) | 1.43 (0.76; 2.69) | 0.91 (0.47; 1.74) | 1.85 (1.25; 2.74) | T+D |

P-score

R+D 0.8995

T+D 0.7996

P+D 0.7098

G+D 0.6593

O+D 0.6440

P 0.6238

A+D 0.4440

R 0.3889

Do 0.2873

G 0.2472

O 0.2195

T 0.0769

②Acute vomiting

| Do | 0.96 (0.80; 1.15) | . | 0.99 (0.85; 1.15) | . | . | . | . | . | . | . |
| --- | --- | --- | --- | --- | --- | --- | --- | --- | --- | --- |
| 0.93 (0.82; 1.06) | G | 1.57 (1.29; 1.91) | 1.09 (0.99; 1.19) | . | 1.61 (0.94; 2.77) | . | . | . | 0.91 (0.65; 1.27) | . |
| 1.45 (1.17; 1.79) | 1.56 (1.31; 1.85) | G+D | . | 1.06 (0.88; 1.28) | . | 1.05 (0.81; 1.36) | . | 0.84 (0.48; 1.46) | . | . |
| 1.00 (0.89; 1.13) | 1.08 (0.99; 1.17) | 0.69 (0.58; 0.83) | O | 1.28 (0.88; 1.85) | 1.98 (0.60; 6.52) | . | 1.06 (0.74; 1.52) | . | 0.78 (0.60; 1.03) | . |
| 1.41 (1.11; 1.79) | 1.51 (1.23; 1.86) | 0.97 (0.84; 1.13) | 1.40 (1.13; 1.74) | O+D | . | 1.32 (1.09; 1.60) | . | . | . | . |
| 1.58 (0.95; 2.62) | 1.69 (1.03; 2.77) | 1.09 (0.64; 1.83) | 1.57 (0.96; 2.58) | 1.12 (0.66; 1.91) | P | . | . | . | . | . |
| 1.73 (1.32; 2.25) | 1.85 (1.46; 2.35) | 1.19 (0.99; 1.43) | 1.72 (1.35; 2.20) | 1.23 (1.04; 1.44) | 1.10 (0.63; 1.89) | P+D | . | . | . | . |
| 0.99 (0.70; 1.40) | 1.06 (0.77; 1.48) | 0.68 (0.48; 0.97) | 0.99 (0.72; 1.36) | 0.70 (0.49; 1.02) | 0.63 (0.35; 1.14) | 0.57 (0.39; 0.85) | R | 1.58 (1.06; 2.37) | . | . |
| 1.44 (0.95; 2.18) | 1.54 (1.03; 2.30) | 0.99 (0.67; 1.48) | 1.43 (0.96; 2.13) | 1.02 (0.67; 1.55) | 0.91 (0.48; 1.72) | 0.83 (0.54; 1.29) | 1.45 (1.02; 2.06) | R+D | . | . |
| 0.79 (0.61; 1.03) | 0.85 (0.67; 1.08) | 0.55 (0.41; 0.73) | 0.79 (0.63; 1.00) | 0.56 (0.41; 0.77) | 0.50 (0.29; 0.87) | 0.46 (0.33; 0.64) | 0.80 (0.54; 1.19) | 0.55 (0.35; 0.87) | T | 1.99 (1.42; 2.79) |
| 1.58 (1.03; 2.42) | 1.70 (1.12; 2.56) | 1.09 (0.70; 1.70) | 1.58 (1.05; 2.37) | 1.12 (0.71; 1.77) | 1.00 (0.53; 1.90) | 0.92 (0.57; 1.47) | 1.59 (0.95; 2.68) | 1.10 (0.62; 1.94) | 1.99 (1.42; 2.79) | T+D |

P-score

P+D 0.9025

T+D 0.7751

P 0.7597

G+D 0.6912

R+D 0.6867

O+D 0.6431

O 0.3077

Do 0.2918

R 0.2629

G 0.1487

T 0.0307

16、The outcomes after the article of “Keyhanian Sh 2009” was deleted.

Acute nausea

| A+D | . | . | 1.16 (0.67; 2.00) | . | . | . | . | . | . | . | . |
| --- | --- | --- | --- | --- | --- | --- | --- | --- | --- | --- | --- |
| 0.90 (0.49; 1.64) | Do | 0.98 (0.85; 1.13) | . | 0.99 (0.68; 1.43) | . | . | . | . | . | . | . |
| 0.88 (0.49; 1.59) | 0.98 (0.86; 1.12) | G | 1.30 (0.99; 1.70) | 1.00 (0.93; 1.08) | . | . | . | 1.32 (0.96; 1.82) | . | 0.41 (0.23; 0.73) | . |
| 1.16 (0.67; 2.00) | 1.30 (0.99; 1.69) | 1.32 (1.05; 1.66) | G+D | . | 1.04 (0.91; 1.19) | . | 0.97 (0.83; 1.13) | . | 1.22 (0.66; 2.23) | . | . |
| 0.87 (0.48; 1.58) | 0.97 (0.84; 1.13) | 0.99 (0.92; 1.07) | 0.75 (0.59; 0.95) | O | 1.26 (0.74; 2.13) | 1.33 (0.80; 2.21) | . | 1.16 (0.72; 1.86) | . | 1.10 (0.78; 1.56) | . |
| 1.15 (0.66; 2.01) | 1.29 (0.97; 1.70) | 1.31 (1.02; 1.68) | 0.99 (0.88; 1.12) | 1.32 (1.03; 1.70) | O+D | . | 1.14 (0.93; 1.39) | . | . | . | . |
| 1.16 (0.53; 2.53) | 1.30 (0.77; 2.19) | 1.32 (0.79; 2.20) | 1.00 (0.57; 1.75) | 1.33 (0.80; 2.21) | 1.01 (0.57; 1.77) | P | . | . | . | . | . |
| 1.19 (0.68; 2.09) | 1.33 (0.99; 1.78) | 1.36 (1.05; 1.76) | 1.03 (0.90; 1.17) | 1.37 (1.05; 1.78) | 1.03 (0.90; 1.19) | 1.03 (0.58; 1.82) | P+D | . | . | . | . |
| 1.08 (0.57; 2.03) | 1.20 (0.91; 1.60) | 1.23 (0.96; 1.58) | 0.93 (0.68; 1.28) | 1.24 (0.96; 1.59) | 0.93 (0.67; 1.30) | 0.93 (0.53; 1.63) | 0.90 (0.64; 1.27) | R | 1.61 (1.16; 2.25) | . | . |
| 1.66 (0.85; 3.22) | 1.85 (1.26; 2.70) | 1.89 (1.32; 2.69) | 1.43 (0.97; 2.09) | 1.90 (1.33; 2.72) | 1.44 (0.97; 2.13) | 1.43 (0.77; 2.65) | 1.39 (0.93; 2.07) | 1.54 (1.14; 2.08) | R+D | . | . |
| 0.74 (0.38; 1.44) | 0.83 (0.59; 1.15) | 0.84 (0.62; 1.14) | 0.64 (0.44; 0.93) | 0.85 (0.63; 1.15) | 0.64 (0.44; 0.95) | 0.64 (0.35; 1.15) | 0.62 (0.42; 0.93) | 0.69 (0.47; 1.02) | 0.45 (0.28; 0.71) | T | 1.85 (1.25; 2.75) |
| 1.37 (0.64; 2.97) | 1.53 (0.92; 2.56) | 1.57 (0.95; 2.57) | 1.18 (0.69; 2.04) | 1.58 (0.96; 2.59) | 1.19 (0.69; 2.07) | 1.18 (0.58; 2.40) | 1.15 (0.66; 2.02) | 1.28 (0.73; 2.22) | 0.83 (0.45; 1.53) | 1.85 (1.25; 2.75) | T+D |

P-score

R+D 0.9454

T+D 0.7798

P+D 0.6922

G+D 0.6385

O+D 0.6212

P 0.6057

R 0.5367

A+D 0.4279

Do 0.2634

G 0.2208

O 0.2017

T 0.0666

Acute vomiting

| Do | 0.96 (0.79; 1.15) | . | 0.99 (0.85; 1.15) | . | . | . | . | . | . | . |
| --- | --- | --- | --- | --- | --- | --- | --- | --- | --- | --- |
| 0.93 (0.82; 1.06) | G | 1.52 (1.22; 1.90) | 1.09 (0.99; 1.19) | . | 1.61 (0.94; 2.77) | . | 1.32 (0.93; 1.87) | . | 0.91 (0.65; 1.27) | . |
| 1.43 (1.14; 1.79) | 1.53 (1.27; 1.85) | G+D | . | 1.06 (0.88; 1.28) | . | 1.05 (0.81; 1.36) | . | 0.84 (0.48; 1.46) | . | . |
| 1.01 (0.89; 1.14) | 1.08 (0.99; 1.18) | 0.71 (0.58; 0.86) | O | 1.28 (0.88; 1.85) | 1.98 (0.60; 6.53) | . | 1.06 (0.74; 1.52) | . | 0.78 (0.60; 1.03) | . |
| 1.39 (1.08; 1.78) | 1.49 (1.20; 1.86) | 0.97 (0.84; 1.13) | 1.38 (1.10; 1.73) | O+D | . | 1.32 (1.08; 1.60) | . | . | . | . |
| 1.57 (0.95; 2.62) | 1.69 (1.03; 2.77) | 1.10 (0.65; 1.87) | 1.56 (0.95; 2.57) | 1.13 (0.66; 1.94) | P | . | . | . | . | . |
| 1.70 (1.29; 2.24) | 1.83 (1.42; 2.35) | 1.19 (0.99; 1.43) | 1.69 (1.31; 2.18) | 1.23 (1.04; 1.44) | 1.08 (0.62; 1.88) | P+D | . | . | . | . |
| 1.09 (0.84; 1.42) | 1.18 (0.93; 1.49) | 0.77 (0.57; 1.02) | 1.09 (0.85; 1.38) | 0.79 (0.58; 1.07) | 0.69 (0.40; 1.20) | 0.64 (0.46; 0.90) | R | 1.58 (1.05; 2.38) | . | . |
| 1.52 (1.03; 2.25) | 1.64 (1.13; 2.38) | 1.07 (0.73; 1.56) | 1.51 (1.04; 2.20) | 1.10 (0.74; 1.64) | 0.97 (0.52; 1.79) | 0.90 (0.59; 1.36) | 1.39 (0.99; 1.96) | R+D | . | . |
| 0.79 (0.61; 1.03) | 0.85 (0.67; 1.08) | 0.56 (0.41; 0.75) | 0.79 (0.62; 1.00) | 0.57 (0.42; 0.79) | 0.50 (0.29; 0.87) | 0.47 (0.33; 0.66) | 0.73 (0.52; 1.01) | 0.52 (0.34; 0.81) | T | 1.99 (1.42; 2.79) |
| 1.58 (1.03; 2.42) | 1.70 (1.13; 2.57) | 1.11 (0.71; 1.74) | 1.57 (1.04; 2.37) | 1.14 (0.72; 1.81) | 1.01 (0.53; 1.91) | 0.93 (0.58; 1.50) | 1.45 (0.90; 2.32) | 1.04 (0.60; 1.80) | 1.99 (1.42; 2.79) | T+D |

P-score

P+D 0.8882

T+D 0.7722

P 0.7543

R+D 0.7449

G+D 0.6682

O+D 0.6238

R 0.3668

O 0.2800

Do 0.2623

G 0.1191

T 0.0202

17、The outcomes after the article of “Kim JS 2004” was deleted.

Acute nausea

| A+D | . | . | 1.16 (0.67; 2.00) | . | . | . | . | . | . | . | . |
| --- | --- | --- | --- | --- | --- | --- | --- | --- | --- | --- | --- |
| 0.89 (0.49; 1.62) | Do | 0.98 (0.85; 1.13) | . | . | . | . | . | . | . | . | . |
| 0.87 (0.49; 1.56) | 0.98 (0.85; 1.13) | G | 1.32 (1.06; 1.63) | 1.00 (0.93; 1.08) | . | . | . | 1.32 (0.96; 1.82) | . | 0.41 (0.23; 0.73) | . |
| 1.16 (0.67; 2.00) | 1.30 (1.02; 1.65) | 1.33 (1.10; 1.61) | G+D | . | 1.04 (0.91; 1.19) | . | 0.97 (0.83; 1.13) | . | 1.22 (0.66; 2.23) | . | . |
| 0.87 (0.48; 1.55) | 0.97 (0.83; 1.14) | 0.99 (0.92; 1.07) | 0.75 (0.61; 0.91) | O | 1.26 (0.74; 2.13) | 1.33 (0.80; 2.21) | . | 1.16 (0.72; 1.86) | . | 1.10 (0.78; 1.56) | . |
| 1.15 (0.66; 2.01) | 1.29 (1.00; 1.67) | 1.32 (1.06; 1.64) | 0.99 (0.88; 1.12) | 1.33 (1.06; 1.67) | O+D | . | 1.14 (0.93; 1.39) | . | . | . | . |
| 1.15 (0.53; 2.49) | 1.29 (0.76; 2.20) | 1.32 (0.79; 2.20) | 0.99 (0.58; 1.71) | 1.33 (0.80; 2.21) | 1.00 (0.58; 1.74) | P | . | . | . | . | . |
| 1.19 (0.68; 2.09) | 1.34 (1.02; 1.75) | 1.36 (1.09; 1.72) | 1.03 (0.90; 1.17) | 1.37 (1.08; 1.74) | 1.03 (0.90; 1.19) | 1.03 (0.59; 1.80) | P+D | . | . | . | . |
| 1.07 (0.58; 2.00) | 1.20 (0.90; 1.60) | 1.23 (0.96; 1.58) | 0.92 (0.68; 1.25) | 1.24 (0.96; 1.59) | 0.93 (0.68; 1.28) | 0.93 (0.53; 1.64) | 0.90 (0.65; 1.24) | R | 1.61 (1.16; 2.25) | . | . |
| 1.65 (0.85; 3.19) | 1.85 (1.26; 2.71) | 1.89 (1.33; 2.69) | 1.42 (0.98; 2.06) | 1.90 (1.33; 2.72) | 1.43 (0.97; 2.11) | 1.43 (0.77; 2.66) | 1.38 (0.94; 2.05) | 1.54 (1.14; 2.08) | R+D | . | . |
| 0.74 (0.38; 1.41) | 0.83 (0.59; 1.15) | 0.84 (0.62; 1.14) | 0.63 (0.44; 0.91) | 0.85 (0.63; 1.15) | 0.64 (0.44; 0.93) | 0.64 (0.35; 1.15) | 0.62 (0.42; 0.90) | 0.69 (0.47; 1.02) | 0.45 (0.28; 0.71) | T | 1.85 (1.25; 2.75) |
| 1.37 (0.64; 2.93) | 1.53 (0.91; 2.57) | 1.56 (0.95; 2.57) | 1.18 (0.69; 2.00) | 1.58 (0.96; 2.59) | 1.19 (0.69; 2.04) | 1.18 (0.58; 2.40) | 1.15 (0.66; 1.98) | 1.27 (0.73; 2.22) | 0.83 (0.45; 1.52) | 1.85 (1.25; 2.75) | T+D |

P-score

R+D 0.9462

T+D 0.7785

P+D 0.6975

G+D 0.6443

O+D 0.6265

P 0.6022

R 0.5306

A+D 0.4324

Do 0.2608

G 0.2177

O 0.1984

T 0.0648

Acute vomiting

| Do | 0.96 (0.80; 1.15) | . | 0.97 (0.83; 1.13) | . | . | . | . | . | . | . |
| --- | --- | --- | --- | --- | --- | --- | --- | --- | --- | --- |
| 0.92 (0.81; 1.05) | G | 1.57 (1.29; 1.91) | 1.09 (0.99; 1.19) | . | 1.61 (0.94; 2.76) | . | 1.32 (0.94; 1.86) | . | 0.91 (0.65; 1.27) | . |
| 1.44 (1.17; 1.78) | 1.57 (1.32; 1.86) | G+D | . | 1.06 (0.88; 1.28) | . | 1.05 (0.81; 1.35) | . | 0.84 (0.48; 1.46) | . | . |
| 0.99 (0.88; 1.13) | 1.08 (0.99; 1.17) | 0.69 (0.57; 0.83) | O | 1.28 (0.88; 1.85) | 1.98 (0.60; 6.52) | . | 1.06 (0.74; 1.52) | . | 0.78 (0.60; 1.03) | . |
| 1.40 (1.10; 1.78) | 1.52 (1.23; 1.87) | 0.97 (0.84; 1.13) | 1.41 (1.14; 1.74) | O+D | . | 1.32 (1.09; 1.60) | . | . | . | . |
| 1.56 (0.94; 2.58) | 1.69 (1.03; 2.77) | 1.08 (0.64; 1.82) | 1.57 (0.95; 2.57) | 1.11 (0.65; 1.90) | P | . | . | . | . | . |
| 1.72 (1.31; 2.24) | 1.86 (1.47; 2.37) | 1.19 (0.99; 1.43) | 1.73 (1.35; 2.20) | 1.23 (1.04; 1.44) | 1.10 (0.64; 1.90) | P+D | . | . | . | . |
| 1.08 (0.83; 1.41) | 1.18 (0.93; 1.49) | 0.75 (0.57; 0.99) | 1.09 (0.86; 1.39) | 0.78 (0.57; 1.05) | 0.70 (0.40; 1.20) | 0.63 (0.46; 0.87) | R | 1.58 (1.06; 2.37) | . | . |
| 1.52 (1.03; 2.24) | 1.65 (1.14; 2.39) | 1.06 (0.73; 1.53) | 1.53 (1.06; 2.22) | 1.09 (0.73; 1.62) | 0.98 (0.53; 1.81) | 0.89 (0.59; 1.34) | 1.40 (1.00; 1.97) | R+D | . | . |
| 0.79 (0.61; 1.02) | 0.85 (0.67; 1.08) | 0.54 (0.41; 0.73) | 0.79 (0.63; 1.00) | 0.56 (0.41; 0.77) | 0.50 (0.29; 0.87) | 0.46 (0.33; 0.64) | 0.72 (0.52; 1.01) | 0.52 (0.33; 0.80) | T | 1.99 (1.42; 2.78) |
| 1.56 (1.02; 2.39) | 1.70 (1.13; 2.56) | 1.08 (0.70; 1.69) | 1.57 (1.05; 2.37) | 1.12 (0.71; 1.77) | 1.00 (0.53; 1.91) | 0.91 (0.57; 1.46) | 1.44 (0.90; 2.31) | 1.03 (0.59; 1.78) | 1.99 (1.42; 2.78) | T+D |

P-score

P+D 0.8960

T+D 0.7616

P 0.7447

R+D 0.7430

G+D 0.6797

O+D 0.6302

R 0.3635

Do 0.2757

O 0.2709

G 0.1155

T 0.0190

Delayed nausea：The network disconnection has made a comparison impossible.

Delayed vomiting： The network disconnection has made a comparison impossible.

18、The outcomes after the article of “Latreille J 1995” was deleted.

Acute vomiting

| Do | 0.96 (0.80; 1.15) | . | 0.99 (0.85; 1.15) | . | . | . | . | . | . | . |
| --- | --- | --- | --- | --- | --- | --- | --- | --- | --- | --- |
| 0.93 (0.82; 1.06) | G | 1.45 (1.10; 1.91) | 1.09 (0.99; 1.19) | . | 1.61 (0.94; 2.77) | . | 1.32 (0.94; 1.87) | . | 0.91 (0.65; 1.27) | . |
| 1.39 (1.09; 1.79) | 1.50 (1.20; 1.86) | G+D | . | 1.06 (0.88; 1.28) | . | 1.05 (0.81; 1.36) | . | 0.84 (0.48; 1.46) | . | . |
| 1.01 (0.89; 1.14) | 1.08 (0.99; 1.18) | 0.72 (0.58; 0.90) | O | 1.28 (0.88; 1.85) | 1.98 (0.60; 6.53) | . | 1.06 (0.74; 1.52) | . | 0.78 (0.60; 1.03) | . |
| 1.36 (1.04; 1.77) | 1.46 (1.15; 1.86) | 0.98 (0.84; 1.14) | 1.35 (1.06; 1.72) | O+D | . | 1.32 (1.08; 1.60) | . | . | . | . |
| 1.57 (0.95; 2.62) | 1.69 (1.03; 2.77) | 1.13 (0.66; 1.94) | 1.57 (0.95; 2.58) | 1.16 (0.67; 2.00) | P | . | . | . | . | . |
| 1.66 (1.24; 2.23) | 1.79 (1.37; 2.34) | 1.20 (1.00; 1.44) | 1.66 (1.26; 2.17) | 1.22 (1.04; 1.44) | 1.06 (0.60; 1.85) | P+D | . | . | . | . |
| 1.09 (0.84; 1.42) | 1.17 (0.92; 1.49) | 0.78 (0.58; 1.06) | 1.08 (0.85; 1.38) | 0.80 (0.58; 1.10) | 0.69 (0.40; 1.20) | 0.66 (0.47; 0.92) | R | 1.58 (1.06; 2.38) | . | . |
| 1.51 (1.02; 2.23) | 1.62 (1.11; 2.36) | 1.08 (0.74; 1.59) | 1.50 (1.03; 2.19) | 1.11 (0.74; 1.66) | 0.96 (0.52; 1.78) | 0.91 (0.60; 1.38) | 1.38 (0.98; 1.95) | R+D | . | . |
| 0.79 (0.61; 1.03) | 0.85 (0.67; 1.08) | 0.57 (0.41; 0.79) | 0.79 (0.62; 1.00) | 0.58 (0.42; 0.81) | 0.50 (0.29; 0.87) | 0.48 (0.33; 0.68) | 0.73 (0.52; 1.01) | 0.53 (0.34; 0.82) | T | 1.99 (1.42; 2.79) |
| 1.58 (1.03; 2.42) | 1.70 (1.13; 2.57) | 1.14 (0.71; 1.81) | 1.57 (1.04; 2.37) | 1.16 (0.73; 1.86) | 1.00 (0.53; 1.91) | 0.95 (0.58; 1.55) | 1.45 (0.90; 2.33) | 1.05 (0.60; 1.82) | 1.99 (1.42; 2.79) | T+D |

P-score

P+D 0.8791

T+D 0.7831

P 0.7646

R+D 0.7477

G+D 0.6544

O+D 0.6157

R 0.3699

O 0.2807

Do 0.2645

G 0.1202

T 0.0202

19、The outcomes after the article of “Mahrous MA 2021” was deleted.

Delayed nausea

| Do | 1.99 (1.37; 2.89) | . | . | 0.91 (0.69; 1.21) | . | . | . | . | . | . | . |
| --- | --- | --- | --- | --- | --- | --- | --- | --- | --- | --- | --- |
| 1.99 (1.37; 2.89) | Do+D | . | . | . | . | . | . | . | . | . | . |
| 1.06 (0.69; 1.63) | 0.53 (0.30; 0.94) | G | 0.97 (0.73; 1.29) | 1.17 (0.72; 1.92) | . | . | . | . | . | . | . |
| 1.14 (0.76; 1.71) | 0.57 (0.33; 0.99) | 1.08 (0.83; 1.39) | G+D | . | 1.07 (0.84; 1.37) | . | 1.17 (0.93; 1.47) | . | 1.02 (0.68; 1.52) | . | . |
| 0.91 (0.69; 1.21) | 0.46 (0.29; 0.73) | 0.86 (0.62; 1.20) | 0.80 (0.60; 1.07) | O | 1.41 (1.07; 1.85) | 1.43 (1.07; 1.90) | . | . | . | 1.21 (0.86; 1.70) | . |
| 1.17 (0.80; 1.70) | 0.59 (0.35; 1.00) | 1.10 (0.82; 1.48) | 1.03 (0.85; 1.23) | 1.28 (1.00; 1.64) | O+D | . | 1.37 (1.12; 1.67) | . | . | . | . |
| 1.30 (0.87; 1.94) | 0.66 (0.38; 1.13) | 1.23 (0.80; 1.90) | 1.14 (0.76; 1.71) | 1.43 (1.07; 1.90) | 1.11 (0.76; 1.63) | P | . | . | . | . | . |
| 1.48 (0.99; 2.22) | 0.74 (0.43; 1.29) | 1.40 (1.03; 1.89) | 1.30 (1.08; 1.56) | 1.62 (1.21; 2.17) | 1.26 (1.07; 1.50) | 1.13 (0.75; 1.71) | P+D | . | . | . | . |
| 0.76 (0.40; 1.44) | 0.38 (0.18; 0.80) | 0.71 (0.41; 1.25) | 0.66 (0.40; 1.09) | 0.83 (0.46; 1.48) | 0.65 (0.38; 1.10) | 0.58 (0.30; 1.10) | 0.51 (0.30; 0.87) | R | 1.53 (1.13; 2.07) | . | . |
| 1.16 (0.66; 2.05) | 0.58 (0.30; 1.15) | 1.09 (0.68; 1.76) | 1.02 (0.68; 1.52) | 1.27 (0.77; 2.08) | 0.99 (0.64; 1.54) | 0.89 (0.50; 1.57) | 0.78 (0.50; 1.22) | 1.53 (1.13; 2.07) | R+D | . | . |
| 1.10 (0.71; 1.72) | 0.55 (0.31; 0.99) | 1.04 (0.65; 1.67) | 0.97 (0.62; 1.51) | 1.21 (0.86; 1.70) | 0.94 (0.62; 1.44) | 0.85 (0.54; 1.32) | 0.74 (0.47; 1.17) | 1.46 (0.74; 2.86) | 0.95 (0.52; 1.74) | T | 1.49 (1.07; 2.06) |
| 1.64 (0.94; 2.84) | 0.82 (0.42; 1.60) | 1.55 (0.87; 2.75) | 1.44 (0.82; 2.50) | 1.79 (1.12; 2.88) | 1.40 (0.82; 2.39) | 1.26 (0.72; 2.18) | 1.11 (0.63; 1.93) | 2.17 (1.03; 4.58) | 1.41 (0.71; 2.80) | 1.49 (1.07; 2.06) | T+D |

P-score

Do+D 0.9412

T+D 0.8361

P+D 0.8126

P 0.6457

O+D 0.5109

R+D 0.4942

G+D 0.4674

T 0.4165

G 0.3551

Do 0.2917

O 0.1460

R 0.0825

Delayed vomiting

| Do | 2.33 (1.75; 3.10) | . | . | 1.27 (0.89; 1.82) | . | . | . | . | . | . | . |
| --- | --- | --- | --- | --- | --- | --- | --- | --- | --- | --- | --- |
| 2.33 (1.75; 3.10) | Do+D | . | . | . | . | . | . | . | . | . | . |
| 1.41 (0.93; 2.13) | 0.60 (0.37; 1.00) | G | 0.93 (0.70; 1.23) | 1.09 (0.82; 1.44) | . | 1.12 (0.82; 1.52) | . | . | . | 1.14 (0.73; 1.79) | . |
| 1.45 (0.95; 2.23) | 0.62 (0.37; 1.04) | 1.03 (0.82; 1.29) | G+D | . | 0.99 (0.82; 1.20) | . | 1.25 (1.08; 1.44) | . | 0.97 (0.60; 1.59) | . | . |
| 1.27 (0.89; 1.82) | 0.54 (0.34; 0.86) | 0.90 (0.73; 1.11) | 0.87 (0.69; 1.10) | O | 1.24 (0.95; 1.63) | 1.93 (1.25; 2.96) | . | . | . | 0.97 (0.79; 1.20) | . |
| 1.43 (0.94; 2.19) | 0.61 (0.37; 1.02) | 1.02 (0.80; 1.29) | 0.99 (0.87; 1.12) | 1.13 (0.91; 1.41) | O+D | . | 1.32 (1.18; 1.48) | . | . | . | . |
| 1.83 (1.16; 2.89) | 0.78 (0.46; 1.35) | 1.30 (1.00; 1.68) | 1.26 (0.91; 1.74) | 1.44 (1.08; 1.92) | 1.28 (0.92; 1.77) | P | . | . | . | . | . |
| 1.86 (1.21; 2.86) | 0.80 (0.48; 1.34) | 1.32 (1.04; 1.68) | 1.28 (1.14; 1.44) | 1.47 (1.16; 1.86) | 1.30 (1.18; 1.43) | 1.02 (0.73; 1.42) | P+D | . | . | . | . |
| 0.87 (0.43; 1.75) | 0.37 (0.17; 0.79) | 0.62 (0.34; 1.12) | 0.60 (0.34; 1.04) | 0.69 (0.38; 1.25) | 0.61 (0.34; 1.07) | 0.48 (0.25; 0.90) | 0.47 (0.26; 0.82) | R | 1.63 (1.26; 2.10) | . | . |
| 1.41 (0.74; 2.71) | 0.61 (0.30; 1.23) | 1.00 (0.58; 1.72) | 0.97 (0.60; 1.59) | 1.11 (0.65; 1.92) | 0.99 (0.59; 1.64) | 0.77 (0.43; 1.39) | 0.76 (0.46; 1.26) | 1.63 (1.26; 2.10) | R+D | . | . |
| 1.28 (0.85; 1.93) | 0.55 (0.33; 0.90) | 0.91 (0.69; 1.18) | 0.88 (0.65; 1.18) | 1.01 (0.82; 1.23) | 0.89 (0.66; 1.19) | 0.70 (0.50; 0.98) | 0.69 (0.51; 0.93) | 1.47 (0.78; 2.76) | 0.90 (0.51; 1.60) | T | 1.55 (1.22; 1.97) |
| 1.98 (1.23; 3.19) | 0.85 (0.49; 1.48) | 1.41 (0.98; 2.01) | 1.36 (0.93; 2.00) | 1.56 (1.14; 2.13) | 1.38 (0.95; 2.01) | 1.08 (0.72; 1.64) | 1.06 (0.72; 1.56) | 2.28 (1.16; 4.46) | 1.40 (0.75; 2.61) | 1.55 (1.22; 1.97) | T+D |

P-score

Do+D 0.9217

T+D 0.8419

P+D 0.8138

P 0.7798

G+D 0.5007

O+D 0.4724

R+D 0.4679

G 0.4473

T 0.3032

O 0.2780

Do 0.1066

R 0.0667

20、The outcomes after the article of “Mantovani G 1996” was deleted.

Acute complete control ：The network disconnection has made a comparison impossible.

21、The outcomes after the article of “Martoni A 1996” was deleted.

Acute nausea

| A+D | . | . | 1.16 (0.67; 2.00) | . | . | . | . | . | . | . | . |
| --- | --- | --- | --- | --- | --- | --- | --- | --- | --- | --- | --- |
| 0.89 (0.49; 1.61) | Do | 0.98 (0.85; 1.13) | . | 0.99 (0.68; 1.43) | . | . | . | . | . | . | . |
| 0.87 (0.49; 1.56) | 0.98 (0.86; 1.12) | G | 1.32 (1.06; 1.63) | 1.00 (0.93; 1.08) | . | . | . | 1.32 (0.96; 1.81) | . | 0.41 (0.23; 0.73) | . |
| 1.16 (0.67; 2.00) | 1.30 (1.03; 1.65) | 1.33 (1.10; 1.61) | G+D | . | 1.04 (0.91; 1.19) | . | 0.97 (0.84; 1.13) | . | 1.22 (0.66; 2.23) | . | . |
| 0.87 (0.49; 1.55) | 0.98 (0.84; 1.13) | 1.00 (0.93; 1.07) | 0.75 (0.61; 0.92) | O | 1.26 (0.74; 2.13) | 1.33 (0.80; 2.21) | . | 1.16 (0.72; 1.86) | . | 1.10 (0.78; 1.56) | . |
| 1.15 (0.66; 2.01) | 1.29 (1.00; 1.67) | 1.32 (1.06; 1.64) | 0.99 (0.88; 1.12) | 1.33 (1.06; 1.66) | O+D | . | 1.14 (0.93; 1.39) | . | . | . | . |
| 1.16 (0.54; 2.50) | 1.30 (0.77; 2.20) | 1.32 (0.80; 2.21) | 1.00 (0.58; 1.72) | 1.33 (0.80; 2.21) | 1.00 (0.58; 1.74) | P | . | . | . | . | . |
| 1.19 (0.68; 2.08) | 1.34 (1.03; 1.74) | 1.36 (1.09; 1.71) | 1.03 (0.90; 1.16) | 1.37 (1.08; 1.74) | 1.03 (0.90; 1.19) | 1.03 (0.59; 1.80) | P+D | . | . | . | . |
| 1.07 (0.58; 2.00) | 1.20 (0.91; 1.60) | 1.23 (0.96; 1.58) | 0.92 (0.69; 1.25) | 1.24 (0.96; 1.59) | 0.93 (0.68; 1.28) | 0.93 (0.53; 1.63) | 0.90 (0.65; 1.24) | R | 1.61 (1.16; 2.25) | . | . |
| 1.65 (0.85; 3.19) | 1.85 (1.27; 2.70) | 1.89 (1.33; 2.69) | 1.42 (0.98; 2.06) | 1.90 (1.33; 2.72) | 1.43 (0.97; 2.11) | 1.43 (0.77; 2.65) | 1.39 (0.94; 2.05) | 1.54 (1.14; 2.08) | R+D | . | . |
| 0.74 (0.38; 1.42) | 0.83 (0.60; 1.15) | 0.85 (0.62; 1.15) | 0.64 (0.44; 0.91) | 0.85 (0.63; 1.15) | 0.64 (0.44; 0.93) | 0.64 (0.36; 1.15) | 0.62 (0.42; 0.90) | 0.69 (0.47; 1.02) | 0.45 (0.28; 0.71) | T | 1.85 (1.25; 2.75) |
| 1.37 (0.64; 2.93) | 1.54 (0.92; 2.57) | 1.57 (0.95; 2.58) | 1.18 (0.69; 2.01) | 1.58 (0.96; 2.58) | 1.19 (0.69; 2.04) | 1.18 (0.58; 2.40) | 1.15 (0.67; 1.98) | 1.28 (0.73; 2.22) | 0.83 (0.45; 1.52) | 1.85 (1.25; 2.75) | T+D |

P-score

R+D 0.9462

T+D 0.7801

P+D 0.6966

G+D 0.6438

O+D 0.6267

P 0.6044

R 0.5312

A+D 0.4319

Do 0.2573

G 0.2146

O 0.2019

T 0.0653

Acute vomiting

| Do | 0.96 (0.79; 1.15) | . | 0.99 (0.85; 1.15) | . | . | . | . | . | . | . |
| --- | --- | --- | --- | --- | --- | --- | --- | --- | --- | --- |
| 0.93 (0.82; 1.06) | G | 1.57 (1.28; 1.91) | 1.09 (0.99; 1.20) | . | 1.61 (0.94; 2.77) | . | 1.32 (0.93; 1.87) | . | 0.91 (0.65; 1.27) | . |
| 1.46 (1.18; 1.80) | 1.57 (1.32; 1.87) | G+D | . | 1.06 (0.88; 1.28) | . | 1.05 (0.81; 1.36) | . | 0.84 (0.48; 1.47) | . | . |
| 1.01 (0.89; 1.14) | 1.09 (1.00; 1.18) | 0.69 (0.57; 0.83) | O | 1.28 (0.88; 1.85) | 1.98 (0.60; 6.53) | . | 1.06 (0.74; 1.52) | . | 0.78 (0.60; 1.03) | . |
| 1.41 (1.11; 1.80) | 1.52 (1.23; 1.88) | 0.97 (0.83; 1.13) | 1.40 (1.13; 1.74) | O+D | . | 1.32 (1.08; 1.60) | . | . | . | . |
| 1.57 (0.94; 2.61) | 1.69 (1.03; 2.77) | 1.08 (0.64; 1.82) | 1.56 (0.95; 2.57) | 1.11 (0.65; 1.90) | P | . | . | . | . | . |
| 1.73 (1.32; 2.27) | 1.87 (1.47; 2.38) | 1.19 (0.99; 1.43) | 1.72 (1.34; 2.20) | 1.23 (1.04; 1.45) | 1.10 (0.64; 1.91) | P+D | . | . | . | . |
| 1.10 (0.84; 1.43) | 1.18 (0.93; 1.50) | 0.75 (0.57; 1.00) | 1.09 (0.85; 1.38) | 0.78 (0.57; 1.05) | 0.70 (0.40; 1.21) | 0.63 (0.46; 0.88) | R | 1.58 (1.05; 2.38) | . | . |
| 1.54 (1.04; 2.27) | 1.66 (1.14; 2.40) | 1.05 (0.72; 1.54) | 1.52 (1.05; 2.22) | 1.09 (0.73; 1.62) | 0.98 (0.53; 1.81) | 0.89 (0.59; 1.34) | 1.40 (1.00; 1.98) | R+D | . | . |
| 0.79 (0.61; 1.03) | 0.86 (0.67; 1.09) | 0.55 (0.41; 0.73) | 0.79 (0.62; 1.00) | 0.56 (0.41; 0.77) | 0.51 (0.29; 0.87) | 0.46 (0.33; 0.64) | 0.72 (0.52; 1.01) | 0.52 (0.33; 0.80) | T | 1.99 (1.42; 2.79) |
| 1.58 (1.03; 2.42) | 1.71 (1.13; 2.58) | 1.09 (0.70; 1.70) | 1.57 (1.04; 2.37) | 1.12 (0.71; 1.77) | 1.01 (0.53; 1.91) | 0.91 (0.57; 1.47) | 1.44 (0.90; 2.32) | 1.03 (0.59; 1.79) | 1.99 (1.42; 2.79) | T+D |

P-score

P+D 0.8957

T+D 0.7626

P 0.7442

R+D 0.7423

G+D 0.6795

O+D 0.6305

R 0.3654

O 0.2813

Do 0.2611

G 0.1170

T 0.0204

Acute complete control

| G | 0.80 (0.61; 1.04) | 0.96 (0.89; 1.04) | . | . | . | . | . | 1.16 (0.92; 1.47) | . |
| --- | --- | --- | --- | --- | --- | --- | --- | --- | --- |
| 0.86 (0.74; 1.01) | G+D | . | 0.99 (0.92; 1.06) | . | 0.98 (0.91; 1.05) | . | 1.05 (0.87; 1.27) | . | . |
| 0.95 (0.88; 1.03) | 1.10 (0.95; 1.28) | O | 0.94 (0.80; 1.12) | 0.98 (0.91; 1.05) | . | . | . | 1.13 (0.89; 1.44) | . |
| 0.87 (0.75; 1.02) | 1.01 (0.95; 1.07) | 0.91 (0.79; 1.06) | O+D | . | 0.93 (0.84; 1.03) | . | . | . | . |
| 0.93 (0.84; 1.03) | 1.08 (0.91; 1.27) | 0.98 (0.91; 1.05) | 1.07 (0.91; 1.26) | P | . | . | . | . | . |
| 0.83 (0.71; 0.98) | 0.96 (0.91; 1.03) | 0.87 (0.75; 1.02) | 0.96 (0.89; 1.03) | 0.90 (0.75; 1.07) | P+D | . | . | . | . |
| 1.14 (0.84; 1.57) | 1.33 (1.01; 1.74) | 1.20 (0.88; 1.64) | 1.32 (1.00; 1.74) | 1.23 (0.89; 1.70) | 1.37 (1.04; 1.82) | R | 0.79 (0.65; 0.96) | . | . |
| 0.91 (0.71; 1.16) | 1.05 (0.87; 1.27) | 0.95 (0.75; 1.22) | 1.04 (0.85; 1.28) | 0.98 (0.76; 1.26) | 1.09 (0.89; 1.33) | 0.79 (0.65; 0.96) | R+D | . | . |
| 1.12 (0.90; 1.40) | 1.30 (1.00; 1.69) | 1.18 (0.95; 1.47) | 1.29 (0.99; 1.67) | 1.21 (0.96; 1.52) | 1.35 (1.03; 1.76) | 0.98 (0.67; 1.43) | 1.24 (0.89; 1.71) | T | 0.65 (0.53; 0.80) |
| 0.73 (0.54; 0.98) | 0.84 (0.60; 1.18) | 0.76 (0.57; 1.03) | 0.84 (0.60; 1.17) | 0.78 (0.57; 1.07) | 0.87 (0.62; 1.23) | 0.64 (0.41; 0.98) | 0.80 (0.55; 1.18) | 0.65 (0.53; 0.80) | T+D |

P-score

T+D 0.9112

P+D 0.8426

G+D 0.6901

O+D 0.6536

R+D 0.5415

P 0.5018

O 0.4013

G 0.2400

T 0.1099

R 0.1081

22、The outcomes after the article of “Marty M 1995” was deleted.

Acute nausea

| A+D | . | . | 1.16 (0.67; 2.00) | . | . | . | . | . | . | . | . |
| --- | --- | --- | --- | --- | --- | --- | --- | --- | --- | --- | --- |
| 0.89 (0.49; 1.60) | Do | 0.98 (0.85; 1.12) | . | 0.99 (0.68; 1.42) | . | . | . | . | . | . | . |
| 0.87 (0.49; 1.55) | 0.98 (0.86; 1.11) | G | 1.32 (1.07; 1.63) | 1.00 (0.93; 1.07) | . | . | . | 1.32 (0.97; 1.81) | . | 0.41 (0.23; 0.73) | . |
| 1.16 (0.67; 2.00) | 1.30 (1.04; 1.64) | 1.33 (1.10; 1.61) | G+D | . | 1.04 (0.91; 1.19) | . | 0.97 (0.84; 1.12) | . | 1.22 (0.66; 2.23) | . | . |
| 0.87 (0.49; 1.56) | 0.98 (0.85; 1.13) | 1.00 (0.94; 1.07) | 0.75 (0.62; 0.92) | O | 1.26 (0.74; 2.12) | 1.33 (0.80; 2.20) | . | 1.16 (0.72; 1.85) | . | . | . |
| 1.15 (0.66; 2.01) | 1.30 (1.01; 1.66) | 1.32 (1.07; 1.64) | 0.99 (0.89; 1.11) | 1.32 (1.06; 1.64) | O+D | . | 1.14 (0.93; 1.38) | . | . | . | . |
| 1.16 (0.54; 2.51) | 1.31 (0.77; 2.21) | 1.34 (0.80; 2.22) | 1.00 (0.58; 1.72) | 1.33 (0.80; 2.20) | 1.01 (0.58; 1.75) | P | . | . | . | . | . |
| 1.19 (0.68; 2.07) | 1.33 (1.03; 1.72) | 1.36 (1.09; 1.70) | 1.02 (0.91; 1.15) | 1.36 (1.08; 1.71) | 1.03 (0.90; 1.18) | 1.02 (0.59; 1.77) | P+D | . | . | . | . |
| 1.07 (0.58; 1.99) | 1.21 (0.91; 1.59) | 1.23 (0.96; 1.58) | 0.93 (0.69; 1.24) | 1.23 (0.96; 1.58) | 0.93 (0.68; 1.27) | 0.92 (0.53; 1.62) | 0.90 (0.66; 1.24) | R | 1.61 (1.16; 2.24) | . | . |
| 1.65 (0.86; 3.19) | 1.86 (1.28; 2.70) | 1.90 (1.34; 2.69) | 1.42 (0.99; 2.06) | 1.89 (1.33; 2.69) | 1.43 (0.98; 2.10) | 1.42 (0.77; 2.63) | 1.39 (0.95; 2.05) | 1.54 (1.14; 2.07) | R+D | . | . |
| 0.36 (0.16; 0.80) | 0.40 (0.22; 0.72) | 0.41 (0.23; 0.73) | 0.31 (0.17; 0.56) | 0.41 (0.23; 0.73) | 0.31 (0.17; 0.57) | 0.31 (0.14; 0.66) | 0.30 (0.16; 0.56) | 0.33 (0.18; 0.62) | 0.21 (0.11; 0.42) | T | 1.85 (1.26; 2.74) |
| 0.66 (0.27; 1.63) | 0.74 (0.36; 1.51) | 0.76 (0.38; 1.52) | 0.57 (0.28; 1.17) | 0.75 (0.37; 1.52) | 0.57 (0.28; 1.19) | 0.57 (0.24; 1.34) | 0.56 (0.27; 1.16) | 0.61 (0.29; 1.29) | 0.40 (0.18; 0.87) | 1.85 (1.26; 2.74) | T+D |

P-score

R+D 0.9709

P+D 0.7507

G+D 0.7039

O+D 0.6895

P 0.6692

R 0.5993

A+D 0.5012

Do 0.3333

O 0.2951

G 0.2846

T+D 0.2010

T 0.0012

Acute vomiting

| Do | 0.96 (0.79; 1.15) | . | 0.99 (0.84; 1.15) | . | . | . | . | . | . | . |
| --- | --- | --- | --- | --- | --- | --- | --- | --- | --- | --- |
| 0.93 (0.82; 1.06) | G | 1.57 (1.28; 1.91) | 1.09 (0.99; 1.19) | . | 1.61 (0.94; 2.77) | . | 1.32 (0.93; 1.87) | . | 0.91 (0.65; 1.27) | . |
| 1.46 (1.18; 1.81) | 1.57 (1.32; 1.87) | G+D | . | 1.06 (0.88; 1.28) | . | 1.05 (0.81; 1.36) | . | 0.84 (0.48; 1.47) | . | . |
| 1.01 (0.89; 1.14) | 1.08 (0.99; 1.18) | 0.69 (0.57; 0.83) | O | 1.28 (0.88; 1.85) | 1.98 (0.60; 6.53) | . | 1.06 (0.74; 1.52) | . | 0.82 (0.53; 1.27) | . |
| 1.41 (1.11; 1.80) | 1.52 (1.23; 1.88) | 0.97 (0.83; 1.13) | 1.41 (1.13; 1.75) | O+D | . | 1.32 (1.08; 1.60) | . | . | . | . |
| 1.57 (0.95; 2.62) | 1.69 (1.03; 2.77) | 1.08 (0.64; 1.82) | 1.56 (0.95; 2.58) | 1.11 (0.65; 1.90) | P | . | . | . | . | . |
| 1.74 (1.33; 2.27) | 1.87 (1.47; 2.38) | 1.19 (0.99; 1.43) | 1.73 (1.35; 2.21) | 1.23 (1.04; 1.45) | 1.10 (0.64; 1.91) | P+D | . | . | . | . |
| 1.10 (0.84; 1.43) | 1.18 (0.93; 1.50) | 0.75 (0.57; 1.00) | 1.09 (0.86; 1.39) | 0.78 (0.57; 1.05) | 0.70 (0.40; 1.20) | 0.63 (0.45; 0.88) | R | 1.58 (1.05; 2.38) | . | . |
| 1.54 (1.04; 2.27) | 1.65 (1.14; 2.40) | 1.05 (0.72; 1.54) | 1.53 (1.05; 2.22) | 1.09 (0.73; 1.62) | 0.98 (0.53; 1.81) | 0.89 (0.59; 1.34) | 1.40 (1.00; 1.98) | R+D | . | . |
| 0.82 (0.58; 1.14) | 0.88 (0.64; 1.20) | 0.56 (0.39; 0.80) | 0.81 (0.59; 1.12) | 0.58 (0.40; 0.84) | 0.52 (0.29; 0.93) | 0.47 (0.32; 0.70) | 0.75 (0.50; 1.11) | 0.53 (0.33; 0.86) | T | 1.99 (1.42; 2.79) |
| 1.63 (1.01; 2.62) | 1.75 (1.10; 2.78) | 1.12 (0.68; 1.83) | 1.62 (1.02; 2.58) | 1.15 (0.69; 1.91) | 1.04 (0.53; 2.03) | 0.94 (0.56; 1.58) | 1.49 (0.89; 2.50) | 1.06 (0.59; 1.91) | 1.99 (1.42; 2.79) | T+D |

P-score

P+D 0.8904

T+D 0.7782

P 0.7409

R+D 0.7379

G+D 0.6772

O+D 0.6276

R 0.3616

O 0.2716

Do 0.2536

G 0.1082

T 0.0528

Delayed nausea ：The network disconnection has made a comparison impossible.

Delayed vomiting

| Do | 2.33 (1.53; 3.56) | . | . | 1.27 (0.79; 2.04) | . | . | . | . | . | . | . |
| --- | --- | --- | --- | --- | --- | --- | --- | --- | --- | --- | --- |
| 2.33 (1.53; 3.56) | Do+D | . | . | . | . | . | . | . | . | . | . |
| 1.39 (0.80; 2.41) | 0.59 (0.30; 1.19) | G | 0.93 (0.61; 1.41) | 1.09 (0.76; 1.56) | . | 1.12 (0.72; 1.73) | . | . | . | 1.14 (0.66; 1.98) | . |
| 1.37 (0.77; 2.47) | 0.59 (0.29; 1.21) | 0.99 (0.71; 1.38) | G+D | . | 0.99 (0.69; 1.43) | . | 1.61 (1.20; 2.17) | . | 0.97 (0.54; 1.74) | . | . |
| 1.27 (0.79; 2.04) | 0.54 (0.29; 1.03) | 0.92 (0.69; 1.21) | 0.92 (0.66; 1.30) | O | 1.28 (0.89; 1.86) | 1.87 (1.16; 3.03) | . | . | . | 1.00 (0.56; 1.78) | . |
| 1.55 (0.88; 2.74) | 0.66 (0.33; 1.35) | 1.12 (0.79; 1.58) | 1.13 (0.89; 1.43) | 1.22 (0.89; 1.67) | O+D | . | 1.33 (1.10; 1.61) | . | . | . | . |
| 1.88 (1.04; 3.41) | 0.81 (0.39; 1.68) | 1.36 (0.96; 1.92) | 1.37 (0.88; 2.14) | 1.48 (1.04; 2.12) | 1.21 (0.78; 1.89) | P | . | . | . | . | . |
| 2.10 (1.17; 3.78) | 0.90 (0.44; 1.86) | 1.52 (1.06; 2.18) | 1.53 (1.21; 1.93) | 1.66 (1.18; 2.33) | 1.36 (1.14; 1.62) | 1.12 (0.71; 1.77) | P+D | . | . | . | . |
| 0.82 (0.33; 2.06) | 0.35 (0.13; 0.97) | 0.59 (0.27; 1.30) | 0.60 (0.29; 1.21) | 0.65 (0.30; 1.42) | 0.53 (0.25; 1.12) | 0.44 (0.19; 1.01) | 0.39 (0.19; 0.82) | R | 1.63 (1.09; 2.44) | . | . |
| 1.34 (0.59; 3.05) | 0.57 (0.23; 1.45) | 0.96 (0.49; 1.89) | 0.97 (0.54; 1.74) | 1.05 (0.54; 2.07) | 0.86 (0.46; 1.62) | 0.71 (0.34; 1.48) | 0.64 (0.34; 1.19) | 1.63 (1.09; 2.44) | R+D | . | . |
| 1.43 (0.71; 2.89) | 0.62 (0.27; 1.39) | 1.04 (0.62; 1.71) | 1.04 (0.59; 1.86) | 1.13 (0.68; 1.89) | 0.93 (0.52; 1.64) | 0.76 (0.42; 1.37) | 0.68 (0.38; 1.23) | 1.75 (0.70; 4.35) | 1.07 (0.47; 2.44) | T | 1.59 (1.10; 2.31) |
| 2.28 (1.03; 5.04) | 0.98 (0.40; 2.40) | 1.65 (0.88; 3.08) | 1.66 (0.83; 3.30) | 1.80 (0.95; 3.39) | 1.47 (0.74; 2.91) | 1.21 (0.61; 2.43) | 1.08 (0.54; 2.17) | 2.77 (1.03; 7.44) | 1.71 (0.69; 4.20) | 1.59 (1.10; 2.31) | T+D |

P-score

T+D 0.8456

Do+D 0.8433

P+D 0.8433

P 0.7405

O+D 0.5551

T 0.4449

R+D 0.4077

G 0.4062

G+D 0.3916

O 0.2940

Do 0.1458

R 0.0818

23、The outcomes after the article of “Mattiuzzi GN 2010” was deleted.

Acute nausea

| A+D | . | . | 1.16 (0.67; 2.00) | . | . | . | . | . | . | . | . |
| --- | --- | --- | --- | --- | --- | --- | --- | --- | --- | --- | --- |
| 0.89 (0.49; 1.61) | Do | 0.98 (0.85; 1.13) | . | 0.99 (0.68; 1.43) | . | . | . | . | . | . | . |
| 0.87 (0.49; 1.56) | 0.98 (0.86; 1.12) | G | 1.32 (1.06; 1.63) | 1.00 (0.93; 1.08) | . | . | . | 1.32 (0.96; 1.82) | . | 0.41 (0.23; 0.73) | . |
| 1.16 (0.67; 2.00) | 1.30 (1.03; 1.65) | 1.33 (1.09; 1.62) | G+D | . | 1.04 (0.91; 1.19) | . | 0.97 (0.83; 1.14) | . | 1.22 (0.66; 2.23) | . | . |
| 0.87 (0.48; 1.55) | 0.97 (0.84; 1.13) | 0.99 (0.92; 1.07) | 0.75 (0.61; 0.92) | O | 1.26 (0.74; 2.13) | 0.98 (0.37; 2.56) | . | 1.16 (0.72; 1.86) | . | 1.10 (0.78; 1.57) | . |
| 1.15 (0.66; 2.01) | 1.29 (1.00; 1.67) | 1.32 (1.06; 1.64) | 0.99 (0.88; 1.12) | 1.33 (1.06; 1.67) | O+D | . | 1.14 (0.93; 1.39) | . | . | . | . |
| 0.85 (0.27; 2.61) | 0.95 (0.36; 2.52) | 0.97 (0.37; 2.55) | 0.73 (0.27; 1.96) | 0.98 (0.37; 2.56) | 0.74 (0.27; 1.98) | P | . | . | . | . | . |
| 1.19 (0.68; 2.09) | 1.34 (1.03; 1.75) | 1.37 (1.08; 1.72) | 1.03 (0.90; 1.17) | 1.38 (1.08; 1.75) | 1.04 (0.90; 1.20) | 1.41 (0.52; 3.80) | P+D | . | . | . | . |
| 1.07 (0.57; 2.00) | 1.20 (0.91; 1.60) | 1.23 (0.96; 1.58) | 0.92 (0.68; 1.25) | 1.24 (0.96; 1.60) | 0.93 (0.68; 1.28) | 1.26 (0.47; 3.43) | 0.90 (0.65; 1.25) | R | 1.61 (1.15; 2.25) | . | . |
| 1.65 (0.85; 3.19) | 1.85 (1.27; 2.71) | 1.89 (1.32; 2.70) | 1.42 (0.98; 2.06) | 1.90 (1.33; 2.73) | 1.43 (0.97; 2.11) | 1.95 (0.70; 5.44) | 1.38 (0.93; 2.05) | 1.54 (1.14; 2.08) | R+D | . | . |
| 0.74 (0.38; 1.42) | 0.83 (0.59; 1.15) | 0.84 (0.62; 1.14) | 0.63 (0.44; 0.91) | 0.85 (0.63; 1.15) | 0.64 (0.44; 0.93) | 0.87 (0.32; 2.38) | 0.62 (0.42; 0.90) | 0.69 (0.46; 1.02) | 0.45 (0.28; 0.71) | T | 1.85 (1.25; 2.75) |
| 1.36 (0.64; 2.93) | 1.53 (0.91; 2.57) | 1.56 (0.95; 2.58) | 1.18 (0.69; 2.01) | 1.57 (0.96; 2.59) | 1.18 (0.69; 2.04) | 1.61 (0.54; 4.76) | 1.14 (0.66; 1.98) | 1.27 (0.73; 2.22) | 0.83 (0.45; 1.53) | 1.85 (1.25; 2.75) | T+D |

P-score

R+D 0.9482

T+D 0.7886

P+D 0.7178

G+D 0.6649

O+D 0.6470

R 0.5561

A+D 0.4561

P 0.3473

Do 0.2926

G 0.2526

O 0.2345

T 0.0942

Acute vomiting

| Do | 0.96 (0.79; 1.16) | . | 0.99 (0.84; 1.16) | . | . | . | . | . | . | . |
| --- | --- | --- | --- | --- | --- | --- | --- | --- | --- | --- |
| 0.93 (0.81; 1.06) | G | 1.57 (1.28; 1.91) | 1.09 (0.99; 1.20) | . | 1.61 (0.94; 2.78) | . | 1.32 (0.93; 1.87) | . | 0.91 (0.65; 1.28) | . |
| 1.46 (1.18; 1.81) | 1.57 (1.32; 1.87) | G+D | . | 1.06 (0.88; 1.28) | . | 1.05 (0.80; 1.37) | . | 0.84 (0.48; 1.47) | . | . |
| 1.01 (0.89; 1.14) | 1.08 (0.99; 1.18) | 0.69 (0.57; 0.83) | O | 1.28 (0.88; 1.86) | 1.96 (0.18; 20.90) | . | 1.06 (0.73; 1.53) | . | 0.78 (0.59; 1.03) | . |
| 1.41 (1.11; 1.80) | 1.52 (1.23; 1.88) | 0.97 (0.83; 1.13) | 1.40 (1.13; 1.74) | O+D | . | 1.32 (1.08; 1.60) | . | . | . | . |
| 1.52 (0.88; 2.62) | 1.63 (0.96; 2.78) | 1.04 (0.60; 1.82) | 1.51 (0.88; 2.58) | 1.07 (0.61; 1.90) | P | . | . | . | . | . |
| 1.74 (1.32; 2.28) | 1.87 (1.46; 2.38) | 1.19 (0.99; 1.43) | 1.72 (1.34; 2.21) | 1.23 (1.04; 1.45) | 1.14 (0.64; 2.05) | P+D | . | . | . | . |
| 1.10 (0.84; 1.43) | 1.18 (0.93; 1.50) | 0.75 (0.57; 1.00) | 1.09 (0.85; 1.39) | 0.78 (0.57; 1.05) | 0.72 (0.40; 1.29) | 0.63 (0.45; 0.88) | R | 1.58 (1.05; 2.38) | . | . |
| 1.54 (1.04; 2.27) | 1.65 (1.14; 2.40) | 1.05 (0.72; 1.54) | 1.52 (1.05; 2.22) | 1.09 (0.73; 1.62) | 1.01 (0.53; 1.94) | 0.89 (0.58; 1.34) | 1.40 (0.99; 1.98) | R+D | . | . |
| 0.79 (0.61; 1.03) | 0.85 (0.67; 1.09) | 0.54 (0.41; 0.73) | 0.79 (0.62; 1.00) | 0.56 (0.41; 0.77) | 0.52 (0.29; 0.94) | 0.46 (0.33; 0.64) | 0.72 (0.52; 1.01) | 0.52 (0.33; 0.80) | T | 1.99 (1.42; 2.80) |
| 1.58 (1.03; 2.43) | 1.70 (1.12; 2.58) | 1.08 (0.69; 1.70) | 1.57 (1.04; 2.38) | 1.12 (0.70; 1.78) | 1.04 (0.53; 2.04) | 0.91 (0.56; 1.47) | 1.44 (0.90; 2.33) | 1.03 (0.59; 1.80) | 1.99 (1.42; 2.80) | T+D |

P-score

P+D 0.8994

T+D 0.7658

R+D 0.7457

P 0.7095

G+D 0.6861

O+D 0.6361

R 0.3690

O 0.2836

Do 0.2632

G 0.1203

T 0.0214

Acute complete control

| G | 0.80 (0.61; 1.04) | 0.96 (0.89; 1.03) | . | . | . | . | . | 1.16 (0.92; 1.47) | . |
| --- | --- | --- | --- | --- | --- | --- | --- | --- | --- |
| 0.86 (0.74; 1.01) | G+D | . | 0.99 (0.92; 1.06) | . | 0.98 (0.91; 1.05) | . | 1.05 (0.87; 1.27) | . | . |
| 0.95 (0.88; 1.02) | 1.10 (0.95; 1.28) | O | 0.94 (0.80; 1.12) | 1.00 (0.88; 1.14) | . | . | . | 1.13 (0.89; 1.44) | . |
| 0.87 (0.74; 1.01) | 1.01 (0.95; 1.07) | 0.91 (0.79; 1.06) | O+D | . | 0.93 (0.84; 1.03) | . | . | . | . |
| 0.95 (0.82; 1.11) | 1.11 (0.91; 1.35) | 1.00 (0.88; 1.14) | 1.10 (0.90; 1.33) | P | . | . | . | . | . |
| 0.83 (0.71; 0.98) | 0.96 (0.91; 1.03) | 0.87 (0.75; 1.02) | 0.96 (0.89; 1.03) | 0.87 (0.71; 1.07) | P+D | . | . | . | . |
| 1.14 (0.83; 1.57) | 1.33 (1.01; 1.74) | 1.20 (0.88; 1.64) | 1.32 (1.00; 1.74) | 1.20 (0.86; 1.68) | 1.37 (1.04; 1.82) | R | 0.79 (0.65; 0.96) | . | . |
| 0.91 (0.71; 1.16) | 1.05 (0.87; 1.27) | 0.95 (0.75; 1.22) | 1.04 (0.85; 1.28) | 0.95 (0.72; 1.26) | 1.09 (0.89; 1.33) | 0.79 (0.65; 0.96) | R+D | . | . |
| 1.12 (0.90; 1.39) | 1.30 (1.00; 1.69) | 1.18 (0.95; 1.47) | 1.29 (0.99; 1.67) | 1.18 (0.91; 1.52) | 1.35 (1.03; 1.76) | 0.98 (0.67; 1.43) | 1.24 (0.89; 1.71) | T | 0.65 (0.53; 0.80) |
| 0.73 (0.54; 0.98) | 0.84 (0.60; 1.18) | 0.77 (0.57; 1.03) | 0.84 (0.60; 1.17) | 0.76 (0.55; 1.06) | 0.87 (0.62; 1.23) | 0.64 (0.41; 0.98) | 0.80 (0.55; 1.18) | 0.65 (0.53; 0.80) | T+D |

P-score

T+D 0.9119

P+D 0.8441

G+D 0.6937

O+D 0.6581

R+D 0.5491

O 0.4314

P 0.4249

G 0.2582

T 0.1155

R 0.1132

Delayed nausea

| Do | 1.99 (1.25; 3.15) | . | . | 0.91 (0.62; 1.35) | . | . | . | . | . | . | . |
| --- | --- | --- | --- | --- | --- | --- | --- | --- | --- | --- | --- |
| 1.99 (1.25; 3.15) | Do+D | . | . | . | . | . | . | . | . | . | . |
| 0.99 (0.56; 1.74) | 0.50 (0.24; 1.03) | G | 0.97 (0.65; 1.44) | 1.17 (0.67; 2.06) | . | . | . | . | . | . | . |
| 1.08 (0.63; 1.84) | 0.54 (0.27; 1.10) | 1.09 (0.77; 1.54) | G+D | . | 1.07 (0.74; 1.55) | . | 1.42 (1.07; 1.88) | . | 1.02 (0.62; 1.65) | . | . |
| 0.91 (0.62; 1.35) | 0.46 (0.25; 0.84) | 0.92 (0.62; 1.39) | 0.85 (0.59; 1.22) | O | 1.44 (1.02; 2.02) | 1.54 (0.94; 2.52) | . | . | . | 1.21 (0.78; 1.87) | . |
| 1.20 (0.73; 1.98) | 0.60 (0.31; 1.19) | 1.22 (0.83; 1.79) | 1.11 (0.86; 1.44) | 1.32 (0.97; 1.79) | O+D | . | 1.37 (1.04; 1.81) | . | . | . | . |
| 1.40 (0.75; 2.64) | 0.71 (0.32; 1.54) | 1.42 (0.75; 2.69) | 1.30 (0.70; 2.41) | 1.54 (0.94; 2.52) | 1.17 (0.65; 2.09) | P | . | . | . | . | . |
| 1.59 (0.93; 2.72) | 0.80 (0.39; 1.62) | 1.61 (1.08; 2.39) | 1.47 (1.17; 1.86) | 1.74 (1.20; 2.53) | 1.32 (1.05; 1.67) | 1.13 (0.61; 2.11) | P+D | . | . | . | . |
| 0.71 (0.31; 1.64) | 0.36 (0.14; 0.93) | 0.72 (0.35; 1.49) | 0.66 (0.35; 1.25) | 0.78 (0.38; 1.63) | 0.59 (0.30; 1.18) | 0.51 (0.21; 1.23) | 0.45 (0.23; 0.88) | R | 1.53 (1.02; 2.30) | . | . |
| 1.09 (0.53; 2.26) | 0.55 (0.23; 1.30) | 1.11 (0.61; 2.01) | 1.02 (0.62; 1.65) | 1.20 (0.65; 2.20) | 0.91 (0.53; 1.58) | 0.78 (0.36; 1.71) | 0.69 (0.40; 1.18) | 1.53 (1.02; 2.30) | R+D | . | . |
| 1.10 (0.61; 1.98) | 0.55 (0.26; 1.17) | 1.12 (0.61; 2.03) | 1.02 (0.58; 1.81) | 1.21 (0.78; 1.87) | 0.92 (0.54; 1.57) | 0.79 (0.41; 1.52) | 0.69 (0.39; 1.23) | 1.54 (0.66; 3.62) | 1.01 (0.48; 2.13) | T | 1.49 (0.97; 2.27) |
| 1.64 (0.79; 3.38) | 0.82 (0.35; 1.94) | 1.66 (0.80; 3.45) | 1.52 (0.75; 3.09) | 1.79 (0.98; 3.30) | 1.36 (0.69; 2.70) | 1.17 (0.53; 2.56) | 1.03 (0.50; 2.10) | 2.29 (0.88; 5.94) | 1.50 (0.63; 3.54) | 1.49 (0.97; 2.27) | T+D |

P-score

Do+D 0.8994

P+D 0.8289

T+D 0.7939

P 0.6796

O+D 0.5565

R+D 0.4490

T 0.4326

G+D 0.4119

Do 0.3334

G 0.3103

O 0.2006

R 0.1038

Delayed vomiting

| Do | 2.33 (1.52; 3.59) | . | . | 1.27 (0.78; 2.06) | . | . | . | . | . | . | . |
| --- | --- | --- | --- | --- | --- | --- | --- | --- | --- | --- | --- |
| 2.33 (1.52; 3.59) | Do+D | . | . | . | . | . | . | . | . | . | . |
| 1.38 (0.79; 2.41) | 0.59 (0.29; 1.20) | G | 0.93 (0.61; 1.43) | 1.09 (0.76; 1.57) | . | 1.12 (0.71; 1.75) | . | . | . | 1.14 (0.66; 1.99) | . |
| 1.37 (0.76; 2.48) | 0.59 (0.28; 1.22) | 0.99 (0.71; 1.39) | G+D | . | 0.99 (0.68; 1.44) | . | 1.63 (1.20; 2.20) | . | 0.97 (0.54; 1.75) | . | . |
| 1.27 (0.78; 2.06) | 0.54 (0.28; 1.04) | 0.92 (0.70; 1.22) | 0.93 (0.66; 1.31) | O | 1.29 (0.89; 1.87) | 2.36 (1.27; 4.39) | . | . | . | 0.98 (0.70; 1.35) | . |
| 1.55 (0.87; 2.76) | 0.67 (0.32; 1.37) | 1.13 (0.79; 1.60) | 1.13 (0.89; 1.44) | 1.22 (0.89; 1.68) | O+D | . | 1.33 (1.09; 1.61) | . | . | . | . |
| 1.93 (1.03; 3.63) | 0.83 (0.39; 1.78) | 1.40 (0.96; 2.04) | 1.41 (0.87; 2.28) | 1.52 (1.01; 2.29) | 1.25 (0.77; 2.01) | P | . | . | . | . | . |
| 2.11 (1.16; 3.82) | 0.90 (0.43; 1.88) | 1.53 (1.06; 2.21) | 1.54 (1.21; 1.95) | 1.66 (1.17; 2.35) | 1.36 (1.14; 1.62) | 1.09 (0.67; 1.79) | P+D | . | . | . | . |
| 0.82 (0.32; 2.08) | 0.35 (0.13; 0.98) | 0.59 (0.27; 1.31) | 0.60 (0.29; 1.23) | 0.64 (0.29; 1.43) | 0.53 (0.25; 1.13) | 0.42 (0.18; 1.00) | 0.39 (0.18; 0.83) | R | 1.63 (1.08; 2.46) | . | . |
| 1.33 (0.58; 3.07) | 0.57 (0.22; 1.46) | 0.97 (0.49; 1.91) | 0.97 (0.54; 1.75) | 1.05 (0.53; 2.08) | 0.86 (0.45; 1.62) | 0.69 (0.32; 1.47) | 0.63 (0.34; 1.19) | 1.63 (1.08; 2.46) | R+D | . | . |
| 1.30 (0.73; 2.32) | 0.56 (0.27; 1.15) | 0.94 (0.65; 1.38) | 0.95 (0.61; 1.49) | 1.02 (0.75; 1.41) | 0.84 (0.54; 1.29) | 0.67 (0.41; 1.10) | 0.62 (0.39; 0.97) | 1.59 (0.68; 3.70) | 0.98 (0.47; 2.05) | T | 1.59 (1.09; 2.33) |
| 2.07 (1.04; 4.13) | 0.89 (0.39; 2.00) | 1.50 (0.88; 2.57) | 1.51 (0.84; 2.72) | 1.63 (1.00; 2.67) | 1.33 (0.75; 2.37) | 1.07 (0.58; 2.00) | 0.98 (0.54; 1.78) | 2.53 (1.00; 6.39) | 1.55 (0.68; 3.57) | 1.59 (1.09; 2.33) | T+D |

P-score

P+D 0.8566

Do+D 0.8537

T+D 0.8099

P 0.7727

O+D 0.5746

G 0.4179

R+D 0.4162

G+D 0.4036

T 0.3506

O 0.3077

Do 0.1511

R 0.0853

24、The outcomes after the article of “Nakamura K 2012” was deleted.

Acute nausea

| Do | 0.98 (0.85; 1.12) | . | 0.99 (0.68; 1.42) | . | . | . | . | . | . | . |
| --- | --- | --- | --- | --- | --- | --- | --- | --- | --- | --- |
| 0.98 (0.86; 1.11) | G | 1.32 (1.07; 1.63) | 1.00 (0.93; 1.07) | . | . | . | 1.32 (0.97; 1.81) | . | 0.41 (0.23; 0.73) | . |
| 1.30 (1.04; 1.64) | 1.33 (1.10; 1.61) | G+D | . | 1.04 (0.91; 1.19) | . | 0.97 (0.84; 1.12) | . | 1.22 (0.66; 2.23) | . | . |
| 0.97 (0.85; 1.12) | 0.99 (0.93; 1.06) | 0.75 (0.61; 0.91) | O | 1.26 (0.74; 2.12) | 1.33 (0.80; 2.20) | . | 1.16 (0.72; 1.85) | . | 1.10 (0.78; 1.56) | . |
| 1.29 (1.01; 1.66) | 1.32 (1.07; 1.63) | 0.99 (0.89; 1.11) | 1.33 (1.07; 1.66) | O+D | . | 1.14 (0.93; 1.38) | . | . | . | . |
| 1.30 (0.77; 2.19) | 1.32 (0.80; 2.20) | 0.99 (0.58; 1.71) | 1.33 (0.80; 2.20) | 1.00 (0.58; 1.73) | P | . | . | . | . | . |
| 1.33 (1.03; 1.72) | 1.36 (1.09; 1.70) | 1.02 (0.91; 1.15) | 1.37 (1.09; 1.72) | 1.03 (0.90; 1.18) | 1.03 (0.59; 1.79) | P+D | . | . | . | . |
| 1.20 (0.91; 1.59) | 1.23 (0.96; 1.57) | 0.92 (0.69; 1.24) | 1.24 (0.96; 1.59) | 0.93 (0.68; 1.27) | 0.93 (0.53; 1.63) | 0.90 (0.66; 1.24) | R | 1.61 (1.16; 2.24) | . | . |
| 1.85 (1.28; 2.69) | 1.89 (1.33; 2.69) | 1.42 (0.99; 2.06) | 1.90 (1.34; 2.71) | 1.43 (0.98; 2.10) | 1.43 (0.77; 2.65) | 1.39 (0.95; 2.05) | 1.54 (1.14; 2.07) | R+D | . | . |
| 0.83 (0.60; 1.15) | 0.85 (0.63; 1.14) | 0.64 (0.45; 0.91) | 0.85 (0.63; 1.15) | 0.64 (0.44; 0.93) | 0.64 (0.36; 1.15) | 0.62 (0.43; 0.90) | 0.69 (0.47; 1.01) | 0.45 (0.28; 0.71) | T | 1.85 (1.26; 2.74) |
| 1.54 (0.93; 2.56) | 1.57 (0.96; 2.57) | 1.18 (0.70; 2.00) | 1.58 (0.97; 2.58) | 1.19 (0.70; 2.03) | 1.19 (0.59; 2.40) | 1.15 (0.67; 1.98) | 1.28 (0.74; 2.21) | 0.83 (0.45; 1.52) | 1.85 (1.26; 2.74) | T+D |

P-score

R+D 0.9488

T+D 0.7816

P+D 0.6898

G+D 0.6383

O+D 0.6223

P 0.5991

R 0.5264

Do 0.2488

G 0.2059

O 0.1855

T 0.0536

25、The outcomes after the article of “Navari R 1995” was deleted.

Acute nausea

| A+D | . | . | 1.16 (0.67; 2.01) | . | . | . | . | . | . | . | . |
| --- | --- | --- | --- | --- | --- | --- | --- | --- | --- | --- | --- |
| 0.89 (0.49; 1.62) | Do | 0.98 (0.84; 1.14) | . | 0.99 (0.68; 1.44) | . | . | . | . | . | . | . |
| 0.87 (0.49; 1.56) | 0.98 (0.85; 1.13) | G | 1.32 (1.06; 1.64) | 1.02 (0.90; 1.15) | . | . | . | 1.32 (0.95; 1.83) | . | 0.41 (0.23; 0.73) | . |
| 1.16 (0.67; 2.01) | 1.30 (1.02; 1.66) | 1.33 (1.09; 1.62) | G+D | . | 1.04 (0.90; 1.20) | . | 0.97 (0.82; 1.15) | . | 1.22 (0.66; 2.24) | . | . |
| 0.87 (0.48; 1.57) | 0.98 (0.83; 1.16) | 1.00 (0.89; 1.11) | 0.75 (0.60; 0.93) | O | 1.26 (0.74; 2.14) | 1.33 (0.80; 2.21) | . | 1.16 (0.71; 1.87) | . | 1.10 (0.77; 1.57) | . |
| 1.15 (0.66; 2.02) | 1.29 (0.99; 1.68) | 1.32 (1.05; 1.65) | 0.99 (0.88; 1.12) | 1.32 (1.04; 1.68) | O+D | . | 1.14 (0.93; 1.39) | . | . | . | . |
| 1.16 (0.53; 2.52) | 1.30 (0.76; 2.22) | 1.33 (0.79; 2.23) | 1.00 (0.57; 1.73) | 1.33 (0.80; 2.21) | 1.00 (0.57; 1.76) | P | . | . | . | . | . |
| 1.20 (0.68; 2.11) | 1.34 (1.02; 1.77) | 1.37 (1.08; 1.74) | 1.03 (0.90; 1.18) | 1.38 (1.07; 1.77) | 1.04 (0.90; 1.21) | 1.03 (0.59; 1.82) | P+D | . | . | . | . |
| 1.07 (0.57; 2.01) | 1.20 (0.90; 1.61) | 1.23 (0.95; 1.58) | 0.92 (0.68; 1.25) | 1.23 (0.95; 1.61) | 0.93 (0.67; 1.29) | 0.93 (0.52; 1.64) | 0.90 (0.64; 1.25) | R | 1.61 (1.15; 2.26) | . | . |
| 1.65 (0.85; 3.21) | 1.85 (1.26; 2.72) | 1.89 (1.32; 2.71) | 1.42 (0.97; 2.07) | 1.89 (1.31; 2.74) | 1.43 (0.97; 2.12) | 1.42 (0.76; 2.66) | 1.38 (0.92; 2.05) | 1.54 (1.13; 2.09) | R+D | . | . |
| 0.73 (0.38; 1.42) | 0.83 (0.59; 1.16) | 0.84 (0.62; 1.15) | 0.63 (0.44; 0.91) | 0.85 (0.62; 1.15) | 0.64 (0.44; 0.94) | 0.64 (0.35; 1.15) | 0.61 (0.42; 0.91) | 0.69 (0.46; 1.02) | 0.45 (0.28; 0.72) | T | 1.85 (1.24; 2.76) |
| 1.36 (0.63; 2.95) | 1.53 (0.91; 2.59) | 1.56 (0.94; 2.60) | 1.17 (0.68; 2.02) | 1.57 (0.95; 2.59) | 1.18 (0.68; 2.06) | 1.18 (0.58; 2.40) | 1.14 (0.65; 1.99) | 1.27 (0.72; 2.24) | 0.83 (0.45; 1.54) | 1.85 (1.24; 2.76) | T+D |

P-score

R+D 0.9440

T+D 0.7737

P+D 0.7019

G+D 0.6436

O+D 0.6238

P 0.6049

R 0.5292

A+D 0.4329

Do 0.2568

G 0.2137

O 0.2096

T 0.0658

Acute vomiting

| Do | 0.96 (0.78; 1.17) | . | 0.99 (0.84; 1.17) | . | . | . | . | . | . | . |
| --- | --- | --- | --- | --- | --- | --- | --- | --- | --- | --- |
| 0.93 (0.80; 1.07) | G | 1.56 (1.27; 1.93) | 1.09 (0.96; 1.25) | . | 1.61 (0.93; 2.80) | . | 1.32 (0.92; 1.89) | . | 0.90 (0.64; 1.28) | . |
| 1.46 (1.17; 1.83) | 1.57 (1.31; 1.89) | G+D | . | 1.06 (0.87; 1.29) | . | 1.05 (0.79; 1.39) | . | 0.84 (0.48; 1.47) | . | . |
| 1.01 (0.88; 1.16) | 1.09 (0.98; 1.21) | 0.69 (0.57; 0.84) | O | 1.29 (0.88; 1.88) | 1.98 (0.60; 6.54) | . | 1.06 (0.73; 1.54) | . | 0.78 (0.59; 1.04) | . |
| 1.41 (1.10; 1.82) | 1.52 (1.22; 1.90) | 0.97 (0.82; 1.14) | 1.40 (1.11; 1.76) | O+D | . | 1.31 (1.08; 1.61) | . | . | . | . |
| 1.58 (0.94; 2.65) | 1.70 (1.03; 2.80) | 1.08 (0.63; 1.84) | 1.56 (0.94; 2.59) | 1.11 (0.65; 1.92) | P | . | . | . | . | . |
| 1.74 (1.31; 2.31) | 1.87 (1.45; 2.41) | 1.19 (0.98; 1.45) | 1.72 (1.33; 2.24) | 1.23 (1.04; 1.46) | 1.10 (0.63; 1.93) | P+D | . | . | . | . |
| 1.10 (0.83; 1.45) | 1.18 (0.92; 1.51) | 0.75 (0.56; 1.00) | 1.08 (0.84; 1.40) | 0.77 (0.56; 1.06) | 0.70 (0.40; 1.21) | 0.63 (0.45; 0.89) | R | 1.58 (1.04; 2.41) | . | . |
| 1.53 (1.03; 2.29) | 1.65 (1.13; 2.41) | 1.05 (0.71; 1.54) | 1.52 (1.03; 2.23) | 1.08 (0.72; 1.63) | 0.97 (0.52; 1.82) | 0.88 (0.58; 1.35) | 1.40 (0.98; 1.99) | R+D | . | . |
| 0.79 (0.60; 1.04) | 0.85 (0.67; 1.10) | 0.54 (0.40; 0.74) | 0.78 (0.61; 1.00) | 0.56 (0.41; 0.78) | 0.50 (0.29; 0.88) | 0.46 (0.32; 0.65) | 0.72 (0.51; 1.02) | 0.52 (0.33; 0.81) | T | 2.00 (1.41; 2.82) |
| 1.58 (1.02; 2.46) | 1.71 (1.11; 2.61) | 1.08 (0.68; 1.72) | 1.57 (1.03; 2.40) | 1.12 (0.70; 1.80) | 1.01 (0.52; 1.94) | 0.91 (0.56; 1.49) | 1.45 (0.89; 2.36) | 1.03 (0.59; 1.82) | 2.00 (1.41; 2.82) | T+D |

P-score

P+D 0.8951

T+D 0.7603

P 0.7440

R+D 0.7373

G+D 0.6822

O+D 0.6313

R 0.3633

O 0.2816

Do 0.2584

G 0.1239

T 0.0226

Acute complete control

| G | 0.80 (0.61; 1.04) | 0.93 (0.85; 1.02) | . | . | . | . | . | 1.16 (0.92; 1.47) | . |
| --- | --- | --- | --- | --- | --- | --- | --- | --- | --- |
| 0.85 (0.72; 0.99) | G+D | . | 0.99 (0.92; 1.06) | . | 0.98 (0.91; 1.05) | . | 1.05 (0.87; 1.27) | . | . |
| 0.92 (0.84; 1.01) | 1.09 (0.94; 1.27) | O | 0.94 (0.80; 1.12) | 0.98 (0.91; 1.05) | . | . | . | 1.13 (0.89; 1.44) | . |
| 0.85 (0.73; 1.00) | 1.01 (0.95; 1.07) | 0.92 (0.80; 1.07) | O+D | . | 0.93 (0.84; 1.03) | . | . | . | . |
| 0.90 (0.80; 1.01) | 1.07 (0.90; 1.26) | 0.98 (0.91; 1.05) | 1.06 (0.90; 1.25) | P | . | . | . | . | . |
| 0.81 (0.69; 0.96) | 0.96 (0.91; 1.03) | 0.88 (0.75; 1.03) | 0.96 (0.89; 1.03) | 0.90 (0.76; 1.08) | P+D | . | . | . | . |
| 1.12 (0.82; 1.54) | 1.33 (1.01; 1.74) | 1.21 (0.89; 1.66) | 1.32 (1.00; 1.74) | 1.24 (0.90; 1.71) | 1.38 (1.04; 1.82) | R | 0.79 (0.65; 0.96) | . | . |
| 0.89 (0.69; 1.14) | 1.05 (0.87; 1.27) | 0.96 (0.75; 1.23) | 1.04 (0.85; 1.28) | 0.99 (0.76; 1.27) | 1.09 (0.89; 1.33) | 0.79 (0.65; 0.96) | R+D | . | . |
| 1.11 (0.89; 1.38) | 1.31 (1.01; 1.70) | 1.20 (0.96; 1.49) | 1.30 (1.00; 1.69) | 1.23 (0.97; 1.55) | 1.36 (1.04; 1.77) | 0.99 (0.68; 1.44) | 1.24 (0.90; 1.72) | T | 0.65 (0.53; 0.80) |
| 0.72 (0.53; 0.97) | 0.85 (0.61; 1.19) | 0.78 (0.57; 1.05) | 0.84 (0.60; 1.18) | 0.80 (0.58; 1.09) | 0.88 (0.63; 1.23) | 0.64 (0.42; 0.99) | 0.81 (0.55; 1.19) | 0.65 (0.53; 0.80) | T+D |

P-score

T+D 0.9045

P+D 0.8407

G+D 0.6844

O+D 0.6528

R+D 0.5406

P 0.5240

O 0.4226

G 0.2115

R 0.1115

T 0.1074

26、The outcomes after the article of “Noda K 2002” was deleted.

Acute nausea

| A+D | . | . | 1.16 (0.67; 2.00) | . | . | . | . | . | . | . | . |
| --- | --- | --- | --- | --- | --- | --- | --- | --- | --- | --- | --- |
| 0.89 (0.49; 1.61) | Do | 0.98 (0.85; 1.13) | . | 0.99 (0.68; 1.43) | . | . | . | . | . | . | . |
| 0.87 (0.49; 1.55) | 0.98 (0.86; 1.12) | G | 1.32 (1.06; 1.63) | 1.00 (0.93; 1.08) | . | . | . | 1.32 (0.96; 1.82) | . | 0.41 (0.23; 0.73) | . |
| 1.16 (0.67; 2.00) | 1.31 (1.03; 1.65) | 1.33 (1.10; 1.62) | G+D | . | 1.04 (0.91; 1.19) | . | 0.97 (0.84; 1.13) | . | 1.22 (0.66; 2.23) | . | . |
| 0.86 (0.48; 1.55) | 0.97 (0.84; 1.12) | 0.99 (0.92; 1.07) | 0.74 (0.61; 0.91) | O | 1.26 (0.74; 2.13) | 1.33 (0.80; 2.21) | . | . | . | 1.10 (0.78; 1.56) | . |
| 1.15 (0.66; 2.01) | 1.30 (1.01; 1.67) | 1.32 (1.06; 1.64) | 0.99 (0.88; 1.11) | 1.33 (1.06; 1.67) | O+D | . | 1.14 (0.93; 1.39) | . | . | . | . |
| 1.15 (0.53; 2.48) | 1.29 (0.77; 2.19) | 1.32 (0.79; 2.20) | 0.99 (0.58; 1.71) | 1.33 (0.80; 2.21) | 1.00 (0.57; 1.73) | P | . | . | . | . | . |
| 1.19 (0.68; 2.08) | 1.34 (1.03; 1.74) | 1.37 (1.09; 1.72) | 1.03 (0.90; 1.16) | 1.38 (1.09; 1.74) | 1.03 (0.90; 1.19) | 1.03 (0.59; 1.81) | P+D | . | . | . | . |
| 1.10 (0.58; 2.08) | 1.24 (0.90; 1.70) | 1.26 (0.94; 1.68) | 0.95 (0.68; 1.32) | 1.27 (0.94; 1.71) | 0.95 (0.67; 1.35) | 0.95 (0.53; 1.72) | 0.92 (0.65; 1.31) | R | 1.61 (1.16; 2.25) | . | . |
| 1.68 (0.86; 3.28) | 1.89 (1.27; 2.81) | 1.93 (1.33; 2.80) | 1.45 (0.98; 2.13) | 1.94 (1.33; 2.84) | 1.46 (0.98; 2.18) | 1.46 (0.78; 2.75) | 1.41 (0.94; 2.12) | 1.53 (1.13; 2.07) | R+D | . | . |
| 0.74 (0.38; 1.41) | 0.83 (0.59; 1.15) | 0.84 (0.62; 1.14) | 0.63 (0.44; 0.91) | 0.85 (0.63; 1.15) | 0.64 (0.44; 0.93) | 0.64 (0.36; 1.15) | 0.62 (0.42; 0.90) | 0.67 (0.44; 1.02) | 0.44 (0.27; 0.71) | T | 1.85 (1.25; 2.75) |
| 1.36 (0.64; 2.92) | 1.53 (0.92; 2.56) | 1.56 (0.95; 2.57) | 1.17 (0.69; 2.00) | 1.58 (0.96; 2.59) | 1.18 (0.69; 2.03) | 1.19 (0.58; 2.40) | 1.15 (0.66; 1.98) | 1.24 (0.70; 2.21) | 0.81 (0.44; 1.51) | 1.85 (1.25; 2.75) | T+D |

P-score

R+D 0.9496

T+D 0.7733

P+D 0.6919

G+D 0.6389

O+D 0.6217

P 0.5968

R 0.5568

A+D 0.4311

Do 0.2588

G 0.2195

O 0.1970

T 0.0645

Acute vomiting

| Do | 0.96 (0.79; 1.15) | . | 0.99 (0.84; 1.15) | . | . | . | . | . | . | . |
| --- | --- | --- | --- | --- | --- | --- | --- | --- | --- | --- |
| 0.93 (0.82; 1.06) | G | 1.57 (1.28; 1.91) | 1.09 (0.99; 1.19) | . | 1.61 (0.94; 2.77) | . | 1.32 (0.93; 1.87) | . | 0.91 (0.65; 1.27) | . |
| 1.46 (1.18; 1.81) | 1.57 (1.32; 1.87) | G+D | . | 1.06 (0.88; 1.28) | . | 1.05 (0.81; 1.36) | . | 0.84 (0.48; 1.47) | . | . |
| 1.01 (0.89; 1.14) | 1.08 (0.99; 1.18) | 0.69 (0.57; 0.83) | O | 1.28 (0.88; 1.85) | 1.98 (0.60; 6.53) | . | . | . | 0.78 (0.60; 1.03) | . |
| 1.42 (1.11; 1.80) | 1.52 (1.23; 1.88) | 0.97 (0.83; 1.13) | 1.41 (1.13; 1.75) | O+D | . | 1.32 (1.08; 1.60) | . | . | . | . |
| 1.57 (0.95; 2.62) | 1.69 (1.03; 2.77) | 1.08 (0.64; 1.82) | 1.56 (0.95; 2.58) | 1.11 (0.65; 1.90) | P | . | . | . | . | . |
| 1.74 (1.33; 2.28) | 1.87 (1.47; 2.38) | 1.19 (0.99; 1.43) | 1.73 (1.35; 2.22) | 1.23 (1.04; 1.45) | 1.10 (0.64; 1.91) | P+D | . | . | . | . |
| 1.12 (0.80; 1.57) | 1.20 (0.88; 1.64) | 0.77 (0.55; 1.08) | 1.11 (0.81; 1.54) | 0.79 (0.55; 1.14) | 0.71 (0.40; 1.28) | 0.64 (0.44; 0.94) | R | 1.58 (1.05; 2.38) | . | . |
| 1.56 (1.03; 2.36) | 1.68 (1.13; 2.49) | 1.07 (0.72; 1.59) | 1.55 (1.04; 2.32) | 1.10 (0.72; 1.67) | 0.99 (0.53; 1.86) | 0.90 (0.58; 1.38) | 1.39 (0.98; 1.98) | R+D | . | . |
| 0.79 (0.61; 1.03) | 0.85 (0.67; 1.08) | 0.54 (0.40; 0.73) | 0.79 (0.62; 1.00) | 0.56 (0.41; 0.77) | 0.50 (0.29; 0.87) | 0.46 (0.33; 0.64) | 0.71 (0.48; 1.05) | 0.51 (0.32; 0.81) | T | 1.99 (1.42; 2.79) |
| 1.58 (1.03; 2.43) | 1.70 (1.12; 2.57) | 1.08 (0.69; 1.69) | 1.57 (1.04; 2.37) | 1.12 (0.70; 1.77) | 1.00 (0.53; 1.91) | 0.91 (0.56; 1.47) | 1.41 (0.84; 2.37) | 1.01 (0.57; 1.80) | 1.99 (1.42; 2.79) | T+D |

P-score

P+D 0.8924

T+D 0.7557

R+D 0.7506

P 0.7398

G+D 0.6752

O+D 0.6241

R 0.3747

O 0.2807

Do 0.2619

G 0.1230

T 0.0217

27、 The outcomes after the article of “Öge A 2000” was deleted.

Acute vomiting

| Do | 0.96 (0.80; 1.15) | . | 0.99 (0.85; 1.14) | . | . | . | . | . | . | . |
| --- | --- | --- | --- | --- | --- | --- | --- | --- | --- | --- |
| 0.94 (0.82; 1.06) | G | 1.57 (1.29; 1.91) | 1.08 (0.98; 1.18) | . | 1.61 (0.94; 2.76) | . | 1.32 (0.94; 1.86) | . | 0.46 (0.21; 1.01) | . |
| 1.46 (1.19; 1.80) | 1.56 (1.32; 1.86) | G+D | . | 1.06 (0.88; 1.28) | . | 1.05 (0.81; 1.35) | . | 0.84 (0.48; 1.46) | . | . |
| 1.00 (0.89; 1.13) | 1.07 (0.98; 1.16) | 0.68 (0.57; 0.82) | O | 1.27 (0.88; 1.84) | 1.98 (0.60; 6.52) | . | 1.06 (0.74; 1.52) | . | 0.76 (0.54; 1.07) | . |
| 1.42 (1.12; 1.80) | 1.52 (1.23; 1.87) | 0.97 (0.84; 1.12) | 1.42 (1.14; 1.75) | O+D | . | 1.32 (1.09; 1.60) | . | . | . | . |
| 1.58 (0.95; 2.62) | 1.69 (1.03; 2.76) | 1.08 (0.64; 1.82) | 1.58 (0.96; 2.59) | 1.11 (0.65; 1.90) | P | . | . | . | . | . |
| 1.74 (1.33; 2.27) | 1.86 (1.47; 2.36) | 1.19 (0.99; 1.42) | 1.74 (1.36; 2.22) | 1.23 (1.04; 1.44) | 1.10 (0.64; 1.90) | P+D | . | . | . | . |
| 1.10 (0.85; 1.43) | 1.17 (0.93; 1.49) | 0.75 (0.57; 0.99) | 1.10 (0.86; 1.39) | 0.77 (0.57; 1.05) | 0.70 (0.40; 1.20) | 0.63 (0.46; 0.87) | R | 1.58 (1.06; 2.37) | . | . |
| 1.54 (1.05; 2.27) | 1.65 (1.14; 2.38) | 1.05 (0.73; 1.53) | 1.54 (1.06; 2.23) | 1.09 (0.73; 1.61) | 0.98 (0.53; 1.80) | 0.89 (0.59; 1.34) | 1.40 (1.00; 1.97) | R+D | . | . |
| 0.69 (0.50; 0.97) | 0.74 (0.54; 1.03) | 0.47 (0.33; 0.68) | 0.69 (0.51; 0.95) | 0.49 (0.33; 0.72) | 0.44 (0.24; 0.79) | 0.40 (0.27; 0.59) | 0.63 (0.43; 0.94) | 0.45 (0.28; 0.73) | T | 1.99 (1.43; 2.78) |
| 1.38 (0.86; 2.22) | 1.48 (0.93; 2.35) | 0.94 (0.58; 1.55) | 1.38 (0.87; 2.19) | 0.97 (0.59; 1.62) | 0.87 (0.45; 1.72) | 0.79 (0.47; 1.34) | 1.26 (0.75; 2.11) | 0.90 (0.50; 1.62) | 1.99 (1.43; 2.78) | T+D |

P-score

P+D 0.9118

P 0.7623

R+D 0.7613

G+D 0.7034

O+D 0.6520

T+D 0.6352

R 0.3816

O 0.2791

Do 0.2716

G 0.1337

T 0.0079

Delayed vomiting

| Do | 2.33 (1.53; 3.57) | . | . | 1.27 (0.79; 2.04) | . | . | . | . | . | . | . |
| --- | --- | --- | --- | --- | --- | --- | --- | --- | --- | --- | --- |
| 2.33 (1.53; 3.57) | Do+D | . | . | . | . | . | . | . | . | . | . |
| 1.50 (0.84; 2.67) | 0.64 (0.31; 1.32) | G | 0.93 (0.61; 1.42) | 1.06 (0.66; 1.70) | . | 1.12 (0.72; 1.73) | . | . | . | . | . |
| 1.43 (0.79; 2.59) | 0.61 (0.30; 1.27) | 0.96 (0.68; 1.35) | G+D | . | 0.99 (0.69; 1.43) | . | 1.61 (1.20; 2.17) | . | 0.97 (0.54; 1.74) | . | . |
| 1.27 (0.79; 2.04) | 0.54 (0.29; 1.03) | 0.85 (0.61; 1.17) | 0.89 (0.62; 1.26) | O | 1.28 (0.89; 1.86) | 1.87 (1.16; 3.03) | . | . | . | 0.96 (0.65; 1.42) | . |
| 1.60 (0.90; 2.83) | 0.68 (0.34; 1.40) | 1.06 (0.74; 1.53) | 1.11 (0.88; 1.41) | 1.26 (0.92; 1.73) | O+D | . | 1.33 (1.10; 1.61) | . | . | . | . |
| 1.96 (1.07; 3.59) | 0.84 (0.40; 1.76) | 1.31 (0.92; 1.87) | 1.37 (0.88; 2.14) | 1.55 (1.07; 2.24) | 1.23 (0.79; 1.92) | P | . | . | . | . | . |
| 2.17 (1.20; 3.92) | 0.93 (0.45; 1.93) | 1.45 (1.00; 2.11) | 1.52 (1.20; 1.92) | 1.71 (1.21; 2.43) | 1.36 (1.14; 1.62) | 1.11 (0.70; 1.75) | P+D | . | . | . | . |
| 0.86 (0.34; 2.16) | 0.37 (0.13; 1.02) | 0.57 (0.26; 1.26) | 0.60 (0.29; 1.22) | 0.68 (0.31; 1.49) | 0.54 (0.25; 1.14) | 0.44 (0.19; 1.01) | 0.39 (0.19; 0.83) | R | 1.63 (1.09; 2.44) | . | . |
| 1.39 (0.61; 3.20) | 0.60 (0.24; 1.52) | 0.93 (0.47; 1.83) | 0.97 (0.54; 1.74) | 1.10 (0.56; 2.17) | 0.87 (0.47; 1.64) | 0.71 (0.34; 1.48) | 0.64 (0.34; 1.20) | 1.63 (1.09; 2.44) | R+D | . | . |
| 1.22 (0.66; 2.27) | 0.52 (0.25; 1.11) | 0.82 (0.49; 1.36) | 0.85 (0.50; 1.44) | 0.96 (0.65; 1.42) | 0.77 (0.46; 1.27) | 0.62 (0.36; 1.07) | 0.56 (0.33; 0.95) | 1.43 (0.59; 3.45) | 0.88 (0.40; 1.92) | T | 1.59 (1.09; 2.31) |
| 1.95 (0.95; 4.00) | 0.83 (0.36; 1.92) | 1.30 (0.69; 2.44) | 1.36 (0.71; 2.59) | 1.53 (0.89; 2.63) | 1.22 (0.65; 2.28) | 0.99 (0.52; 1.91) | 0.90 (0.47; 1.70) | 2.27 (0.87; 5.92) | 1.40 (0.59; 3.33) | 1.59 (1.09; 2.31) | T+D |

P-score

P+D 0.8714

Do+D 0.8471

P 0.7783

T+D 0.7421

O+D 0.5788

G 0.4935

R+D 0.4414

G+D 0.4324

O 0.2933

T 0.2799

Do 0.1436

R 0.0982

28、The outcomes after the article of “Olver I 1996” was deleted.

②Acute vomiting

| Do | 0.96 (0.82; 1.12) | . | 0.98 (0.87; 1.10) | . | . | . | . | . | . | . |
| --- | --- | --- | --- | --- | --- | --- | --- | --- | --- | --- |
| 0.93 (0.84; 1.03) | G | 1.57 (1.32; 1.88) | 1.08 (1.00; 1.17) | . | 1.61 (0.96; 2.72) | . | 1.32 (0.96; 1.81) | . | 0.92 (0.67; 1.26) | . |
| 1.56 (1.29; 1.89) | 1.68 (1.42; 1.98) | G+D | . | 1.07 (0.91; 1.25) | . | 1.05 (0.85; 1.30) | . | 0.84 (0.49; 1.45) | . | . |
| 1.00 (0.90; 1.10) | 1.07 (1.00; 1.14) | 0.64 (0.53; 0.76) | O | 2.44 (1.41; 4.24) | 1.98 (0.61; 6.50) | . | 1.06 (0.76; 1.48) | . | 0.78 (0.61; 1.00) | . |
| 1.59 (1.27; 2.00) | 1.71 (1.39; 2.10) | 1.02 (0.89; 1.17) | 1.60 (1.30; 1.98) | O+D | . | 1.32 (1.10; 1.58) | . | . | . | . |
| 1.57 (0.96; 2.56) | 1.68 (1.04; 2.72) | 1.01 (0.61; 1.67) | 1.58 (0.97; 2.55) | 0.98 (0.59; 1.66) | P | . | . | . | . | . |
| 1.90 (1.49; 2.43) | 2.04 (1.63; 2.55) | 1.22 (1.04; 1.43) | 1.91 (1.51; 2.41) | 1.19 (1.03; 1.38) | 1.21 (0.71; 2.05) | P+D | . | . | . | . |
| 1.11 (0.87; 1.40) | 1.19 (0.95; 1.48) | 0.71 (0.55; 0.92) | 1.11 (0.89; 1.38) | 0.69 (0.52; 0.93) | 0.70 (0.42; 1.19) | 0.58 (0.43; 0.79) | R | 1.58 (1.08; 2.31) | . | . |
| 1.59 (1.11; 2.29) | 1.71 (1.20; 2.43) | 1.02 (0.71; 1.46) | 1.60 (1.13; 2.28) | 1.00 (0.68; 1.46) | 1.02 (0.56; 1.84) | 0.84 (0.57; 1.24) | 1.44 (1.04; 1.99) | R+D | . | . |
| 0.79 (0.63; 1.01) | 0.85 (0.68; 1.06) | 0.51 (0.39; 0.67) | 0.80 (0.64; 0.99) | 0.50 (0.37; 0.67) | 0.51 (0.30; 0.86) | 0.42 (0.31; 0.57) | 0.72 (0.53; 0.98) | 0.50 (0.33; 0.75) | T | 1.98 (1.45; 2.72) |
| 1.58 (1.06; 2.34) | 1.69 (1.15; 2.48) | 1.01 (0.67; 1.53) | 1.58 (1.08; 2.32) | 0.99 (0.64; 1.53) | 1.00 (0.54; 1.85) | 0.83 (0.53; 1.29) | 1.43 (0.92; 2.21) | 0.99 (0.59; 1.66) | 1.98 (1.45; 2.72) | T+D |

P-score

P+D 0.9348

R+D 0.7250

O+D 0.7167

T+D 0.7110

P 0.7014

G+D 0.6808

R 0.3722

Do 0.2674

O 0.2631

G 0.1127

T 0.0150

Acute complete control

| G | 0.80 (0.61; 1.04) | 0.96 (0.89; 1.03) | . | . | . | . | . | 1.16 (0.92; 1.47) | . |
| --- | --- | --- | --- | --- | --- | --- | --- | --- | --- |
| 0.79 (0.65; 0.96) | G+D | . | 0.99 (0.92; 1.06) | . | 0.98 (0.91; 1.05) | . | 1.05 (0.87; 1.27) | . | . |
| 0.96 (0.89; 1.03) | 1.21 (0.99; 1.48) | O | 0.82 (0.61; 1.08) | 0.98 (0.91; 1.05) | . | . | . | 1.13 (0.89; 1.44) | . |
| 0.79 (0.65; 0.97) | 1.00 (0.95; 1.07) | 0.83 (0.68; 1.01) | O+D | . | 0.93 (0.84; 1.03) | . | . | . | . |
| 0.93 (0.84; 1.04) | 1.18 (0.96; 1.46) | 0.98 (0.91; 1.05) | 1.18 (0.95; 1.46) | P | . | . | . | . | . |
| 0.76 (0.62; 0.93) | 0.96 (0.91; 1.02) | 0.79 (0.65; 0.98) | 0.96 (0.89; 1.03) | 0.81 (0.65; 1.01) | P+D | . | . | . | . |
| 1.05 (0.75; 1.47) | 1.33 (1.01; 1.74) | 1.09 (0.78; 1.53) | 1.32 (1.00; 1.75) | 1.12 (0.79; 1.58) | 1.38 (1.04; 1.82) | R | 0.79 (0.65; 0.96) | . | . |
| 0.83 (0.63; 1.09) | 1.05 (0.87; 1.27) | 0.87 (0.66; 1.14) | 1.05 (0.86; 1.28) | 0.89 (0.67; 1.18) | 1.09 (0.89; 1.34) | 0.79 (0.65; 0.96) | R+D | . | . |
| 1.12 (0.90; 1.40) | 1.42 (1.07; 1.91) | 1.17 (0.94; 1.46) | 1.42 (1.06; 1.90) | 1.20 (0.95; 1.52) | 1.48 (1.10; 1.99) | 1.07 (0.72; 1.60) | 1.36 (0.96; 1.92) | T | 0.65 (0.53; 0.80) |
| 0.73 (0.54; 0.99) | 0.92 (0.65; 1.32) | 0.76 (0.56; 1.03) | 0.92 (0.64; 1.32) | 0.78 (0.57; 1.07) | 0.96 (0.67; 1.38) | 0.70 (0.45; 1.09) | 0.88 (0.59; 1.32) | 0.65 (0.53; 0.80) | T+D |

P-score

P+D 0.8784

T+D 0.8313

G+D 0.7293

O+D 0.7158

R+D 0.6210

P 0.4161

O 0.3344

G 0.2038

R 0.1912

T 0.0787

Delayed nausea

| Do | 1.99 (1.26; 3.14) | . | . | 0.91 (0.62; 1.34) | . | . | . | . | . | . | . |
| --- | --- | --- | --- | --- | --- | --- | --- | --- | --- | --- | --- |
| 1.99 (1.26; 3.14) | Do+D | . | . | . | . | . | . | . | . | . | . |
| 1.00 (0.56; 1.81) | 0.50 (0.24; 1.06) | G | 0.97 (0.66; 1.43) | 1.17 (0.67; 2.05) | . | . | . | . | . | . | . |
| 1.10 (0.60; 2.01) | 0.55 (0.26; 1.18) | 1.10 (0.77; 1.56) | G+D | . | 1.07 (0.75; 1.54) | . | 1.42 (1.08; 1.87) | . | 1.02 (0.63; 1.64) | . | . |
| 0.91 (0.62; 1.34) | 0.46 (0.25; 0.83) | 0.91 (0.58; 1.43) | 0.83 (0.52; 1.32) | O | 1.77 (0.99; 3.17) | 1.42 (1.03; 1.97) | . | . | . | 1.21 (0.78; 1.86) | . |
| 1.23 (0.68; 2.23) | 0.62 (0.29; 1.31) | 1.22 (0.82; 1.84) | 1.12 (0.87; 1.44) | 1.34 (0.85; 2.12) | O+D | . | 1.37 (1.04; 1.80) | . | . | . | . |
| 1.30 (0.78; 2.15) | 0.65 (0.33; 1.29) | 1.30 (0.74; 2.25) | 1.18 (0.67; 2.09) | 1.42 (1.03; 1.97) | 1.06 (0.60; 1.85) | P | . | . | . | . | . |
| 1.62 (0.87; 3.01) | 0.81 (0.38; 1.76) | 1.62 (1.07; 2.43) | 1.47 (1.17; 1.86) | 1.77 (1.09; 2.88) | 1.32 (1.05; 1.66) | 1.25 (0.70; 2.23) | P+D | . | . | . | . |
| 0.73 (0.30; 1.74) | 0.37 (0.14; 0.98) | 0.73 (0.35; 1.49) | 0.66 (0.35; 1.24) | 0.80 (0.37; 1.74) | 0.59 (0.30; 1.17) | 0.56 (0.24; 1.31) | 0.45 (0.23; 0.88) | R | 1.53 (1.03; 2.29) | . | . |
| 1.12 (0.52; 2.42) | 0.56 (0.23; 1.38) | 1.11 (0.61; 2.02) | 1.02 (0.63; 1.64) | 1.22 (0.63; 2.39) | 0.91 (0.53; 1.57) | 0.86 (0.41; 1.81) | 0.69 (0.40; 1.18) | 1.53 (1.03; 2.29) | R+D | . | . |
| 1.10 (0.62; 1.97) | 0.55 (0.27; 1.16) | 1.10 (0.59; 2.05) | 1.00 (0.53; 1.89) | 1.21 (0.78; 1.86) | 0.90 (0.48; 1.68) | 0.85 (0.49; 1.46) | 0.68 (0.36; 1.30) | 1.51 (0.62; 3.70) | 0.99 (0.44; 2.19) | T | 1.49 (0.98; 2.26) |
| 1.64 (0.80; 3.35) | 0.82 (0.35; 1.92) | 1.63 (0.77; 3.46) | 1.49 (0.70; 3.19) | 1.79 (0.98; 3.28) | 1.33 (0.63; 2.84) | 1.26 (0.64; 2.50) | 1.01 (0.47; 2.19) | 2.25 (0.84; 6.03) | 1.47 (0.60; 3.61) | 1.49 (0.98; 2.26) | T+D |

P-score

Do+D 0.8972

P+D 0.8409

T+D 0.7910

P 0.6204

O+D 0.5677

R+D 0.4629

T 0.4288

G+D 0.4246

Do 0.3307

G 0.3192

O 0.2028

R 0.1138

Delayed vomiting

| Do | 2.33 (1.52; 3.59) | . | . | 1.27 (0.78; 2.06) | . | . | . | . | . | . | . |
| --- | --- | --- | --- | --- | --- | --- | --- | --- | --- | --- | --- |
| 2.33 (1.52; 3.59) | Do+D | . | . | . | . | . | . | . | . | . | . |
| 1.38 (0.79; 2.42) | 0.59 (0.29; 1.20) | G | 0.93 (0.61; 1.42) | 1.09 (0.76; 1.57) | . | 1.12 (0.71; 1.74) | . | . | . | 1.14 (0.66; 1.99) | . |
| 1.41 (0.74; 2.69) | 0.60 (0.28; 1.32) | 1.02 (0.70; 1.49) | G+D | . | 0.99 (0.68; 1.44) | . | 1.62 (1.20; 2.20) | . | 0.97 (0.54; 1.75) | . | . |
| 1.27 (0.78; 2.06) | 0.54 (0.29; 1.04) | 0.92 (0.69; 1.23) | 0.90 (0.58; 1.38) | O | 1.66 (0.82; 3.38) | 1.87 (1.15; 3.03) | . | . | . | 0.98 (0.70; 1.35) | . |
| 1.62 (0.83; 3.13) | 0.69 (0.31; 1.52) | 1.17 (0.77; 1.78) | 1.15 (0.89; 1.47) | 1.27 (0.81; 2.00) | O+D | . | 1.33 (1.09; 1.61) | . | . | . | . |
| 1.88 (1.03; 3.44) | 0.81 (0.38; 1.69) | 1.36 (0.96; 1.94) | 1.33 (0.81; 2.19) | 1.48 (1.03; 2.13) | 1.16 (0.69; 1.97) | P | . | . | . | . | . |
| 2.19 (1.12; 4.26) | 0.94 (0.42; 2.07) | 1.59 (1.04; 2.42) | 1.55 (1.22; 1.97) | 1.72 (1.09; 2.73) | 1.35 (1.13; 1.62) | 1.16 (0.68; 1.98) | P+D | . | . | . | . |
| 0.84 (0.32; 2.22) | 0.36 (0.13; 1.04) | 0.61 (0.27; 1.38) | 0.60 (0.29; 1.22) | 0.66 (0.29; 1.53) | 0.52 (0.24; 1.12) | 0.45 (0.19; 1.08) | 0.39 (0.18; 0.82) | R | 1.63 (1.08; 2.45) | . | . |
| 1.37 (0.57; 3.29) | 0.59 (0.22; 1.56) | 1.00 (0.49; 2.00) | 0.97 (0.54; 1.75) | 1.08 (0.52; 2.24) | 0.85 (0.45; 1.61) | 0.73 (0.34; 1.58) | 0.63 (0.33; 1.18) | 1.63 (1.08; 2.45) | R+D | . | . |
| 1.30 (0.73; 2.31) | 0.56 (0.27; 1.14) | 0.94 (0.64; 1.38) | 0.92 (0.55; 1.53) | 1.03 (0.75; 1.40) | 0.80 (0.47; 1.37) | 0.69 (0.44; 1.10) | 0.60 (0.35; 1.02) | 1.54 (0.64; 3.71) | 0.95 (0.44; 2.06) | T | 1.59 (1.09; 2.32) |
| 2.07 (1.04; 4.13) | 0.89 (0.39; 2.00) | 1.50 (0.88; 2.57) | 1.47 (0.78; 2.77) | 1.63 (1.00; 2.67) | 1.28 (0.67; 2.46) | 1.10 (0.61; 2.00) | 0.95 (0.49; 1.83) | 2.45 (0.94; 6.39) | 1.51 (0.64; 3.58) | 1.59 (1.09; 2.32) | T+D |

P-score

P+D 0.8694

Do+D 0.8433

T+D 0.7974

P 0.7400

O+D 0.5939

R+D 0.4329

G+D 0.4233

G 0.4068

T 0.3433

O 0.3049

Do 0.1484

R 0.0965

Delayed complete control

| G | 1.12 (0.73; 1.71) | . | . | . | . | . |
| --- | --- | --- | --- | --- | --- | --- |
| 1.12 (0.73; 1.71) | G+D | . | 0.94 (0.72; 1.23) | . | 0.80 (0.62; 1.04) | 0.92 (0.57; 1.50) |
| 1.77 (0.94; 3.33) | 1.58 (0.99; 2.53) | O | 0.63 (0.42; 0.96) | 0.70 (0.48; 1.02) | . | . |
| 1.12 (0.70; 1.81) | 1.01 (0.81; 1.24) | 0.63 (0.42; 0.96) | O+D | . | 0.70 (0.55; 0.90) | . |
| 1.23 (0.59; 2.58) | 1.11 (0.61; 2.02) | 0.70 (0.48; 1.02) | 1.10 (0.63; 1.93) | P | . | . |
| 0.84 (0.52; 1.35) | 0.75 (0.61; 0.93) | 0.47 (0.30; 0.75) | 0.75 (0.61; 0.91) | 0.68 (0.37; 1.23) | P+D | . |
| 1.03 (0.54; 1.97) | 0.92 (0.57; 1.50) | 0.58 (0.30; 1.15) | 0.92 (0.54; 1.56) | 0.84 (0.39; 1.81) | 1.23 (0.73; 2.09) | R+D |

p-score

P+D 0.9066

G 0.6361

R+D 0.5907

G+D 0.4677

O+D 0.4659

P 0.4040

O 0.0290

29、The outcomes after the article of “Roila F 1991” was deleted.

Acute vomiting

| Do | 0.96 (0.81; 1.13) | . | 0.98 (0.86; 1.12) | . | . | . | . | . | . | . |
| --- | --- | --- | --- | --- | --- | --- | --- | --- | --- | --- |
| 0.92 (0.82; 1.04) | G | 1.57 (1.30; 1.89) | 1.08 (1.00; 1.18) | . | 1.61 (0.95; 2.74) | . | 1.32 (0.95; 1.83) | . | 0.92 (0.66; 1.27) | . |
| 1.41 (1.16; 1.72) | 1.53 (1.29; 1.80) | G+D | . | 1.06 (0.90; 1.26) | . | 1.05 (0.83; 1.32) | . | 0.84 (0.49; 1.46) | . | . |
| 1.00 (0.90; 1.12) | 1.09 (1.01; 1.17) | 0.71 (0.60; 0.85) | O | 1.06 (0.72; 1.56) | 1.98 (0.60; 6.51) | . | 1.06 (0.75; 1.49) | . | 0.78 (0.60; 1.01) | . |
| 1.35 (1.08; 1.70) | 1.46 (1.20; 1.79) | 0.96 (0.83; 1.10) | 1.35 (1.10; 1.66) | O+D | . | 1.32 (1.10; 1.59) | . | . | . | . |
| 1.56 (0.95; 2.57) | 1.69 (1.04; 2.74) | 1.11 (0.66; 1.85) | 1.56 (0.96; 2.53) | 1.16 (0.68; 1.95) | P | . | . | . | . | . |
| 1.66 (1.29; 2.13) | 1.80 (1.43; 2.25) | 1.18 (0.99; 1.39) | 1.65 (1.31; 2.09) | 1.23 (1.05; 1.43) | 1.06 (0.62; 1.81) | P+D | . | . | . | . |
| 1.09 (0.85; 1.40) | 1.18 (0.94; 1.48) | 0.77 (0.59; 1.01) | 1.09 (0.87; 1.37) | 0.81 (0.60; 1.08) | 0.70 (0.41; 1.19) | 0.66 (0.48; 0.90) | R | 1.58 (1.07; 2.34) | . | . |
| 1.52 (1.05; 2.21) | 1.65 (1.15; 2.36) | 1.08 (0.75; 1.56) | 1.52 (1.06; 2.18) | 1.13 (0.77; 1.66) | 0.98 (0.53; 1.78) | 0.92 (0.62; 1.37) | 1.40 (1.00; 1.94) | R+D | . | . |
| 0.79 (0.62; 1.02) | 0.86 (0.68; 1.08) | 0.56 (0.43; 0.74) | 0.79 (0.63; 0.99) | 0.59 (0.44; 0.79) | 0.51 (0.30; 0.87) | 0.48 (0.35; 0.66) | 0.73 (0.53; 1.00) | 0.52 (0.34; 0.79) | T | 1.99 (1.44; 2.75) |
| 1.58 (1.05; 2.37) | 1.71 (1.15; 2.54) | 1.12 (0.73; 1.71) | 1.57 (1.06; 2.33) | 1.17 (0.75; 1.81) | 1.01 (0.54; 1.88) | 0.95 (0.60; 1.50) | 1.45 (0.92; 2.27) | 1.04 (0.61; 1.76) | 1.99 (1.44; 2.75) | T+D |

P-score

P+D 0.8798

T+D 0.7842

P 0.7596

R+D 0.7590

G+D 0.6717

O+D 0.6008

R 0.3703

O 0.2793

Do 0.2663

G 0.1108

T 0.0181

30、The outcomes after the article of “Ruff P 1994” was deleted.

Acute nausea

| A+D | . | . | 1.16 (0.67; 2.00) | . | . | . | . | . | . | . | . |
| --- | --- | --- | --- | --- | --- | --- | --- | --- | --- | --- | --- |
| 0.89 (0.49; 1.60) | Do | 0.98 (0.85; 1.12) | . | 0.99 (0.68; 1.42) | . | . | . | . | . | . | . |
| 0.87 (0.49; 1.55) | 0.98 (0.86; 1.11) | G | 1.32 (1.07; 1.63) | 1.02 (0.95; 1.10) | . | . | . | 1.32 (0.97; 1.81) | . | 0.41 (0.23; 0.73) | . |
| 1.16 (0.67; 2.00) | 1.30 (1.04; 1.64) | 1.33 (1.10; 1.61) | G+D | . | 1.04 (0.91; 1.19) | . | 0.97 (0.84; 1.12) | . | 1.22 (0.66; 2.23) | . | . |
| 0.88 (0.49; 1.57) | 0.99 (0.86; 1.14) | 1.01 (0.94; 1.09) | 0.76 (0.62; 0.93) | O | 1.26 (0.74; 2.12) | 1.33 (0.80; 2.20) | . | 1.16 (0.72; 1.85) | . | 1.10 (0.78; 1.56) | . |
| 1.15 (0.66; 2.01) | 1.30 (1.01; 1.66) | 1.33 (1.07; 1.64) | 0.99 (0.89; 1.11) | 1.31 (1.05; 1.64) | O+D | . | 1.14 (0.93; 1.38) | . | . | . | . |
| 1.17 (0.54; 2.52) | 1.32 (0.78; 2.22) | 1.35 (0.81; 2.24) | 1.01 (0.59; 1.74) | 1.33 (0.80; 2.20) | 1.02 (0.59; 1.76) | P | . | . | . | . | . |
| 1.19 (0.68; 2.07) | 1.33 (1.03; 1.72) | 1.36 (1.09; 1.70) | 1.02 (0.91; 1.16) | 1.35 (1.07; 1.70) | 1.03 (0.90; 1.18) | 1.01 (0.58; 1.76) | P+D | . | . | . | . |
| 1.08 (0.58; 2.00) | 1.21 (0.92; 1.59) | 1.24 (0.97; 1.58) | 0.93 (0.69; 1.25) | 1.22 (0.95; 1.57) | 0.93 (0.68; 1.27) | 0.92 (0.52; 1.61) | 0.91 (0.66; 1.24) | R | 1.61 (1.16; 2.24) | . | . |
| 1.66 (0.86; 3.19) | 1.86 (1.28; 2.70) | 1.90 (1.34; 2.70) | 1.43 (0.99; 2.06) | 1.88 (1.32; 2.68) | 1.43 (0.98; 2.10) | 1.41 (0.76; 2.62) | 1.39 (0.95; 2.05) | 1.54 (1.14; 2.07) | R+D | . | . |
| 0.75 (0.39; 1.43) | 0.84 (0.61; 1.16) | 0.86 (0.63; 1.16) | 0.64 (0.45; 0.92) | 0.85 (0.63; 1.14) | 0.65 (0.45; 0.93) | 0.64 (0.36; 1.14) | 0.63 (0.43; 0.91) | 0.69 (0.47; 1.02) | 0.45 (0.28; 0.72) | T | 1.85 (1.26; 2.74) |
| 1.39 (0.65; 2.95) | 1.56 (0.94; 2.59) | 1.59 (0.97; 2.60) | 1.19 (0.70; 2.02) | 1.57 (0.96; 2.57) | 1.20 (0.70; 2.05) | 1.18 (0.59; 2.39) | 1.17 (0.68; 2.00) | 1.29 (0.74; 2.23) | 0.84 (0.46; 1.53) | 1.85 (1.26; 2.74) | T+D |

P-score

R+D 0.9459

T+D 0.7884

P+D 0.6894

G+D 0.6398

O+D 0.6258

P 0.6156

R 0.5308

A+D 0.4271

Do 0.2481

O 0.2262

G 0.1937

T 0.0689

Acute vomiting

| Do | 0.96 (0.80; 1.15) | . | 0.99 (0.85; 1.15) | . | . | . | . | . | . | . |
| --- | --- | --- | --- | --- | --- | --- | --- | --- | --- | --- |
| 0.92 (0.81; 1.04) | G | 1.57 (1.29; 1.91) | 1.12 (1.01; 1.24) | . | 1.61 (0.94; 2.76) | . | 1.32 (0.94; 1.86) | . | 0.91 (0.65; 1.27) | . |
| 1.44 (1.17; 1.78) | 1.58 (1.33; 1.87) | G+D | . | 1.06 (0.88; 1.28) | . | 1.05 (0.81; 1.35) | . | 0.84 (0.48; 1.46) | . | . |
| 1.01 (0.90; 1.15) | 1.11 (1.01; 1.21) | 0.70 (0.58; 0.85) | O | 1.27 (0.88; 1.85) | 1.98 (0.60; 6.52) | . | 1.06 (0.74; 1.52) | . | 0.78 (0.60; 1.03) | . |
| 1.41 (1.11; 1.78) | 1.53 (1.24; 1.89) | 0.97 (0.84; 1.13) | 1.38 (1.12; 1.72) | O+D | . | 1.32 (1.09; 1.60) | . | . | . | . |
| 1.56 (0.94; 2.58) | 1.70 (1.04; 2.78) | 1.08 (0.64; 1.82) | 1.53 (0.93; 2.52) | 1.11 (0.65; 1.89) | P | . | . | . | . | . |
| 1.72 (1.32; 2.24) | 1.88 (1.48; 2.38) | 1.19 (0.99; 1.43) | 1.70 (1.33; 2.17) | 1.22 (1.04; 1.44) | 1.11 (0.64; 1.91) | P+D | . | . | . | . |
| 1.09 (0.84; 1.42) | 1.19 (0.94; 1.51) | 0.76 (0.57; 1.00) | 1.08 (0.85; 1.37) | 0.78 (0.57; 1.05) | 0.70 (0.41; 1.21) | 0.63 (0.46; 0.88) | R | 1.58 (1.06; 2.37) | . | . |
| 1.53 (1.04; 2.25) | 1.67 (1.15; 2.41) | 1.06 (0.73; 1.54) | 1.51 (1.04; 2.19) | 1.09 (0.73; 1.62) | 0.98 (0.53; 1.82) | 0.89 (0.59; 1.34) | 1.40 (1.00; 1.97) | R+D | . | . |
| 0.79 (0.61; 1.03) | 0.87 (0.68; 1.10) | 0.55 (0.41; 0.74) | 0.78 (0.62; 0.99) | 0.57 (0.41; 0.77) | 0.51 (0.30; 0.88) | 0.46 (0.33; 0.64) | 0.73 (0.52; 1.01) | 0.52 (0.34; 0.80) | T | 1.99 (1.42; 2.78) |
| 1.58 (1.04; 2.42) | 1.73 (1.14; 2.60) | 1.10 (0.70; 1.71) | 1.56 (1.04; 2.34) | 1.13 (0.71; 1.78) | 1.02 (0.54; 1.93) | 0.92 (0.57; 1.47) | 1.45 (0.91; 2.32) | 1.03 (0.60; 1.79) | 1.99 (1.42; 2.78) | T+D |

P-score

P+D 0.8950

T+D 0.7681

R+D 0.7439

P 0.7405

G+D 0.6755

O+D 0.6309

R 0.3636

O 0.2913

Do 0.2607

G 0.1087

T 0.0219

Acute complete control

| G | 0.80 (0.61; 1.04) | 0.97 (0.88; 1.06) | . | . | . | . | . | 1.16 (0.92; 1.47) | . |
| --- | --- | --- | --- | --- | --- | --- | --- | --- | --- |
| 0.87 (0.74; 1.02) | G+D | . | 0.99 (0.92; 1.06) | . | 0.98 (0.91; 1.05) | . | 1.05 (0.87; 1.27) | . | . |
| 0.96 (0.87; 1.05) | 1.11 (0.95; 1.29) | O | 0.94 (0.80; 1.12) | 0.98 (0.91; 1.05) | . | . | . | 1.13 (0.89; 1.44) | . |
| 0.87 (0.75; 1.02) | 1.01 (0.95; 1.07) | 0.91 (0.79; 1.06) | O+D | . | 0.93 (0.84; 1.03) | . | . | . | . |
| 0.93 (0.83; 1.05) | 1.08 (0.91; 1.28) | 0.98 (0.91; 1.05) | 1.07 (0.91; 1.26) | P | . | . | . | . | . |
| 0.84 (0.71; 0.99) | 0.96 (0.91; 1.03) | 0.87 (0.74; 1.02) | 0.96 (0.89; 1.03) | 0.89 (0.75; 1.07) | P+D | . | . | . | . |
| 1.15 (0.84; 1.58) | 1.33 (1.01; 1.74) | 1.20 (0.88; 1.64) | 1.32 (1.00; 1.74) | 1.23 (0.89; 1.69) | 1.37 (1.04; 1.82) | R | 0.79 (0.65; 0.96) | . | . |
| 0.91 (0.71; 1.17) | 1.05 (0.87; 1.27) | 0.95 (0.74; 1.22) | 1.04 (0.85; 1.27) | 0.97 (0.75; 1.26) | 1.09 (0.89; 1.33) | 0.79 (0.65; 0.96) | R+D | . | . |
| 1.12 (0.90; 1.40) | 1.30 (1.00; 1.69) | 1.17 (0.94; 1.47) | 1.29 (0.99; 1.67) | 1.20 (0.95; 1.52) | 1.35 (1.03; 1.76) | 0.98 (0.67; 1.43) | 1.24 (0.89; 1.71) | T | 0.65 (0.53; 0.80) |
| 0.73 (0.54; 0.99) | 0.84 (0.60; 1.18) | 0.76 (0.56; 1.03) | 0.84 (0.60; 1.17) | 0.78 (0.57; 1.07) | 0.87 (0.62; 1.23) | 0.64 (0.41; 0.98) | 0.80 (0.54; 1.18) | 0.65 (0.53; 0.80) | T+D |

P-score

T+D 0.9120

P+D 0.8424

G+D 0.6902

O+D 0.6529

R+D 0.5413

P 0.4948

O 0.3914

G 0.2562

T 0.1110

R 0.1078

31、The outcomes after the article of “Saito M 2009” was deleted.

Acute nausea

| A+D | . | . | 1.16 (0.67; 2.00) | . | . | . | . | . | . | . | . |
| --- | --- | --- | --- | --- | --- | --- | --- | --- | --- | --- | --- |
| 0.90 (0.50; 1.61) | Do | 0.98 (0.85; 1.12) | . | 0.99 (0.68; 1.42) | . | . | . | . | . | . | . |
| 0.88 (0.49; 1.56) | 0.98 (0.86; 1.11) | G | 1.32 (1.07; 1.63) | 1.00 (0.93; 1.07) | . | . | . | 1.32 (0.97; 1.81) | . | 0.41 (0.23; 0.73) | . |
| 1.16 (0.67; 2.00) | 1.30 (1.03; 1.63) | 1.32 (1.09; 1.60) | G+D | . | 1.04 (0.91; 1.19) | . | . | . | 1.22 (0.66; 2.23) | . | . |
| 0.87 (0.49; 1.55) | 0.97 (0.85; 1.12) | 0.99 (0.93; 1.06) | 0.75 (0.62; 0.92) | O | 1.26 (0.74; 2.12) | 1.33 (0.80; 2.20) | . | 1.16 (0.72; 1.85) | . | 1.10 (0.78; 1.56) | . |
| 1.20 (0.69; 2.10) | 1.34 (1.04; 1.73) | 1.37 (1.10; 1.71) | 1.04 (0.91; 1.18) | 1.38 (1.10; 1.73) | O+D | . | 1.14 (0.93; 1.38) | . | . | . | . |
| 1.16 (0.54; 2.50) | 1.30 (0.77; 2.19) | 1.32 (0.80; 2.20) | 1.00 (0.58; 1.72) | 1.33 (0.80; 2.20) | 0.97 (0.56; 1.68) | P | . | . | . | . | . |
| 1.37 (0.76; 2.46) | 1.52 (1.11; 2.10) | 1.56 (1.16; 2.09) | 1.18 (0.93; 1.48) | 1.57 (1.16; 2.11) | 1.14 (0.93; 1.38) | 1.18 (0.65; 2.11) | P+D | . | . | . | . |
| 1.08 (0.58; 2.00) | 1.20 (0.91; 1.59) | 1.23 (0.96; 1.57) | 0.93 (0.69; 1.25) | 1.24 (0.96; 1.59) | 0.90 (0.65; 1.23) | 0.93 (0.53; 1.63) | 0.79 (0.54; 1.14) | R | 1.61 (1.16; 2.24) | . | . |
| 1.66 (0.86; 3.19) | 1.85 (1.28; 2.69) | 1.89 (1.33; 2.68) | 1.43 (0.99; 2.06) | 1.90 (1.33; 2.71) | 1.38 (0.94; 2.03) | 1.43 (0.77; 2.64) | 1.21 (0.79; 1.87) | 1.54 (1.14; 2.07) | R+D | . | . |
| 0.74 (0.39; 1.42) | 0.83 (0.60; 1.15) | 0.85 (0.63; 1.14) | 0.64 (0.45; 0.91) | 0.85 (0.63; 1.15) | 0.62 (0.43; 0.90) | 0.64 (0.36; 1.15) | 0.54 (0.36; 0.83) | 0.69 (0.47; 1.01) | 0.45 (0.28; 0.71) | T | 1.85 (1.26; 2.74) |
| 1.38 (0.65; 2.94) | 1.54 (0.93; 2.56) | 1.57 (0.96; 2.57) | 1.19 (0.70; 2.01) | 1.58 (0.97; 2.58) | 1.15 (0.67; 1.96) | 1.19 (0.59; 2.40) | 1.01 (0.57; 1.79) | 1.28 (0.74; 2.21) | 0.83 (0.46; 1.52) | 1.85 (1.26; 2.74) | T+D |

P-score

R+D 0.9326

P+D 0.8118

T+D 0.7630

O+D 0.6530

G+D 0.5941

P 0.5851

R 0.5118

A+D 0.4124

Do 0.2568

G 0.2159

O 0.1984

T 0.065

Acute vomiting

| Do | 0.96 (0.80; 1.14) | . | 0.98 (0.85; 1.14) | . | . | . | . | . | . | . |
| --- | --- | --- | --- | --- | --- | --- | --- | --- | --- | --- |
| 0.93 (0.82; 1.05) | G | 1.57 (1.29; 1.90) | 1.09 (0.99; 1.19) | . | 1.61 (0.94; 2.75) | . | 1.32 (0.94; 1.85) | . | 0.91 (0.66; 1.27) | . |
| 1.44 (1.17; 1.77) | 1.55 (1.31; 1.84) | G+D | . | 1.06 (0.89; 1.27) | . | . | . | 0.84 (0.48; 1.46) | . | . |
| 1.01 (0.89; 1.13) | 1.08 (1.00; 1.18) | 0.70 (0.58; 0.84) | O | 1.27 (0.88; 1.83) | 1.98 (0.60; 6.52) | . | 1.06 (0.74; 1.51) | . | 0.78 (0.60; 1.02) | . |
| 1.48 (1.16; 1.88) | 1.59 (1.29; 1.97) | 1.03 (0.87; 1.21) | 1.47 (1.18; 1.82) | O+D | . | 1.32 (1.09; 1.59) | . | . | . | . |
| 1.57 (0.95; 2.59) | 1.69 (1.04; 2.76) | 1.09 (0.65; 1.83) | 1.56 (0.95; 2.55) | 1.06 (0.62; 1.81) | P | . | . | . | . | . |
| 1.94 (1.43; 2.64) | 2.10 (1.58; 2.79) | 1.35 (1.05; 1.74) | 1.93 (1.45; 2.58) | 1.32 (1.09; 1.59) | 1.24 (0.70; 2.18) | P+D | . | . | . | . |
| 1.09 (0.85; 1.41) | 1.18 (0.93; 1.49) | 0.76 (0.58; 1.00) | 1.09 (0.86; 1.38) | 0.74 (0.55; 1.00) | 0.70 (0.41; 1.20) | 0.56 (0.39; 0.81) | R | 1.58 (1.06; 2.36) | . | . |
| 1.53 (1.05; 2.24) | 1.65 (1.15; 2.38) | 1.07 (0.74; 1.54) | 1.52 (1.06; 2.20) | 1.04 (0.70; 1.54) | 0.98 (0.53; 1.80) | 0.79 (0.51; 1.22) | 1.40 (1.00; 1.96) | R+D | . | . |
| 0.79 (0.62; 1.02) | 0.86 (0.68; 1.08) | 0.55 (0.42; 0.74) | 0.79 (0.63; 0.99) | 0.54 (0.39; 0.73) | 0.51 (0.30; 0.87) | 0.41 (0.28; 0.59) | 0.73 (0.52; 1.00) | 0.52 (0.34; 0.80) | T | 1.99 (1.43; 2.77) |
| 1.58 (1.04; 2.40) | 1.70 (1.14; 2.55) | 1.10 (0.71; 1.70) | 1.57 (1.05; 2.35) | 1.07 (0.68; 1.69) | 1.01 (0.53; 1.90) | 0.81 (0.50; 1.33) | 1.44 (0.91; 2.30) | 1.03 (0.60; 1.77) | 1.99 (1.43; 2.77) | T+D |

P-score

P+D 0.9412

T+D 0.7445

P 0.7268

R+D 0.7217

O+D 0.6822

G+D 0.6431

R 0.3640

O 0.2795

Do 0.2632

G 0.1145

T 0.0192

Acute complete control

| G | 0.80 (0.61; 1.04) | 0.96 (0.89; 1.03) | . | . | . | . | . | 1.16 (0.92; 1.47) | . |
| --- | --- | --- | --- | --- | --- | --- | --- | --- | --- |
| 0.87 (0.74; 1.02) | G+D | . | 0.99 (0.92; 1.06) | . | . | . | 1.05 (0.87; 1.27) | . | . |
| 0.95 (0.88; 1.02) | 1.09 (0.94; 1.27) | O | 0.94 (0.80; 1.12) | 0.98 (0.91; 1.05) | . | . | . | 1.13 (0.89; 1.44) | . |
| 0.87 (0.74; 1.01) | 1.00 (0.93; 1.07) | 0.91 (0.79; 1.05) | O+D | . | 0.93 (0.84; 1.03) | . | . | . | . |
| 0.93 (0.83; 1.03) | 1.07 (0.90; 1.26) | 0.98 (0.91; 1.05) | 1.07 (0.91; 1.26) | P | . | . | . | . | . |
| 0.80 (0.67; 0.97) | 0.93 (0.82; 1.05) | 0.85 (0.71; 1.01) | 0.93 (0.84; 1.03) | 0.87 (0.72; 1.05) | P+D | . | . | . | . |
| 1.15 (0.84; 1.58) | 1.33 (1.01; 1.74) | 1.21 (0.89; 1.66) | 1.33 (1.01; 1.76) | 1.24 (0.90; 1.71) | 1.43 (1.06; 1.93) | R | 0.79 (0.65; 0.96) | . | . |
| 0.91 (0.71; 1.17) | 1.05 (0.87; 1.27) | 0.96 (0.75; 1.23) | 1.06 (0.86; 1.29) | 0.99 (0.76; 1.27) | 1.14 (0.90; 1.43) | 0.79 (0.65; 0.96) | R+D | . | . |
| 1.12 (0.90; 1.39) | 1.29 (0.99; 1.68) | 1.18 (0.95; 1.47) | 1.29 (1.00; 1.68) | 1.21 (0.96; 1.52) | 1.39 (1.05; 1.84) | 0.97 (0.67; 1.42) | 1.23 (0.88; 1.70) | T | 0.65 (0.53; 0.80) |
| 0.73 (0.54; 0.98) | 0.84 (0.60; 1.17) | 0.77 (0.57; 1.03) | 0.84 (0.60; 1.17) | 0.78 (0.58; 1.07) | 0.90 (0.64; 1.28) | 0.63 (0.41; 0.97) | 0.80 (0.54; 1.17) | 0.65 (0.53; 0.80) | T+D |

P-score

T+D 0.9056

P+D 0.8687

O+D 0.6766

G+D 0.6611

R+D 0.5192

P 0.5058

O 0.4077

G 0.2412

T 0.1126

R 0.1015

Delayed nausea

| Do | 1.99 (1.41; 2.80) | . | . | 0.91 (0.72; 1.16) | . | . | . | . | . | . | . |
| --- | --- | --- | --- | --- | --- | --- | --- | --- | --- | --- | --- |
| 1.99 (1.41; 2.80) | Do+D | . | . | . | . | . | . | . | . | . | . |
| 0.98 (0.67; 1.44) | 0.49 (0.30; 0.82) | G | 0.97 (0.76; 1.24) | 1.17 (0.73; 1.87) | . | . | . | . | . | . | . |
| 1.01 (0.71; 1.44) | 0.51 (0.31; 0.83) | 1.03 (0.83; 1.29) | G+D | . | 1.07 (0.88; 1.31) | . | 1.96 (1.40; 2.74) | . | 1.02 (0.70; 1.47) | . | . |
| 0.91 (0.72; 1.16) | 0.46 (0.30; 0.70) | 0.93 (0.69; 1.25) | 0.90 (0.69; 1.17) | O | 1.40 (1.09; 1.78) | 1.43 (1.09; 1.87) | . | . | . | 1.21 (0.89; 1.64) | . |
| 1.20 (0.86; 1.66) | 0.60 (0.37; 0.96) | 1.22 (0.94; 1.58) | 1.18 (1.00; 1.40) | 1.31 (1.05; 1.64) | O+D | . | 1.37 (1.15; 1.62) | . | . | . | . |
| 1.31 (0.91; 1.87) | 0.66 (0.40; 1.08) | 1.33 (0.89; 1.99) | 1.29 (0.89; 1.88) | 1.43 (1.09; 1.87) | 1.09 (0.77; 1.55) | P | . | . | . | . | . |
| 1.70 (1.19; 2.43) | 0.86 (0.52; 1.40) | 1.73 (1.30; 2.31) | 1.68 (1.37; 2.05) | 1.86 (1.42; 2.44) | 1.42 (1.22; 1.66) | 1.30 (0.89; 1.91) | P+D | . | . | . | . |
| 0.67 (0.38; 1.19) | 0.34 (0.17; 0.66) | 0.68 (0.41; 1.14) | 0.66 (0.42; 1.04) | 0.74 (0.43; 1.24) | 0.56 (0.35; 0.91) | 0.51 (0.28; 0.93) | 0.39 (0.24; 0.65) | R | 1.53 (1.18; 1.99) | . | . |
| 1.03 (0.62; 1.72) | 0.52 (0.28; 0.96) | 1.05 (0.68; 1.62) | 1.02 (0.70; 1.47) | 1.13 (0.71; 1.78) | 0.86 (0.57; 1.30) | 0.79 (0.46; 1.34) | 0.60 (0.40; 0.92) | 1.53 (1.18; 1.99) | R+D | . | . |
| 1.10 (0.75; 1.63) | 0.55 (0.33; 0.93) | 1.12 (0.73; 1.73) | 1.09 (0.73; 1.63) | 1.21 (0.89; 1.64) | 0.92 (0.63; 1.35) | 0.84 (0.56; 1.27) | 0.65 (0.43; 0.98) | 1.64 (0.89; 3.02) | 1.07 (0.62; 1.86) | T | 1.49 (1.11; 1.98) |
| 1.64 (1.01; 2.66) | 0.82 (0.46; 1.49) | 1.67 (0.99; 2.80) | 1.62 (0.98; 2.66) | 1.79 (1.18; 2.74) | 1.37 (0.85; 2.21) | 1.25 (0.76; 2.07) | 0.96 (0.58; 1.59) | 2.44 (1.24; 4.79) | 1.59 (0.86; 2.96) | 1.49 (1.11; 1.98) | T+D |

P-score

Do+D 0.9425

P+D 0.8825

T+D 0.8412

P 0.6688

O+D 0.5992

T 0.4595

R+D 0.3893

G+D 0.3498

Do 0.3368

G 0.3052

O 0.1881

R 0.0370

Delayed vomiting;

| Do | 1.99 (1.41; 2.80) | . | . | 0.91 (0.72; 1.16) | . | . | . | . | . | . | . |
| --- | --- | --- | --- | --- | --- | --- | --- | --- | --- | --- | --- |
| 1.99 (1.41; 2.80) | Do+D | . | . | . | . | . | . | . | . | . | . |
| 0.98 (0.67; 1.44) | 0.49 (0.30; 0.82) | G | 0.97 (0.76; 1.24) | 1.17 (0.73; 1.87) | . | . | . | . | . | . | . |
| 1.01 (0.71; 1.44) | 0.51 (0.31; 0.83) | 1.03 (0.83; 1.29) | G+D | . | 1.07 (0.88; 1.31) | . | 1.96 (1.40; 2.74) | . | 1.02 (0.70; 1.47) | . | . |
| 0.91 (0.72; 1.16) | 0.46 (0.30; 0.70) | 0.93 (0.69; 1.25) | 0.90 (0.69; 1.17) | O | 1.40 (1.09; 1.78) | 1.43 (1.09; 1.87) | . | . | . | 1.21 (0.89; 1.64) | . |
| 1.20 (0.86; 1.66) | 0.60 (0.37; 0.96) | 1.22 (0.94; 1.58) | 1.18 (1.00; 1.40) | 1.31 (1.05; 1.64) | O+D | . | 1.37 (1.15; 1.62) | . | . | . | . |
| 1.31 (0.91; 1.87) | 0.66 (0.40; 1.08) | 1.33 (0.89; 1.99) | 1.29 (0.89; 1.88) | 1.43 (1.09; 1.87) | 1.09 (0.77; 1.55) | P | . | . | . | . | . |
| 1.70 (1.19; 2.43) | 0.86 (0.52; 1.40) | 1.73 (1.30; 2.31) | 1.68 (1.37; 2.05) | 1.86 (1.42; 2.44) | 1.42 (1.22; 1.66) | 1.30 (0.89; 1.91) | P+D | . | . | . | . |
| 0.67 (0.38; 1.19) | 0.34 (0.17; 0.66) | 0.68 (0.41; 1.14) | 0.66 (0.42; 1.04) | 0.74 (0.43; 1.24) | 0.56 (0.35; 0.91) | 0.51 (0.28; 0.93) | 0.39 (0.24; 0.65) | R | 1.53 (1.18; 1.99) | . | . |
| 1.03 (0.62; 1.72) | 0.52 (0.28; 0.96) | 1.05 (0.68; 1.62) | 1.02 (0.70; 1.47) | 1.13 (0.71; 1.78) | 0.86 (0.57; 1.30) | 0.79 (0.46; 1.34) | 0.60 (0.40; 0.92) | 1.53 (1.18; 1.99) | R+D | . | . |
| 1.10 (0.75; 1.63) | 0.55 (0.33; 0.93) | 1.12 (0.73; 1.73) | 1.09 (0.73; 1.63) | 1.21 (0.89; 1.64) | 0.92 (0.63; 1.35) | 0.84 (0.56; 1.27) | 0.65 (0.43; 0.98) | 1.64 (0.89; 3.02) | 1.07 (0.62; 1.86) | T | 1.49 (1.11; 1.98) |
| 1.64 (1.01; 2.66) | 0.82 (0.46; 1.49) | 1.67 (0.99; 2.80) | 1.62 (0.98; 2.66) | 1.79 (1.18; 2.74) | 1.37 (0.85; 2.21) | 1.25 (0.76; 2.07) | 0.96 (0.58; 1.59) | 2.44 (1.24; 4.79) | 1.59 (0.86; 2.96) | 1.49 (1.11; 1.98) | T+D |

P-score

Do+D 0.9425

P+D 0.8825

T+D 0.8412

P 0.6688

O+D 0.5992

T 0.4595

R+D 0.3893

G+D 0.3498

Do 0.3368

G 0.3052

O 0.1881

R 0.0370

Delayed complete control

| G | 1.12 (0.75; 1.66) | . | . | . | . | . |
| --- | --- | --- | --- | --- | --- | --- |
| 1.12 (0.75; 1.66) | G+D | . | 0.94 (0.76; 1.16) | . | . | 0.92 (0.58; 1.46) |
| 1.71 (1.01; 2.88) | 1.53 (1.08; 2.16) | O | 0.61 (0.47; 0.81) | 0.70 (0.50; 0.98) | . | . |
| 1.05 (0.67; 1.64) | 0.94 (0.76; 1.16) | 0.61 (0.47; 0.81) | O+D | . | 0.70 (0.57; 0.87) | . |
| 1.19 (0.64; 2.23) | 1.07 (0.66; 1.73) | 0.70 (0.50; 0.98) | 1.14 (0.73; 1.76) | P | . | . |
| 0.74 (0.45; 1.21) | 0.66 (0.49; 0.89) | 0.43 (0.31; 0.61) | 0.70 (0.57; 0.87) | 0.62 (0.38; 1.01) | P+D | . |
| 1.03 (0.56; 1.89) | 0.92 (0.58; 1.46) | 0.60 (0.34; 1.07) | 0.98 (0.59; 1.63) | 0.87 (0.44; 1.69) | 1.40 (0.81; 2.42) | R+D |

P-score

P+D 0.9569

G 0.6049

R+D 0.5589

O+D 0.5554

G+D 0.4235

P 0.3848

O 0.0154

32、The outcomes after the article of “Sorbe B 1994” was deleted.

Acute vomiting

| Do | 0.96 (0.79; 1.15) | . | 0.99 (0.85; 1.15) | . | . | . | . | . | . | . |
| --- | --- | --- | --- | --- | --- | --- | --- | --- | --- | --- |
| 0.93 (0.82; 1.06) | G | 1.57 (1.28; 1.91) | 1.09 (0.99; 1.19) | . | 1.61 (0.94; 2.77) | . | 1.32 (0.93; 1.87) | . | 0.91 (0.65; 1.27) | . |
| 1.46 (1.18; 1.80) | 1.57 (1.32; 1.87) | G+D | . | 1.06 (0.88; 1.28) | . | 1.05 (0.81; 1.36) | . | 0.84 (0.48; 1.46) | . | . |
| 1.01 (0.89; 1.14) | 1.08 (1.00; 1.18) | 0.69 (0.57; 0.83) | O | 1.28 (0.88; 1.85) | 1.98 (0.60; 6.53) | . | 1.06 (0.74; 1.52) | . | 0.78 (0.60; 1.03) | . |
| 1.41 (1.11; 1.80) | 1.52 (1.23; 1.88) | 0.97 (0.83; 1.13) | 1.40 (1.13; 1.74) | O+D | . | 1.32 (1.08; 1.60) | . | . | . | . |
| 1.57 (0.95; 2.61) | 1.69 (1.03; 2.77) | 1.08 (0.64; 1.82) | 1.56 (0.95; 2.57) | 1.11 (0.65; 1.90) | P | . | . | . | . | . |
| 1.73 (1.33; 2.27) | 1.87 (1.47; 2.37) | 1.19 (0.99; 1.43) | 1.72 (1.35; 2.21) | 1.23 (1.04; 1.45) | 1.10 (0.64; 1.91) | P+D | . | . | . | . |
| 1.10 (0.84; 1.43) | 1.18 (0.93; 1.50) | 0.75 (0.57; 1.00) | 1.09 (0.86; 1.39) | 0.78 (0.57; 1.05) | 0.70 (0.40; 1.20) | 0.63 (0.46; 0.88) | R | 1.58 (1.06; 2.38) | . | . |
| 1.54 (1.04; 2.27) | 1.65 (1.14; 2.40) | 1.05 (0.72; 1.54) | 1.53 (1.05; 2.22) | 1.09 (0.73; 1.62) | 0.98 (0.53; 1.81) | 0.89 (0.59; 1.34) | 1.40 (1.00; 1.98) | R+D | . | . |
| 0.79 (0.61; 1.03) | 0.85 (0.67; 1.08) | 0.54 (0.41; 0.73) | 0.79 (0.62; 1.00) | 0.56 (0.41; 0.77) | 0.50 (0.29; 0.87) | 0.46 (0.33; 0.64) | 0.72 (0.52; 1.01) | 0.52 (0.33; 0.80) | T | 1.89 (1.29; 2.77) |
| 1.50 (0.94; 2.38) | 1.61 (1.03; 2.53) | 1.03 (0.64; 1.66) | 1.49 (0.95; 2.33) | 1.06 (0.65; 1.74) | 0.95 (0.49; 1.86) | 0.86 (0.52; 1.44) | 1.37 (0.82; 2.27) | 0.98 (0.55; 1.74) | 1.89 (1.29; 2.77) | T+D |

P-score

P+D 0.9024

P 0.7510

R+D 0.7499

T+D 0.7118

G+D 0.6893

O+D 0.6396

R 0.3706

O 0.2822

Do 0.2638

G 0.1193

T 0.0202

Delayed vomiting

| Do | 2.33 (1.53; 3.56) | . | . | 1.27 (0.79; 2.04) | . | . | . | . | . | . | . |
| --- | --- | --- | --- | --- | --- | --- | --- | --- | --- | --- | --- |
| 2.33 (1.53; 3.56) | Do+D | . | . | . | . | . | . | . | . | . | . |
| 1.32 (0.77; 2.29) | 0.57 (0.28; 1.13) | G | 0.93 (0.61; 1.41) | 1.09 (0.76; 1.56) | . | 1.12 (0.72; 1.73) | . | . | . | 1.14 (0.66; 1.98) | . |
| 1.25 (0.69; 2.26) | 0.54 (0.26; 1.11) | 0.94 (0.67; 1.33) | G+D | . | 0.99 (0.69; 1.43) | . | 3.52 (1.94; 6.38) | . | 0.97 (0.54; 1.74) | . | . |
| 1.27 (0.79; 2.04) | 0.54 (0.29; 1.03) | 0.96 (0.73; 1.26) | 1.01 (0.71; 1.45) | O | 1.28 (0.89; 1.86) | 1.87 (1.16; 3.03) | . | . | . | 0.98 (0.71; 1.35) | . |
| 1.61 (0.91; 2.85) | 0.69 (0.34; 1.40) | 1.22 (0.85; 1.73) | 1.29 (0.97; 1.71) | 1.27 (0.93; 1.74) | O+D | . | 1.33 (1.10; 1.61) | . | . | . | . |
| 1.84 (1.01; 3.33) | 0.79 (0.38; 1.63) | 1.39 (0.98; 1.96) | 1.47 (0.93; 2.31) | 1.45 (1.01; 2.07) | 1.14 (0.73; 1.78) | P | . | . | . | . | . |
| 2.29 (1.26; 4.14) | 0.98 (0.47; 2.03) | 1.73 (1.17; 2.54) | 1.83 (1.33; 2.50) | 1.80 (1.26; 2.57) | 1.42 (1.18; 1.71) | 1.25 (0.77; 2.00) | P+D | . | . | . | . |
| 0.75 (0.30; 1.88) | 0.32 (0.12; 0.89) | 0.57 (0.26; 1.24) | 0.60 (0.29; 1.21) | 0.59 (0.27; 1.30) | 0.46 (0.22; 1.00) | 0.41 (0.18; 0.95) | 0.33 (0.15; 0.71) | R | 1.63 (1.09; 2.43) | . | . |
| 1.22 (0.53; 2.79) | 0.52 (0.21; 1.33) | 0.92 (0.47; 1.80) | 0.97 (0.54; 1.74) | 0.96 (0.49; 1.90) | 0.76 (0.40; 1.44) | 0.66 (0.32; 1.39) | 0.53 (0.27; 1.03) | 1.63 (1.09; 2.43) | R+D | . | . |
| 1.29 (0.73; 2.27) | 0.55 (0.27; 1.12) | 0.97 (0.67; 1.41) | 1.03 (0.66; 1.62) | 1.02 (0.75; 1.38) | 0.80 (0.52; 1.23) | 0.70 (0.45; 1.11) | 0.56 (0.36; 0.89) | 1.72 (0.74; 3.99) | 1.06 (0.51; 2.21) | T | 1.52 (1.02; 2.26) |
| 1.96 (0.98; 3.92) | 0.84 (0.37; 1.89) | 1.48 (0.86; 2.55) | 1.57 (0.86; 2.86) | 1.54 (0.93; 2.55) | 1.22 (0.68; 2.18) | 1.07 (0.58; 1.95) | 0.86 (0.47; 1.58) | 2.62 (1.04; 6.63) | 1.61 (0.70; 3.72) | 1.52 (1.02; 2.26) | T+D |

P-score

P+D 0.9033

Do+D 0.8628

T+D 0.7802

P 0.7481

O+D 0.6375

G 0.4069

T 0.3724

R+D 0.3648

O 0.3481

G+D 0.3387

Do 0.1726

R 0.0646

33、The outcomes after the article of “Spector JI 1998” was deleted.

Acute nausea

| A+D | . | . | 1.16 (0.67; 2.00) | . | . | . | . | . | . | . | . |
| --- | --- | --- | --- | --- | --- | --- | --- | --- | --- | --- | --- |
| 0.89 (0.49; 1.61) | Do | 0.98 (0.85; 1.12) | . | 0.99 (0.68; 1.42) | . | . | . | . | . | . | . |
| 0.88 (0.49; 1.56) | 0.98 (0.86; 1.12) | G | 1.32 (1.07; 1.63) | 0.98 (0.91; 1.06) | . | . | . | 1.32 (0.97; 1.81) | . | 0.41 (0.23; 0.73) | . |
| 1.16 (0.67; 2.00) | 1.30 (1.04; 1.64) | 1.33 (1.10; 1.60) | G+D | . | 1.04 (0.91; 1.19) | . | 0.97 (0.84; 1.12) | . | 1.22 (0.66; 2.23) | . | . |
| 0.85 (0.48; 1.52) | 0.96 (0.83; 1.11) | 0.98 (0.91; 1.05) | 0.74 (0.60; 0.90) | O | 1.26 (0.74; 2.12) | 1.33 (0.80; 2.20) | . | 1.16 (0.72; 1.85) | . | 1.10 (0.78; 1.56) | . |
| 1.15 (0.66; 2.01) | 1.29 (1.01; 1.66) | 1.32 (1.06; 1.63) | 0.99 (0.89; 1.11) | 1.35 (1.08; 1.68) | O+D | . | 1.14 (0.93; 1.38) | . | . | . | . |
| 1.14 (0.53; 2.45) | 1.28 (0.76; 2.16) | 1.30 (0.78; 2.16) | 0.98 (0.57; 1.69) | 1.33 (0.80; 2.20) | 0.99 (0.57; 1.71) | P | . | . | . | . | . |
| 1.19 (0.68; 2.07) | 1.33 (1.03; 1.72) | 1.36 (1.09; 1.69) | 1.02 (0.91; 1.15) | 1.39 (1.10; 1.75) | 1.03 (0.90; 1.18) | 1.04 (0.60; 1.82) | P+D | . | . | . | . |
| 1.07 (0.58; 1.99) | 1.20 (0.91; 1.59) | 1.22 (0.96; 1.56) | 0.92 (0.69; 1.24) | 1.25 (0.97; 1.61) | 0.93 (0.68; 1.27) | 0.94 (0.54; 1.65) | 0.90 (0.66; 1.24) | R | 1.61 (1.16; 2.24) | . | . |
| 1.65 (0.86; 3.18) | 1.85 (1.27; 2.69) | 1.88 (1.33; 2.68) | 1.42 (0.98; 2.05) | 1.93 (1.35; 2.75) | 1.43 (0.98; 2.10) | 1.45 (0.78; 2.68) | 1.39 (0.94; 2.04) | 1.54 (1.15; 2.07) | R+D | . | . |
| 0.73 (0.38; 1.40) | 0.82 (0.59; 1.14) | 0.84 (0.62; 1.13) | 0.63 (0.44; 0.90) | 0.86 (0.64; 1.15) | 0.64 (0.44; 0.92) | 0.64 (0.36; 1.15) | 0.62 (0.42; 0.90) | 0.68 (0.46; 1.01) | 0.44 (0.28; 0.70) | T | 1.85 (1.26; 2.74) |
| 1.36 (0.64; 2.89) | 1.52 (0.92; 2.53) | 1.55 (0.95; 2.54) | 1.17 (0.69; 1.98) | 1.59 (0.97; 2.59) | 1.18 (0.69; 2.01) | 1.19 (0.59; 2.41) | 1.14 (0.67; 1.96) | 1.27 (0.73; 2.19) | 0.82 (0.45; 1.50) | 1.85 (1.26; 2.74) | T+D |

P-score

R+D 0.9486

T+D 0.7770

P+D 0.6969

G+D 0.6485

O+D 0.6314

P 0.5910

R 0.5331

A+D 0.4360

Do 0.2666

G 0.2366

O 0.1723

T 0.0621

Acute vomiting

| Do | 0.96 (0.79; 1.15) | . | 0.99 (0.84; 1.15) | . | . | . | . | . | . | . |
| --- | --- | --- | --- | --- | --- | --- | --- | --- | --- | --- |
| 0.93 (0.82; 1.06) | G | 1.57 (1.28; 1.91) | 1.08 (0.97; 1.19) | . | 1.61 (0.94; 2.77) | . | 1.32 (0.93; 1.87) | . | 0.91 (0.65; 1.27) | . |
| 1.46 (1.18; 1.81) | 1.57 (1.31; 1.87) | G+D | . | 1.06 (0.88; 1.28) | . | 1.05 (0.81; 1.36) | . | 0.84 (0.48; 1.47) | . | . |
| 1.00 (0.89; 1.14) | 1.07 (0.98; 1.18) | 0.69 (0.57; 0.83) | O | 1.28 (0.88; 1.85) | 1.98 (0.60; 6.53) | . | 1.06 (0.74; 1.52) | . | 0.78 (0.60; 1.03) | . |
| 1.42 (1.11; 1.80) | 1.52 (1.23; 1.87) | 0.97 (0.83; 1.13) | 1.41 (1.14; 1.75) | O+D | . | 1.32 (1.08; 1.60) | . | . | . | . |
| 1.58 (0.95; 2.63) | 1.69 (1.03; 2.77) | 1.08 (0.64; 1.82) | 1.57 (0.95; 2.59) | 1.11 (0.65; 1.90) | P | . | . | . | . | . |
| 1.74 (1.33; 2.28) | 1.86 (1.46; 2.37) | 1.19 (0.99; 1.43) | 1.73 (1.35; 2.22) | 1.23 (1.04; 1.45) | 1.10 (0.64; 1.91) | P+D | . | . | . | . |
| 1.10 (0.84; 1.43) | 1.18 (0.92; 1.49) | 0.75 (0.57; 1.00) | 1.09 (0.86; 1.39) | 0.77 (0.57; 1.05) | 0.70 (0.40; 1.20) | 0.63 (0.45; 0.88) | R | 1.58 (1.05; 2.38) | . | . |
| 1.54 (1.04; 2.27) | 1.65 (1.14; 2.39) | 1.05 (0.72; 1.53) | 1.53 (1.06; 2.23) | 1.09 (0.73; 1.62) | 0.98 (0.53; 1.81) | 0.89 (0.59; 1.34) | 1.40 (1.00; 1.98) | R+D | . | . |
| 0.79 (0.61; 1.03) | 0.85 (0.67; 1.08) | 0.54 (0.40; 0.73) | 0.79 (0.62; 1.00) | 0.56 (0.41; 0.77) | 0.50 (0.29; 0.87) | 0.46 (0.33; 0.64) | 0.72 (0.52; 1.01) | 0.52 (0.33; 0.80) | T | 1.99 (1.42; 2.79) |
| 1.58 (1.03; 2.42) | 1.69 (1.12; 2.56) | 1.08 (0.69; 1.69) | 1.58 (1.04; 2.38) | 1.12 (0.70; 1.77) | 1.00 (0.53; 1.91) | 0.91 (0.56; 1.46) | 1.44 (0.90; 2.32) | 1.03 (0.59; 1.79) | 1.99 (1.42; 2.79) | T+D |

P-score

P+D 0.8961

T+D 0.7599

P 0.7461

R+D 0.7419

G+D 0.6814

O+D 0.6302

R 0.3662

O 0.2739

Do 0.2608

G 0.1237

T 0.0197

Acute complete control

| G | 0.80 (0.61; 1.04) | 0.97 (0.90; 1.06) | . | . | . | . | . | 1.16 (0.92; 1.47) | . |
| --- | --- | --- | --- | --- | --- | --- | --- | --- | --- |
| 0.87 (0.74; 1.02) | G+D | . | 0.99 (0.92; 1.06) | . | 0.98 (0.91; 1.05) | . | 1.05 (0.87; 1.27) | . | . |
| 0.97 (0.89; 1.04) | 1.11 (0.95; 1.29) | O | 0.94 (0.80; 1.12) | 0.98 (0.91; 1.05) | . | . | . | 1.13 (0.89; 1.44) | . |
| 0.88 (0.75; 1.03) | 1.01 (0.95; 1.07) | 0.91 (0.79; 1.05) | O+D | . | 0.93 (0.84; 1.03) | . | . | . | . |
| 0.94 (0.85; 1.05) | 1.08 (0.91; 1.28) | 0.98 (0.91; 1.05) | 1.07 (0.91; 1.26) | P | . | . | . | . | . |
| 0.84 (0.71; 0.99) | 0.96 (0.91; 1.03) | 0.87 (0.74; 1.02) | 0.96 (0.89; 1.03) | 0.89 (0.75; 1.06) | P+D | . | . | . | . |
| 1.15 (0.84; 1.58) | 1.33 (1.01; 1.74) | 1.20 (0.88; 1.63) | 1.31 (1.00; 1.74) | 1.23 (0.89; 1.69) | 1.37 (1.04; 1.82) | R | 0.79 (0.65; 0.96) | . | . |
| 0.92 (0.71; 1.17) | 1.05 (0.87; 1.27) | 0.95 (0.74; 1.21) | 1.04 (0.85; 1.27) | 0.97 (0.75; 1.26) | 1.09 (0.89; 1.33) | 0.79 (0.65; 0.96) | R+D | . | . |
| 1.13 (0.91; 1.40) | 1.30 (1.00; 1.69) | 1.17 (0.94; 1.46) | 1.28 (0.99; 1.67) | 1.20 (0.95; 1.51) | 1.34 (1.03; 1.75) | 0.98 (0.67; 1.43) | 1.23 (0.89; 1.71) | T | 0.65 (0.53; 0.80) |
| 0.73 (0.54; 0.99) | 0.84 (0.60; 1.18) | 0.76 (0.56; 1.03) | 0.83 (0.60; 1.16) | 0.78 (0.57; 1.06) | 0.87 (0.62; 1.22) | 0.63 (0.41; 0.98) | 0.80 (0.54; 1.18) | 0.65 (0.53; 0.80) | T+D |

P-score

T+D 0.9139

P+D 0.8429

G+D 0.6919

O+D 0.6531

R+D 0.5415

P 0.4884

O 0.3852

G 0.2644

T 0.1118

R 0.1068

34、The outcomes after the article of “Tan J 2017” was deleted.

Acute nausea

| A+D | . | . | 1.16 (0.67; 2.00) | . | . | . | . | . | . | . | . |
| --- | --- | --- | --- | --- | --- | --- | --- | --- | --- | --- | --- |
| 0.90 (0.50; 1.61) | Do | 0.98 (0.85; 1.12) | . | 0.99 (0.68; 1.42) | . | . | . | . | . | . | . |
| 0.88 (0.49; 1.56) | 0.98 (0.86; 1.11) | G | 1.32 (1.07; 1.63) | 1.00 (0.93; 1.07) | . | . | . | 1.32 (0.97; 1.81) | . | 0.41 (0.23; 0.73) | . |
| 1.16 (0.67; 2.00) | 1.30 (1.03; 1.63) | 1.32 (1.09; 1.60) | G+D | . | 1.04 (0.91; 1.19) | . | 0.97 (0.84; 1.12) | . | 1.22 (0.66; 2.23) | . | . |
| 0.87 (0.49; 1.55) | 0.97 (0.85; 1.12) | 0.99 (0.93; 1.06) | 0.75 (0.62; 0.92) | O | 1.26 (0.74; 2.12) | 1.33 (0.80; 2.20) | . | 1.16 (0.72; 1.85) | . | 1.10 (0.78; 1.56) | . |
| 1.20 (0.69; 2.10) | 1.34 (1.04; 1.73) | 1.37 (1.10; 1.71) | 1.04 (0.91; 1.18) | 1.38 (1.10; 1.73) | O+D | . | . | . | . | . | . |
| 1.16 (0.54; 2.50) | 1.30 (0.77; 2.19) | 1.32 (0.80; 2.20) | 1.00 (0.58; 1.72) | 1.33 (0.80; 2.20) | 0.97 (0.56; 1.68) | P | . | . | . | . | . |
| 1.13 (0.64; 1.98) | 1.26 (0.96; 1.65) | 1.28 (1.01; 1.63) | 0.97 (0.84; 1.12) | 1.29 (1.01; 1.65) | 0.94 (0.78; 1.13) | 0.97 (0.55; 1.70) | P+D | . | . | . | . |
| 1.08 (0.58; 2.00) | 1.20 (0.91; 1.59) | 1.23 (0.96; 1.57) | 0.93 (0.69; 1.25) | 1.24 (0.96; 1.59) | 0.90 (0.65; 1.23) | 0.93 (0.53; 1.63) | 0.96 (0.69; 1.33) | R | 1.61 (1.16; 2.24) | . | . |
| 1.66 (0.86; 3.19) | 1.85 (1.28; 2.69) | 1.89 (1.33; 2.68) | 1.43 (0.99; 2.06) | 1.90 (1.33; 2.71) | 1.38 (0.94; 2.03) | 1.43 (0.77; 2.64) | 1.47 (0.99; 2.18) | 1.54 (1.14; 2.07) | R+D | . | . |
| 0.74 (0.39; 1.42) | 0.83 (0.60; 1.15) | 0.85 (0.63; 1.14) | 0.64 (0.45; 0.91) | 0.85 (0.63; 1.15) | 0.62 (0.43; 0.90) | 0.64 (0.36; 1.15) | 0.66 (0.45; 0.97) | 0.69 (0.47; 1.01) | 0.45 (0.28; 0.71) | T | 1.85 (1.26; 2.74) |
| 1.38 (0.65; 2.94) | 1.54 (0.93; 2.56) | 1.57 (0.96; 2.57) | 1.19 (0.70; 2.01) | 1.58 (0.97; 2.58) | 1.15 (0.67; 1.96) | 1.19 (0.59; 2.40) | 1.22 (0.71; 2.11) | 1.28 (0.74; 2.21) | 0.83 (0.46; 1.52) | 1.85 (1.26; 2.74) | T+D |

P-score

R+D 0.9473

T+D 0.7859

O+D 0.7118

G+D 0.6461

P 0.6076

P+D 0.5892

R 0.5381

A+D 0.4294

Do 0.2606

G 0.2176

O 0.2000

T 0.0663

Acute vomiting

| Do | 0.96 (0.79; 1.15) | . | 0.99 (0.84; 1.15) | . | . | . | . | . | . | . |
| --- | --- | --- | --- | --- | --- | --- | --- | --- | --- | --- |
| 0.93 (0.82; 1.06) | G | 1.57 (1.28; 1.91) | 1.09 (0.99; 1.19) | . | 1.61 (0.94; 2.77) | . | 1.32 (0.93; 1.87) | . | 0.91 (0.65; 1.27) | . |
| 1.46 (1.18; 1.81) | 1.57 (1.32; 1.88) | G+D | . | 1.06 (0.88; 1.28) | . | 1.05 (0.81; 1.36) | . | 0.84 (0.48; 1.47) | . | . |
| 1.01 (0.89; 1.14) | 1.08 (0.99; 1.18) | 0.69 (0.57; 0.83) | O | 1.28 (0.88; 1.85) | 1.98 (0.60; 6.53) | . | 1.06 (0.74; 1.52) | . | 0.78 (0.60; 1.03) | . |
| 1.39 (1.09; 1.78) | 1.50 (1.21; 1.85) | 0.95 (0.82; 1.11) | 1.39 (1.11; 1.72) | O+D | . | 1.53 (1.18; 1.97) | . | . | . | . |
| 1.57 (0.95; 2.62) | 1.69 (1.03; 2.77) | 1.08 (0.64; 1.81) | 1.56 (0.95; 2.57) | 1.13 (0.66; 1.93) | P | . | . | . | . | . |
| 1.82 (1.37; 2.41) | 1.95 (1.51; 2.52) | 1.24 (1.02; 1.51) | 1.80 (1.39; 2.34) | 1.30 (1.07; 1.59) | 1.15 (0.66; 2.01) | P+D | . | . | . | . |
| 1.10 (0.84; 1.43) | 1.18 (0.93; 1.50) | 0.75 (0.56; 0.99) | 1.09 (0.86; 1.39) | 0.79 (0.58; 1.07) | 0.70 (0.40; 1.21) | 0.60 (0.43; 0.85) | R | 1.58 (1.05; 2.38) | . | . |
| 1.54 (1.04; 2.27) | 1.66 (1.14; 2.40) | 1.05 (0.72; 1.53) | 1.53 (1.05; 2.22) | 1.10 (0.74; 1.65) | 0.98 (0.53; 1.81) | 0.85 (0.56; 1.29) | 1.40 (1.00; 1.98) | R+D | . | . |
| 0.79 (0.61; 1.03) | 0.85 (0.67; 1.08) | 0.54 (0.40; 0.73) | 0.79 (0.62; 1.00) | 0.57 (0.42; 0.78) | 0.50 (0.29; 0.87) | 0.44 (0.31; 0.62) | 0.72 (0.52; 1.01) | 0.52 (0.33; 0.80) | T | 1.99 (1.42; 2.79) |
| 1.58 (1.03; 2.43) | 1.70 (1.12; 2.57) | 1.08 (0.69; 1.69) | 1.57 (1.04; 2.37) | 1.13 (0.71; 1.80) | 1.01 (0.53; 1.91) | 0.87 (0.54; 1.41) | 1.44 (0.90; 2.32) | 1.03 (0.59; 1.79) | 1.99 (1.42; 2.79) | T+D |

P-score

P+D 0.9158

T+D 0.7565

P 0.7403

R+D 0.7386

G+D 0.6881

O+D 0.6146

R 0.3668

O 0.2794

Do 0.2609

G 0.1188

T 0.0202

Acute complete control

| G | 0.80 (0.61; 1.04) | 0.96 (0.89; 1.03) | . | . | . | . | . | 1.16 (0.92; 1.47) | . |
| --- | --- | --- | --- | --- | --- | --- | --- | --- | --- |
| 0.87 (0.74; 1.02) | G+D | . | 0.99 (0.92; 1.06) | . | 0.98 (0.91; 1.05) | . | 1.05 (0.87; 1.27) | . | . |
| 0.95 (0.88; 1.02) | 1.09 (0.94; 1.27) | O | 0.94 (0.80; 1.12) | 0.98 (0.91; 1.05) | . | . | . | 1.13 (0.89; 1.44) | . |
| 0.87 (0.74; 1.01) | 1.00 (0.93; 1.07) | 0.91 (0.79; 1.05) | O+D | . | . | . | . | . | . |
| 0.93 (0.83; 1.03) | 1.07 (0.90; 1.26) | 0.98 (0.91; 1.05) | 1.07 (0.91; 1.26) | P | . | . | . | . | . |
| 0.85 (0.71; 1.01) | 0.98 (0.91; 1.05) | 0.89 (0.76; 1.06) | 0.98 (0.89; 1.08) | 0.92 (0.76; 1.10) | P+D | . | . | . | . |
| 1.15 (0.84; 1.58) | 1.33 (1.01; 1.74) | 1.21 (0.89; 1.66) | 1.33 (1.01; 1.76) | 1.24 (0.90; 1.71) | 1.36 (1.02; 1.80) | R | 0.79 (0.65; 0.96) | . | . |
| 0.91 (0.71; 1.17) | 1.05 (0.87; 1.27) | 0.96 (0.75; 1.23) | 1.06 (0.86; 1.29) | 0.99 (0.76; 1.27) | 1.07 (0.88; 1.32) | 0.79 (0.65; 0.96) | R+D | . | . |
| 1.12 (0.90; 1.39) | 1.29 (0.99; 1.68) | 1.18 (0.95; 1.47) | 1.29 (1.00; 1.68) | 1.21 (0.96; 1.52) | 1.32 (1.00; 1.73) | 0.97 (0.67; 1.42) | 1.23 (0.88; 1.70) | T | 0.65 (0.53; 0.80) |
| 0.73 (0.54; 0.98) | 0.84 (0.60; 1.17) | 0.77 (0.57; 1.03) | 0.84 (0.60; 1.17) | 0.78 (0.58; 1.07) | 0.86 (0.61; 1.21) | 0.63 (0.41; 0.97) | 0.80 (0.54; 1.17) | 0.65 (0.53; 0.80) | T+D |

P-score

T+D 0.9165

P+D 0.7719

O+D 0.7081

G+D 0.6796

R+D 0.5313

P 0.5174

O 0.4149

G 0.2438

T 0.1142

R 0.1023

Delayed nausea

| Do | 1.99 (1.16; 3.41) | . | . | 0.91 (0.57; 1.47) | . | . | . | . | . | . | . |
| --- | --- | --- | --- | --- | --- | --- | --- | --- | --- | --- | --- |
| 1.99 (1.16; 3.41) | Do+D | . | . | . | . | . | . | . | . | . | . |
| 0.95 (0.48; 1.88) | 0.48 (0.20; 1.14) | G | 0.97 (0.60; 1.57) | 1.17 (0.63; 2.20) | . | . | . | . | . | . | . |
| 1.03 (0.52; 2.04) | 0.52 (0.22; 1.23) | 1.09 (0.71; 1.66) | G+D | . | 1.07 (0.68; 1.70) | . | 1.45 (1.03; 2.05) | . | 1.02 (0.58; 1.78) | . | . |
| 0.91 (0.57; 1.47) | 0.46 (0.22; 0.94) | 0.96 (0.59; 1.57) | 0.89 (0.55; 1.44) | O | 1.45 (0.98; 2.17) | 1.42 (0.98; 2.05) | . | . | . | 1.21 (0.72; 2.03) | . |
| 1.23 (0.67; 2.24) | 0.62 (0.28; 1.39) | 1.30 (0.79; 2.13) | 1.19 (0.79; 1.79) | 1.34 (0.93; 1.94) | O+D | . | . | . | . | . | . |
| 1.29 (0.71; 2.37) | 0.65 (0.29; 1.46) | 1.37 (0.74; 2.52) | 1.26 (0.69; 2.30) | 1.42 (0.98; 2.05) | 1.05 (0.63; 1.77) | P | . | . | . | . | . |
| 1.49 (0.70; 3.20) | 0.75 (0.30; 1.91) | 1.58 (0.92; 2.72) | 1.45 (1.03; 2.05) | 1.64 (0.90; 2.96) | 1.22 (0.71; 2.08) | 1.15 (0.57; 2.32) | P+D | . | . | . | . |
| 0.68 (0.25; 1.87) | 0.34 (0.11; 1.08) | 0.72 (0.31; 1.70) | 0.66 (0.31; 1.40) | 0.75 (0.31; 1.82) | 0.56 (0.24; 1.30) | 0.53 (0.20; 1.38) | 0.46 (0.20; 1.04) | R | 1.53 (0.94; 2.51) | . | . |
| 1.05 (0.43; 2.53) | 0.53 (0.19; 1.48) | 1.11 (0.55; 2.23) | 1.02 (0.58; 1.78) | 1.15 (0.55; 2.40) | 0.85 (0.43; 1.71) | 0.81 (0.35; 1.84) | 0.70 (0.36; 1.35) | 1.53 (0.94; 2.51) | R+D | . | . |
| 1.10 (0.54; 2.23) | 0.55 (0.23; 1.35) | 1.16 (0.57; 2.38) | 1.07 (0.53; 2.17) | 1.21 (0.72; 2.03) | 0.90 (0.48; 1.69) | 0.85 (0.45; 1.61) | 0.74 (0.34; 1.62) | 1.62 (0.58; 4.52) | 1.05 (0.43; 2.60) | T | 1.49 (0.89; 2.47) |
| 1.64 (0.69; 3.91) | 0.82 (0.30; 2.29) | 1.73 (0.72; 4.15) | 1.59 (0.66; 3.80) | 1.79 (0.87; 3.71) | 1.33 (0.59; 3.01) | 1.26 (0.56; 2.85) | 1.10 (0.43; 2.80) | 2.40 (0.76; 7.56) | 1.57 (0.56; 4.41) | 1.49 (0.89; 2.47) | T+D |

P-score

Do+D 0.8836

T+D 0.7841

P+D 0.7682

P 0.6389

O+D 0.5976

T 0.4575

R+D 0.4372

G+D 0.3918

Do 0.3675

G 0.3051

O 0.2416

R 0.1269

Delayed vomiting

| Do | 2.33 (1.43; 3.81) | . | . | 1.27 (0.74; 2.17) | . | . | . | . | . | . | . |
| --- | --- | --- | --- | --- | --- | --- | --- | --- | --- | --- | --- |
| 2.33 (1.43; 3.81) | Do+D | . | . | . | . | . | . | . | . | . | . |
| 1.32 (0.71; 2.45) | 0.57 (0.26; 1.25) | G | 0.93 (0.57; 1.51) | 1.09 (0.73; 1.63) | . | 1.12 (0.67; 1.85) | . | . | . | 1.14 (0.63; 2.09) | . |
| 1.24 (0.63; 2.44) | 0.53 (0.23; 1.23) | 0.94 (0.64; 1.39) | G+D | . | 0.99 (0.64; 1.54) | . | 3.52 (1.85; 6.71) | . | 0.97 (0.52; 1.83) | . | . |
| 1.27 (0.74; 2.17) | 0.54 (0.26; 1.13) | 0.96 (0.71; 1.31) | 1.02 (0.68; 1.54) | O | 1.30 (0.86; 1.97) | 1.85 (1.11; 3.07) | . | . | . | 0.98 (0.67; 1.42) | . |
| 1.64 (0.86; 3.12) | 0.70 (0.31; 1.58) | 1.24 (0.83; 1.86) | 1.32 (0.94; 1.84) | 1.29 (0.90; 1.84) | O+D | . | 1.29 (0.94; 1.78) | . | . | . | . |
| 1.86 (0.96; 3.60) | 0.80 (0.35; 1.82) | 1.41 (0.95; 2.07) | 1.49 (0.89; 2.50) | 1.46 (0.99; 2.16) | 1.13 (0.69; 1.87) | P | . | . | . | . | . |
| 2.44 (1.21; 4.90) | 1.04 (0.44; 2.45) | 1.85 (1.15; 2.97) | 1.96 (1.32; 2.91) | 1.92 (1.23; 3.00) | 1.49 (1.11; 2.00) | 1.31 (0.75; 2.31) | P+D | . | . | . | . |
| 0.74 (0.26; 2.10) | 0.32 (0.10; 1.01) | 0.56 (0.23; 1.36) | 0.60 (0.27; 1.32) | 0.59 (0.24; 1.43) | 0.45 (0.19; 1.07) | 0.40 (0.16; 1.03) | 0.30 (0.13; 0.74) | R | 1.63 (1.01; 2.61) | . | . |
| 1.21 (0.48; 3.05) | 0.52 (0.18; 1.48) | 0.92 (0.43; 1.93) | 0.97 (0.52; 1.83) | 0.95 (0.45; 2.02) | 0.74 (0.36; 1.51) | 0.65 (0.29; 1.47) | 0.50 (0.24; 1.05) | 1.63 (1.01; 2.61) | R+D | . | . |
| 1.29 (0.68; 2.47) | 0.55 (0.25; 1.25) | 0.98 (0.64; 1.49) | 1.04 (0.62; 1.75) | 1.02 (0.71; 1.46) | 0.79 (0.48; 1.29) | 0.70 (0.42; 1.16) | 0.53 (0.30; 0.93) | 1.74 (0.68; 4.49) | 1.07 (0.47; 2.43) | T | 1.61 (1.05; 2.48) |
| 2.08 (0.96; 4.52) | 0.89 (0.36; 2.24) | 1.58 (0.86; 2.88) | 1.68 (0.85; 3.29) | 1.64 (0.94; 2.87) | 1.27 (0.66; 2.45) | 1.12 (0.58; 2.18) | 0.85 (0.42; 1.73) | 2.80 (0.99; 7.93) | 1.72 (0.68; 4.35) | 1.61 (1.05; 2.48) | T+D |

P-score

P+D 0.9062

Do+D 0.8323

T+D 0.7961

P 0.7356

O+D 0.6348

G 0.4006

T 0.3775

R+D 0.3647

O 0.3494

G+D 0.3359

Do 0.1872

R 0.0796

Delayed complete control

| G | 1.12 (0.78; 1.60) | . | . | . | . | . |
| --- | --- | --- | --- | --- | --- | --- |
| 1.12 (0.78; 1.60) | G+D | . | 0.94 (0.82; 1.07) | . | 0.80 (0.71; 0.91) | 0.92 (0.60; 1.42) |
| 1.71 (1.08; 2.70) | 1.53 (1.15; 2.03) | O | 0.61 (0.48; 0.79) | 0.70 (0.52; 0.94) | . | . |
| 1.05 (0.71; 1.54) | 0.94 (0.82; 1.07) | 0.61 (0.48; 0.79) | O+D | . | . | . |
| 1.19 (0.69; 2.06) | 1.07 (0.71; 1.61) | 0.70 (0.52; 0.94) | 1.14 (0.77; 1.68) | P | . | . |
| 0.89 (0.61; 1.31) | 0.80 (0.71; 0.91) | 0.52 (0.38; 0.71) | 0.85 (0.71; 1.02) | 0.75 (0.49; 1.15) | P+D | . |
| 1.03 (0.59; 1.81) | 0.92 (0.60; 1.42) | 0.60 (0.36; 1.01) | 0.98 (0.63; 1.54) | 0.87 (0.48; 1.57) | 1.15 (0.74; 1.80) | R+D |

P-score

P+D 0.8862

G 0.6447

R+D 0.5908

O+D 0.5802

G+D 0.4062

P 0.3836

O 0.0082

35、The outcomes after the article of “Villalon A 2004” was deleted.

Acute nausea

| A+D | . | . | 1.16 (0.67; 2.00) | . | . | . | . | . | . | . | . |
| --- | --- | --- | --- | --- | --- | --- | --- | --- | --- | --- | --- |
| 0.91 (0.50; 1.64) | Do | 0.98 (0.85; 1.12) | . | 0.99 (0.68; 1.42) | . | . | . | . | . | . | . |
| 0.89 (0.50; 1.58) | 0.98 (0.86; 1.12) | G | 1.32 (1.06; 1.63) | 1.00 (0.93; 1.08) | . | . | . | 1.32 (0.96; 1.81) | . | 0.41 (0.23; 0.73) | . |
| 1.16 (0.67; 2.00) | 1.28 (1.01; 1.62) | 1.31 (1.07; 1.59) | G+D | . | 1.04 (0.91; 1.19) | . | 0.97 (0.84; 1.12) | . | 1.22 (0.66; 2.23) | . | . |
| 0.88 (0.49; 1.58) | 0.97 (0.84; 1.12) | 0.99 (0.93; 1.07) | 0.76 (0.62; 0.93) | O | 1.26 (0.74; 2.13) | 1.33 (0.80; 2.20) | . | 1.16 (0.72; 1.86) | . | 1.10 (0.78; 1.56) | . |
| 1.15 (0.66; 2.01) | 1.27 (0.99; 1.65) | 1.30 (1.04; 1.62) | 0.99 (0.89; 1.11) | 1.31 (1.04; 1.64) | O+D | . | 1.14 (0.93; 1.38) | . | . | . | . |
| 1.17 (0.54; 2.54) | 1.30 (0.77; 2.19) | 1.32 (0.79; 2.20) | 1.01 (0.59; 1.75) | 1.33 (0.80; 2.20) | 1.02 (0.59; 1.77) | P | . | . | . | . | . |
| 1.19 (0.68; 2.08) | 1.31 (1.01; 1.71) | 1.34 (1.06; 1.69) | 1.02 (0.90; 1.16) | 1.35 (1.06; 1.71) | 1.03 (0.90; 1.18) | 1.01 (0.58; 1.77) | P+D | . | . | . | . |
| 1.12 (0.59; 2.12) | 1.24 (0.92; 1.66) | 1.27 (0.97; 1.65) | 0.97 (0.70; 1.35) | 1.27 (0.97; 1.66) | 0.97 (0.69; 1.37) | 0.96 (0.54; 1.69) | 0.94 (0.67; 1.34) | R | . | . | . |
| 1.41 (0.63; 3.19) | 1.56 (0.81; 2.99) | 1.59 (0.84; 3.01) | 1.22 (0.66; 2.23) | 1.60 (0.84; 3.04) | 1.22 (0.66; 2.27) | 1.20 (0.53; 2.72) | 1.19 (0.64; 2.20) | 1.26 (0.63; 2.50) | R+D | . | . |
| 0.75 (0.39; 1.44) | 0.83 (0.60; 1.15) | 0.85 (0.63; 1.14) | 0.65 (0.45; 0.93) | 0.85 (0.63; 1.15) | 0.65 (0.45; 0.94) | 0.64 (0.36; 1.15) | 0.63 (0.43; 0.92) | 0.67 (0.45; 1.00) | 0.53 (0.26; 1.08) | T | 1.85 (1.25; 2.74) |
| 1.39 (0.65; 2.98) | 1.54 (0.92; 2.56) | 1.57 (0.96; 2.57) | 1.20 (0.70; 2.04) | 1.58 (0.96; 2.58) | 1.21 (0.70; 2.07) | 1.19 (0.59; 2.40) | 1.17 (0.68; 2.02) | 1.24 (0.71; 2.17) | 0.99 (0.44; 2.21) | 1.85 (1.25; 2.74) | T+D |

P-score

T+D 0.8035

R+D 0.7839

P+D 0.7024

G+D 0.6484

O+D 0.6332

P 0.6255

R 0.6021

A+D 0.4300

Do 0.2685

G 0.2252

O 0.2073

T 0.0701

Acute vomiting

| Do | 0.96 (0.80; 1.15) | . | 0.99 (0.85; 1.14) | . | . | . | . | . | . | . |
| --- | --- | --- | --- | --- | --- | --- | --- | --- | --- | --- |
| 0.93 (0.82; 1.05) | G | 1.57 (1.29; 1.91) | 1.09 (0.99; 1.19) | . | 1.61 (0.94; 2.76) | . | 1.32 (0.94; 1.86) | . | 0.91 (0.65; 1.27) | . |
| 1.42 (1.15; 1.76) | 1.53 (1.28; 1.83) | G+D | . | 1.06 (0.88; 1.27) | . | 1.05 (0.81; 1.35) | . | 0.84 (0.48; 1.46) | . | . |
| 1.01 (0.89; 1.14) | 1.08 (1.00; 1.18) | 0.71 (0.59; 0.85) | O | 1.27 (0.88; 1.84) | 1.98 (0.60; 6.52) | . | 1.06 (0.74; 1.52) | . | 0.78 (0.60; 1.02) | . |
| 1.39 (1.09; 1.76) | 1.49 (1.21; 1.84) | 0.97 (0.84; 1.13) | 1.38 (1.11; 1.71) | O+D | . | 1.32 (1.09; 1.60) | . | . | . | . |
| 1.57 (0.95; 2.60) | 1.69 (1.04; 2.77) | 1.10 (0.66; 1.86) | 1.56 (0.95; 2.56) | 1.13 (0.66; 1.93) | P | . | . | . | . | . |
| 1.70 (1.30; 2.22) | 1.83 (1.44; 2.32) | 1.19 (1.00; 1.43) | 1.69 (1.32; 2.16) | 1.22 (1.04; 1.44) | 1.08 (0.63; 1.86) | P+D | . | . | . | . |
| 1.15 (0.87; 1.51) | 1.24 (0.96; 1.59) | 0.81 (0.59; 1.09) | 1.14 (0.89; 1.47) | 0.83 (0.60; 1.14) | 0.73 (0.42; 1.27) | 0.68 (0.48; 0.95) | R | . | . | . |
| 1.20 (0.66; 2.17) | 1.29 (0.72; 2.31) | 0.84 (0.48; 1.46) | 1.19 (0.66; 2.13) | 0.86 (0.49; 1.53) | 0.76 (0.36; 1.63) | 0.70 (0.39; 1.26) | 1.04 (0.55; 1.96) | R+D | . | . |
| 0.79 (0.61; 1.03) | 0.86 (0.68; 1.08) | 0.56 (0.42; 0.75) | 0.79 (0.63; 1.00) | 0.57 (0.42; 0.78) | 0.51 (0.29; 0.87) | 0.47 (0.34; 0.65) | 0.69 (0.49; 0.97) | 0.66 (0.35; 1.24) | T | 1.99 (1.43; 2.78) |
| 1.58 (1.04; 2.41) | 1.70 (1.13; 2.57) | 1.11 (0.71; 1.73) | 1.57 (1.05; 2.36) | 1.14 (0.72; 1.80) | 1.01 (0.53; 1.91) | 0.93 (0.58; 1.50) | 1.38 (0.86; 2.22) | 1.32 (0.65; 2.69) | 1.99 (1.43; 2.78) | T+D |

P-score

P+D 0.9054

T+D 0.7938

P 0.7729

G+D 0.6982

O+D 0.6544

R+D 0.4850

R 0.4514

O 0.2982

Do 0.2801

G 0.1320

T 0.0286

Acute complete control

| G | 0.80 (0.61; 1.04) | 0.96 (0.89; 1.03) | . | . | . | . | 1.16 (0.92; 1.47) | . |
| --- | --- | --- | --- | --- | --- | --- | --- | --- |
| 0.86 (0.74; 1.01) | G+D | . | 0.99 (0.92; 1.06) | . | 0.98 (0.91; 1.05) | 1.05 (0.87; 1.27) | . | . |
| 0.95 (0.88; 1.02) | 1.10 (0.95; 1.28) | O | 0.94 (0.80; 1.12) | 0.98 (0.91; 1.05) | . | . | 1.13 (0.89; 1.44) | . |
| 0.87 (0.74; 1.01) | 1.01 (0.95; 1.07) | 0.91 (0.79; 1.06) | O+D | . | 0.93 (0.84; 1.03) | . | . | . |
| 0.93 (0.83; 1.03) | 1.08 (0.91; 1.27) | 0.98 (0.91; 1.05) | 1.07 (0.91; 1.26) | P | . | . | . | . |
| 0.83 (0.71; 0.98) | 0.96 (0.91; 1.03) | 0.87 (0.75; 1.02) | 0.96 (0.89; 1.03) | 0.90 (0.75; 1.07) | P+D | . | . | . |
| 0.91 (0.71; 1.16) | 1.05 (0.87; 1.27) | 0.95 (0.75; 1.22) | 1.04 (0.85; 1.28) | 0.98 (0.76; 1.26) | 1.09 (0.89; 1.33) | R+D | . | . |
| 1.12 (0.90; 1.39) | 1.30 (1.00; 1.69) | 1.18 (0.95; 1.47) | 1.29 (0.99; 1.67) | 1.21 (0.96; 1.52) | 1.35 (1.03; 1.76) | 1.24 (0.89; 1.71) | T | 0.65 (0.53; 0.80) |
| 0.73 (0.54; 0.98) | 0.84 (0.60; 1.18) | 0.77 (0.57; 1.03) | 0.84 (0.60; 1.17) | 0.78 (0.57; 1.07) | 0.87 (0.62; 1.23) | 0.80 (0.55; 1.18) | 0.65 (0.53; 0.80) | T+D |

P-score

T+D 0.9023

P+D 0.8245

G+D 0.6538

O+D 0.6138

R+D 0.4854

P 0.4533

O 0.3435

G 0.1677

T 0.0558

Delayed nausea

| Do | 1.99 (1.28; 3.08) | . | . | 0.91 (0.63; 1.31) | . | . | . | . | . | . |
| --- | --- | --- | --- | --- | --- | --- | --- | --- | --- | --- |
| 1.99 (1.28; 3.08) | Do+D | . | . | . | . | . | . | . | . | . |
| 1.00 (0.59; 1.69) | 0.50 (0.25; 1.00) | G | 0.97 (0.67; 1.40) | 1.17 (0.68; 2.02) | . | . | . | . | . | . |
| 1.08 (0.65; 1.79) | 0.54 (0.28; 1.06) | 1.09 (0.79; 1.50) | G+D | . | 1.07 (0.76; 1.51) | . | 1.41 (1.09; 1.83) | 1.02 (0.64; 1.61) | . | . |
| 0.91 (0.63; 1.31) | 0.46 (0.26; 0.81) | 0.92 (0.62; 1.35) | 0.84 (0.60; 1.19) | O | 1.43 (1.03; 1.98) | 1.42 (1.04; 1.95) | . | . | 1.21 (0.80; 1.83) | . |
| 1.20 (0.75; 1.91) | 0.60 (0.32; 1.14) | 1.20 (0.84; 1.73) | 1.11 (0.87; 1.40) | 1.31 (0.98; 1.76) | O+D | . | 1.37 (1.06; 1.78) | . | . | . |
| 1.30 (0.80; 2.10) | 0.65 (0.34; 1.25) | 1.30 (0.79; 2.15) | 1.20 (0.75; 1.92) | 1.42 (1.04; 1.95) | 1.09 (0.70; 1.67) | P | . | . | . | . |
| 1.58 (0.95; 2.62) | 0.80 (0.41; 1.55) | 1.59 (1.09; 2.30) | 1.46 (1.18; 1.82) | 1.73 (1.22; 2.46) | 1.32 (1.06; 1.64) | 1.22 (0.76; 1.95) | P+D | . | . | . |
| 1.10 (0.55; 2.18) | 0.55 (0.25; 1.24) | 1.10 (0.63; 1.94) | 1.02 (0.64; 1.61) | 1.20 (0.67; 2.15) | 0.92 (0.55; 1.54) | 0.84 (0.44; 1.63) | 0.69 (0.42; 1.16) | R+D | . | . |
| 1.10 (0.64; 1.91) | 0.55 (0.27; 1.12) | 1.11 (0.63; 1.95) | 1.02 (0.59; 1.75) | 1.21 (0.80; 1.83) | 0.92 (0.55; 1.53) | 0.85 (0.50; 1.43) | 0.70 (0.41; 1.20) | 1.00 (0.49; 2.05) | T | 1.49 (1.00; 2.22) |
| 1.64 (0.83; 3.23) | 0.82 (0.37; 1.85) | 1.64 (0.82; 3.29) | 1.51 (0.77; 2.96) | 1.79 (1.01; 3.19) | 1.37 (0.72; 2.61) | 1.26 (0.65; 2.43) | 1.04 (0.53; 2.03) | 1.49 (0.66; 3.37) | 1.49 (1.00; 2.22) | T+D |

P-score

Do+D 0.9077

P+D 0.8272

T+D 0.7956

P 0.6095

O+D 0.5223

R+D 0.4001

T 0.3933

G+D 0.3661

Do 0.2819

G 0.2626

O 0.1339

Delayed vomiting

| Do | 2.33 (1.54; 3.53) | . | . | 1.27 (0.79; 2.03) | . | . | . | . | . | . |
| --- | --- | --- | --- | --- | --- | --- | --- | --- | --- | --- |
| 2.33 (1.54; 3.53) | Do+D | . | . | . | . | . | . | . | . | . |
| 1.33 (0.77; 2.28) | 0.57 (0.29; 1.12) | G | 0.93 (0.62; 1.40) | 1.09 (0.77; 1.55) | . | 1.12 (0.72; 1.72) | . | . | 1.14 (0.67; 1.96) | . |
| 1.25 (0.70; 2.25) | 0.54 (0.26; 1.10) | 0.95 (0.68; 1.32) | G+D | . | 0.99 (0.69; 1.42) | . | 3.52 (1.95; 6.35) | 0.97 (0.55; 1.73) | . | . |
| 1.27 (0.79; 2.03) | 0.54 (0.29; 1.02) | 0.96 (0.73; 1.25) | 1.01 (0.71; 1.43) | O | 1.28 (0.89; 1.84) | 1.88 (1.16; 3.02) | . | . | 0.98 (0.71; 1.34) | . |
| 1.61 (0.92; 2.82) | 0.69 (0.34; 1.38) | 1.21 (0.86; 1.71) | 1.28 (0.97; 1.69) | 1.27 (0.93; 1.72) | O+D | . | 1.33 (1.10; 1.60) | . | . | . |
| 1.83 (1.02; 3.30) | 0.79 (0.38; 1.61) | 1.38 (0.98; 1.95) | 1.46 (0.93; 2.29) | 1.44 (1.01; 2.05) | 1.14 (0.73; 1.77) | P | . | . | . | . |
| 2.28 (1.27; 4.09) | 0.98 (0.48; 2.00) | 1.72 (1.17; 2.51) | 1.82 (1.33; 2.47) | 1.79 (1.26; 2.55) | 1.42 (1.18; 1.70) | 1.24 (0.78; 1.99) | P+D | . | . | . |
| 1.22 (0.54; 2.77) | 0.52 (0.21; 1.31) | 0.92 (0.47; 1.79) | 0.97 (0.55; 1.73) | 0.96 (0.49; 1.88) | 0.76 (0.40; 1.44) | 0.67 (0.32; 1.38) | 0.54 (0.28; 1.03) | R+D | . | . |
| 1.29 (0.74; 2.25) | 0.55 (0.28; 1.11) | 0.97 (0.67; 1.40) | 1.03 (0.66; 1.60) | 1.01 (0.75; 1.38) | 0.80 (0.53; 1.22) | 0.70 (0.45; 1.10) | 0.57 (0.36; 0.89) | 1.06 (0.51; 2.18) | T | 1.59 (1.10; 2.29) |
| 2.04 (1.05; 3.99) | 0.88 (0.40; 1.92) | 1.54 (0.92; 2.59) | 1.63 (0.92; 2.90) | 1.61 (1.00; 2.59) | 1.27 (0.73; 2.23) | 1.12 (0.63; 1.99) | 0.90 (0.50; 1.61) | 1.68 (0.74; 3.78) | 1.59 (1.10; 2.29) | T+D |

P-score

P+D 0.8888

Do+D 0.8497

T+D 0.7924

P 0.7194

O+D 0.5974

G 0.3531

T 0.3173

R+D 0.3002

O 0.2895

G+D 0.2789

Do 0.1134

36、The outcomes after the article of “Yu ZC 2009” was deleted.

Acute vomiting

| Do | 0.96 (0.79; 1.15) | . | 0.99 (0.85; 1.15) | . | . | . | . | . | . | . |
| --- | --- | --- | --- | --- | --- | --- | --- | --- | --- | --- |
| 0.93 (0.82; 1.06) | G | 1.57 (1.28; 1.91) | 1.09 (0.99; 1.19) | . | . | . | 1.32 (0.93; 1.87) | . | 0.91 (0.65; 1.27) | . |
| 1.46 (1.18; 1.80) | 1.57 (1.32; 1.87) | G+D | . | 1.06 (0.88; 1.28) | . | 1.05 (0.81; 1.36) | . | 0.84 (0.48; 1.47) | . | . |
| 1.01 (0.89; 1.14) | 1.08 (1.00; 1.18) | 0.69 (0.57; 0.83) | O | 1.28 (0.88; 1.85) | 1.98 (0.60; 6.53) | . | 1.06 (0.74; 1.52) | . | 0.78 (0.60; 1.03) | . |
| 1.41 (1.11; 1.80) | 1.52 (1.23; 1.88) | 0.97 (0.83; 1.13) | 1.40 (1.13; 1.74) | O+D | . | 1.32 (1.08; 1.60) | . | . | . | . |
| 2.00 (0.60; 6.61) | 2.15 (0.65; 7.10) | 1.37 (0.41; 4.58) | 1.98 (0.60; 6.53) | 1.41 (0.42; 4.74) | P | . | . | . | . | . |
| 1.73 (1.32; 2.27) | 1.87 (1.47; 2.38) | 1.19 (0.99; 1.43) | 1.72 (1.34; 2.20) | 1.23 (1.04; 1.45) | 0.87 (0.26; 2.93) | P+D | . | . | . | . |
| 1.10 (0.84; 1.43) | 1.18 (0.93; 1.50) | 0.75 (0.57; 1.00) | 1.09 (0.86; 1.38) | 0.78 (0.57; 1.05) | 0.55 (0.16; 1.85) | 0.63 (0.46; 0.88) | R | 1.58 (1.05; 2.38) | . | . |
| 1.54 (1.04; 2.27) | 1.66 (1.14; 2.40) | 1.05 (0.72; 1.54) | 1.53 (1.05; 2.22) | 1.09 (0.73; 1.62) | 0.77 (0.22; 2.68) | 0.89 (0.59; 1.34) | 1.40 (1.00; 1.98) | R+D | . | . |
| 0.79 (0.61; 1.03) | 0.85 (0.67; 1.09) | 0.54 (0.41; 0.73) | 0.79 (0.62; 1.00) | 0.56 (0.41; 0.77) | 0.40 (0.12; 1.34) | 0.46 (0.33; 0.64) | 0.72 (0.52; 1.01) | 0.52 (0.33; 0.80) | T | 1.99 (1.42; 2.79) |
| 1.58 (1.03; 2.42) | 1.70 (1.13; 2.57) | 1.09 (0.69; 1.70) | 1.57 (1.04; 2.37) | 1.12 (0.71; 1.77) | 0.79 (0.22; 2.79) | 0.91 (0.57; 1.47) | 1.44 (0.90; 2.32) | 1.03 (0.59; 1.79) | 1.99 (1.42; 2.79) | T+D |

P-score

P+D 0.8731

P 0.7705

T+D 0.7473

R+D 0.7292

G+D 0.6713

O+D 0.6244

R 0.3723

O 0.2894

Do 0.2699

G 0.1262

T 0.0264

Delayed vomiting

| Do | 2.33 (1.58; 3.44) | . | . | 1.27 (0.81; 1.98) | . | . | . | . | . | . | . |
| --- | --- | --- | --- | --- | --- | --- | --- | --- | --- | --- | --- |
| 2.33 (1.58; 3.44) | Do+D | . | . | . | . | . | . | . | . | . | . |
| 1.20 (0.71; 2.04) | 0.52 (0.27; 0.99) | G | 0.93 (0.63; 1.37) | 1.09 (0.78; 1.53) | . | . | . | . | . | 1.14 (0.68; 1.93) | . |
| 1.19 (0.68; 2.08) | 0.51 (0.26; 1.01) | 0.99 (0.72; 1.36) | G+D | . | 0.99 (0.71; 1.37) | . | 3.52 (1.99; 6.23) | . | 0.97 (0.56; 1.70) | . | . |
| 1.27 (0.81; 1.98) | 0.54 (0.30; 0.98) | 1.05 (0.80; 1.40) | 1.06 (0.76; 1.49) | O | 1.28 (0.90; 1.80) | 1.89 (1.18; 3.01) | . | . | . | 0.97 (0.72; 1.31) | . |
| 1.54 (0.90; 2.63) | 0.66 (0.34; 1.28) | 1.28 (0.91; 1.79) | 1.29 (0.99; 1.67) | 1.21 (0.90; 1.63) | O+D | . | 1.33 (1.12; 1.57) | . | . | . | . |
| 2.39 (1.26; 4.56) | 1.03 (0.48; 2.18) | 1.99 (1.15; 3.43) | 2.01 (1.13; 3.56) | 1.89 (1.18; 3.01) | 1.56 (0.90; 2.71) | P | . | . | . | . | . |
| 2.16 (1.24; 3.78) | 0.93 (0.47; 1.83) | 1.80 (1.25; 2.59) | 1.81 (1.36; 2.42) | 1.71 (1.22; 2.38) | 1.41 (1.19; 1.66) | 0.90 (0.51; 1.61) | P+D | . | . | . | . |
| 0.71 (0.30; 1.70) | 0.31 (0.12; 0.79) | 0.59 (0.28; 1.24) | 0.60 (0.31; 1.17) | 0.56 (0.27; 1.19) | 0.46 (0.23; 0.95) | 0.30 (0.12; 0.72) | 0.33 (0.16; 0.68) | R | 1.63 (1.13; 2.35) | . | . |
| 1.16 (0.53; 2.55) | 0.50 (0.21; 1.20) | 0.96 (0.51; 1.83) | 0.97 (0.56; 1.70) | 0.91 (0.48; 1.75) | 0.75 (0.41; 1.40) | 0.48 (0.22; 1.08) | 0.54 (0.29; 1.00) | 1.63 (1.13; 2.35) | R+D | . | . |
| 1.26 (0.74; 2.14) | 0.54 (0.28; 1.04) | 1.05 (0.73; 1.50) | 1.06 (0.70; 1.61) | 0.99 (0.75; 1.32) | 0.82 (0.55; 1.22) | 0.53 (0.30; 0.91) | 0.58 (0.38; 0.89) | 1.77 (0.80; 3.89) | 1.09 (0.54; 2.18) | T | 1.58 (1.12; 2.22) |
| 1.99 (1.06; 3.74) | 0.85 (0.41; 1.79) | 1.65 (1.01; 2.72) | 1.67 (0.97; 2.86) | 1.57 (1.00; 2.45) | 1.29 (0.77; 2.19) | 0.83 (0.44; 1.59) | 0.92 (0.53; 1.59) | 2.79 (1.18; 6.59) | 1.72 (0.79; 3.73) | 1.58 (1.12; 2.22) | T+D |

P-score

P 0.8854

Do+D 0.8582

P+D 0.8509

T+D 0.7859

O+D 0.6094

O 0.3957

T 0.3834

R+D 0.3413

G 0.3294

G+D 0.3213

Do 0.1882

R 0.0509
