# Supplementary material for: Comparative efficacy of 5-hydroxytryptamine-3 (5-HT3) receptor antagonists with or without dexamethasone for prevention of chemotherapy-induced nausea and vomiting following highly emetogenic chemotherapy (HEC): a network meta-analysis
Source: PeerJ. 2026 Apr 2;14:e21047. doi: 10.7717/peerj.21047 (PMC13050518; doi:10.7717/peerj.21047)
Supplement: Supplemental Information 6 [file peerj-14-21047-s006.docx]

**Supplement 5 The heterogeneity within the network**

|  | tau^^2^ | tau | I^^2^ |
| --- | --- | --- | --- |
| Acute nausea | 0 | 0 | 0% [0.0%; 48.9%] |
| Acute vomiting | 0.0050 | 0.0708 | 18% [0.0%; 47.2%] |
| Acute complete control | 0.0050 | 0.0553 | 0% [0.0%; 52.3%] |
| Delayed nausea | 0.0256 | 0.1601 | 65.5% [26.5%; 83.8%] |
| Delayed vomiting | 0.0231 | 0.1521 | 49% [5.4%; 72.5%] |
| Delayed complete control | 0.0078 | 0.0886 | 37.9% [0.0%; 78.7%] |
