# Supplementary material for: Comparative efficacy of 5-hydroxytryptamine-3 (5-HT3) receptor antagonists with or without dexamethasone for prevention of chemotherapy-induced nausea and vomiting following highly emetogenic chemotherapy (HEC): a network meta-analysis
Source: PeerJ. 2026 Apr 2;14:e21047. doi: 10.7717/peerj.21047 (PMC13050518; doi:10.7717/peerj.21047)
Supplement: Supplemental Information 7 [file peerj-14-21047-s007.docx]

**Supplement 6 Inconsistency between studies**

|  | Q | df | p-value | tau.within | tau^2^.within |
| --- | --- | --- | --- | --- | --- |
| Acute nausea | 10.96 | 6 | 0.0896 | 0 | 0 |
| Acute vomiting | 8.21 | 9 | 0.5130 | 0.0741 | 0.0055 |
| Acute complete control | 1.75 | 3 | 0.6249 | 0 | 0 |
| Delayed nausea | 1.39 | 2 | 0.4987 | 0.1836 | 0.0337 |
| Delayed vomiting | 3.15 | 5 | 0.6766 | 0.1968 | 0.0387 |
| Delayed complete control | 1.08 | 1 | 0.2986 | 0.0821 | 0.0067 |
