# Supplementary material for: Comparative efficacy of 5-hydroxytryptamine-3 (5-HT3) receptor antagonists with or without dexamethasone for prevention of chemotherapy-induced nausea and vomiting following highly emetogenic chemotherapy (HEC): a network meta-analysis
Source: PeerJ. 2026 Apr 2;14:e21047. doi: 10.7717/peerj.21047 (PMC13050518; doi:10.7717/peerj.21047)
Supplement: Supplemental Information 8 [file peerj-14-21047-s008.docx]

Supplement 7 Netsplit of the outcomes

Netsplit of acute nausea

| comparison | k | prop | nma | direct | indir. | RoR | z | p-value |
| --- | --- | --- | --- | --- | --- | --- | --- | --- |
| A+D vs Do | 0 | 0 | 0.8907 | . | 0.8907 | . | . | . |
| A+D vs G | 0 | 0 | 0.8732 | . | 0.8732 | . | . | . |
| A+D vs G+D | 1 | 1 | 1.1607 | 1.1607 | . | . | . | . |
| A+D vs O | 0 | 0 | 0.8673 | . | 0.8673 | . | . | . |
| A+D vs O+D | 0 | 0 | 1.1534 | . | 1.1534 | . | . | . |
| A+D vs P | 0 | 0 | 1.1546 | . | 1.1546 | . | . | . |
| A+D vs P+D | 0 | 0 | 1.1874 | . | 1.1874 | . | . | . |
| A+D vs R | 0 | 0 | 1.0732 | . | 1.0732 | . | . | . |
| A+D vs R+D | 0 | 0 | 1.652 | . | 1.652 | . | . | . |
| A+D vs T | 0 | 0 | 0.7394 | . | 0.7394 | . | . | . |
| A+D vs T+D | 0 | 0 | 1.3712 | . | 1.3712 | . | . | . |
| Do vs G | 2 | 0.88 | 0.9804 | 0.9786 | 0.9935 | 0.985 | -0.07 | 0.9405 |
| Do vs G+D | 0 | 0 | 1.3031 | . | 1.3031 | . | . | . |
| Do vs O | 1 | 0.15 | 0.9736 | 0.9862 | 0.9715 | 1.0152 | 0.07 | 0.9405 |
| Do vs O+D | 0 | 0 | 1.2949 | . | 1.2949 | . | . | . |
| Do vs P | 0 | 0 | 1.2962 | . | 1.2962 | . | . | . |
| Do vs P+D | 0 | 0 | 1.333 | . | 1.333 | . | . | . |
| Do vs R | 0 | 0 | 1.2048 | . | 1.2048 | . | . | . |
| Do vs R+D | 0 | 0 | 1.8546 | . | 1.8546 | . | . | . |
| Do vs T | 0 | 0 | 0.8301 | . | 0.8301 | . | . | . |
| Do vs T+D | 0 | 0 | 1.5393 | . | 1.5393 | . | . | . |
| G vs G+D | 2 | 0.81 | 1.3292 | 1.316 | 1.3873 | 0.9486 | -0.21 | 0.8312 |
| G vs O | 7 | 0.93 | 0.9931 | 1.0008 | 0.8944 | 1.1189 | 0.82 | 0.4115 |
| G vs O+D | 0 | 0 | 1.3208 | . | 1.3208 | . | . | . |
| G vs P | 0 | 0 | 1.3222 | . | 1.3222 | . | . | . |
| G vs P+D | 0 | 0 | 1.3598 | . | 1.3598 | . | . | . |
| G vs R | 1 | 0.62 | 1.229 | 1.3211 | 1.0942 | 1.2073 | 0.73 | 0.4659 |
| G vs R+D | 0 | 0 | 1.8917 | . | 1.8917 | . | . | . |
| G vs T | 1 | 0.27 | 0.8467 | 0.4076 | 1.1067 | 0.3683 | -2.88 | 0.0039 |
| G vs T+D | 0 | 0 | 1.5702 | . | 1.5702 | . | . | . |
| G+D vs O | 0 | 0 | 0.7472 | . | 0.7472 | . | . | . |
| G+D vs O+D | 2 | 0.75 | 0.9937 | 1.0415 | 0.866 | 1.2026 | 1.41 | 0.1598 |
| G+D vs P | 0 | 0 | 0.9947 | . | 0.9947 | . | . | . |
| G+D vs P+D | 1 | 0.73 | 1.023 | 0.9712 | 1.1765 | 0.8255 | -1.38 | 0.1677 |
| G+D vs R | 0 | 0 | 0.9246 | . | 0.9246 | . | . | . |
| G+D vs R+D | 1 | 0.37 | 1.4232 | 1.2162 | 1.561 | 0.7791 | -0.64 | 0.5209 |
| G+D vs T | 0 | 0 | 0.637 | . | 0.637 | . | . | . |
| G+D vs T+D | 0 | 0 | 1.1813 | . | 1.1813 | . | . | . |
| O+D vs O | 1 | 0.18 | 0.7519 | 0.7959 | 0.7429 | 1.0713 | 0.23 | 0.8154 |
| P vs O | 3 | 1 | 0.7511 | 0.7511 | . | . | . | . |
| P+D vs O | 0 | 0 | 0.7304 | . | 0.7304 | . | . | . |
| R vs O | 1 | 0.28 | 0.8081 | 0.8651 | 0.787 | 1.0992 | 0.33 | 0.7393 |
| R+D vs O | 0 | 0 | 0.525 | . | 0.525 | . | . | . |
| T vs O | 1 | 0.74 | 1.1729 | 0.9064 | 2.4609 | 0.3683 | -2.88 | 0.0039 |
| T+D vs O | 0 | 0 | 0.6325 | . | 0.6325 | . | . | . |
| O+D vs P | 0 | 0 | 1.001 | . | 1.001 | . | . | . |
| O+D vs P+D | 2 | 0.49 | 1.0295 | 1.1361 | 0.9378 | 1.2115 | 1.38 | 0.1677 |
| O+D vs R | 0 | 0 | 0.9305 | . | 0.9305 | . | . | . |
| O+D vs R+D | 0 | 0 | 1.4323 | . | 1.4323 | . | . | . |
| O+D vs T | 0 | 0 | 0.6411 | . | 0.6411 | . | . | . |
| O+D vs T+D | 0 | 0 | 1.1888 | . | 1.1888 | . | . | . |
| P vs P+D | 0 | 0 | 1.0284 | . | 1.0284 | . | . | . |
| P vs R | 0 | 0 | 0.9295 | . | 0.9295 | . | . | . |
| P vs R+D | 0 | 0 | 1.4308 | . | 1.4308 | . | . | . |
| P vs T | 0 | 0 | 0.6404 | . | 0.6404 | . | . | . |
| P vs T+D | 0 | 0 | 1.1876 | . | 1.1876 | . | . | . |
| P+D vs R | 0 | 0 | 0.9038 | . | 0.9038 | . | . | . |
| P+D vs R+D | 0 | 0 | 1.3912 | . | 1.3912 | . | . | . |
| P+D vs T | 0 | 0 | 0.6227 | . | 0.6227 | . | . | . |
| P+D vs T+D | 0 | 0 | 1.1548 | . | 1.1548 | . | . | . |
| R vs R+D | 1 | 0.81 | 1.5393 | 1.6123 | 1.2562 | 1.2835 | 0.64 | 0.5209 |
| R vs T | 0 | 0 | 0.689 | . | 0.689 | . | . | . |
| R vs T+D | 0 | 0 | 1.2777 | . | 1.2777 | . | . | . |
| R+D vs T | 0 | 0 | 0.4476 | . | 0.4476 | . | . | . |
| R+D vs T+D | 0 | 0 | 0.83 | . | 0.83 | . | . | . |
| T vs T+D | 1 | 1 | 1.8544 | 1.8544 | . | . | . | . |

Netsplit of acute vomiting

| comparison | k | prop | nma | direct | indir. | RoR | z | p-value |
| --- | --- | --- | --- | --- | --- | --- | --- | --- |
| Do vs G | 2 | 0.48 | 0.9287 | 0.957 | 0.9035 | 1.0592 | 0.45 | 0.6562 |
| Do vs G+D | 0 | 0 | 1.4564 | . | 1.4564 | . | . | . |
| Do vs O | 3 | 0.64 | 1.0057 | 0.9854 | 1.0437 | 0.9441 | -0.45 | 0.6562 |
| Do vs O+D | 0 | 0 | 1.4135 | . | 1.4135 | . | . | . |
| Do vs P | 0 | 0 | 1.5716 | . | 1.5716 | . | . | . |
| Do vs P+D | 0 | 0 | 1.7324 | . | 1.7324 | . | . | . |
| Do vs R | 0 | 0 | 1.0959 | . | 1.0959 | . | . | . |
| Do vs R+D | 0 | 0 | 1.5379 | . | 1.5379 | . | . | . |
| Do vs T | 0 | 0 | 0.7939 | . | 0.7939 | . | . | . |
| Do vs T+D | 0 | 0 | 1.5811 | . | 1.5811 | . | . | . |
| G vs G+D | 3 | 0.77 | 1.5682 | 1.5665 | 1.5738 | 0.9953 | -0.02 | 0.9822 |
| G vs O | 9 | 0.79 | 1.0829 | 1.0857 | 1.0725 | 1.0123 | 0.12 | 0.907 |
| G vs O+D | 0 | 0 | 1.522 | . | 1.522 | . | . | . |
| G vs P | 1 | 0.83 | 1.6923 | 1.6111 | 2.1515 | 0.7488 | -0.43 | 0.665 |
| G vs P+D | 0 | 0 | 1.8654 | . | 1.8654 | . | . | . |
| G vs R | 1 | 0.48 | 1.18 | 1.3211 | 1.064 | 1.2416 | 0.89 | 0.3717 |
| G vs R+D | 0 | 0 | 1.656 | . | 1.656 | . | . | . |
| G vs T | 2 | 0.5 | 0.8548 | 0.9108 | 0.8022 | 1.1354 | 0.53 | 0.5995 |
| G vs T+D | 0 | 0 | 1.7024 | . | 1.7024 | . | . | . |
| G+D vs O | 0 | 0 | 0.6906 | . | 0.6906 | . | . | . |
| G+D vs O+D | 2 | 0.66 | 0.9706 | 1.0615 | 0.8168 | 1.2996 | 1.63 | 0.1025 |
| G+D vs P | 0 | 0 | 1.0791 | . | 1.0791 | . | . | . |
| G+D vs P+D | 1 | 0.5 | 1.1895 | 1.0484 | 1.35 | 0.7766 | -1.37 | 0.1705 |
| G+D vs R | 0 | 0 | 0.7525 | . | 0.7525 | . | . | . |
| G+D vs R+D | 2 | 0.45 | 1.056 | 0.8404 | 1.2772 | 0.658 | -1.09 | 0.2748 |
| G+D vs T | 0 | 0 | 0.5451 | . | 0.5451 | . | . | . |
| G+D vs T+D | 0 | 0 | 1.0856 | . | 1.0856 | . | . | . |
| O+D vs O | 3 | 0.33 | 0.7115 | 0.7845 | 0.6777 | 1.1576 | 0.63 | 0.5263 |
| P vs O | 3 | 0.17 | 0.6399 | 0.5039 | 0.673 | 0.7488 | -0.43 | 0.665 |
| P+D vs O | 0 | 0 | 0.5805 | . | 0.5805 | . | . | . |
| R vs O | 1 | 0.44 | 0.9178 | 0.944 | 0.8976 | 1.0517 | 0.21 | 0.8371 |
| R+D vs O | 0 | 0 | 0.654 | . | 0.654 | . | . | . |
| T vs O | 2 | 0.75 | 1.2668 | 1.2783 | 1.2339 | 1.036 | 0.13 | 0.8972 |
| T+D vs O | 0 | 0 | 0.6361 | . | 0.6361 | . | . | . |
| O+D vs P | 0 | 0 | 1.1118 | . | 1.1118 | . | . | . |
| O+D vs P+D | 4 | 0.72 | 1.2256 | 1.3164 | 1.0224 | 1.2876 | 1.37 | 0.1705 |
| O+D vs R | 0 | 0 | 0.7753 | . | 0.7753 | . | . | . |
| O+D vs R+D | 0 | 0 | 1.088 | . | 1.088 | . | . | . |
| O+D vs T | 0 | 0 | 0.5616 | . | 0.5616 | . | . | . |
| O+D vs T+D | 0 | 0 | 1.1185 | . | 1.1185 | . | . | . |
| P vs P+D | 0 | 0 | 1.1023 | . | 1.1023 | . | . | . |
| P vs R | 0 | 0 | 0.6973 | . | 0.6973 | . | . | . |
| P vs R+D | 0 | 0 | 0.9786 | . | 0.9786 | . | . | . |
| P vs T | 0 | 0 | 0.5052 | . | 0.5052 | . | . | . |
| P vs T+D | 0 | 0 | 1.006 | . | 1.006 | . | . | . |
| P+D vs R | 0 | 0 | 0.6326 | . | 0.6326 | . | . | . |
| P+D vs R+D | 0 | 0 | 0.8877 | . | 0.8877 | . | . | . |
| P+D vs T | 0 | 0 | 0.4583 | . | 0.4583 | . | . | . |
| P+D vs T+D | 0 | 0 | 0.9126 | . | 0.9126 | . | . | . |
| R vs R+D | 1 | 0.71 | 1.4034 | 1.5837 | 1.0421 | 1.5197 | 1.09 | 0.2748 |
| R vs T | 0 | 0 | 0.7245 | . | 0.7245 | . | . | . |
| R vs T+D | 0 | 0 | 1.4428 | . | 1.4428 | . | . | . |
| R+D vs T | 0 | 0 | 0.5162 | . | 0.5162 | . | . | . |
| R+D vs T+D | 0 | 0 | 1.0281 | . | 1.0281 | . | . | . |
| T vs T+D | 2 | 1 | 1.9915 | 1.9915 | . | . | . | . |

Netsplit of acute complete control

| comparison | k | prop | nma | direct | indir. | RoR | z | p-value |
| --- | --- | --- | --- | --- | --- | --- | --- | --- |
| G vs G+D | 1 | 0.36 | 0.8619 | 0.7988 | 0.8999 | 0.8877 | -0.71 | 0.4771 |
| G vs O | 8 | 0.94 | 0.9507 | 0.957 | 0.8495 | 1.1265 | 0.71 | 0.4771 |
| G vs O+D | 0 | 0 | 0.869 | . | 0.869 | . | . | . |
| G vs P | 0 | 0 | 0.9274 | . | 0.9274 | . | . | . |
| G vs P+D | 0 | 0 | 0.8313 | . | 0.8313 | . | . | . |
| G vs R | 0 | 0 | 1.1431 | . | 1.1431 | . | . | . |
| G vs R+D | 0 | 0 | 0.9061 | . | 0.9061 | . | . | . |
| G vs T | 1 | 0.86 | 1.1206 | 1.1615 | 0.8958 | 1.2967 | 0.8 | 0.4217 |
| G vs T+D | 0 | 0 | 0.7273 | . | 0.7273 | . | . | . |
| G+D vs O | 0 | 0 | 1.103 | . | 1.103 | . | . | . |
| G+D vs O+D | 2 | 0.74 | 1.0082 | 0.9897 | 1.0616 | 0.9323 | -1.02 | 0.308 |
| G+D vs P | 0 | 0 | 1.076 | . | 1.076 | . | . | . |
| G+D vs P+D | 1 | 0.74 | 0.9646 | 0.9783 | 0.926 | 1.0564 | 0.76 | 0.446 |
| G+D vs R | 0 | 0 | 1.3262 | . | 1.3262 | . | . | . |
| G+D vs R+D | 2 | 1 | 1.0513 | 1.0513 | . | . | . | . |
| G+D vs T | 0 | 0 | 1.3002 | . | 1.3002 | . | . | . |
| G+D vs T+D | 0 | 0 | 0.8439 | . | 0.8439 | . | . | . |
| O+D vs O | 2 | 0.73 | 1.094 | 1.059 | 1.193 | 0.8877 | -0.71 | 0.4771 |
| P vs O | 3 | 1 | 1.0251 | 1.0251 | . | . | . | . |
| P+D vs O | 0 | 0 | 1.1435 | . | 1.1435 | . | . | . |
| R vs O | 0 | 0 | 0.8317 | . | 0.8317 | . | . | . |
| R+D vs O | 0 | 0 | 1.0492 | . | 1.0492 | . | . | . |
| T vs O | 1 | 0.83 | 0.8483 | 0.8836 | 0.6956 | 1.2703 | 0.8 | 0.4217 |
| T+D vs O | 0 | 0 | 1.3071 | . | 1.3071 | . | . | . |
| O+D vs P | 0 | 0 | 1.0672 | . | 1.0672 | . | . | . |
| O+D vs P+D | 2 | 0.49 | 0.9567 | 0.9301 | 0.9826 | 0.9466 | -0.76 | 0.446 |
| O+D vs R | 0 | 0 | 1.3154 | . | 1.3154 | . | . | . |
| O+D vs R+D | 0 | 0 | 1.0427 | . | 1.0427 | . | . | . |
| O+D vs T | 0 | 0 | 1.2896 | . | 1.2896 | . | . | . |
| O+D vs T+D | 0 | 0 | 0.837 | . | 0.837 | . | . | . |
| P vs P+D | 0 | 0 | 0.8964 | . | 0.8964 | . | . | . |
| P vs R | 0 | 0 | 1.2325 | . | 1.2325 | . | . | . |
| P vs R+D | 0 | 0 | 0.977 | . | 0.977 | . | . | . |
| P vs T | 0 | 0 | 1.2083 | . | 1.2083 | . | . | . |
| P vs T+D | 0 | 0 | 0.7842 | . | 0.7842 | . | . | . |
| P+D vs R | 0 | 0 | 1.375 | . | 1.375 | . | . | . |
| P+D vs R+D | 0 | 0 | 1.0899 | . | 1.0899 | . | . | . |
| P+D vs T | 0 | 0 | 1.348 | . | 1.348 | . | . | . |
| P+D vs T+D | 0 | 0 | 0.8749 | . | 0.8749 | . | . | . |
| R vs R+D | 1 | 1 | 0.7927 | 0.7927 | . | . | . | . |
| R vs T | 0 | 0 | 0.9804 | . | 0.9804 | . | . | . |
| R vs T+D | 0 | 0 | 0.6363 | . | 0.6363 | . | . | . |
| R+D vs T | 0 | 0 | 1.2368 | . | 1.2368 | . | . | . |
| R+D vs T+D | 0 | 0 | 0.8027 | . | 0.8027 | . | . | . |
| T vs T+D | 1 | 1 | 0.649 | 0.649 | . | . | . | . |

Netsplit of delayed nausea

| comparison | k | prop | nma | direct | indir. | RoR | z | p-value |
| --- | --- | --- | --- | --- | --- | --- | --- | --- |
| Do vs Do+D | 1 | 1 | 1.9886 | 1.9886 | . | . | . | . |
| Do vs G | 0 | 0 | 0.996 | . | 0.996 | . | . | . |
| Do vs G+D | 0 | 0 | 1.0815 | . | 1.0815 | . | . | . |
| Do vs O | 1 | 1 | 0.9128 | 0.9128 | . | . | . | . |
| Do vs O+D | 0 | 0 | 1.197 | . | 1.197 | . | . | . |
| Do vs P | 0 | 0 | 1.2995 | . | 1.2995 | . | . | . |
| Do vs P+D | 0 | 0 | 1.5811 | . | 1.5811 | . | . | . |
| Do vs R | 0 | 0 | 0.7162 | . | 0.7162 | . | . | . |
| Do vs R+D | 0 | 0 | 1.098 | . | 1.098 | . | . | . |
| Do vs T | 0 | 0 | 1.1021 | . | 1.1021 | . | . | . |
| Do vs T+D | 0 | 0 | 1.6377 | . | 1.6377 | . | . | . |
| Do+D vs G | 0 | 0 | 0.5008 | . | 0.5008 | . | . | . |
| Do+D vs G+D | 0 | 0 | 0.5438 | . | 0.5438 | . | . | . |
| Do+D vs O | 0 | 0 | 0.459 | . | 0.459 | . | . | . |
| Do+D vs O+D | 0 | 0 | 0.6019 | . | 0.6019 | . | . | . |
| Do+D vs P | 0 | 0 | 0.6535 | . | 0.6535 | . | . | . |
| Do+D vs P+D | 0 | 0 | 0.7951 | . | 0.7951 | . | . | . |
| Do+D vs R | 0 | 0 | 0.3601 | . | 0.3601 | . | . | . |
| Do+D vs R+D | 0 | 0 | 0.5521 | . | 0.5521 | . | . | . |
| Do+D vs T | 0 | 0 | 0.5542 | . | 0.5542 | . | . | . |
| Do+D vs T+D | 0 | 0 | 0.8236 | . | 0.8236 | . | . | . |
| G vs G+D | 1 | 0.77 | 1.0858 | 0.9703 | 1.5975 | 0.6074 | -1.26 | 0.2065 |
| G vs O | 1 | 0.51 | 0.9164 | 1.1728 | 0.7123 | 1.6465 | 1.26 | 0.2065 |
| G vs O+D | 0 | 0 | 1.2018 | . | 1.2018 | . | . | . |
| G vs P | 0 | 0 | 1.3048 | . | 1.3048 | . | . | . |
| G vs P+D | 0 | 0 | 1.5874 | . | 1.5874 | . | . | . |
| G vs R | 0 | 0 | 0.7191 | . | 0.7191 | . | . | . |
| G vs R+D | 0 | 0 | 1.1024 | . | 1.1024 | . | . | . |
| G vs T | 0 | 0 | 1.1065 | . | 1.1065 | . | . | . |
| G vs T+D | 0 | 0 | 1.6443 | . | 1.6443 | . | . | . |
| G+D vs O | 0 | 0 | 0.844 | . | 0.844 | . | . | . |
| G+D vs O+D | 1 | 0.48 | 1.1068 | 1.0715 | 1.1407 | 0.9393 | -0.26 | 0.7948 |
| G+D vs P | 0 | 0 | 1.2016 | . | 1.2016 | . | . | . |
| G+D vs P+D | 2 | 0.71 | 1.462 | 1.4085 | 1.5987 | 0.881 | -0.52 | 0.6042 |
| G+D vs R | 0 | 0 | 0.6622 | . | 0.6622 | . | . | . |
| G+D vs R+D | 1 | 1 | 1.0153 | 1.0153 | . | . | . | . |
| G+D vs T | 0 | 0 | 1.0191 | . | 1.0191 | . | . | . |
| G+D vs T+D | 0 | 0 | 1.5143 | . | 1.5143 | . | . | . |
| O+D vs O | 2 | 0.82 | 0.7625 | 0.6986 | 1.1502 | 0.6074 | -1.26 | 0.2065 |
| P vs O | 3 | 1 | 0.7024 | 0.7024 | . | . | . | . |
| P+D vs O | 0 | 0 | 0.5773 | . | 0.5773 | . | . | . |
| R vs O | 0 | 0 | 1.2745 | . | 1.2745 | . | . | . |
| R+D vs O | 0 | 0 | 0.8313 | . | 0.8313 | . | . | . |
| T vs O | 1 | 1 | 0.8282 | 0.8282 | . | . | . | . |
| T+D vs O | 0 | 0 | 0.5573 | . | 0.5573 | . | . | . |
| O+D vs P | 0 | 0 | 1.0857 | . | 1.0857 | . | . | . |
| O+D vs P+D | 2 | 0.71 | 1.3209 | 1.3706 | 1.2075 | 1.1351 | 0.52 | 0.6042 |
| O+D vs R | 0 | 0 | 0.5983 | . | 0.5983 | . | . | . |
| O+D vs R+D | 0 | 0 | 0.9173 | . | 0.9173 | . | . | . |
| O+D vs T | 0 | 0 | 0.9207 | . | 0.9207 | . | . | . |
| O+D vs T+D | 0 | 0 | 1.3682 | . | 1.3682 | . | . | . |
| P vs P+D | 0 | 0 | 1.2167 | . | 1.2167 | . | . | . |
| P vs R | 0 | 0 | 0.5511 | . | 0.5511 | . | . | . |
| P vs R+D | 0 | 0 | 0.8449 | . | 0.8449 | . | . | . |
| P vs T | 0 | 0 | 0.8481 | . | 0.8481 | . | . | . |
| P vs T+D | 0 | 0 | 1.2602 | . | 1.2602 | . | . | . |
| P+D vs R | 0 | 0 | 0.453 | . | 0.453 | . | . | . |
| P+D vs R+D | 0 | 0 | 0.6945 | . | 0.6945 | . | . | . |
| P+D vs T | 0 | 0 | 0.6971 | . | 0.6971 | . | . | . |
| P+D vs T+D | 0 | 0 | 1.0358 | . | 1.0358 | . | . | . |
| R vs R+D | 1 | 1 | 1.5331 | 1.5331 | . | . | . | . |
| R vs T | 0 | 0 | 1.5388 | . | 1.5388 | . | . | . |
| R vs T+D | 0 | 0 | 2.2867 | . | 2.2867 | . | . | . |
| R+D vs T | 0 | 0 | 1.0037 | . | 1.0037 | . | . | . |
| R+D vs T+D | 0 | 0 | 1.4915 | . | 1.4915 | . | . | . |
| T vs T+D | 1 | 1 | 1.486 | 1.486 | . | . | . | . |

Netsplit of delayed vomiting

| comparison | k | prop | nma | direct | indir. | RoR | z | p-value |
| --- | --- | --- | --- | --- | --- | --- | --- | --- |
| Do vs Do+D | 1 | 1 | 2.3331 | 2.3331 | . | . | . | . |
| Do vs G | 0 | 0 | 1.3649 | . | 1.3649 | . | . | . |
| Do vs G+D | 0 | 0 | 1.3652 | . | 1.3652 | . | . | . |
| Do vs O | 1 | 1 | 1.2692 | 1.2692 | . | . | . | . |
| Do vs O+D | 0 | 0 | 1.5366 | . | 1.5366 | . | . | . |
| Do vs P | 0 | 0 | 1.8615 | . | 1.8615 | . | . | . |
| Do vs P+D | 0 | 0 | 2.0795 | . | 2.0795 | . | . | . |
| Do vs R | 0 | 0 | 0.8166 | . | 0.8166 | . | . | . |
| Do vs R+D | 0 | 0 | 1.3283 | . | 1.3283 | . | . | . |
| Do vs T | 0 | 0 | 1.296 | . | 1.296 | . | . | . |
| Do vs T+D | 0 | 0 | 2.0562 | . | 2.0562 | . | . | . |
| Do+D vs G | 0 | 0 | 0.585 | . | 0.585 | . | . | . |
| Do+D vs G+D | 0 | 0 | 0.5852 | . | 0.5852 | . | . | . |
| Do+D vs O | 0 | 0 | 0.544 | . | 0.544 | . | . | . |
| Do+D vs O+D | 0 | 0 | 0.6586 | . | 0.6586 | . | . | . |
| Do+D vs P | 0 | 0 | 0.7979 | . | 0.7979 | . | . | . |
| Do+D vs P+D | 0 | 0 | 0.8913 | . | 0.8913 | . | . | . |
| Do+D vs R | 0 | 0 | 0.35 | . | 0.35 | . | . | . |
| Do+D vs R+D | 0 | 0 | 0.5694 | . | 0.5694 | . | . | . |
| Do+D vs T | 0 | 0 | 0.5555 | . | 0.5555 | . | . | . |
| Do+D vs T+D | 0 | 0 | 0.8813 | . | 0.8813 | . | . | . |
| G vs G+D | 1 | 0.63 | 1.0003 | 0.9296 | 1.1305 | 0.8223 | -0.57 | 0.5661 |
| G vs O | 2 | 0.57 | 0.9299 | 1.0922 | 0.7495 | 1.4572 | 1.37 | 0.17 |
| G vs O+D | 0 | 0 | 1.1258 | . | 1.1258 | . | . | . |
| G vs P | 1 | 0.63 | 1.3639 | 1.1163 | 1.9148 | 0.583 | -1.5 | 0.1334 |
| G vs P+D | 0 | 0 | 1.5236 | . | 1.5236 | . | . | . |
| G vs R | 0 | 0 | 0.5983 | . | 0.5983 | . | . | . |
| G vs R+D | 0 | 0 | 0.9732 | . | 0.9732 | . | . | . |
| G vs T | 1 | 0.46 | 0.9495 | 1.1438 | 0.8123 | 1.4081 | 0.92 | 0.3586 |
| G vs T+D | 0 | 0 | 1.5065 | . | 1.5065 | . | . | . |
| G+D vs O | 0 | 0 | 0.9297 | . | 0.9297 | . | . | . |
| G+D vs O+D | 1 | 0.42 | 1.1255 | 0.9903 | 1.2328 | 0.8033 | -0.92 | 0.3559 |
| G+D vs P | 0 | 0 | 1.3635 | . | 1.3635 | . | . | . |
| G+D vs P+D | 2 | 0.61 | 1.5231 | 1.5974 | 1.4138 | 1.1299 | 0.52 | 0.6036 |
| G+D vs R | 0 | 0 | 0.5981 | . | 0.5981 | . | . | . |
| G+D vs R+D | 1 | 1 | 0.973 | 0.973 | . | . | . | . |
| G+D vs T | 0 | 0 | 0.9493 | . | 0.9493 | . | . | . |
| G+D vs T+D | 0 | 0 | 1.5061 | . | 1.5061 | . | . | . |
| O+D vs O | 2 | 0.71 | 0.826 | 0.7801 | 0.9487 | 0.8223 | -0.57 | 0.5661 |
| P vs O | 3 | 0.54 | 0.6818 | 0.533 | 0.9142 | 0.583 | -1.5 | 0.1334 |
| P+D vs O | 0 | 0 | 0.6104 | . | 0.6104 | . | . | . |
| R vs O | 0 | 0 | 1.5543 | . | 1.5543 | . | . | . |
| R+D vs O | 0 | 0 | 0.9555 | . | 0.9555 | . | . | . |
| T vs O | 2 | 0.93 | 0.9794 | 1.0256 | 0.5469 | 1.8755 | 1.06 | 0.2878 |
| T+D vs O | 0 | 0 | 0.6173 | . | 0.6173 | . | . | . |
| O+D vs P | 0 | 0 | 1.2114 | . | 1.2114 | . | . | . |
| O+D vs P+D | 4 | 0.84 | 1.3533 | 1.3269 | 1.4992 | 0.8851 | -0.52 | 0.6036 |
| O+D vs R | 0 | 0 | 0.5314 | . | 0.5314 | . | . | . |
| O+D vs R+D | 0 | 0 | 0.8645 | . | 0.8645 | . | . | . |
| O+D vs T | 0 | 0 | 0.8434 | . | 0.8434 | . | . | . |
| O+D vs T+D | 0 | 0 | 1.3381 | . | 1.3381 | . | . | . |
| P vs P+D | 0 | 0 | 1.1171 | . | 1.1171 | . | . | . |
| P vs R | 0 | 0 | 0.4387 | . | 0.4387 | . | . | . |
| P vs R+D | 0 | 0 | 0.7136 | . | 0.7136 | . | . | . |
| P vs T | 0 | 0 | 0.6962 | . | 0.6962 | . | . | . |
| P vs T+D | 0 | 0 | 1.1046 | . | 1.1046 | . | . | . |
| P+D vs R | 0 | 0 | 0.3927 | . | 0.3927 | . | . | . |
| P+D vs R+D | 0 | 0 | 0.6388 | . | 0.6388 | . | . | . |
| P+D vs T | 0 | 0 | 0.6232 | . | 0.6232 | . | . | . |
| P+D vs T+D | 0 | 0 | 0.9888 | . | 0.9888 | . | . | . |
| R vs R+D | 1 | 1 | 1.6267 | 1.6267 | . | . | . | . |
| R vs T | 0 | 0 | 1.5871 | . | 1.5871 | . | . | . |
| R vs T+D | 0 | 0 | 2.5181 | . | 2.5181 | . | . | . |
| R+D vs T | 0 | 0 | 0.9756 | . | 0.9756 | . | . | . |
| R+D vs T+D | 0 | 0 | 1.5479 | . | 1.5479 | . | . | . |
| T vs T+D | 2 | 1 | 1.5866 | 1.5866 | . | . | . | . |

Netsplit of delayed complete control

| comparison | k | prop | nma | direct | indir. | RoR | z | p-value |
| --- | --- | --- | --- | --- | --- | --- | --- | --- |
| G vs G+D | 1 | 1 | 1.1161 | 1.1161 | . | . | . | . |
| G vs O | 0 | 0 | 1.8219 | . | 1.8219 | . | . | . |
| G vs O+D | 0 | 0 | 1.1188 | . | 1.1188 | . | . | . |
| G vs P | 0 | 0 | 1.2725 | . | 1.2725 | . | . | . |
| G vs P+D | 0 | 0 | 0.8399 | . | 0.8399 | . | . | . |
| G vs R+D | 0 | 0 | 1.0316 | . | 1.0316 | . | . | . |
| G+D vs O | 0 | 0 | 1.6325 | . | 1.6325 | . | . | . |
| G+D vs O+D | 1 | 0.66 | 1.0025 | 0.9393 | 1.1379 | 0.8255 | -1 | 0.3185 |
| G+D vs P | 0 | 0 | 1.1401 | . | 1.1401 | . | . | . |
| G+D vs P+D | 1 | 0.68 | 0.7525 | 0.8004 | 0.6607 | 1.2114 | 1 | 0.3185 |
| G+D vs R+D | 1 | 1 | 0.9243 | 0.9243 | . | . | . | . |
| O+D vs O | 2 | 1 | 1.6284 | 1.6284 | . | . | . | . |
| P vs O | 1 | 1 | 1.4318 | 1.4318 | . | . | . | . |
| P+D vs O | 0 | 0 | 2.1693 | . | 2.1693 | . | . | . |
| R+D vs O | 0 | 0 | 1.7661 | . | 1.7661 | . | . | . |
| O+D vs P | 0 | 0 | 1.1373 | . | 1.1373 | . | . | . |
| O+D vs P+D | 2 | 0.66 | 0.7507 | 0.7034 | 0.8521 | 0.8255 | -1 | 0.3185 |
| O+D vs R+D | 0 | 0 | 0.922 | . | 0.922 | . | . | . |
| P vs P+D | 0 | 0 | 0.66 | . | 0.66 | . | . | . |
| P vs R+D | 0 | 0 | 0.8107 | . | 0.8107 | . | . | . |
| P+D vs R+D | 0 | 0 | 1.2283 | . | 1.2283 | . | . | . |
