# Supplementary figures and images for: Comparative efficacy of 5-hydroxytryptamine-3 (5-HT3) receptor antagonists with or without dexamethasone for prevention of chemotherapy-induced nausea and vomiting following highly emetogenic chemotherapy (HEC): a network meta-analysis
Source: PeerJ. 2026 Apr 2;14:e21047. doi: 10.7717/peerj.21047 (PMC13050518; doi:10.7717/peerj.21047)

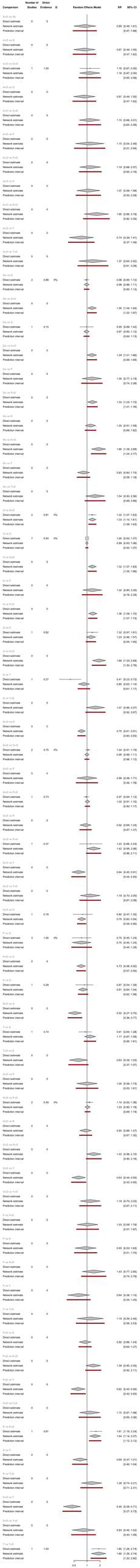

Supplement: Supplemental Information 9 [file peerj-14-21047-s009.pdf]

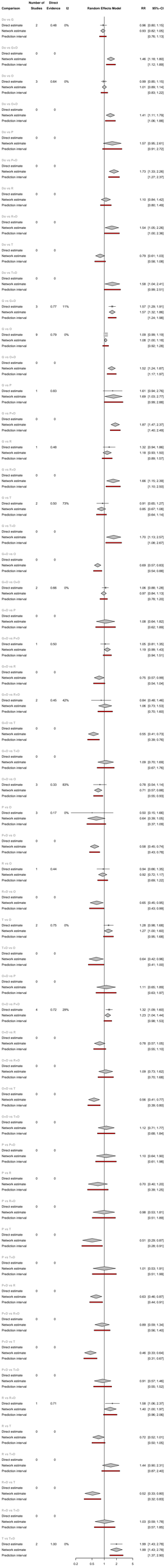

Supplement: Supplemental Information 10 [file peerj-14-21047-s010.pdf]

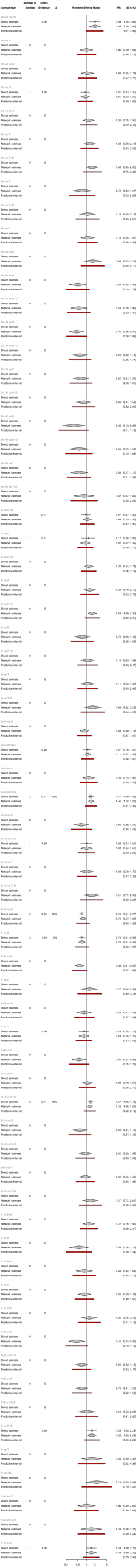

Supplement: Supplemental Information 12 [file peerj-14-21047-s012.pdf]

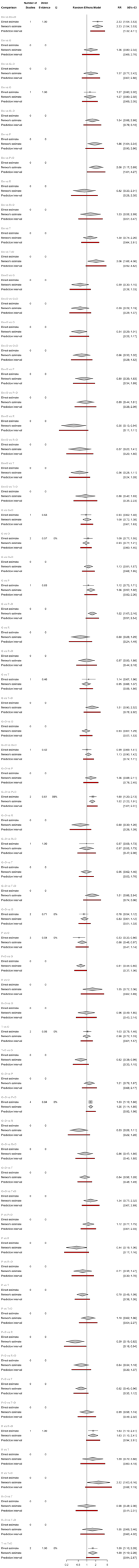

Supplement: Supplemental Information 13 [file peerj-14-21047-s013.pdf]

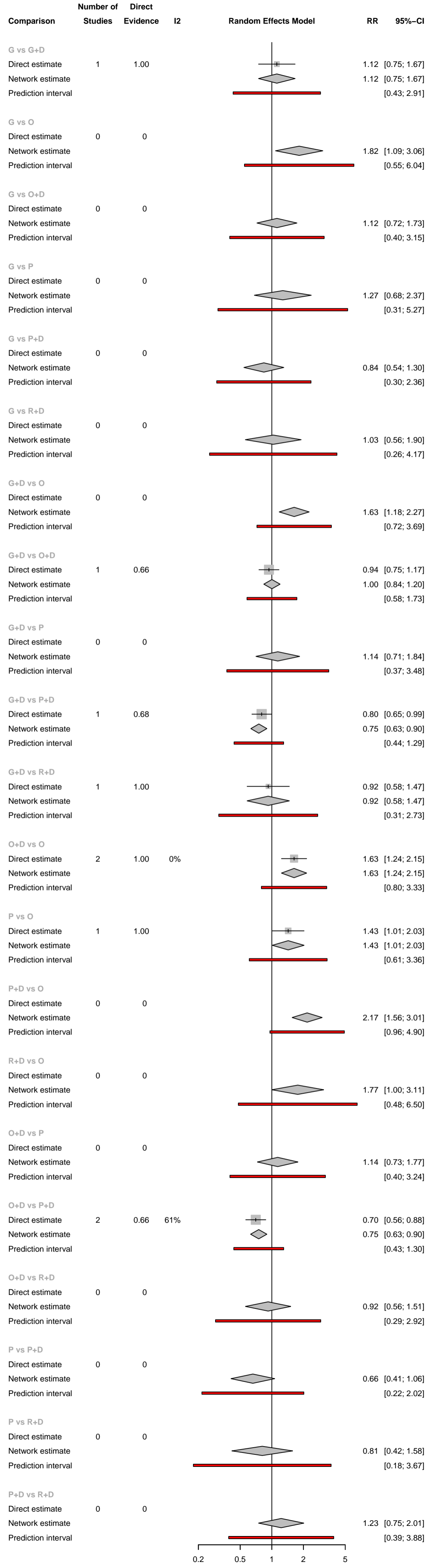

Supplement: Supplemental Information 14 [file peerj-14-21047-s014.pdf]
