# Supplementary material for: Comparative efficacy of 5-hydroxytryptamine-3 (5-HT3) receptor antagonists with or without dexamethasone for prevention of chemotherapy-induced nausea and vomiting following highly emetogenic chemotherapy (HEC): a network meta-analysis
Source: PeerJ. 2026 Apr 2;14:e21047. doi: 10.7717/peerj.21047 (PMC13050518; doi:10.7717/peerj.21047)
Supplement: Supplemental Information 11 [file peerj-14-21047-s011.pdf]

Comparison	Number of Studies	Direct Evidence	I2	RR	95%-CI
**G vs G+D**					
Direct estimate	1	0.36		0.80	[0.61; 1.04]
Network estimate				0.86	[0.74; 1.01]
Prediction interval					[0.73; 1.02]
**G vs O**					
Direct estimate	8	0.94	0%	0.96	[0.89; 1.03]
Network estimate				0.95	[0.88; 1.02]
Prediction interval					[0.88; 1.03]
**G vs O+D**					
Direct estimate	0	0			
Network estimate				0.87	[0.74; 1.01]
Prediction interval					[0.73; 1.02]
**G vs P**					
Direct estimate	0	0			
Network estimate				0.93	[0.83; 1.03]
Prediction interval					[0.83; 1.04]
**G vs P+D**					
Direct estimate	0	0			
Network estimate				0.83	[0.71; 0.98]
Prediction interval					[0.69; 1.00]
**G vs R**					
Direct estimate	0	0			
Network estimate				1.14	[0.83; 1.57]
Prediction interval					[0.81; 1.61]
**G vs R+D**					
Direct estimate	0	0			
Network estimate				0.91	[0.71; 1.16]
Prediction interval					[0.69; 1.19]
**G vs T**					
Direct estimate	1	0.86		1.16	[0.92; 1.47]
Network estimate				1.12	[0.90; 1.39]
Prediction interval					[0.88; 1.42]
**G vs T+D**					
Direct estimate	0	0			
Network estimate				0.73	[0.54; 0.98]
Prediction interval					[0.52; 1.01]
**G+D vs O**					
Direct estimate	0	0			
Network estimate				1.10	[0.95; 1.28]
Prediction interval					[0.93; 1.30]
**G+D vs O+D**					
Direct estimate	2	0.74	0%	0.99	[0.92; 1.06]
Network estimate				1.01	[0.95; 1.07]
Prediction interval					[0.94; 1.08]
**G+D vs P**					
Direct estimate	0	0			
Network estimate				1.08	[0.91; 1.27]
Prediction interval					[0.89; 1.29]
**G+D vs P+D**					
Direct estimate	1	0.74		0.98	[0.91; 1.05]
Network estimate				0.96	[0.91; 1.03]
Prediction interval					[0.90; 1.03]
**G+D vs R**					
Direct estimate	0	0			
Network estimate				1.33	[1.01; 1.74]
Prediction interval					[0.98; 1.79]
**G+D vs R+D**					
Direct estimate	2	1.00	17%	1.05	[0.87; 1.27]
Network estimate				1.05	[0.87; 1.27]
Prediction interval					[0.85; 1.30]
**G+D vs T**					
Direct estimate	0	0			
Network estimate				1.30	[1.00; 1.69]
Prediction interval					[0.98; 1.73]
**G+D vs T+D**					
Direct estimate	0	0			
Network estimate				0.84	[0.60; 1.18]
Prediction interval					[0.59; 1.22]
**O+D vs O**					
Direct estimate	2	0.73	38%	1.06	[0.89; 1.26]
Network estimate				1.09	[0.95; 1.27]
Prediction interval					[0.93; 1.28]
**P vs O**					
Direct estimate	3	1.00	0%	1.03	[0.95; 1.10]
Network estimate				1.03	[0.95; 1.10]
Prediction interval					[0.95; 1.11]
**P+D vs O**					
Direct estimate	0	0			
Network estimate				1.14	[0.98; 1.34]
Prediction interval					[0.96; 1.36]
**R vs O**					
Direct estimate	0	0			
Network estimate				0.83	[0.61; 1.14]
Prediction interval					[0.59; 1.17]
**R+D vs O**					
Direct estimate	0	0			
Network estimate				1.05	[0.82; 1.34]
Prediction interval					[0.80; 1.37]
**T vs O**					
Direct estimate	1	0.83		0.88	[0.69; 1.12]
Network estimate				0.85	[0.68; 1.06]
Prediction interval					[0.67; 1.08]
**T+D vs O**					
Direct estimate	0	0			
Network estimate				1.31	[0.97; 1.77]
Prediction interval					[0.94; 1.82]
**O+D vs P**					
Direct estimate	0	0			
Network estimate				1.07	[0.91; 1.26]
Prediction interval					[0.89; 1.28]
**O+D vs P+D**					
Direct estimate	2	0.49	0%	0.93	[0.84; 1.03]
Network estimate				0.96	[0.89; 1.03]
Prediction interval					[0.89; 1.03]
**O+D vs R**					
Direct estimate	0	0			
Network estimate				1.32	[1.00; 1.74]
Prediction interval					[0.97; 1.78]
**O+D vs R+D**					
Direct estimate	0	0			
Network estimate				1.04	[0.85; 1.28]
Prediction interval					[0.84; 1.30]
**O+D vs T**					
Direct estimate	0	0			
Network estimate				1.29	[0.99; 1.67]
Prediction interval					[0.97; 1.71]
**O+D vs T+D**					
Direct estimate	0	0			
Network estimate				0.84	[0.60; 1.17]
Prediction interval					[0.58; 1.20]
**P vs P+D**					
Direct estimate	0	0			
Network estimate				0.90	[0.75; 1.07]
Prediction interval					[0.74; 1.09]
**P vs R**					
Direct estimate	0	0			
Network estimate				1.23	[0.90; 1.70]
Prediction interval					[0.87; 1.75]
**P vs R+D**					
Direct estimate	0	0			
Network estimate				0.98	[0.76; 1.26]
Prediction interval					[0.74; 1.29]
**P vs T**					
Direct estimate	0	0			
Network estimate				1.21	[0.96; 1.52]
Prediction interval					[0.94; 1.56]
**P vs T+D**					
Direct estimate	0	0			
Network estimate				0.78	[0.57; 1.07]
Prediction interval					[0.56; 1.10]
**P+D vs R**					
Direct estimate	0	0			
Network estimate				1.37	[1.04; 1.82]
Prediction interval					[1.01; 1.87]
**P+D vs R+D**					
Direct estimate	0	0			
Network estimate				1.09	[0.89; 1.33]
Prediction interval					[0.87; 1.36]
**P+D vs T**					
Direct estimate	0	0			
Network estimate				1.35	[1.03; 1.76]
Prediction interval					[1.01; 1.81]
**P+D vs T+D**					
Direct estimate	0	0			
Network estimate				0.87	[0.62; 1.23]
Prediction interval					[0.60; 1.27]
**R vs R+D**					
Direct estimate	1	1.00		0.79	[0.65; 0.96]
Network estimate				0.79	[0.65; 0.96]
Prediction interval					[0.64; 0.98]
**R vs T**					
Direct estimate	0	0			
Network estimate				0.98	[0.67; 1.43]
Prediction interval					[0.65; 1.48]
**R vs T+D**					
Direct estimate	0	0			
Network estimate				0.64	[0.41; 0.98]
Prediction interval					[0.40; 1.02]
**R+D vs T**					
Direct estimate	0	0			
Network estimate				1.24	[0.89; 1.71]
Prediction interval					[0.87; 1.77]
**R+D vs T+D**					
Direct estimate	0	0			
Network estimate				0.80	[0.55; 1.18]
Prediction interval					[0.53; 1.22]
**T vs T+D**					
Direct estimate	1	1.00		0.65	[0.53; 0.80]
Network estimate				0.65	[0.53; 0.80]
Prediction interval					[0.52; 0.81]
