# Supplementary material for: Comparative efficacy of 5-hydroxytryptamine-3 (5-HT3) receptor antagonists with or without dexamethasone for prevention of chemotherapy-induced nausea and vomiting following highly emetogenic chemotherapy (HEC): a network meta-analysis
Source: PeerJ. 2026 Apr 2;14:e21047. doi: 10.7717/peerj.21047 (PMC13050518; doi:10.7717/peerj.21047)
Supplement: Supplemental Information 15 [file peerj-14-21047-s015.docx]

**Supplement 11 Transitivity analyses**

**The transitivity of mean age**

|  | Df | Sum Sq | Mean Sq | F value | Pr(>F) |
| --- | --- | --- | --- | --- | --- |
| Arm | 12 | 2614 | 217.8 | 1.3 | 0.239 |
| Residuals | 70 | 11731 | 167.6 |  |  |


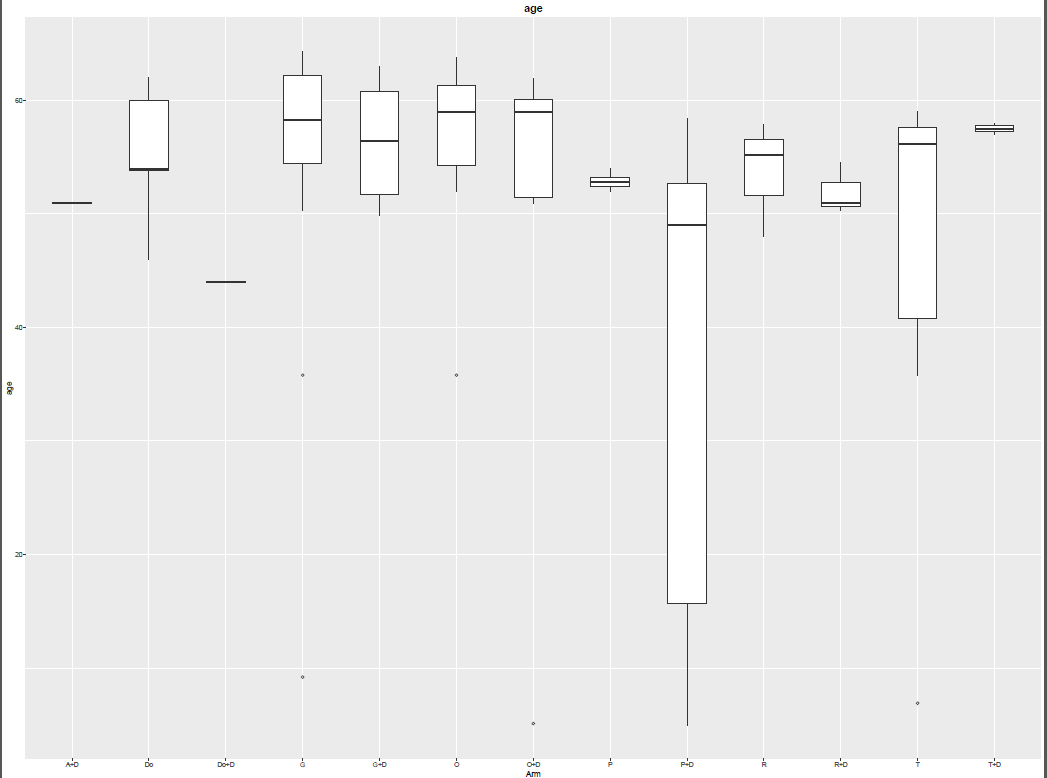


**The transitivity of publication years**

|  | Df | Sum Sq | Mean Sq | F value | Pr(>F) |
| --- | --- | --- | --- | --- | --- |
| Arm | 12 | 1976 | 164.93 | 4.754 | 1.25e-05 *** |
| Residuals | 70 | 2428 | 34.69 |  |  |

**
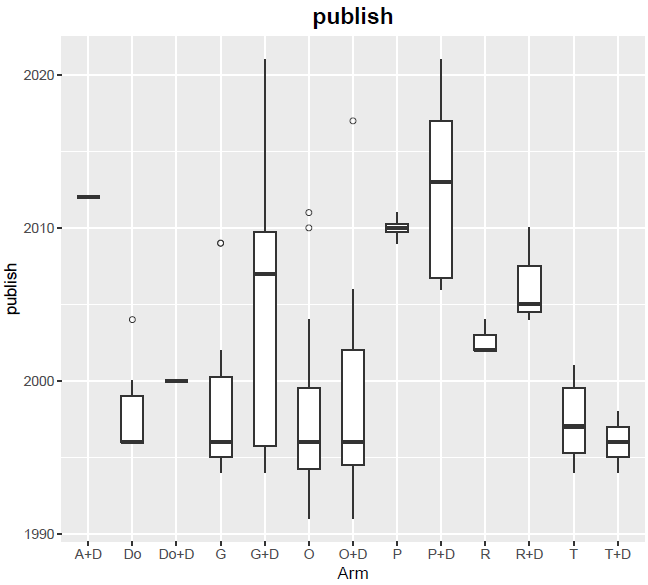
**

**Meta-regression of acute nausea**

**Quantiles for each variable:**

**2.5% 25% 50% 75% 97.5%**

**d.G.Do -0.190663 -0.09986 -0.045885 0.007832 0.10748**

**d.G.GD -0.474654 -0.34423 -0.275089 -0.208465 -0.05652**

**d.G.O -0.312708 -0.18425 -0.115163 -0.046281 0.09843**

**d.G.R -0.475498 -0.33049 -0.242949 -0.155655 0.02549**

**d.G.T -0.117276 0.08305 0.194233 0.310105 0.49538**

**d.GD.AD -0.451039 -0.07175 0.116514 0.323044 0.69109**

**d.GD.OD -0.115331 -0.03530 0.008982 0.052145 0.13540**

**d.GD.PD -0.178285 -0.07742 -0.026016 0.018588 0.11638**

**d.GD.RD -0.777606 -0.54456 -0.399188 -0.256978 -0.02020**

**d.O.P -1.088652 -0.71656 -0.477637 -0.259415 0.10312**

**d.T.TD -1.059011 -0.75510 -0.614044 -0.466103 -0.21464**

**sd.d 0.001958 0.01573 0.033536 0.061274 0.13027**

**B -0.185135 0.14930 0.305889 0.471404 0.77597**

**Meta-regression of acute vomiting**

**Quantiles for each variable:**

**2.5% 25% 50% 75% 97.5%**

**d.Do.DoD -1.24502 -0.8587 -0.68375 -0.507564 -0.1271**

**d.G.GD -0.54748 -0.2144 -0.08245 0.039840 0.3377**

**d.G.O -1.10815 -0.2283 0.10297 0.457933 1.3089**

**d.GD.OD -0.46139 -0.2035 -0.10043 -0.004705 0.2088**

**d.GD.PD -0.73041 -0.4755 -0.37830 -0.294422 -0.1091**

**d.GD.RD -0.59113 -0.1889 -0.01166 0.163043 0.5464**

**d.O.Do -0.62358 -0.2332 -0.08730 0.058794 0.4364**

**d.O.P -1.14956 -0.5632 -0.33354 -0.081682 0.4922**

**d.O.T -1.39450 -0.5485 -0.21783 0.127230 0.9684**

**d.RD.R -0.07863 0.2790 0.42089 0.574634 0.9324**

**d.T.TD -0.94069 -0.5525 -0.39115 -0.236156 0.1481**

**sd.d 0.06844 0.1427 0.19402 0.264897 0.4582**

**B -2.08445 -0.5955 -0.03336 0.465314 2.0673**

**Meta-regression of acute** **complete control**

**Quantiles for each variable:**

**2.5% 25% 50% 75% 97.5%**

**d.Do.DoD -1.25516 -0.8651 -0.694371 -0.51394 -0.1130**

**d.G.GD -0.54427 -0.2208 -0.090351 0.03487 0.3279**

**d.G.O -1.38351 -0.2498 0.082024 0.41706 1.2561**

**d.GD.OD -0.48391 -0.2096 -0.105125 -0.01347 0.1987**

**d.GD.PD -0.73532 -0.4777 -0.378146 -0.29108 -0.1144**

**d.GD.RD -0.59313 -0.1979 -0.009406 0.17539 0.5950**

**d.O.Do -0.60786 -0.2339 -0.091135 0.05212 0.4391**

**d.O.P -1.32763 -0.5817 -0.348140 -0.10693 0.4595**

**d.O.T -1.39051 -0.5260 -0.200630 0.12922 1.2192**

**d.RD.R -0.11775 0.2773 0.430401 0.58354 0.9806**

**d.T.TD -0.88831 -0.5456 -0.395592 -0.23856 0.1463**

**sd.d 0.06137 0.1440 0.197266 0.26501 0.4743**

**B -2.15896 -0.5307 -0.025699 0.50472 2.6443**

**Meta-regression of delayed nausea**

**Quantiles for each variable:**

**2.5% 25% 50% 75% 97.5%**

**d.Do.DoD -1.22598 -0.8584 -0.68170 -0.51598 -0.1304**

**d.G.GD -0.55511 -0.2184 -0.08618 0.03441 0.3312**

**d.G.O -1.39505 -0.2790 0.04940 0.38620 1.4227**

**d.GD.OD -0.45972 -0.2043 -0.10317 -0.01129 0.1822**

**d.GD.PD -0.72309 -0.4723 -0.37714 -0.28787 -0.1199**

**d.GD.RD -0.56801 -0.1961 -0.01842 0.16060 0.5553**

**d.O.Do -0.58642 -0.2310 -0.09342 0.05106 0.4348**

**d.O.P -1.30696 -0.6025 -0.36904 -0.12725 0.5549**

**d.O.T -1.49312 -0.4893 -0.16535 0.15789 1.2203**

**d.RD.R -0.09981 0.2848 0.42676 0.57249 0.9382**

**d.T.TD -0.91415 -0.5448 -0.39871 -0.23639 0.1360**

**sd.d 0.06210 0.1431 0.19655 0.26021 0.4604**

**B -2.38101 -0.4762 0.03200 0.57503 2.5875**

**Meta-regression of delayed vomiting**

**Quantiles for each variable:**

**2.5% 25% 50% 75% 97.5%**

**d.Do.DoD -1.24606 -0.8601 -0.69878 -0.532642 -0.1284**

**d.G.GD -0.56909 -0.2130 -0.08140 0.037087 0.3416**

**d.G.O -1.22480 -0.2442 0.10698 0.456255 1.6567**

**d.GD.OD -0.49128 -0.2088 -0.10141 -0.007815 0.1918**

**d.GD.PD -0.71761 -0.4752 -0.37585 -0.287629 -0.1054**

**d.GD.RD -0.60531 -0.1976 -0.02253 0.160945 0.5573**

**d.O.Do -0.59523 -0.2337 -0.09124 0.049937 0.4157**

**d.O.P -1.22873 -0.5671 -0.33574 -0.083097 0.6648**

**d.O.T -1.67457 -0.5744 -0.22284 0.127145 1.0662**

**d.RD.R -0.08617 0.2736 0.42235 0.576965 0.9622**

**d.T.TD -0.92395 -0.5427 -0.38304 -0.227574 0.1719**

**sd.d 0.06028 0.1425 0.19433 0.264404 0.4898**

**B -2.82018 -0.5997 -0.03352 0.485521 2.2082**

**Meta-regression of delayed complete control**

**Quantiles for each variable:**

**2.5% 25% 50% 75% 97.5%**

**d.GD.G -0.424665 -0.05466 0.1135797 0.28253 0.6627**

**d.GD.RD -0.525956 -0.12731 0.0640261 0.26243 0.7024**

**d.O.P -0.525844 0.12887 0.3806930 0.61492 1.5949**

**d.OD.GD -0.369105 -0.07651 0.0002081 0.08518 0.3640**

**d.OD.O -1.638095 -0.72153 -0.4774444 -0.20558 1.0512**

**d.OD.PD -0.013477 0.20918 0.2832226 0.36864 0.6087**

**sd.d 0.006283 0.07652 0.1452432 0.24198 0.4718**

**B -3.204261 -0.47145 -0.0199824 0.40646 2.2831**
