# Supplementary material for: Comparative efficacy of 5-hydroxytryptamine-3 (5-HT3) receptor antagonists with or without dexamethasone for prevention of chemotherapy-induced nausea and vomiting following highly emetogenic chemotherapy (HEC): a network meta-analysis
Source: PeerJ. 2026 Apr 2;14:e21047. doi: 10.7717/peerj.21047 (PMC13050518; doi:10.7717/peerj.21047)
Supplement: Supplemental Information 16 [file peerj-14-21047-s016.docx]

Spplement 15 Funnel plot and the value of Egger’s test of outcomes


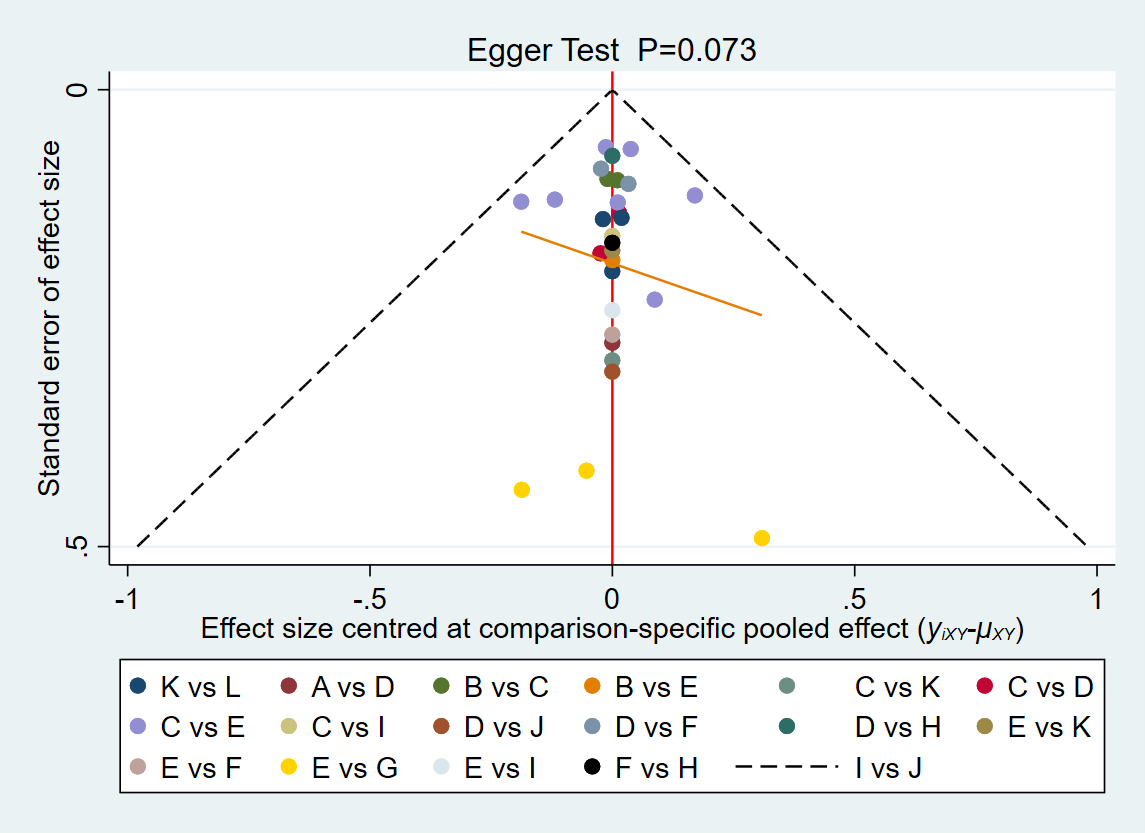


A= Azasetron+ Dexamethasone; B= Dolasetron; C= Granisetron; D= Granisetron+ Dexamethasone

E=Ondansetron; F=Ondansetron+Dexamethasone; G=Palonosetron; H=Palonosetron+ Dexamethasone; I=Ramosetron; J=Ramosetron+Dexamethason; K= Tropisetron; L= Tropisetron+ Dexamethasone

Funnel plot and the value of Egger’s test of acute nausea


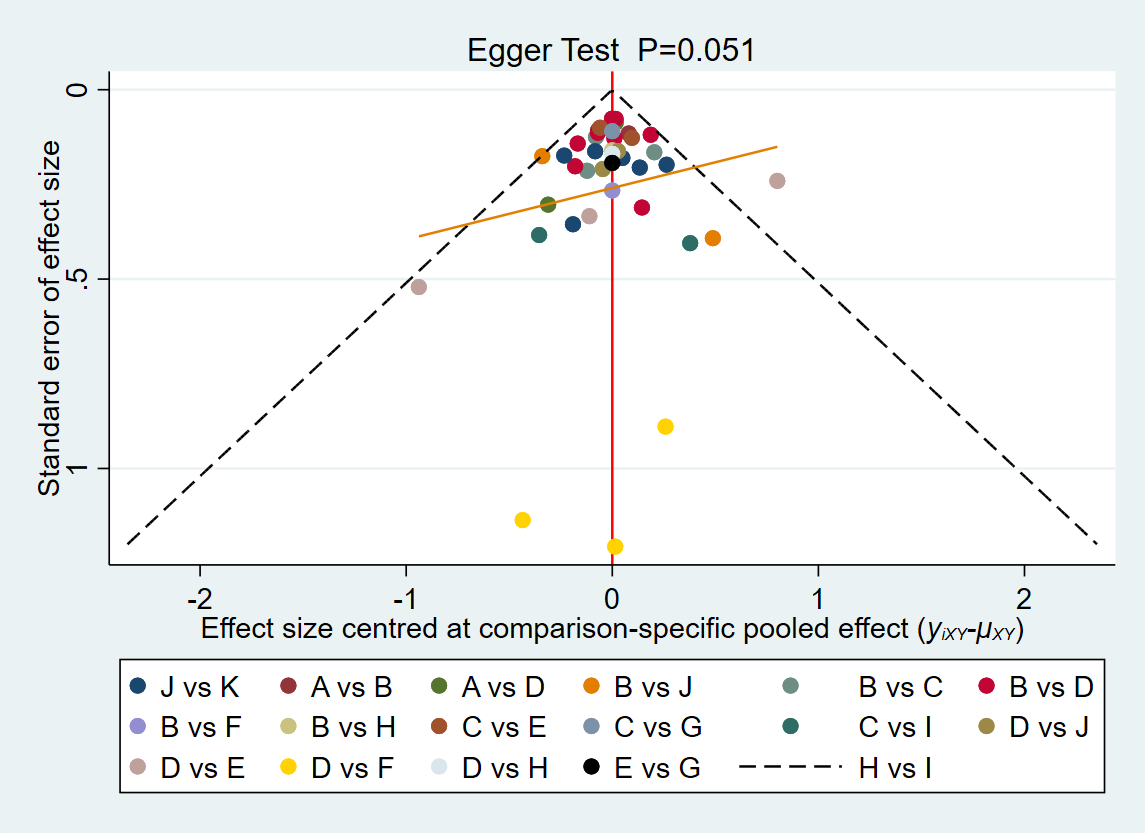


A= Dolasetron; B= Granisetron; C= Granisetron+ Dexamethasone; D= Ondansetron; E= Ondansetron+Dexamethasone; F= Palonosetro; G=Palonosetron+Dexamethasone; H=Ramosetrone; I= Ramosetron+Dexamethaso; J= Tropisetron; K= Tropisetron+ Dexamethasone

Funnel plot and the value of Egger’s test of acute vomiting


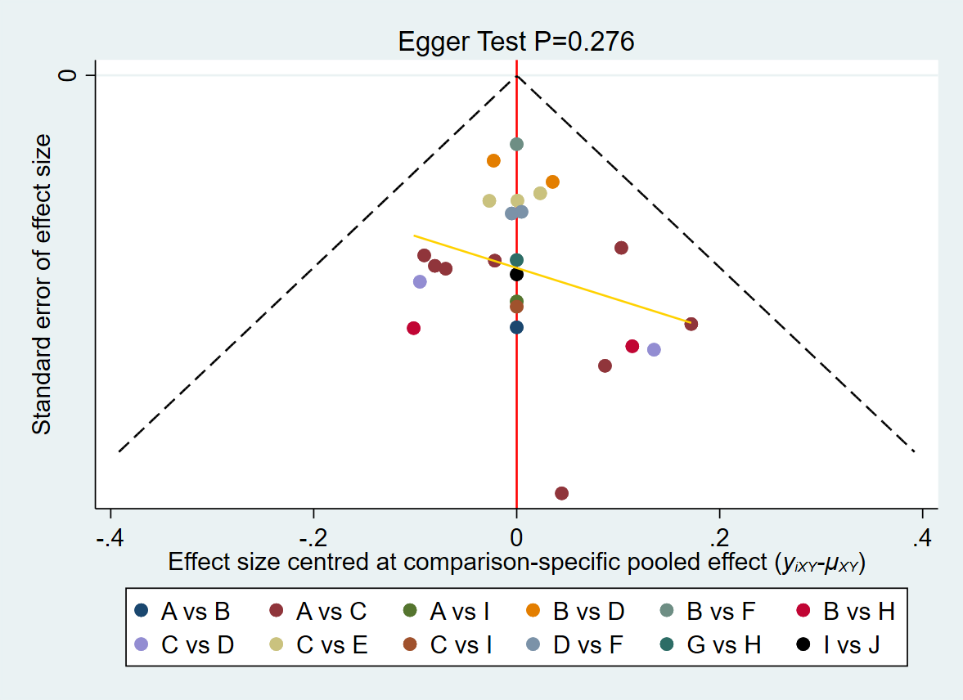


A=Granisetron; B=Granisetron+Dexamethasone; C=Ondansetron; D=Ondansetron+Dexamethasone; E=Palonosetro; F=Palonosetron+Dexamethasone; G=Ramosetrone; H= Ramosetron+Dexamethaso; I= Tropisetron; J= Tropisetron+ Dexamethasone

Funnel plot and the value of Egger’s test of acute complete control


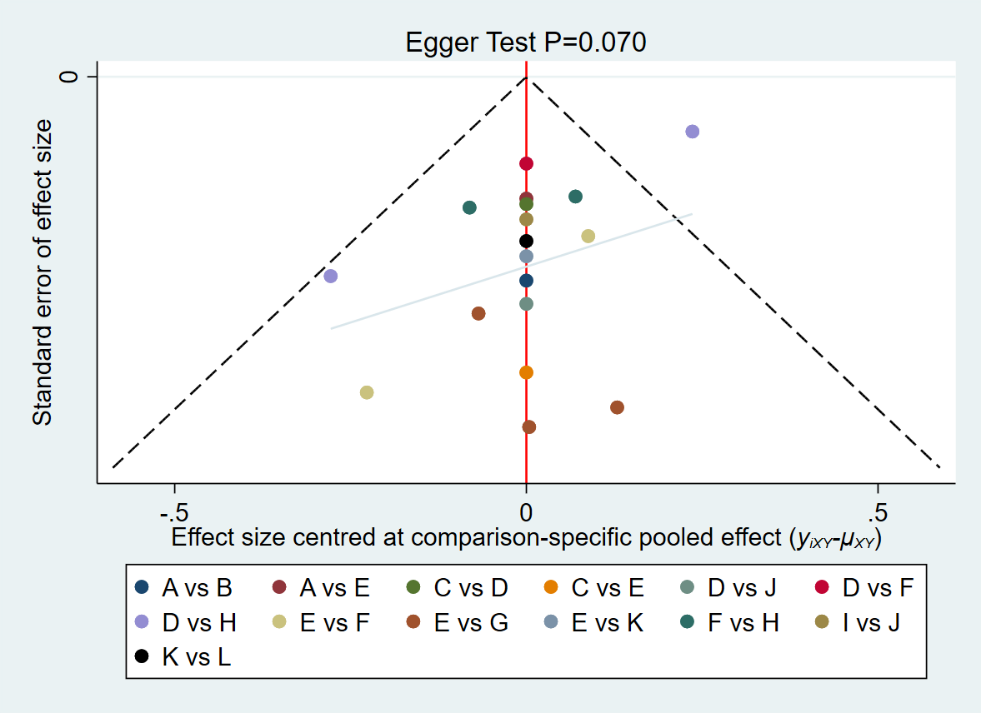


A=Dolasetron; B=Dolasetron+Dexamethasone; C=Granisetron; D=Granisetron+Dexamethasone; E=Ondansetron; F=Ondansetron+Dexamethasone; G=Palonosetro; H=Palonosetron+Dexamethasone; I=Ramosetrone; J= Ramosetron+Dexamethaso; K= Tropisetron; L= Tropisetron+ Dexamethasone.

Funnel plot and the value of Egger’s test of delayed nausea


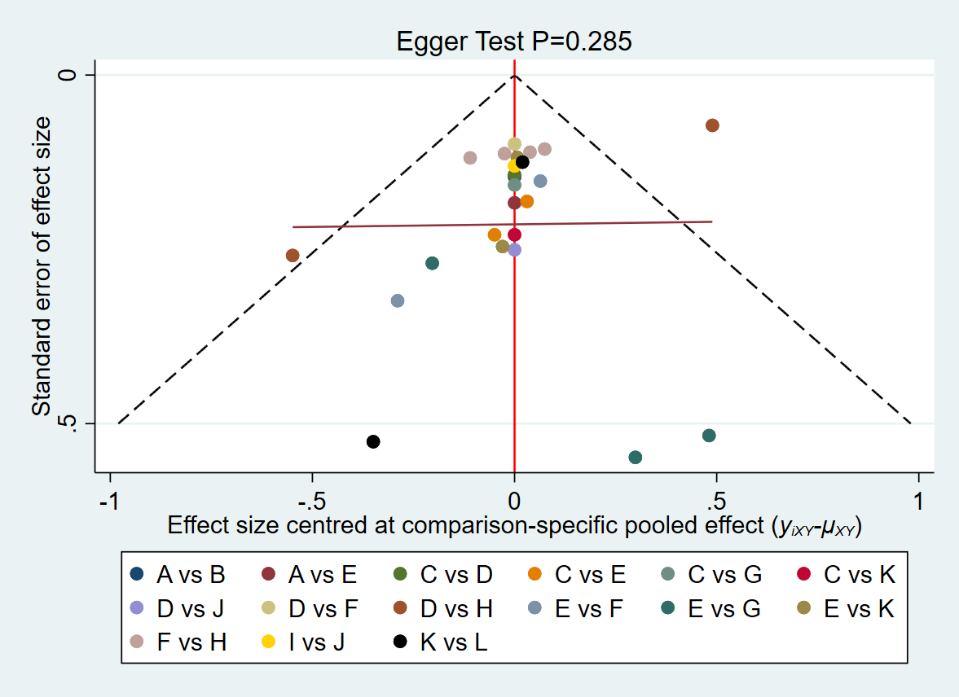


A=Dolasetron; B=Dolasetron+Dexamethasone; C=Granisetron; D=Granisetron+Dexamethasone; E=Ondansetron; F=Ondansetron+Dexamethasone; G=Palonosetro; H=Palonosetron+Dexamethasone; I=Ramosetrone; J= Ramosetron+Dexamethaso; K= Tropisetron; L= Tropisetron+ Dexamethasone.

Funnel plot and the value of Egger’s test of delayed vomiting


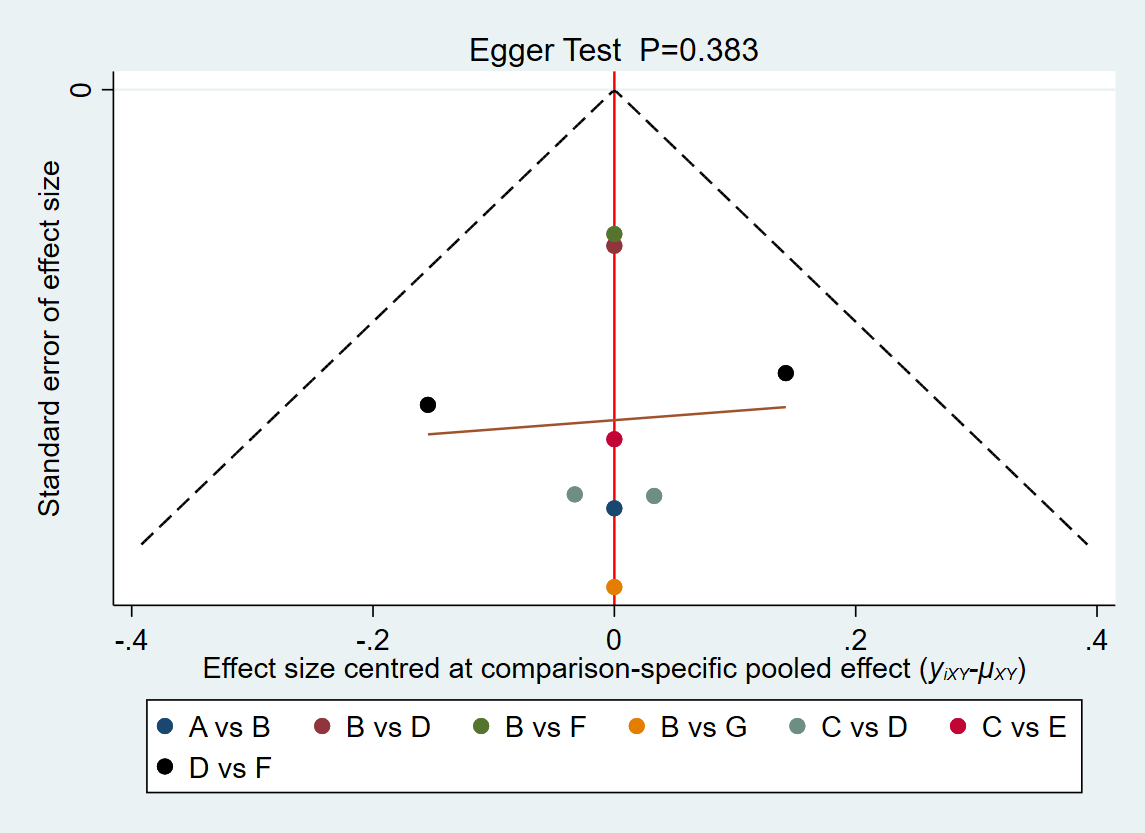


A=Granisetron; B=Granisetron+Dexamethasone; C=Ondansetron; D=Ondansetron+Dexamethasone; E=Palonosetro; F=Palonosetron+Dexamethasone; G= Ramosetron+Dexamethaso.

Funnel plot and the value of Egger’s test of delayed complete control


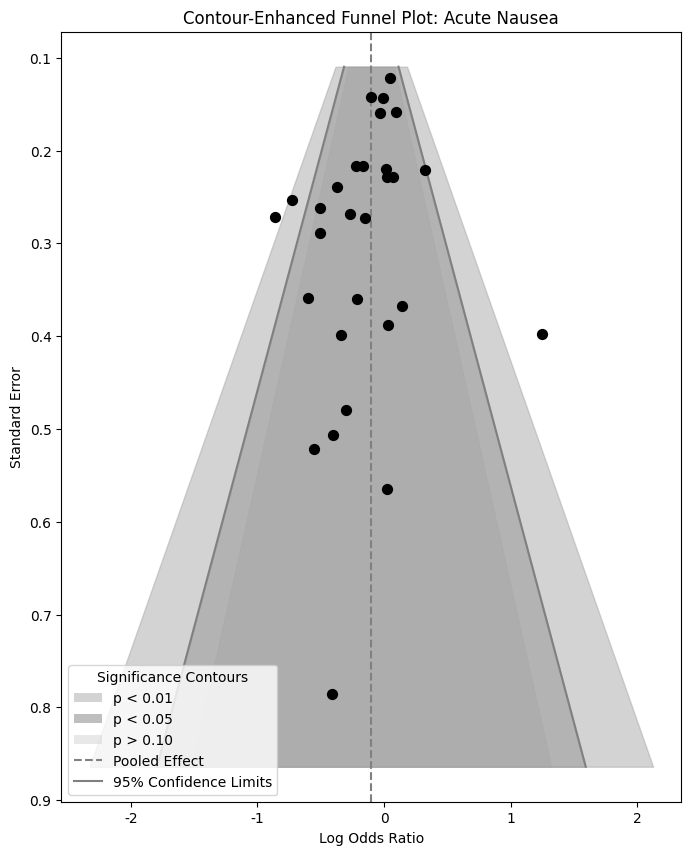


In Acute Nausea, most of the study points fall within the statistical significance region of $p<0.05$. The graph exhibits a certain degree of asymmetry, with some small‑sample studies located in the lower left part of the funnel plot and away from the summary effect line.

This asymmetry may suggest the presence of a small‑study effect or publication bias. However, since the majority of the study points lie within the significance region, such asymmetry could also arise from genuine heterogeneity—for instance, differences in study design or population characteristics among smaller studies.


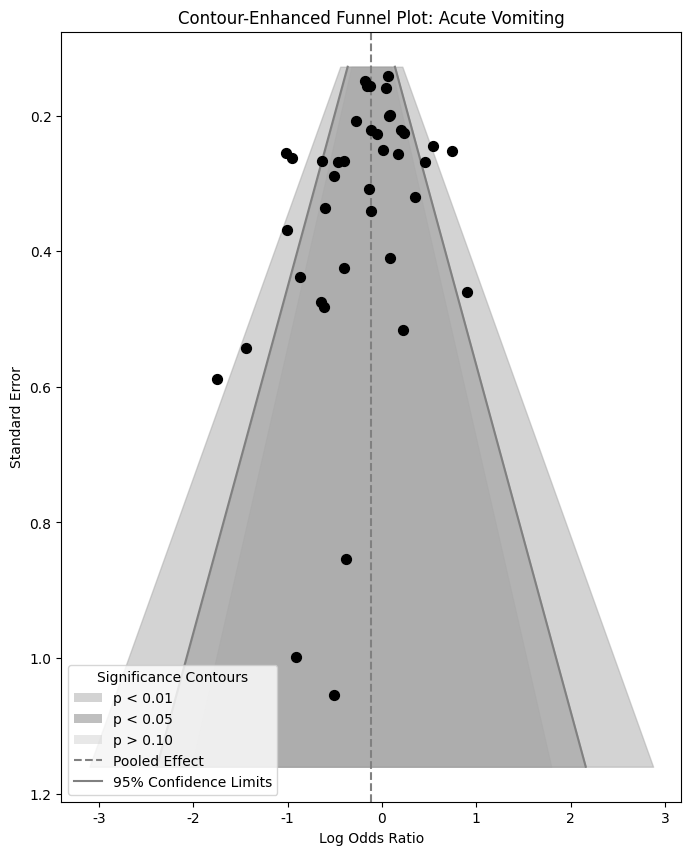


In Acute Vomiting, the study points are relatively evenly distributed, although a small number of small‑sample studies lie near the edges of the funnel plot. The majority of study points are concentrated within the statistical significance region of $p<0.05$.

The funnel plot shows no pronounced asymmetry, suggesting a low risk of publication bias.


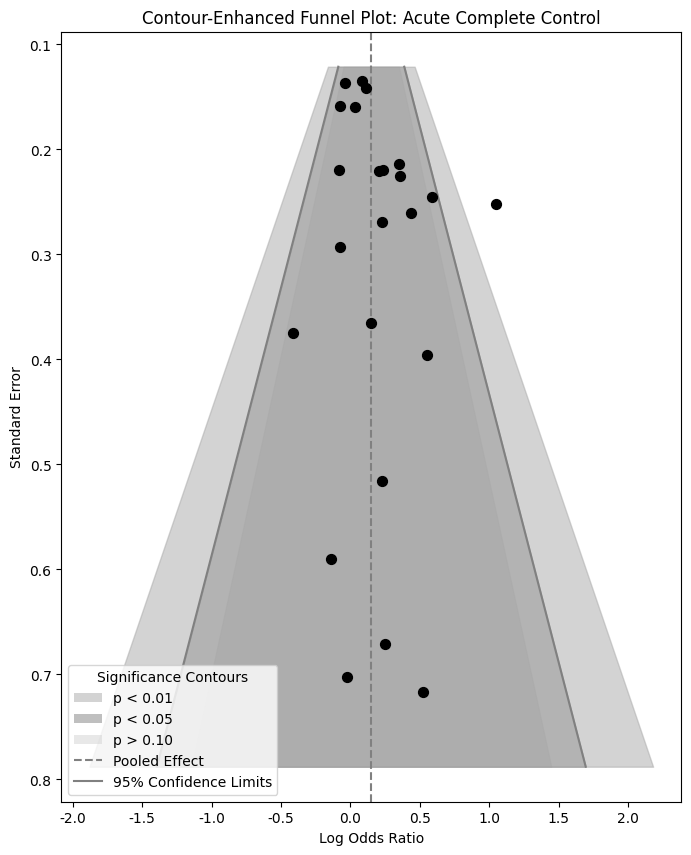


In Acute Complete Control, the study points are distributed in a fairly symmetric pattern, with the majority located within the highly significant region of $p<0.01$.
The funnel plot shows good symmetry, indicating a low risk of publication bias and supporting the robustness of the results.


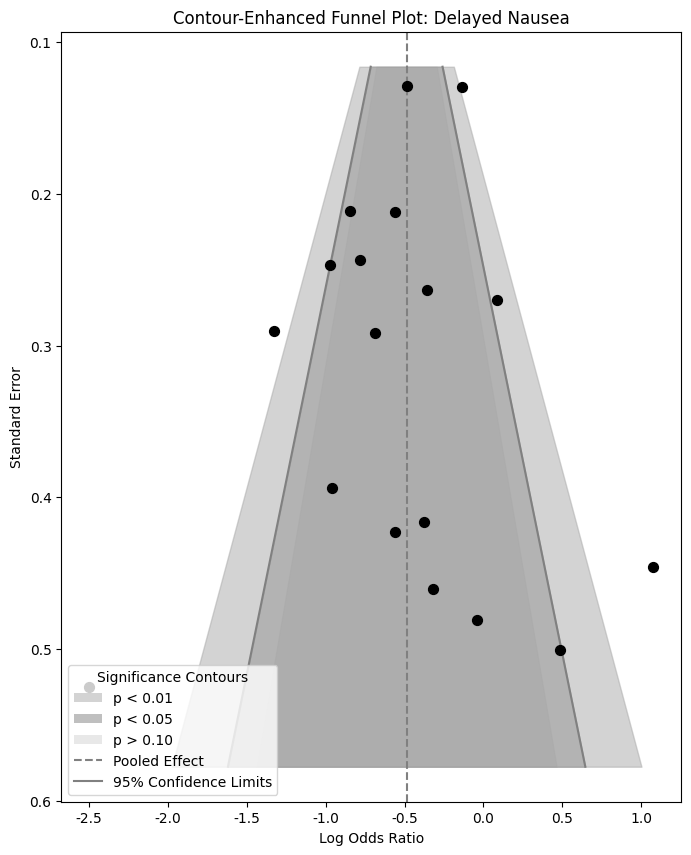


In Delayed Nausea, the graph exhibits a clear asymmetry, with some small-sample studies located on the left side of the funnel plot, away from the summary effect line.

This asymmetry may indicate the presence of publication bias. Given that this outcome measure itself shows moderate to high heterogeneity, such asymmetry could also reflect differences among studies in terms of intervention, population, or measurement methods.


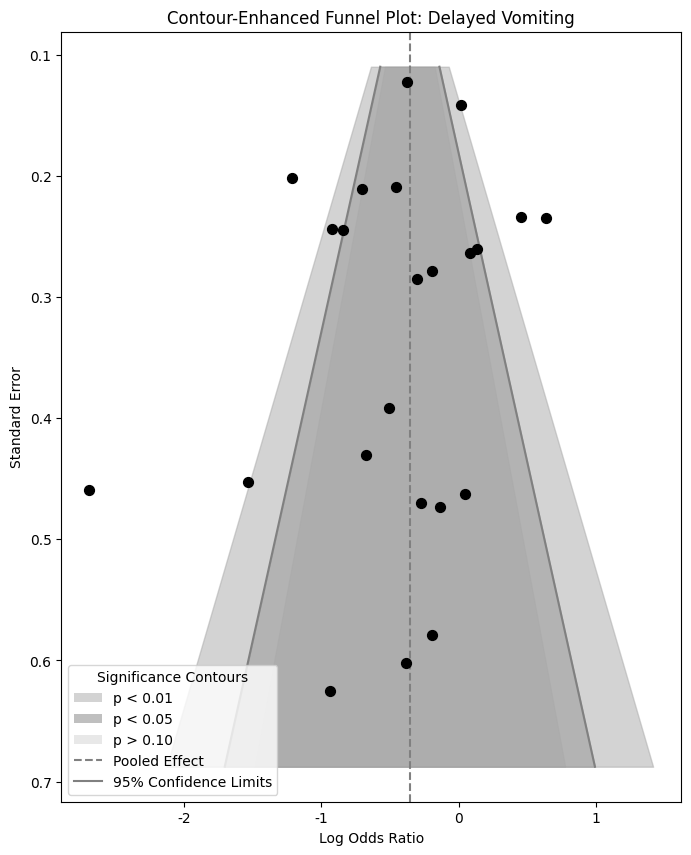


In Delayed Vomiting, the graph shows a certain degree of asymmetry, particularly in areas with larger standard errors (indicating small-sample studies).

This asymmetry should be interpreted in conjunction with the results of Egger's test. The contour lines indicate that most study points fall within the region of statistical significance, suggesting the findings are relatively robust; however, the potential influence of small-sample studies still warrants attention.
