# Supplementary material for: Comparative efficacy of 5-hydroxytryptamine-3 (5-HT3) receptor antagonists with or without dexamethasone for prevention of chemotherapy-induced nausea and vomiting following highly emetogenic chemotherapy (HEC): a network meta-analysis
Source: PeerJ. 2026 Apr 2;14:e21047. doi: 10.7717/peerj.21047 (PMC13050518; doi:10.7717/peerj.21047)
Supplement: Supplemental Information 18 [file peerj-14-21047-s018.docx]

Present the full search strategies for all databases, registers and websites, including any filters and limits used.

**PubMed**

((((((((((((("Ondansetron"[Mesh]) OR (((((((((((Ondansetron, (+,-)-Isomer[Title/Abstract]) OR (Ondansetron Hydrochloride[Title/Abstract])) OR (Hydrochloride, Ondansetron[Title/Abstract])) OR (Ondansetron Monohydrochloride[Title/Abstract])) OR (Monohydrochloride, Ondansetron[Title/Abstract])) OR (Ondansetron Monohydrochloride Dihydrate[Title/Abstract])) OR (Dihydrate, Ondansetron Monohydrochloride[Title/Abstract])) OR (Monohydrochloride Dihydrate, Ondansetron[Title/Abstract])) OR (Ondansetron, (S)-Isomer[Title/Abstract])) OR (Zofran[Title/Abstract])) OR (Ondansetron, (R)-Isomer[Title/Abstract]))) OR ("Granisetron"[Mesh])) OR (((((Kytril[Title/Abstract]) OR (Granisetron Hydrochloride[Title/Abstract])) OR (Hydrochloride, Granisetron[Title/Abstract])) OR (Granisetron Monohydrochloride[Title/Abstract])) OR (Monohydrochloride, Granisetron[Title/Abstract]))) OR ("dolasetron" [Supplementary Concept])) OR ((((dolasetron mesylate[Title/Abstract]) OR (dolasetron mesylate monohydrate[Title/Abstract])) OR (dolasetron mesilate monohydrate[Title/Abstract])) OR (Anzemet[Title/Abstract]))) OR ("Tropisetron"[Mesh])) OR (((Navoban[Title/Abstract]) OR (Indole 3 carboxylic Acid Tropine Ester[Title/Abstract])) OR (Tropisetron Hydrochloride[Title/Abstract]))) OR ("ramosetron" [Supplementary Concept])) OR ((ramosetron hydrochloride[Title/Abstract]) OR (Nasea[Title/Abstract]))) OR ("azasetron" [Supplementary Concept])) OR (("azasetron"[Supplementary Concept] OR azasetron, (+-)-isomer [Title/Abstract]) OR ("Palonosetron"[Mesh])) OR ((((((Palonosetron, (R-(R*,R*))-isomer[Title/Abstract]) OR (Palonosetron, (3R)-[Title/Abstract])) OR (Palonosetron, (R-(R*,S*))-isomer[Title/Abstract])) OR (Aloxi[Title/Abstract])) OR (Palonosetron, (S-(R*,S*))-isomer[Title/Abstract])) OR (Palonosetron Hydrochloride[Title/Abstract]))

**Embase**

(((ondansetron,:ab,ti AND +,-:ab,ti AND -isomer:ab,ti OR 'ondansetron hydrochloride':ab,ti OR 'hydrochloride, ondansetron':ab,ti OR 'ondansetron monohydrochloride':ab,ti OR 'monohydrochloride, ondansetron':ab,ti OR 'ondansetron monohydrochloride dihydrate':ab,ti OR 'dihydrate, ondansetron monohydrochloride':ab,ti OR 'monohydrochloride dihydrate, ondansetron':ab,ti OR (ondansetron,:ab,ti AND s:ab,ti AND -isomer:ab,ti) OR zofran:ab,ti OR (ondansetron,:ab,ti AND r:ab,ti AND -isomer:ab,ti)) OR ('ondansetron'/exp OR 'ondansetron') OR (kytril:ab,ti OR 'granisetron hydrochloride':ab,ti OR 'hydrochloride, granisetron':ab,ti OR 'granisetron monohydrochloride':ab,ti OR 'monohydrochloride, granisetron':ab,ti) OR ('dolasetron mesylate':ab,ti OR 'dolasetron mesylate monohydrate':ab,ti OR 'dolasetron mesilate monohydrate':ab,ti OR anzemet:ab,ti) OR ('tropisetron'/exp OR 'tropisetron') OR ('dolasetron mesilate'/exp OR 'dolasetron mesilate') OR ('granisetron'/exp OR 'granisetron') OR (navoban:ab,ti OR 'indole 3 carboxylic acid tropine ester':ab,ti OR 'tropisetron hydrochloride':ab,ti) OR ('ramosetron'/exp OR 'ramosetron') OR ('ramosetron hydrochloride':ab,ti OR nasea:ab,ti) OR ('azasetron'/exp OR 'azasetron') OR (azasetron,:ab,ti AND +-:ab,ti AND -isomer:ab,ti) OR ('palonosetron'/exp OR 'palonosetron') OR (palonosetron,:ab,ti AND r-:ab,ti AND r*,r*:ab,ti AND -isomer:ab,ti OR (palonosetron,:ab,ti AND 3r:ab,ti AND -:ab,ti) OR (palonosetron,:ab,ti AND r-:ab,ti AND r*,s*:ab,ti AND -isomer:ab,ti) OR aloxi:ab,ti OR (palonosetron,:ab,ti AND s-:ab,ti AND r*,s*:ab,ti AND -isomer:ab,ti) OR 'palonosetron hydrochloride':ab,ti)) AND [01-01-2025]/sd NOT [01-04-2025]/sd AND [<1966-2025]/py) OR (((((ondansetron,:ab,ti AND +,-:ab,ti AND -isomer:ab,ti OR 'ondansetron hydrochloride':ab,ti OR 'hydrochloride, ondansetron':ab,ti OR 'ondansetron monohydrochloride':ab,ti OR 'monohydrochloride, ondansetron':ab,ti OR 'ondansetron monohydrochloride dihydrate':ab,ti OR 'dihydrate, ondansetron monohydrochloride':ab,ti OR 'monohydrochloride dihydrate, ondansetron':ab,ti OR (ondansetron,:ab,ti AND s:ab,ti AND -isomer:ab,ti) OR zofran:ab,ti OR (ondansetron,:ab,ti AND r:ab,ti AND -isomer:ab,ti)) OR ('ondansetron'/exp OR 'ondansetron') OR (kytril:ab,ti OR 'granisetron hydrochloride':ab,ti OR 'hydrochloride, granisetron':ab,ti OR 'granisetron monohydrochloride':ab,ti OR 'monohydrochloride, granisetron':ab,ti) OR ('dolasetron mesylate':ab,ti OR 'dolasetron mesylate monohydrate':ab,ti OR 'dolasetron mesilate monohydrate':ab,ti OR anzemet:ab,ti) OR ('tropisetron'/exp OR 'tropisetron') OR ('dolasetron mesilate'/exp OR 'dolasetron mesilate') OR ('granisetron'/exp OR 'granisetron') OR (navoban:ab,ti OR 'indole 3 carboxylic acid tropine ester':ab,ti OR 'tropisetron hydrochloride':ab,ti) OR ('ramosetron'/exp OR 'ramosetron') OR ('ramosetron hydrochloride':ab,ti OR nasea:ab,ti) OR ('azasetron'/exp OR 'azasetron') OR (azasetron,:ab,ti AND +-:ab,ti AND -isomer:ab,ti) OR ('palonosetron'/exp OR 'palonosetron') OR (palonosetron,:ab,ti AND r-:ab,ti AND r*,r*:ab,ti AND -isomer:ab,ti OR (palonosetron,:ab,ti AND 3r:ab,ti AND -:ab,ti) OR (palonosetron,:ab,ti AND r-:ab,ti AND r*,s*:ab,ti AND -isomer:ab,ti) OR aloxi:ab,ti OR (palonosetron,:ab,ti AND s-:ab,ti AND r*,s*:ab,ti AND -isomer:ab,ti) OR 'palonosetron hydrochloride':ab,ti)) AND [01-01-2025]/sd NOT [01-04-2025]/sd AND [<1966-2025]/py) OR (((ondansetron,:ab,ti AND +,-:ab,ti AND -isomer:ab,ti OR 'ondansetron hydrochloride':ab,ti OR 'hydrochloride, ondansetron':ab,ti OR 'ondansetron monohydrochloride':ab,ti OR 'monohydrochloride, ondansetron':ab,ti OR 'ondansetron monohydrochloride dihydrate':ab,ti OR 'dihydrate, ondansetron monohydrochloride':ab,ti OR 'monohydrochloride dihydrate, ondansetron':ab,ti OR (ondansetron,:ab,ti AND s:ab,ti AND -isomer:ab,ti) OR zofran:ab,ti OR (ondansetron,:ab,ti AND r:ab,ti AND -isomer:ab,ti)) OR ('ondansetron'/exp OR 'ondansetron') OR (kytril:ab,ti OR 'granisetron hydrochloride':ab,ti OR 'hydrochloride, granisetron':ab,ti OR 'granisetron monohydrochloride':ab,ti OR 'monohydrochloride, granisetron':ab,ti) OR ('dolasetron mesylate':ab,ti OR 'dolasetron mesylate monohydrate':ab,ti OR 'dolasetron mesilate monohydrate':ab,ti OR anzemet:ab,ti) OR ('tropisetron'/exp OR 'tropisetron') OR ('dolasetron mesilate'/exp OR 'dolasetron mesilate') OR ('granisetron'/exp OR 'granisetron') OR (navoban:ab,ti OR 'indole 3 carboxylic acid tropine ester':ab,ti OR 'tropisetron hydrochloride':ab,ti) OR ('ramosetron'/exp OR 'ramosetron') OR ('ramosetron hydrochloride':ab,ti OR nasea:ab,ti) OR ('azasetron'/exp OR 'azasetron') OR (azasetron,:ab,ti AND +-:ab,ti AND -isomer:ab,ti) OR ('palonosetron'/exp OR 'palonosetron') OR (palonosetron,:ab,ti AND r-:ab,ti AND r*,r*:ab,ti AND -isomer:ab,ti OR (palonosetron,:ab,ti AND 3r:ab,ti AND -:ab,ti) OR (palonosetron,:ab,ti AND r-:ab,ti AND r*,s*:ab,ti AND -isomer:ab,ti) OR aloxi:ab,ti OR (palonosetron,:ab,ti AND s-:ab,ti AND r*,s*:ab,ti AND -isomer:ab,ti) OR 'palonosetron hydrochloride':ab,ti)) AND [<1966-2025]/py)) AND [<1966-2024]/py)

**Cochrane**

ID Search
#1 MeSH descriptor: [Ondansetron] explode all trees
#2 (Ondansetron, Isomer):ti,ab,kw OR (Ondansetron Hydrochloride):ti,ab,kw OR (Hydrochloride, Ondansetron):ti,ab,kw OR (Ondansetron Monohydrochloride):ti,ab,kw OR (Monohydrochloride, Ondansetron):ti,ab,kw (Word variations have been searched)
#3 (Ondansetron Monohydrochloride Dihydrate):ti,ab,kw OR (Dihydrate, Ondansetron Monohydrochloride):ti,ab,kw OR (Monohydrochloride Dihydrate, Ondansetron):ti,ab,kw OR (Ondansetron, (S) Isomer):ti,ab,kw OR (Zofran):ti,ab,kw (Word variations have been searched)
#4 (Ondansetron, (R) Isomer):ti,ab,kw (Word variations have been searched)
#5 MeSH descriptor: [Granisetron] explode all trees
#6 (Kytril):ti,ab,kw OR (Granisetron Hydrochloride):ti,ab,kw OR (Hydrochloride, Granisetron):ti,ab,kw OR (Granisetron Monohydrochloride):ti,ab,kw OR (Monohydrochloride, Granisetron):ti,ab,kw (Word variations have been searched)
#7 MeSH descriptor: [] explode all trees
#8 (dolasetron mesylate):ti,ab,kw OR (dolasetron mesylate monohydrate):ti,ab,kw OR (dolasetron mesilate monohydrate):ti,ab,kw OR (Anzemet):ti,ab,kw (Word variations have been searched)
#9 MeSH descriptor: [Tropisetron] explode all trees
#10 (Navoban):ti,ab,kw OR (Indole 3 carboxylic Acid Tropine Ester):ti,ab,kw OR (Tropisetron Hydrochloride):ti,ab,kw (Word variations have been searched)
#11 MeSH descriptor: [] explode all trees
#12 (ramosetron hydrochloride):ti,ab,kw OR (Nasea):ti,ab,kw (Word variations have been searched)
#13 MeSH descriptor: [] explode all trees
#14 (azasetron, isomer):ti,ab,kw (Word variations have been searched)
#15 MeSH descriptor: [Palonosetron] explode all trees
#16 (Palonosetron, (R (R*,R*)) isomer):ti,ab,kw OR (Palonosetron, (3R)):ti,ab,kw OR (Palonosetron, (R (R*,S*)) isomer):ti,ab,kw OR (Aloxi):ti,ab,kw OR (Palonosetron, (S (R*,S*)) isomer):ti,ab,kw (Word variations have been searched)
#17 (Palonosetron Hydrochloride):ti,ab,kw (Word variations have been searched)
#18 #1 or #2 or #3 or #4 or #5 or #6 or #7 or #8 or #9 or #10 or #11 or #12 or #13 or #14 or #15 or #16 or #17
#19 #1 or #2 or #3 or #4 or #5 or #6 or #7 or #8 or #9 or #10 or #11 or #12 or #13 or #14 or #15 or #16 or #17 with Cochrane Library publication date Between Jan 1950 and Mar 2025

**Web of Science**

"(((((((((((TS=(Ondansetron)) OR TS=(Ondansetron, (+,-)-Isomer)) OR TS=(Ondansetron Hydrochloride)) OR TS=(Hydrochloride, Ondansetron)) OR TS=(Ondansetron Monohydrochloride)) OR TS=(Monohydrochloride, Ondansetron)) OR TS=(Ondansetron Monohydrochloride Dihydrate)) OR TS=(Dihydrate, Ondansetron Monohydrochloride)) OR TS=(Monohydrochloride Dihydrate, Ondansetron)) OR TS=(Ondansetron, (S)-Isomer)) OR TS=(Zofran)) OR TS=(Ondansetron, (R)-Isomer) and Preprint Citation Index

"(((((TS=(Granisetron)) OR TS=(Kytril)) OR TS=(Granisetron Hydrochloride)) OR TS=(Hydrochloride, Granisetron)) OR TS=(Granisetron Monohydrochloride)) OR TS=(Monohydrochloride, Granisetron) and Preprint Citation Index

"((((TS=(Dolasetron)) OR TS=(dolasetron mesylate)) OR TS=(dolasetron mesylate monohydrate)) OR TS=(dolasetron mesilate monohydrate)) OR TS=(Anzemet) and Preprint Citation Index

"(((TS=(Tropisetron)) OR TS=(Navoban)) OR TS=(Indole 3 carboxylic Acid Tropine Ester)) OR TS=(Tropisetron Hydrochloride) and Preprint Citation Index

"((TS=(Ramosetron)) OR TS=(ramosetron hydrochloride)) OR TS=(Nasea) and Preprint Citation Index

"(TS=(Azasetron)) OR TS=(azasetron, (+-)-isomer) and Preprint Citation Index

"((((((TS=(Palonosetron)) OR TS=(Palonosetron, (R-(R, R))-isomer)) OR TS=(Palonosetron, (3R))) OR TS=(Palonosetron, (R-(R,S))-isomer)) OR TS=(Aloxi)) OR TS=(Palonosetron, (S-(R,S))-isomer)) OR TS=(Palonosetron Hydrochloride) and Preprint Citation Index

"#160 OR #128 OR #127 OR #123 OR #119 OR #109 OR #13 and Preprint Citation Index

"#163 and Preprint Citation Index 入库时间: 1950-01-01 to 2025-03-31
